# Balancing Stability and Payload Release in Glutathione-Responsive PROTAC Prodrugs Targeting Prostate Cancer

**DOI:** 10.1021/jacsau.6c00397

**Published:** 2026-06-10

**Authors:** Eleen Laul, Katherine A. Gosselé, Christian M. Matter, Wei-Hong W. Liu, Jorge A. González, Jason P. Holland, Amedeo Caflisch, Cristina Nevado

**Affiliations:** † Department of Chemistry, University of Zurich, Winterthurerstrasse 190, 8057 Zürich, Switzerland; ‡ Department of Biochemistry, University of Zurich, Winterthurerstrasse 190, 8057 Zürich, Switzerland

**Keywords:** prodrugs, PSMA, carbonate, kinetics, molecular dynamics, chameleonicity

## Abstract

BET-targeting PROteolysis TArgeting Chimeras (PROTACs) outperform their parent inhibitors in preclinical prostate cancer (PCa) models, yet their enhanced potency is expected to amplify dose-limiting on-target toxicities. To widen the therapeutic window, we designed a two-tier selective prodrug strategy enabling glutathione (GSH)-responsive and PCa-targeted delivery of the BET PROTAC **MZ1** caged via a carbonate moiety. Our design integrates a prostate-specific membrane antigen (PSMA) ligand with a GSH-cleavable disulfide linker to achieve tumor-associated activation. Systematic optimization of the commonly used but hydrolytically labile carbonate–disulfide motif led to secondary carbonate variant **2a** with markedly improved stability. In cellular systems, this analogue demonstrated strong disulfide dependence, confirming tight GSH control over prodrug activation. Importantly, while resistance to premature hydrolysis was significantly enhanced compared to the primary carbonate, GSH-mediated cleavage and the subsequent **MZ1**-releasing cyclization step proceeded with minimal kinetic penalty. In contrast, selective PSMA-mediated uptake could not be demonstrated. Molecular dynamics simulations revealed unexpected intramolecular folding that generates a compact prodrug conformation in which acidic functionalities are effectively masked. This structural feature may promote passive membrane permeability, which underscores the complexity of dual-targeting strategies. Collectively, our study establishes design principles for stable yet GSH-responsive carbonate–disulfide prodrugs and provides a framework for the rational development of PROTAC prodrugs, emphasizing the importance of coordinated optimization of their individual components to achieve predictable biological behavior.

## Graphical abstract

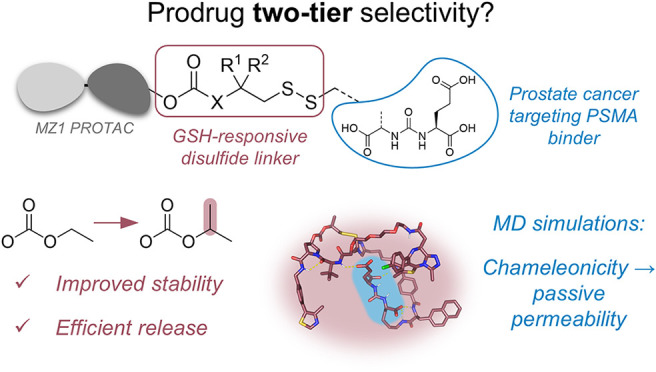

## Introduction

PROteolysis TArgeting Chimeras (PROTACs) are novel therapeutic modalities that induce the degradation of target proteins by hijacking the cellular proteolysis machinery.^1,2^ These heterobifunctional molecules simultaneously bind a protein of interest (POI) and an E3 ligase, forming a ternary complex that leads to the polyubiquitination and subsequent proteasomal degradation of the POI. In contrast to occupancy-based inhibitors, PROTACs achieve enhanced efficacy through the event-driven, catalytic degradation of entire proteins, resulting in sustained target removal at substoichiometric drug concentrations. These advantages are exemplified by PROTACs targeting bromo- and extraterminal-domain (BET) family proteins, which exhibited superior efficacy in preclinical prostate cancer (PCa) models compared to their parent inhibitors.^3,4^ However, these same properties can similarly amplify off-target effects and on-target toxicities in healthy cells. Given that the clinical application of BET inhibitors in cancer was restricted by dose-limiting on-target toxicities,^5^ BET-targeting PROTACs are likely to encounter similar constraints, limiting their therapeutic potential for the treatment of PCa.

Prodrug strategies have recently emerged to enhance the site selectivity of PROTACs in oncology. By exploiting tumor-associated features, such as overexpressed enzymes, hypoxia, reactive oxygen species, or cell surface antigens, prodrugs enable the conditional activation of potent yet systemically toxic molecules in target tissues.^6^ Prostate-specific membrane antigen (PSMA) is a cell surface protein whose highly restricted expression in advanced PCa enabled the clinical success of targeted radioligands such as Pluvicto (^177^Lu-PSMA-617; Novartis).^7–11^ Despite PSMA’s proven therapeutic relevance, only a handful of degraders have been reported that utilize PSMA binders for selective delivery to PCa.^12–14^ These remain limited in number and efficacy, highlighting a clear opportunity for innovation at the interface of targeted delivery and protein degradation. The cellular antioxidant glutathione (GSH), which is present at markedly elevated concentrations in tumor cells,^15,16^ is widely exploited for the intracellular release of prodrug payloads. GSH-cleavable linkers typically employ reducible disulfides combined with adjacent carbonate linkages.^17–19^ Although these carbonates facilitate efficient drug release, they are prone to premature cleavage through serum instability or esterase- and nonspecific hydrolysis.^20,21^ This trade-off between stability and responsiveness has limited their applicability, including in PROTAC prodrugs,^22,23^ underscoring the need to improve their pharmacological profile.

In this study, we aimed to achieve the PCa-selective and GSH-controlled release of a BET degrader by systematically optimizing a disulfide–carbonate linkage to balance biological stability and drug release. Using a model system based on a derivative of PSMA-617 and the BET PROTAC **MZ1**, we explored how chemical substitutions adjacent to the carbonate influence the kinetics of the individual steps of GSH-mediated drug release, resistance to undesired cleavage under various biological conditions, and degradation activity in PSMA-positive and PSMA-negative cell lines. The use of a secondary carbonate improved stability without compromising PROTAC release; however, PSMA-selective uptake could not be demonstrated. Supported by molecular dynamics simulations, we propose that the chameleonic nature of these high-molecular-weight small molecules facilitates their passive permeability into cells, thus limiting their PCa-selectivity.

## Results and Discussion

### Design and Synthesis of a PCa-Targeting MZ1 Prodrug Library

The highly potent BET family (BRD2, 3, and 4) degrader **MZ1**, a well-established but widely toxic PROTAC, was chosen as the basis of a model system to explore GSH-controlled delivery to PCa.^24^ This compound encompasses the Von Hippel-Lindau (VHL) E3 ligase ligand **VH032**, a poly­(ethylene glycol) (PEG) linker, and the BET-bromodomain (BRD) inhibitor **JQ1** (Scheme 1). For targeted delivery to PCa, we selected a small-molecule PSMA binder derived from PSMA-617 (Scheme 1). PSMA expression is highly enriched in the prostate, in contrast to the ubiquitous expression of BRD4, and is further elevated in PCa (Figure S1 in the Supporting Information (SI)). Binding of this ligand to PSMA will lead to the endocytosis of the receptor-prodrug complex, delivering **MZ1** selectively into PSMA-expressing cells.^25^ The PSMA ligand was covalently conjugated via a carbonate to the hydroxyproline of the VHL ligand. Addition of the prodrug moiety in this position ensures that **MZ1** remains inactive as a degrader until the prodrug is cleaved, as the free −OH group is critical for VHL binding. Inversion at this stereocenter is widely used to demonstrate the VHL-dependence of PROTACs by inhibiting VHL binding,^26^ and the hydroxyproline is deeply buried and pointing into the VHL binding pocket (Figure S2 in the SI). Although the **JQ1** pharmacophore of **MZ1** is unaffected and can therefore continue to act as a BRD inhibitor, conjugation to the large and highly acidic PSMA ligand should prevent prodrug uptake into non-target cells, thus reducing its toxicity.

**Scheme 1:**
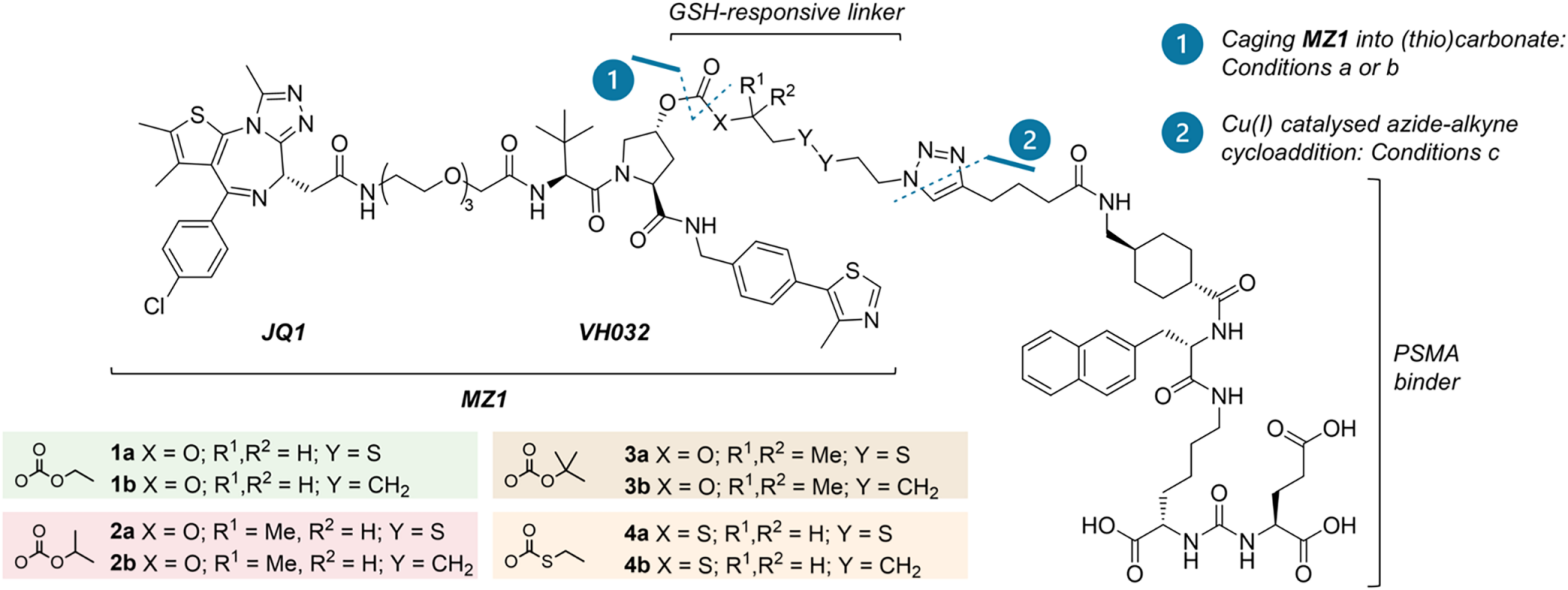
Optimizing Carbonate Prodrug Stability by Structural Modification of GSH-Responsive PROTAC Prodrugs^a^

a Structures of PCa-targeting GSH-responsive BET PROTAC prodrugs bearing a disulfide linker with a primary, secondary, or tertiary carbonate or thiocarbonate linkage (**1a**–**4a**) and the negative control models bearing an alkylic linker (**1b**–**4b**); reaction conditions for key transformations: (a) Triphosgene (0.4 or 0.35 equiv), linker (2 equiv), DMAP (3 equiv), DCM, 0 to 20 °C, 1 h; then formed chloroformate linker (2 equiv), **MZ1** (1 equiv), DCM, 0 to 20 °C, 15 h; (b) NaHMDS (0–1 equiv), 4-nitrophenyl carbonochloridate (1.1–2 equiv), DMAP (0.1–2 equiv), THF or DCM, 0 to 20 °C, 1–18 h; then formed (thio)­carbonate linker (1–2.2 equiv), **MZ1** (1–1.3 equiv), DMAP (1.5–3 equiv), DCM, 20 °C, 18–96 h; (c) azide (1 equiv), alkyne (1.5 equiv), CuSO_4_·5H_2_O (1.5 equiv), sodium ascorbate (1.5 equiv), TBTA (0.2 equiv), NMP, 20 °C, 16 h.

To facilitate both the intracellular release of **MZ1** and attain a second layer of tumor specificity, we conjugated the PSMA ligand through a GSH-responsive disulfide linker. In order to explore how modifications to the sterics and electronics of this linkage can affect prodrug performance, our medicinal chemistry campaign targeted three carbonate–disulfide prodrugs (**1a**–**3a**) featuring an increasing steric bulk around the carbonate moiety (X = O; Y = S; **1a**, R^1^ = R^2^ = H; **2a**, R^1^ = Me, R^2^ = H; **3a**, R^1^ = R^2^ = Me) as well as a thiocarbonate derivative (**4a**, X = S; Y = S; R^1^ = R^2^ = H) (Scheme 1).^27,28^ These compounds were synthesized by caging **MZ1** into a (thio)­carbonate using a nonsymmetric disulfide linker functionalized with either an alcohol or a thiol on one end, and an azide group on the other (Disconnection 1 in Scheme 1). The azide group was then used in a Cu­(I)-mediated cycloaddition (CuAAC) with an alkyne-functionalized PSMA ligand (Disconnection 2 in Scheme 1). To assess the disulfide dependence of prodrug activity, four negative control compounds featuring a noncleavable alkylic chain (Y = CH_2_) in place of the GSH-responsive disulfide linker were also synthesized in an analogous manner (**1b**–**4b** in Scheme 1; for experimental details, see SI Section 4).

### Carbonate Linkage Substitutions Affect *Ex Cellulo* Stability

First, we determined the susceptibility of disulfide prodrugs **1a**–**4a** to undesired esterase-mediated hydrolysis (Figure 1A and S3A in SI). While the primary carbonate **1a** was rapidly cleaved to release **MZ1**, the addition of a single methyl group in **2a** partially stabilized the compound. Continuing this trend, tertiary carbonate **3a** was completely resistant to esterases under the tested conditions but exhibited partial cleavage through esterase-independent mechanisms. The altered electronics of the thiocarbonate in **4a** also significantly improved the resistance to esterases relative to **1a**. As expected, analogous results were observed for the negative control **1b**–**4b** (Figure S3B in SI). Similar trends were apparent in the presence of fetal bovine serum (FBS)—a common and essential additive to cell culture media (Figures 1B and S3C in SI for negative controls **1b**–**4b**). Primary carbonate **1a** exhibited mild hydrolysis (∼10% of **MZ1** released), whereas stability was improved with increased steric hindrance (**2a**) and the use of thiocarbonate (**4a**), both of which almost completely prevented **MZ1** release. As with esterase-mediated hydrolysis, tertiary carbonate **3a** showed serum-independent **MZ1** release. Overall, our *ex cellulo* results clearly indicate that careful tuning of steric and electronic factors can improve the biological stability of carbonate-conjugated prodrugs.

**Figure 1:**
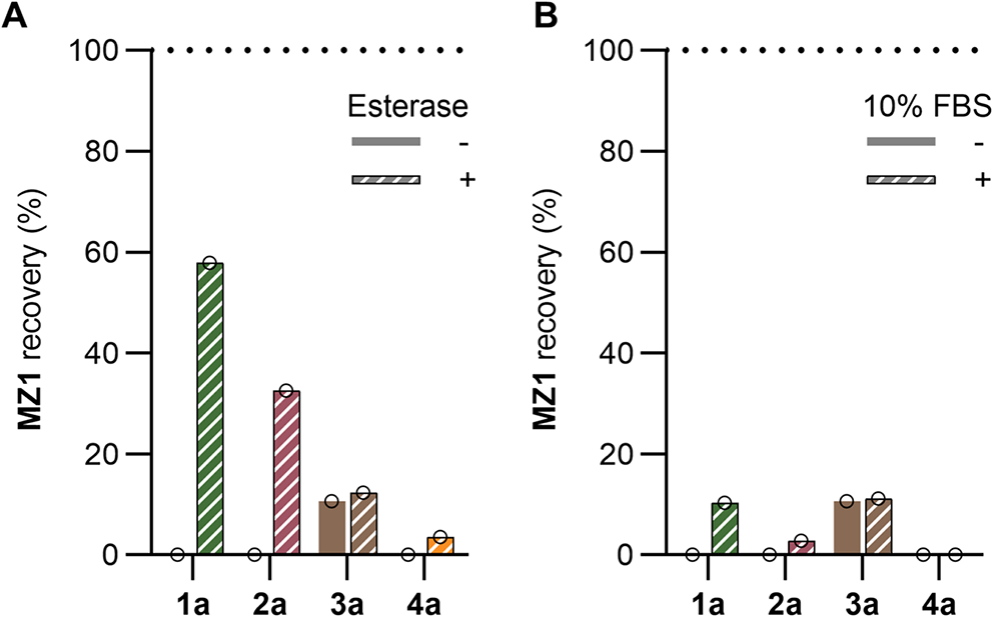
Assessment of e*x cellulo* biological stability of prodrugs **1a**–**4a**. (A) Recovery of **MZ1** upon the incubation of **1a**–**4a** (20 μM) in the presence or absence of porcine liver esterase (1 U/mL) after 4 h (pH 7.4, 37 °C), samples analyzed after protein precipitation by UHPLC-MS. (B) Recovery of **MZ1** upon the incubation of **1a**–**4a** (10 μM) in PBS in the presence or absence of 10% FBS after 4 h (pH 7.4, 37 °C), samples analyzed after protein precipitation by UHPLC-MS.

### Carbonate Linkage Substitutions Affect Kinetics of GSH-Mediated MZ1 Release

In prodrugs, biological stability and efficient cargo release must be well-balanced. However, the kinetics of the individual steps leading to GSH-mediated drug release from disulfide-carbonate conjugates and their dependence on structural modifications remain poorly studied. To address this gap, disulfides **1a**–**4a** were incubated with a 200-fold excess of GSH, and the reactions were monitored by UHPLC-MS (Figures 2A–D and S4–S7). All compounds displayed a pseudo-first-order decay (Figure S8 in SI section 3), for which the observed rate constants (*k*_obs_) were determined and normalized to the primary carbonate (**1a**). Thiocarbonate **4a** showed a nearly identical rate to **1a** (*k*_obs_ = 6.39 × 10^–4^ s^–1^ and 6.50 × 10^–4^ s^–1^, respectively), but the secondary **2a** (3.70 × 10^–4^ s^–1^) and tertiary carbonate **3a** (2.37 × 10^–4^ s^–1^) revealed 42 and 63% slower decay, respectively. In parallel, initial rates for **MZ1** release (v_0_) were also determined (Figures 2A–D and S9 in the SI Section 3). The tertiary carbonate **3a** released **MZ1** at nearly the same rate as **1a** (v_0_ = 2.09 × 10^–9^ M·s^–1^ and 1.99 × 10^–9^ M·s^–1^, respectively). The secondary carbonate **2a** (v_0_ = 1.52 × 10^–9^ M·s^–1^) was 24% slower, whereas the release rate from thiocarbonate **4a** (v_0_ = 9.31 × 10^–11^ M·s^–1^) was negligible. Promisingly, increasing GSH levels accelerated both prodrug decay and **MZ1** release for all compounds, supporting the intended tumor-selective activation mechanism (Figure S10 in SI). Overall, these results emphasize that structural differences affect the rates of GSH-mediated decay and cargo release, not only to a different extent but also distinctly between compounds.

**Figure 2:**
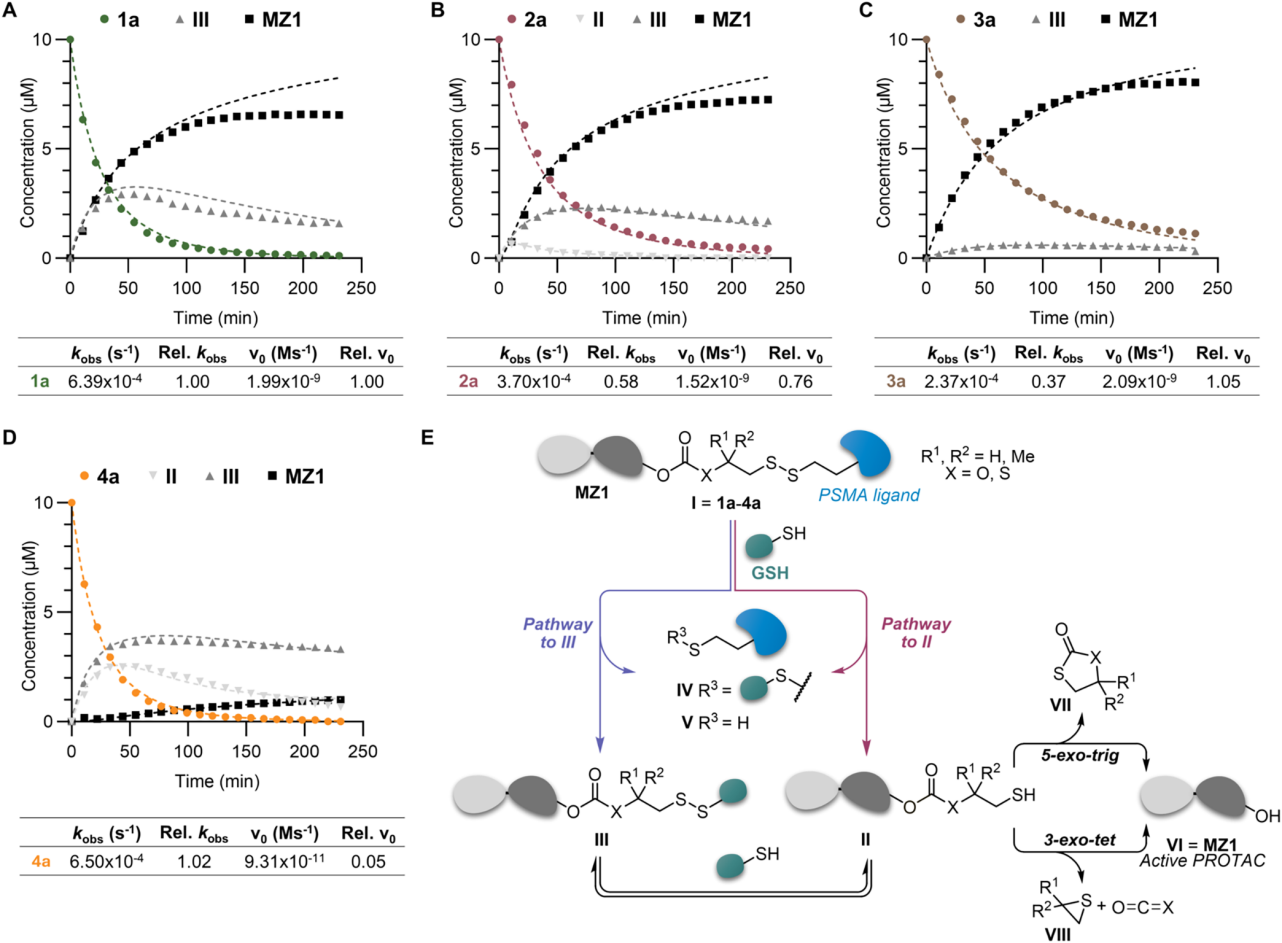
GSH-mediated disulfide-prodrug cleavage mechanism and kinetics. (A–D) Experimental data of GSH-mediated **MZ1** release from compounds **1a**–**4a** (10 μM) in the presence of GSH (2 mM) (pH 7.4, 37 °C), reaction was monitored and analyzed by UHPLC-MS (UHPLC-MS traces and MS spectra in Figures S4–S7 in SI); Intermediates **II** and **III** are introduced in section E; dotted lines correspond to the simulated concentration of each species using Dynochem software (simulation constants represented in Table S1 in SI Section 3); the pseudo-first-order observed rate constants (*k*_obs_) of prodrug decay were obtained within the linear section of the curve (initial 50–60% conversion; Figure S8 in SI Section 3) and initial rate (v_0_) of **MZ1** release obtained within the linear section of the curve (for **1a**–**3a** 27–30% conversion, for **4a** 5% conversion; Figure S9 in SI Section 3); the obtained rate constants and initial rates were normalized to the primary carbonate **1a** (Rel. *k*_obs_ and Rel. v_0_). (E) Expected cleavage mechanism and all intermediates of the disulfide-containing PCa-targeting PROTAC prodrugs **1a**–**4a**.

To shed light on these discrepancies, the elementary steps in the release mechanism were simulated with Dynochem software (dotted lines in Figure 2A–D and Table S1 in SI Section 3). The expected mechanism of GSH-dependent cargo release is depicted in Figure 2E. First, GSH reduces the disulfide bond of the prodrugs (**I**) to form either the key thiol **II** or the GSH-containing intermediate **III**, while releasing the GSH-adduct **IV** or thiol **V** (pathways red and blue), respectively.^29^ With a second molecule of GSH, intermediate **III** can also be converted into **II**—the immediate precursor for cargo release. Interestingly, GSH-adduct **III** can be observed in the reaction mixture for all compounds, whereas free thiol **II** is detected only for secondary carbonate **2a** and thiocarbonate **4a** (Figure 2A–D). To release the active PROTAC **MZ1** (**VI**), thiol **II** undergoes either a *5-exo-trig* or *3-exo-tet* cyclization, simultaneously generating either oxa/dithiolanone (**VII**) or thiirane (**VIII**) derivatives.

Our initial analysis focused on the two possible pathways of prodrug cleavage by GSH that yield intermediate **II** (red) or **III** (blue). The simulated rates of cleavage to intermediate **II** were similar for all prodrugs (*k*_I→II_ = 0.13–0.15 M^–1^·s^–1^; Table S1 in SI Section 3). However, variability in rates en route to **III** could explain the relative differences in the observed decay rates. While the calculated rate constants for the conversion of primary (**1a**) and thiocarbonate (**4a**) to intermediate **III** were comparable (*k*_I→III_ = 0.18 and 0.22 M^–1^·s^–1^, respectively), the rate for secondary carbonate (**2a**) was 2-fold slower (*k*_I→III_ = 0.10 M^–1^·s^–1^), and an even larger drop (ca. 10-fold) was observed for tertiary carbonate **3a** (*k*_I→III_ = 0.02 M^–1^·s^–1^). Next, we investigated the parameters underpinning the experimentally determined differences in the rate of cargo release for **1a**–**4a**, focusing on the conversion of intermediate **III** to **II** and the subsequent release of **MZ1** (**VI**) via the key cyclization of **II**. Simulated rates of **MZ1** release showed low sensitivity toward the parameters governing the conversion of **III** to **II**: except for **4a**, the kinetic profile was insensitive to the equilibrium constant for the interconversion of **II** and **III** (Table S1 in SI Section 3); with **4a** the equilibrium is shifted toward compound **III** (*K*_eq_ = 0.3), but the cyclization rate of **II** is much slower making this preceding equilibrium less relevant to the kinetics of **MZ1** release. In contrast, the combined rates of the *5-exo-trig* and *3-exo-tet* cyclizations for intermediate **II** (*k*_II→VI_) showed major differences between compounds. Relative to **1a** (*k*_II→VI_ = 0.1 s^–1^), cyclization and **MZ1** release were 300-fold slower in the secondary carbonate **2a** (*k*_II→VI_ = 3 × 10^–4^ s^–1^) and 2200-fold slower in the thiocarbonate **4a** (*k*_II→VI_ = 4.5 × 10^–5^ s^–1^), while tertiary carbonate **3a** exhibited a rate (*k*_II→VI_ = 0.1 s^–1^) comparable to that of **1a**, consistent with the differences in observed **MZ1** release (v_0_).

Together, these data suggest that steric and electronic factors govern the kinetics of both prodrug activation by GSH and **MZ1** release. Increasing steric bulk (**2a**, **3a**) slowed substrate consumption, a trend reflected in the simulated rate constants for the pathway involving GSH interaction with the sulfur atom closest to the methyl substituents, leading to intermediate **III**. However, the initial rates (v_0_) for **MZ1** release, defined by the cyclization of intermediate **II**, did not reflect the same relative trends and thus cannot be explained by steric effects alone. Compared to **1a**, the secondary carbonate **2a** cyclized more slowly. Since the attack on the trigonal (sp^2^) carbon in the *5-exo-trig* pathway is less affected by steric bulk, the reduced rate likely reflects the steric hindrance of the alternative attack on the tetragonal (sp^3^) carbon in the *3-exo-tet* pathway. In contrast, and despite additional steric hindrance, which likely further slows the *3-exo-tet* pathway, the geminal dimethyl groups in tertiary carbonate **3a** appear to accelerate the *5-exo-trig* cyclization via Thorpe-Ingold effects, such that the combination of the two routes is faster than for **2a**. Overall, the initial slow reactivity of **3a** with GSH is overcome by these fast cyclization kinetics, resulting in an **MZ1** release rate comparable to that of **1a**. Even though thiocarbonate **4a** showed the most efficient activation by GSH, it exhibited the poorest release of **MZ1** from intermediate **II**, which is indeed observable in the reaction media throughout the experiment (Figure 2D). This could arise either from different electronics on the carbon sp^2^ atom or, more likely, from the perpetual self-generation of intermediate **II** by the extrusion of the free thiol rather than the cargo alcohol in the cyclization step.

### Cellular Prodrug Activity Studies

To examine if the observed differences in *ex cellulo* stability and **MZ1** release translate to the *in cellulo* activity of our probes, we selected the PSMA-positive PCa line LNCaP as a model system along with the PSMA-negative PCa line PC3 as a control to explore prodrug PSMA dependence. As expected, a FITC-conjugated derivative of our prodrug’s PSMA ligand (**PSMA-FITC**) bound strongly to LNCaP but not PC3 cells (Figure S11A,B in SI). This binding to LNCaP cells could be reduced by blocking with **PSMA-Cbz**, our PSMA-targeting ligand appended with a Cbz group (Figure S11C–D in SI), or by siRNA-mediated knockdown of *folh1*, the gene encoding PSMA (Figure S11E in SI). In addition, we could observe the uptake of **PSMA-FITC** into LNCaP cells (Figure S11F in the SI), demonstrating the ability of our chosen ligand to guide compound internalization into PSMA-positive cells.

In both LNCaP and PC3 cells, **MZ1** has a stronger effect on cell viability than **JQ1**, the BET-BRD ligand on which **MZ1** is based (Figure S11G,H in the SI). As this indicates that protein degradation contributes to the antiproliferative activity of **MZ1**, we began by determining the effect of our prodrug series on LNCaP cell viability (Figure 3A). A robust antiproliferative effect comparable to **MZ1** (GI_50_ = 0.048 μM) was observed with the disulfide-bearing carbonates **1a**, **2a**, and **3a** (GI_50_ = 0.084, 0.076, and 0.061 μM, respectively), indicating successful **MZ1** release from these prodrugs. In contrast, thiocarbonate **4a** was 10-fold less potent (GI_50_ = 0.437 μM), consistent with its high stability *ex cellulo* and its subsequent sluggish cyclization-mediated release. The alkylic negative controls **1b** and **3b** exhibited similar behavior to their disulfide counterparts (GI_50_ = 0.131 and 0.098 μM, respectively), confirming that poor prodrug stability results in GSH-independent **MZ1** release *in cellulo*. Nevertheless, the secondary carbonate control **2b** was notably less potent than its disulfide analogue **2a** (GI_50_ = 0.345 μM), demonstrating that the activity of **2a** requires the disulfide and is thus most likely controlled by the GSH-mediated cleavage of its bioresponsive linker. Detailed examination of the dose–response curves showed that the maximal effect of **2b** on cell viability is reduced compared to that of **2a** and **MZ1** but is similar to that of **JQ1**, suggesting less protein degradation (Figure 3B).

**Figure 3:**
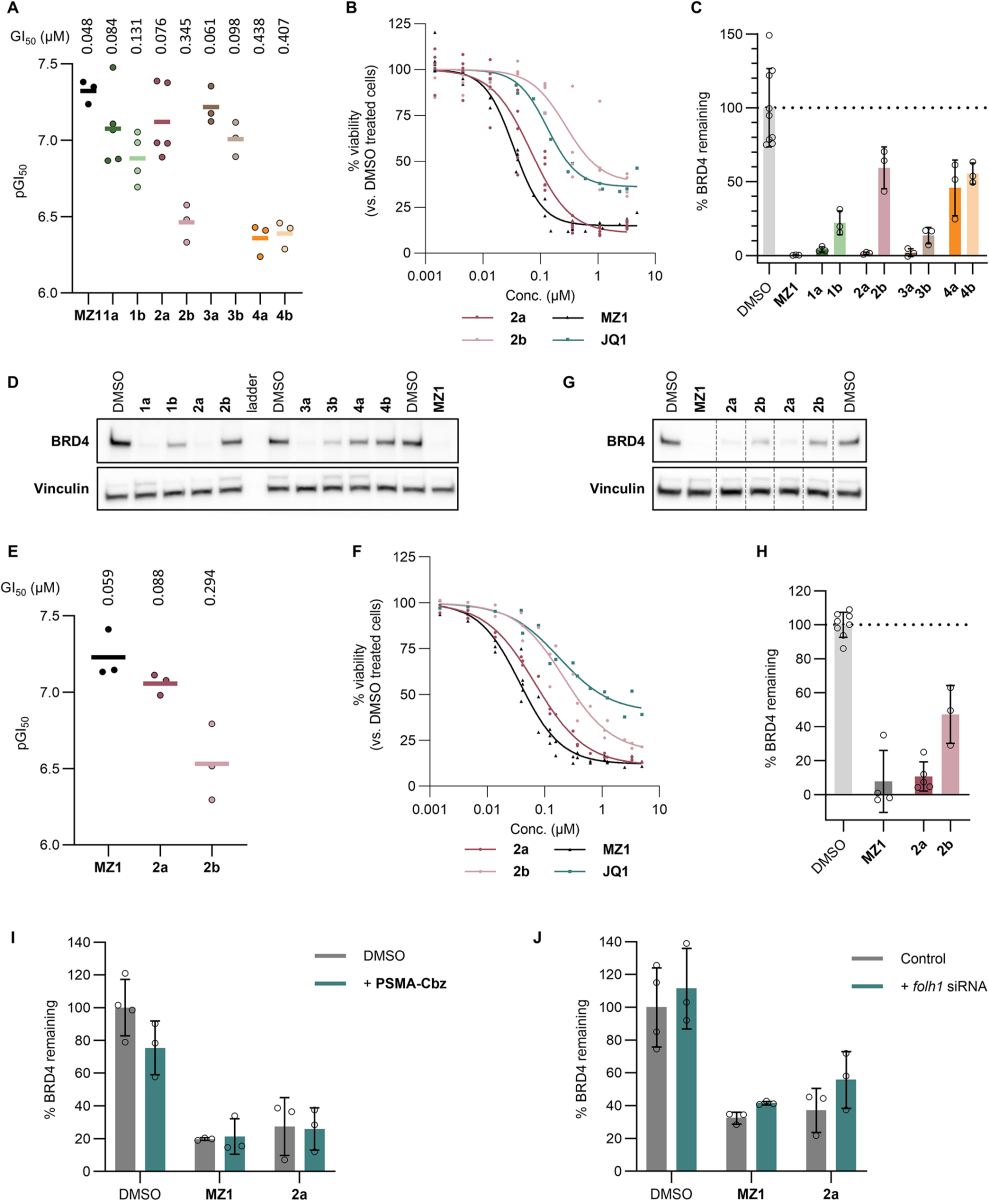
Assessment of the cellular activity of prodrugs **1a**–**4a**, the negative analogues **1b**–**4b** and **MZ1** in LNCaP and PC3 cells. (A, B) Antiproliferative effect in LNCaP cells following a three-day treatment. (A) pGI_50_ = −log_10_(GI_50_), where GI_50_ corresponds to the compound concentration required to reduce cell viability by 50% relative to DMSO-treated cells. One data point per experimental run, bar, and displayed GI_50_ show geometric mean of replicates; full dose response curves are shown in Figure S12 in SI. (B) Dose response curves, calculated from displayed points which represent the average viability of replicates within a single experimental run, individual curves in Figure S13 in SI. (C, D) BRD4 degradation in LNCaP cells, following a 4 h treatment with 100 nM compounds. Degradation was determined by Western blotting, using Vinculin as a loading control, and percentage remaining was calculated by comparison to DMSO-treated cells run on the same gel. (C) Quantification of all replicates and (D) a representative blot. See Figure S14 in SI for images of all blots used for quantification. (E, F) Antiproliferative effect in PC3 cells following a three-day treatment. Data processed and displayed as for (A, B), full dose response curves in Figures S16 and S17 in SI. (G, H) BRD4 degradation in PC3 cells, following a 4 h treatment with 100 nM compounds. (G) A representative blot and (H) quantification of all replicates. Data processed and displayed as for (C), images for all blots used for quantification in Figure S18 in SI. (I) BRD4 degradation in LNCaP cells, following 30 min pre-treatment with 10 μM **PSMA-Cbz** and 2 h treatment with 10 nM **MZ1**/**2a**. Data processed and displayed as for (C), images for all blots used for quantification in Figure S19 in SI. (J) BRD4 degradation in LNCaP cells treated for 2 h with 10 nM **MZ1**/**2a**, 72 h after siRNA-mediated knockdown of *folh1* (or treatment with non-targeting siRNA = control). Data processed and displayed as for (C), images for all blots used for quantification in Figure S20 in SI.

The direct measurement of BRD4 protein levels by Western blotting displayed similar trends between compounds, confirming that the observed differences in antiproliferation stem from BRD4 degradation (Figure 3C–D). Similarly to **MZ1**, all of the disulfide compounds except for the thiocarbonate (**1a**, **2a**, and **3a**) completely degraded BRD4, with the secondary carbonate alkylic control (**2b**) showing the greatest reduction in activity relative to its disulfide counterpart, although mild degradation was still detectable. At a lower concentration, where **MZ1** and **2a** only partially degraded BRD4, they did so to the same extent, demonstrating the highly efficient release of **MZ1** from this prodrug (Figure S15A in the SI). Together, and as predicted by the *ex cellulo* studies, our cellular results demonstrate that the secondary carbonate scaffold exhibits the most advantageous balance between efficient **MZ1** release and biological stability, successfully enabling the disulfide-dependent release of **MZ1** in cells.

To assess the PSMA-dependency, we profiled the activity of **2a** and **2b** in the PSMA-negative PCa cell line PC3. Although the substantial difference in activity between **2a** and **2b** was maintained, **2a** unexpectedly exhibited activity comparable to **MZ1** both in antiproliferation (GI_50_ [**MZ1**] = 0.059 and GI_50_ [**2a**] = 0.088 μM; Figure 3E,F) and in BRD4 degradation assays (Figures 3G,H and S15B in SI). Since no clear loss of **2a** activity was observed in PSMA-negative cells, we further blocked PSMA-mediated uptake of **2a** in LNCaP cells: either by saturating the receptor with excess **PSMA-Cbz**, or by siRNA-mediated knockdown of *folh1*. Neither approach reduced BRD4 degradation by **2a**, confirming a PSMA-independent uptake (Figure 3I,J). Our *ex cellulo* studies indicate that **2a** is stable in cellular media, making substantial extracellular release of **MZ1** from **2a** unlikely, although this cannot be completely excluded due to the different compound concentrations used *in* and *ex cellulo*. Based on these observations, we hypothesized that an alternative pathway beyond PSMA-guided endocytosis exists for the (passive) cellular permeability of **2a**, despite its high molecular weight and negative charge, delivering it to the intracellular milieu where GSH acts to release **MZ1**.

### Molecular Dynamics Simulations Reveal Intramolecular Folding

PROTACs commonly display better cellular permeability than predicted by methods such as Lipinski’s rule of five.^30^ This has been attributed to chameleonic properties of PROTACs, where, in nonpolar environments, hydrophobic collapse and masking of polar groups through intramolecular folding permit the passive permeability of these high-molecular-weight compounds.^31–35^ Given that our prodrugs have an increased opportunity for intramolecular folding compared to PROTACs, we hypothesized that, within the cell membrane, they might adopt a compact structure in which the carboxylic acid groups of the PSMA ligand are masked, enabling passive permeability into cells. Indeed, several observations throughout our work hinted at intramolecular folding occurring in an aqueous environment (Figure S21 in SI).

To explore this hypothesis, we performed multiple molecular dynamics (MD) simulations of **2a** in aqueous buffer as well as in chloroform to mimic the apolar environment of the inner cell membrane. The cumulative sampling was 10.4 and 26.2 μs in aqueous buffer and chloroform, respectively (see Materials and Methods in SI). In both cases, the distances between representative carbons on the PSMA ligand and either **JQ1** or **VH032** (C35, C88, and C94, respectively, Figure 4A) are relatively short, revealing extensive folding between these rather hydrophobic sites of the molecule. The solvent plays a key role, with **2a** forming different compact structures in aqueous buffer and chloroform (Figure 4B–C). These differences have large effects on the solvent accessible surface area (SASA) around the triply charged Glu-CO-Lys urea moiety of the PSMA binder, leaving this tricarboxylate solvent exposed in aqueous buffer, whereas it is predominantly shielded from the solvent in chloroform (Figure 4D). After single-linkage clustering of all frames from the MD simulations, visual inspection of representative structures of the most populated cluster in each solvent revealed that in aqueous buffer, **2a** undergoes a hydrophobic collapse of the **JQ1**, **VH032**, and naphthalene moieties while leaving the tricarboxylate solvent exposed (Figure 4E). As the PSMA ligand binding pocket is accessed through a long tunnel (Figure S22 in SI), the bulkiness of the folded compound around the naphthalene could inhibit binding of **2a** to PSMA. In contrast, in chloroform, the tricarboxylate can engage in extensive intramolecular hydrogen bonding, resulting in a compact structure with a generally hydrophobic surface (Figure 4F). This conformation may allow **2a** to passively penetrate apolar lipid membranes, explaining its biological activity in PSMA-negative cells.

**Figure 4:**
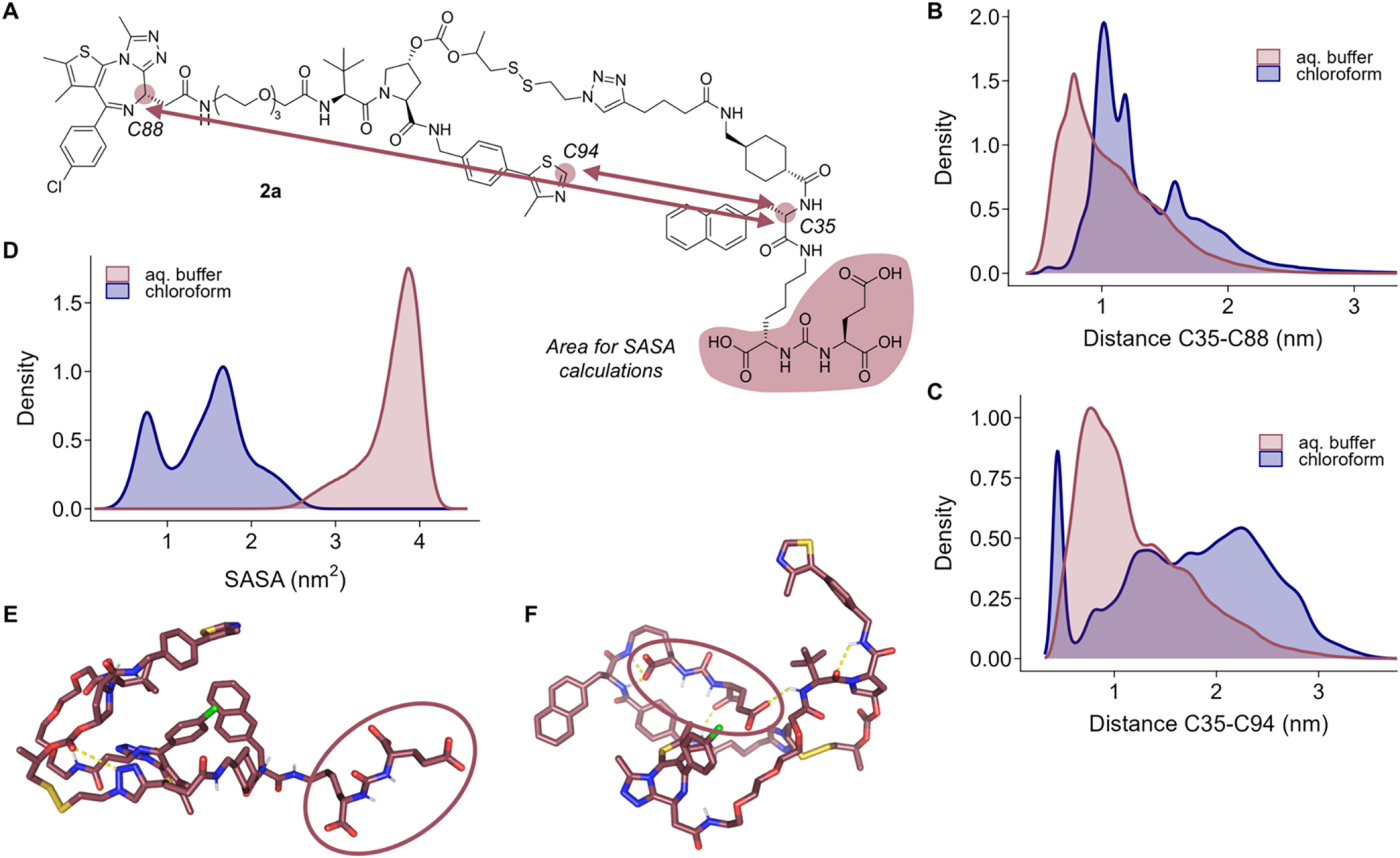
MD simulations of **2a** in aqueous buffer and chloroform. (A) Structure of **2a** with atoms used for distance calculations annotated and region used for SASA calculations highlighted in red. (B, C) Distances between C35 and C88 (B) or C94 (C) across all trajectories. (D) SASA of PSMA ligand tricarboxylate across all trajectories. (E, F) Representative structures of the most populated clusters in aqueous buffer (E, cluster includes 48% of structures, cut off = 3.2 Å) and chloroform (F, cluster includes 78% of structures, cut off 2.7 Å). PSMA ligand tricarboxylate circled, intramolecular polar contacts shown as dashed yellow lines.

### Linker Composition Affects Intramolecular Folding

To enforce PSMA-dependent prodrug activity, we introduced substantial structural modifications between the bioresponsive disulfide and the PSMA-targeting tricarboxylate while retaining the optimized secondary carbonate linkage. Specifically, the lipophilic naphthalene moiety was removed to minimize hydrophobic collapse near the PSMA-binding region and thereby enhance receptor engagement at the cell surface. In addition, extra charged functionalities were incorporated to reduce passive membrane permeability based on the hypothesis that these charges would not be effectively masked by the remainder of the molecule. More precisely, the new linker consisted of a *trans*-cyclohexane and hexanoic acid spacer, three d-glutamic acid residues, and a functionalizable lysine residue (**5a**, Figure 5A). The design of the highly negatively charged region was based on analogous linkers previously reported by the groups of Heston and Basilion, which, when coupled to a Glu-CO-Glu headgroup, reduced nonspecific background binding without affecting PSMA-affinity.^36,37^

**Figure 5:**
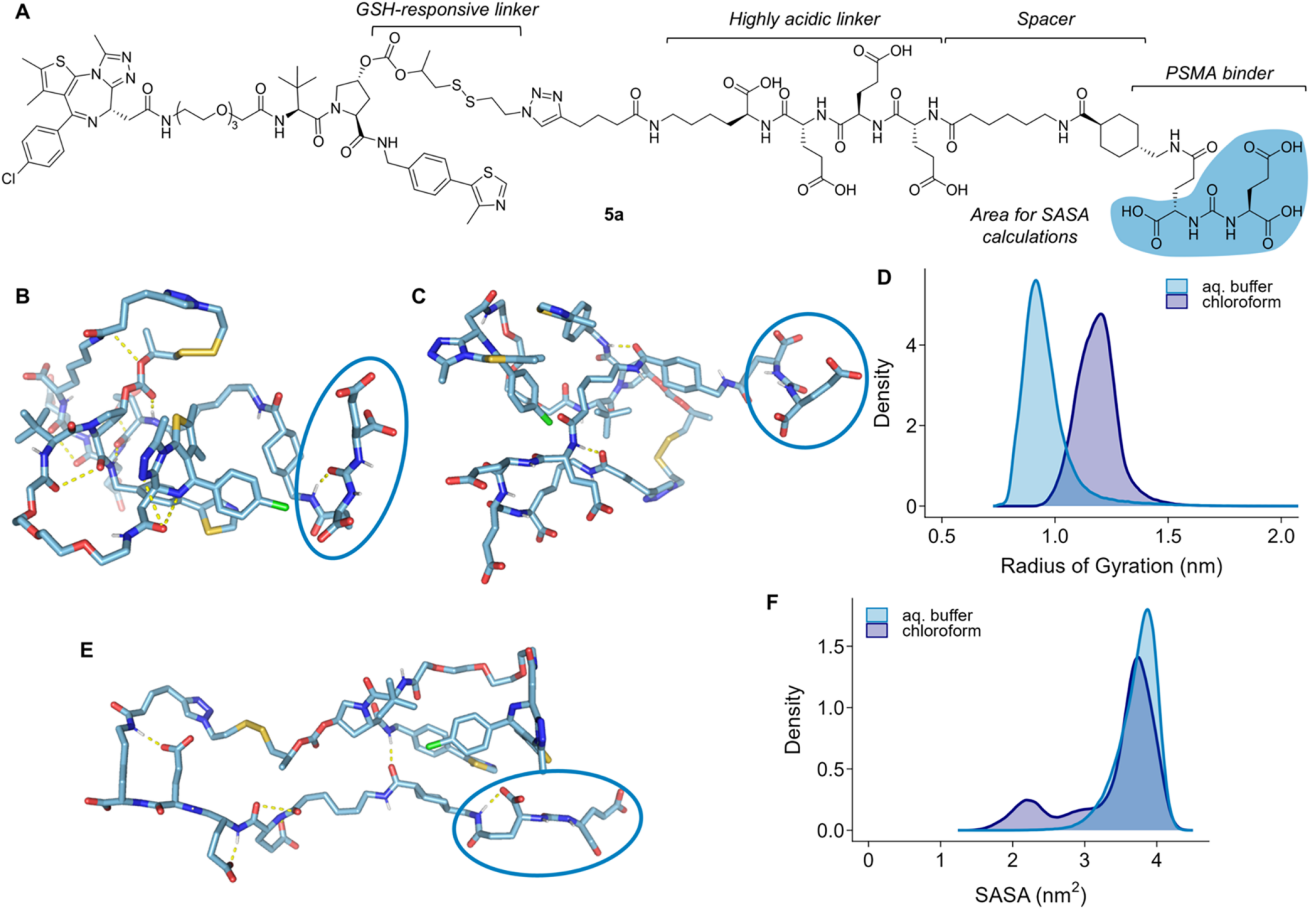
Structure of **5a** and MD simulations of its conformation in aqueous buffer and chloroform. (A) Structure of **5a** with region used for SASA calculations highlighted in blue. (B, C) Representative structures of the most populated clusters in aqueous buffer (B, cluster includes 13% of structures/C, cluster includes 10% of structures, both using a cut off = 3.6 Å). PSMA ligand tricarboxylate circled, intramolecular polar contacts shown as dashed yellow lines. (D) Radius of gyration across all trajectories. (E) Representative structure of the most populated cluster in chloroform (cluster includes 51% of structures, cut off = 2.7 Å). PSMA ligand tricarboxylate circled, intramolecular polar contacts shown as dashed yellow lines. (F) SASA of PSMA ligand tricarboxylate across all trajectories.

Multiple MD simulations of compound **5a** were carried out for a cumulative sampling of 4.4 and 9.2 μs in aqueous buffer and chloroform, respectively. The radius of gyration, alongside visual examination of the most populated clusters, showed that **5a** is still relatively compact in an aqueous buffer, with all charged groups on the surface and the PSMA ligand tricarboxylate again extending out into solution (Figure 5B–D). However, compared to **2a**, **5a** is much less compact in chloroform as there are insufficient hydrogen bond donors present, leaving many charged groups extended into the solvent (Figure 5D,E). This is clearly reflected in the SASA of the PSMA tricarboxylate, which is almost as solvent exposed in chloroform as in aqueous buffer (Figure 5F), suggesting that the passive permeability of **5a** should be reduced compared to **2a**.

### Synthesis of a PCa-Targeting MZ1 Prodrug with a Highly Acidic Linker

As the MD simulations with **5a** suggested a reduction in the potential for passive permeability, we synthesized disulfide **5a** and its analogous negative control alkylic compound **5b** for *in cellulo* testing (Scheme 2). The new acidic linker was prepared by solid phase peptide synthesis (SPPS) on HMPA resin, starting from the orthogonally protected Lys residue and following a standard Fmoc-strategy. The incorporation of the PSMA-binding moiety Glu-CO-Glu (Disconnection 1 in Scheme 2), as well as the linker functionalization with a hex-5-ynoic acid (Disconnection 2), was also carried out on the resin through the formation of isopeptide bonds. In parallel, **MZ1** was caged into azide-bearing secondary carbonate analogues (Disconnection 3) in a fashion similar to that discussed previously. The alkyne and azide handles on the respective intermediates were used in Cu­(I)-mediated azide–alkyne cycloaddition (Disconnection 4) to obtain the final conjugates (for experimental details, see SI Section 4).

**Scheme 2:**
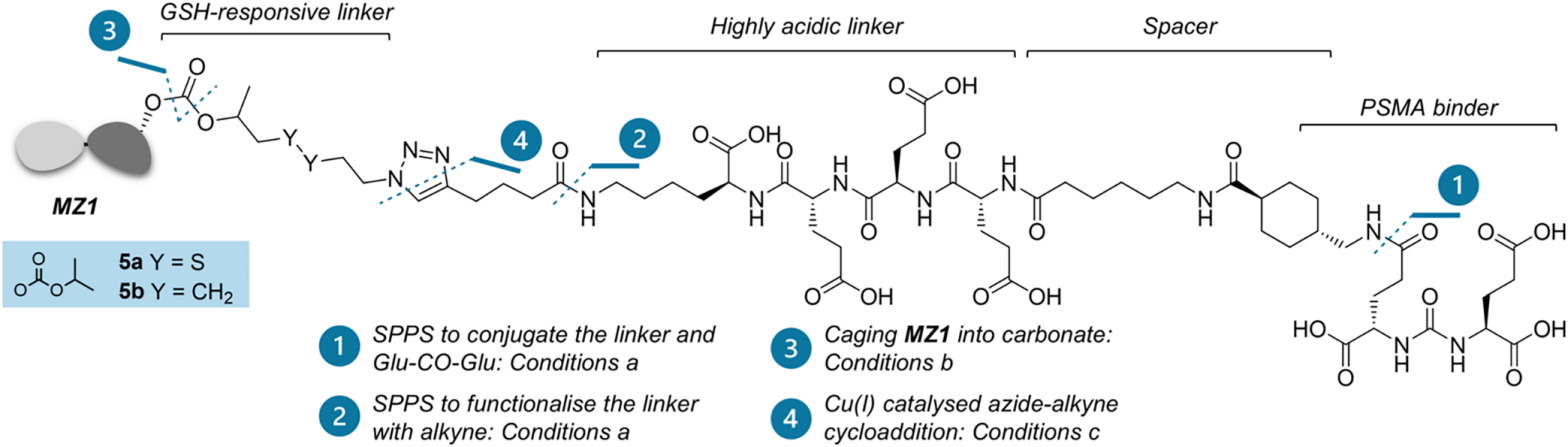
Synthesis of a Secondary Carbonate PSMA-Targeting **MZ1** Prodrug **5a** and Its Negative Control Analogue **5b** Bearing an Alkylic Linker^a^

a Reaction conditions for key transformations: (a) solid phase peptide synthesis (SPPS) through standard Fmoc-strategy (details in SI); (b) 4-Nitrophenyl carbonochloridate (1.1–2 equiv), DMAP (1.1–2 equiv), THF or DCM, 0 to 20 °C, 2–18 h; then formed carbonate linker (1–2.2 equiv), **MZ1** (1 equiv), DMAP (1.5 equiv), DCM, 20 °C, 18 h; (c) azide (1 equiv), Alkyne (1.5 equiv), CuSO_4_·5H_2_O (3 equiv), sodium ascorbate (3 equiv), TBTA (0.2 equiv), NMP, 20 °C, 16 h.

### Cellular Activity Studies of 5a and Additional MD Simulations

As with our original prodrug library, **5a** and **5b** were screened *in cellulo* in both PSMA-positive LNCaP cells and PSMA-negative PC3 cells, using antiproliferation assays and the direct measurement of BRD4 degradation by Western blotting. Promisingly, **5a** maintained a potency similar to that of **2a** and **MZ1** in LNCaP cells in both assays, demonstrating that **MZ1** is still efficiently released (Figures 6A–C and S24A in SI; for comparison with **2a**, see Figures 3A–D and S15A in SI). In contrast, **5b** was almost inactive, demonstrating a substantial reduction in the disulfide-independent release of **MZ1** compared with that of **2b**. This suggests an increased reliance of the activity of **5a** on intracellular GSH relative to **2a**, despite their structural differences being distant from both the disulfide where GSH attacks and the carbonate group, which had previously been identified as a key point of liability.

**Figure 6:**
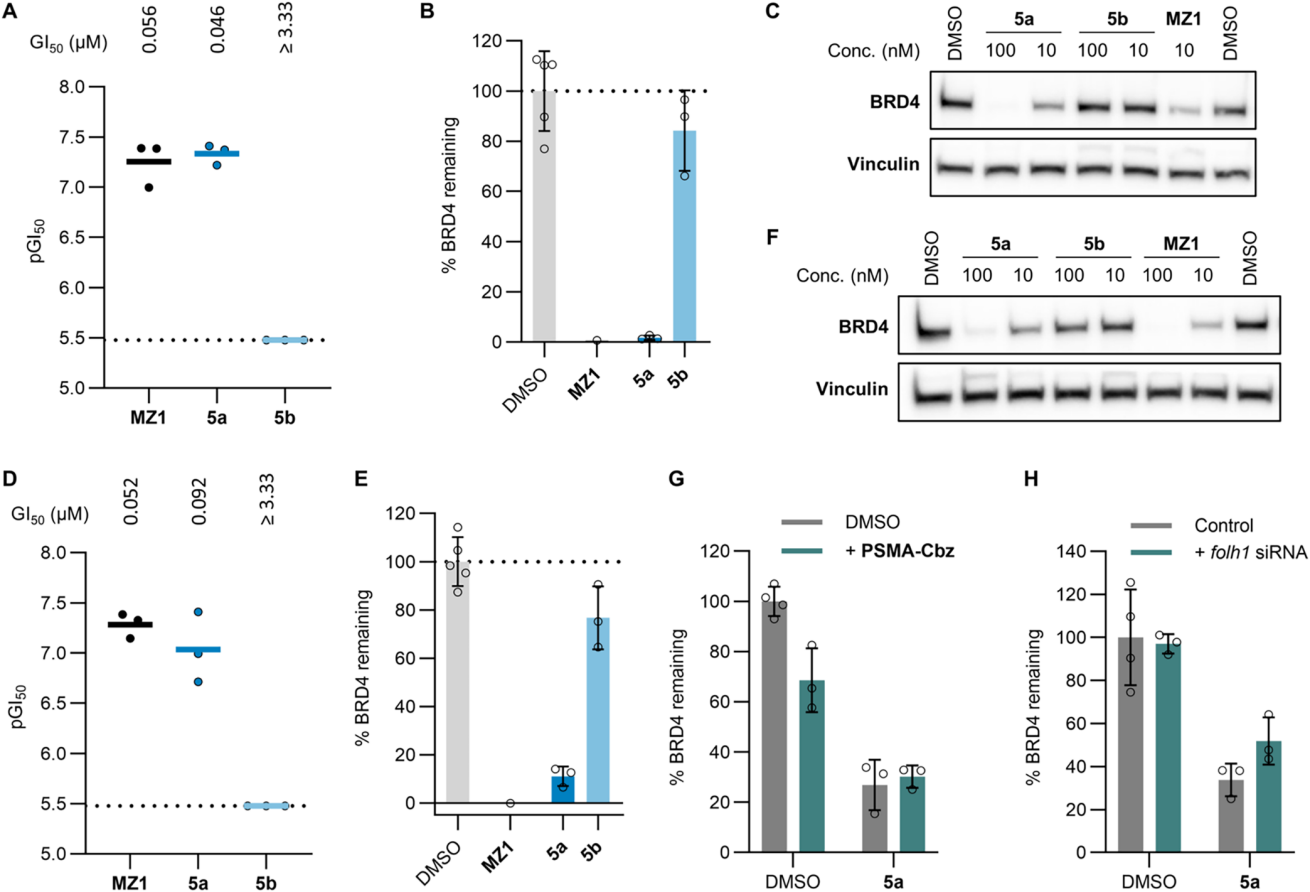
Assessment of the cellular activity of **5a**, **5b**, and **MZ1** in LNCaP and PC3 cells. (A) Antiproliferative effect in LNCaP cells following a three-day treatment. pGI_50_ = −log_10_(GI_50_), where GI_50_ corresponds to compound concentration required to reduce cell viability by 50% relative to DMSO-treated cells. One data point per experimental run, bar, and displayed GI_50_ show geometric mean of replicates; full dose response curves are shown in Figure S23 in SI. (B, C) BRD4 degradation in LNCaP cells, following a 4 h treatment. Degradation was determined by Western blotting, using Vinculin as a loading control, and percentage remaining was calculated by comparison to DMSO-treated cells run on the same gel. (B) Quantification of all replicates with 100 nM compound treatment and (C) a representative blot. See Figure S24B in SI for images of all blots used for quantification. (D) Antiproliferative effect in PC3 cells following a three-day treatment. Data processed and displayed as for (A), full dose response curves in Figure S25 in SI. (E, F) BRD4 degradation in PC3 cells, following a 4 h treatment. (E) Quantification of all replicates with 100 nM compound treatment, data processed and displayed as for B, images for all blots used for quantification in Figure S26B in SI. (F) A representative blot. (G) BRD4 degradation in LNCaP cells, following 30 min pre-treatment with 10 μM **PSMA-Cbz** and 2 h treatment with 10 nM **5a**. Data processed and displayed as for (B), images for all blots used for quantification in Figure S27 in SI. (H) BRD4 degradation in LNCaP cells, treated for 2 h with 10 nM **5a** 72 h after siRNA-mediated knockdown of *folh1* (or treatment with non-targeting siRNA = control). Data processed and displayed as for B, images for all blots used for quantification in Figure S28 in SI.

In PSMA-negative PC3 cells, **5b** remained largely inactive, while **5a** retained near-**MZ1** potency, in line with the profile observed for **2a** (Figures 6D–F and S26A in SI; for comparison with **2a** see Figures 3E–H and S15B in the SI). The PSMA-independent activity of **5a** in LNCaP cells was confirmed by blocking with excess ligand and siRNA-mediated knockdown of *folh1*, both of which did not have strong effects on BRD4 degradation by **5a** (Figure 6G,H). Together, these results suggest an efficient but PSMA-independent uptake of **5a** into cells, following which **MZ1** is effectively released in a GSH-dependent manner.

To explore the PSMA-independent activity of **5a**, we revisited the MD simulation performed in chloroform, which had indicated that **5a** would not be passively permeable. As **5a** is highly negatively charged in aqueous buffer, it may already associate with positive ions present in the cell culture medium before entering the cell membrane. Thus, additional MD simulations in chloroform were performed in the presence of seven Na^+^ ions to balance the seven negatively charged carboxylic acid groups in **5a** (cumulative sampling of 5.3 μs). The MD trajectories revealed that interactions with Na^+^ induce **5a** to adopt a much more compact structure (Figure 7A), resulting in the masking of the PSMA ligand tricarboxylate (Figure 7B,C). This involved the formation of a network of salt-bridges consisting of the tricarboxylate and the Na^+^ ions, which was very stable in the MD simulations at 300 K, such that the conformation of **5a** was mostly defined by the starting frame of the simulation. MD simulations at an elevated temperature (360 K, cumulative sampling of 8.7 μs) were far less reliant on the starting frame and exhibited very similar masking of the PSMA ligand tricarboxylate (Figure 7A,B). Hence, cotransport of positive ions may permit the passive permeability of **5a**, despite its highly negative charge, possibly explaining the PSMA-independent activity of this prodrug and highlighting the challenges of developing complex small-molecule constructs for targeted delivery.

**Figure 7:**
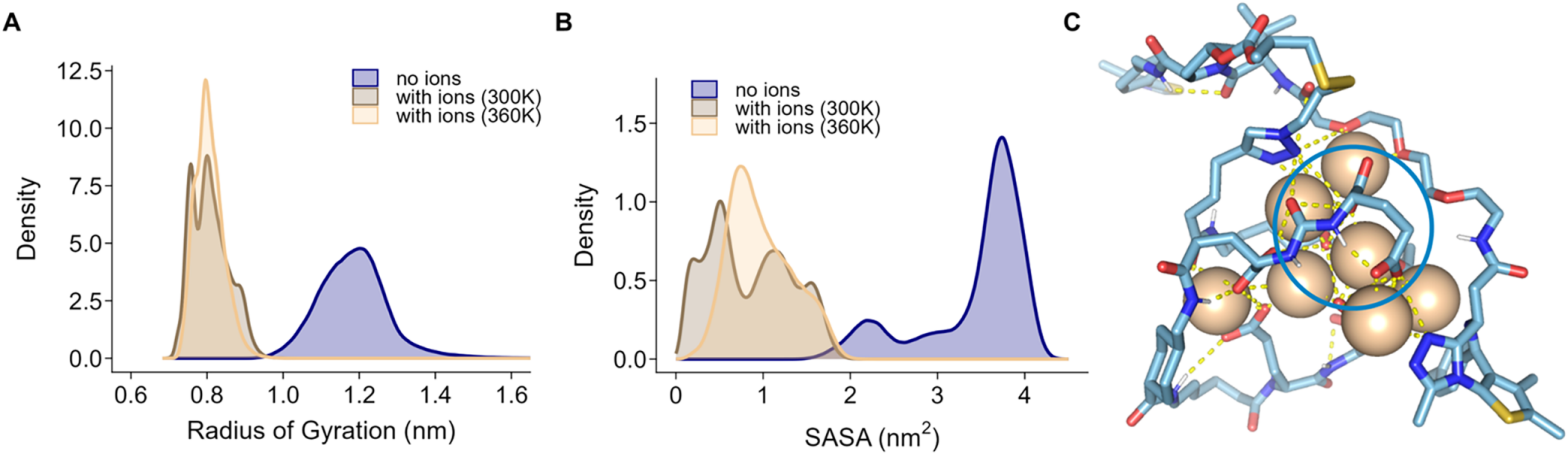
MD simulation of **5a** in chloroform in the presence of 7 Na^+^ ions. Radius of gyration (A) and SASA of PSMA ligand tricarboxylate (B, atoms used for calculation as in Figure 5A) across all trajectories. (C) Representative structure of the most populated cluster at 300 K with ions (includes 33% of structures, cut off = 2.7 Å). PSMA ligand tricarboxylate circled, intramolecular polar contacts shown as dashed yellow lines, and Na^+^ ions shown in beige.

## Conclusion

In this study, we present a series of PROTAC prodrugs featuring GSH-responsive disulfide linkers and a PSMA-delivery vector to selectively target prostate cancer. By sterically and electronically tuning the widely used but labile disulfide–primary carbonate linker, we significantly enhanced resistance to esterase- and serum-mediated hydrolysis through increased steric hindrance or thiocarbonate substitution. Biochemical and kinetic analyses revealed that these modifications selectively modulate the GSH accessibility of the disulfide and the downstream uncaging mechanism, providing tunable control over the kinetics of payload release. Notably, increased steric hindrance slowed disulfide reduction while simultaneously accelerating the downstream drug release via Thorpe–Ingold effects. Within our PSMA-targeting **MZ1** prodrugs, the disulfide–secondary carbonate linker offered an optimal balance between biological stability and GSH-mediated uncaging *ex cellulo*. Crucially, these trends translated directly to cellular activity. The disulfide–secondary carbonate prodrug **2a** demonstrated markedly enhanced antiproliferative effects and BRD4 degradation in PCa cells relative to its negative control, confirming the intended GSH-controlled release of **MZ1**. Unfortunately, PSMA-selective uptake could not be demonstrated. MD simulations revealed that intramolecular folding in nonpolar environments shields the tricarboxylate moiety of the PSMA ligand, promoting passive membrane permeability and explaining activity in PSMA-negative cells. Remarkably, introducing additional distal carboxylic acid groups into compound **5a** increased the dependence of activity on the disulfide. Simulations suggest that cotransported counterions may form salt-bridge networks that mask acidic groups, enabling their PSMA-independent uptake. Notably, this emergent passive permeability may compromise the intended targeting specificity and safety profile, highlighting the need for future designs that limit intramolecular folding—for example, through linker rigidification or shortening between the PSMA ligand and the disulfide, or by employing more rigid degraders.

Overall, our work demonstrates that subtle chemical modifications adjacent to the disulfide–carbonate linkage can markedly improve the balance between prodrug stability and payload release, establishing secondary carbonates as a promising scaffold for next-generation prodrug design. More broadly, our findings highlight how conformational dynamics and intramolecular interactions play decisive roles in the chameleonicity of large molecules. Collectively, this study provides design insights and mechanistic understanding to guide the rational creation of selective, tunable, and efficacious PROTAC prodrugs, emphasizing the value of holistic design strategies in PROTAC prodrug development beyond modular optimization.

## Supplementary Material



## Notes

Supplementary table (S1) and figures (S1-S28) including details of kinetic analysis, cell line validation, full dose response curves from antiproliferation assays and Western blot images used for quantification; experimental methods and procedures; and compound characterization including ^1^H and ^13^C NMR spectra (PDF)
