# Supplementary material for: Balancing Stability and Payload Release in Glutathione-Responsive PROTAC Prodrugs Targeting Prostate Cancer
Source: JACS Au. 2026 Jun 10;6(7):3816–27. doi: 10.1021/jacsau.6c00397 (PMC13417236; doi:10.1021/jacsau.6c00397)
Supplement: Supplementary file 1 [file au6c00397_si_001.pdf]

Balancing Stability and Payload Release in Glutathione-Responsive PROTAC Prodrugs Targeting Prostate Cancer

Eleen Laul,+[a] Katherine A. Gosselé,+[a] Christian M. Matter,[b] Wei-Hong Winston Liu,[a] Jorge A. González,[a] Jason P. Holland,[a] Amedeo Caflisch[b] and Cristina Nevado*[a]

[a] Department of Chemistry, University of Zurich, Switzerland

[b] Department of Biochemistry, University of Zurich, Switzerland

+These authors contributed equally

*cristina.nevado@chem.uzh.ch

Supporting Information

Table of Contents

1. Supplementary Tables and Figures 2
2. Materials and Methods 22
3. Mechanistic Studies of the GSH-Mediated Release Mechanism 31
4. Synthetic Experimental Procedures, Schemes and Compound Characterisation 36
5. ^{1}H and ^{13}C NMR Spectra 66
6. References 103

1. Supplementary Tables and Figures

For **Table S1** see section 3. Mechanistic studies of the GSH-mediated release mechanism.

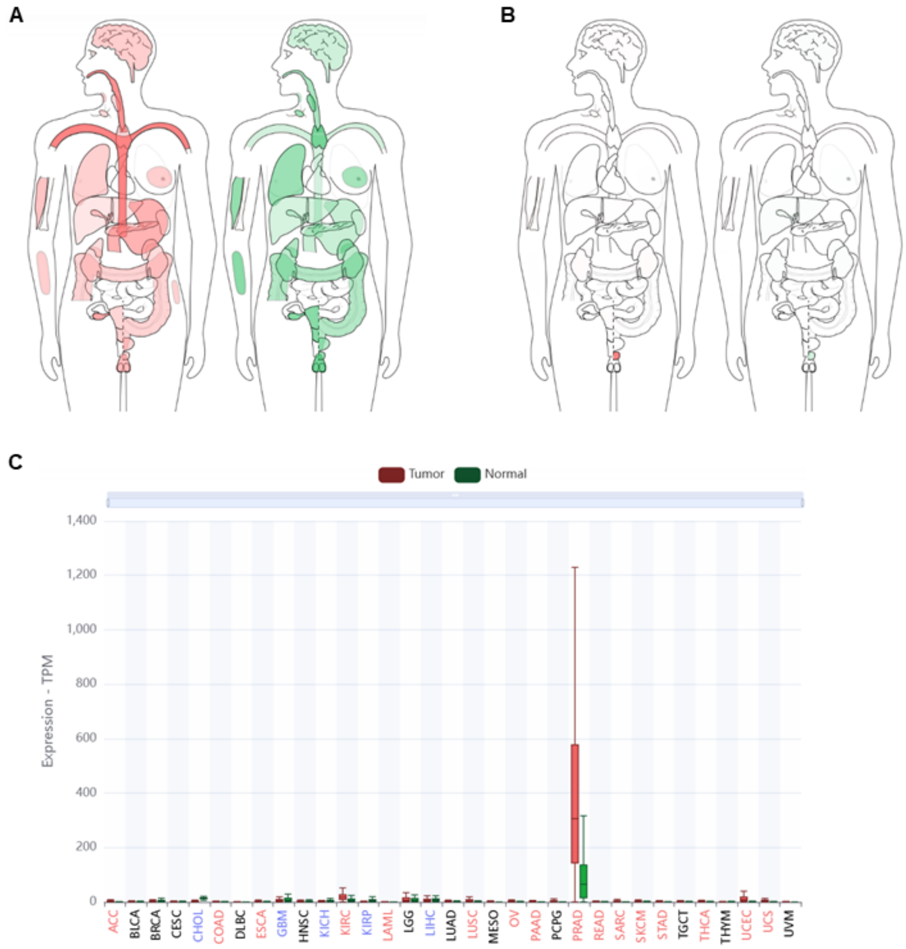

Figure S1. Median expression of *brd4* (A) or *folh1* [gene encoding PSMA] (B) in tumour (red) and normal (green) tissue samples. Darker shading indicates higher expression. C) Expression profile of *folh1* across all tumour samples and normal tissues. PRAD = prostate adenocarcinoma. All plots taken from the GEPIA 3 webpage, accessed Feb. 2026.[1]

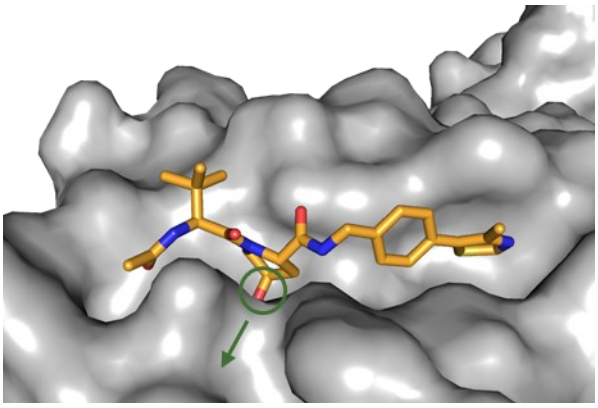

Figure S2. Crystal structure of **VH032** in complex with VHL (PDB: 4W9H). Position and vector of prodrug appendage indicated with green circle and arrow.

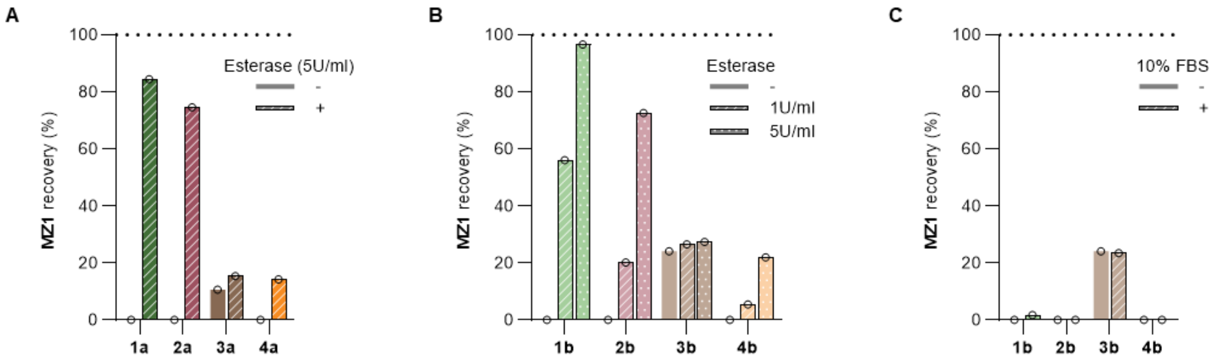

Figure S3. A,B) Recovery of **MZ1** upon the incubation of the disulfide prodrugs **1a**-**4a** and negative control compounds **1b**-**4b** (20 µM) in the presence of porcine liver esterase (1 or 5 U/ml) after 4 h (pH 7.4, 37 °C), samples analysed by UHPLC-MS after protein precipitation. C) Recovery of **MZ1** upon the incubation of **1b**-**4b** (10 µM) in PBS in the presence or absence of 10% FBS after 4 h (pH 7.4, 37 °C), samples analysed by UHPLC-MS after protein precipitation.

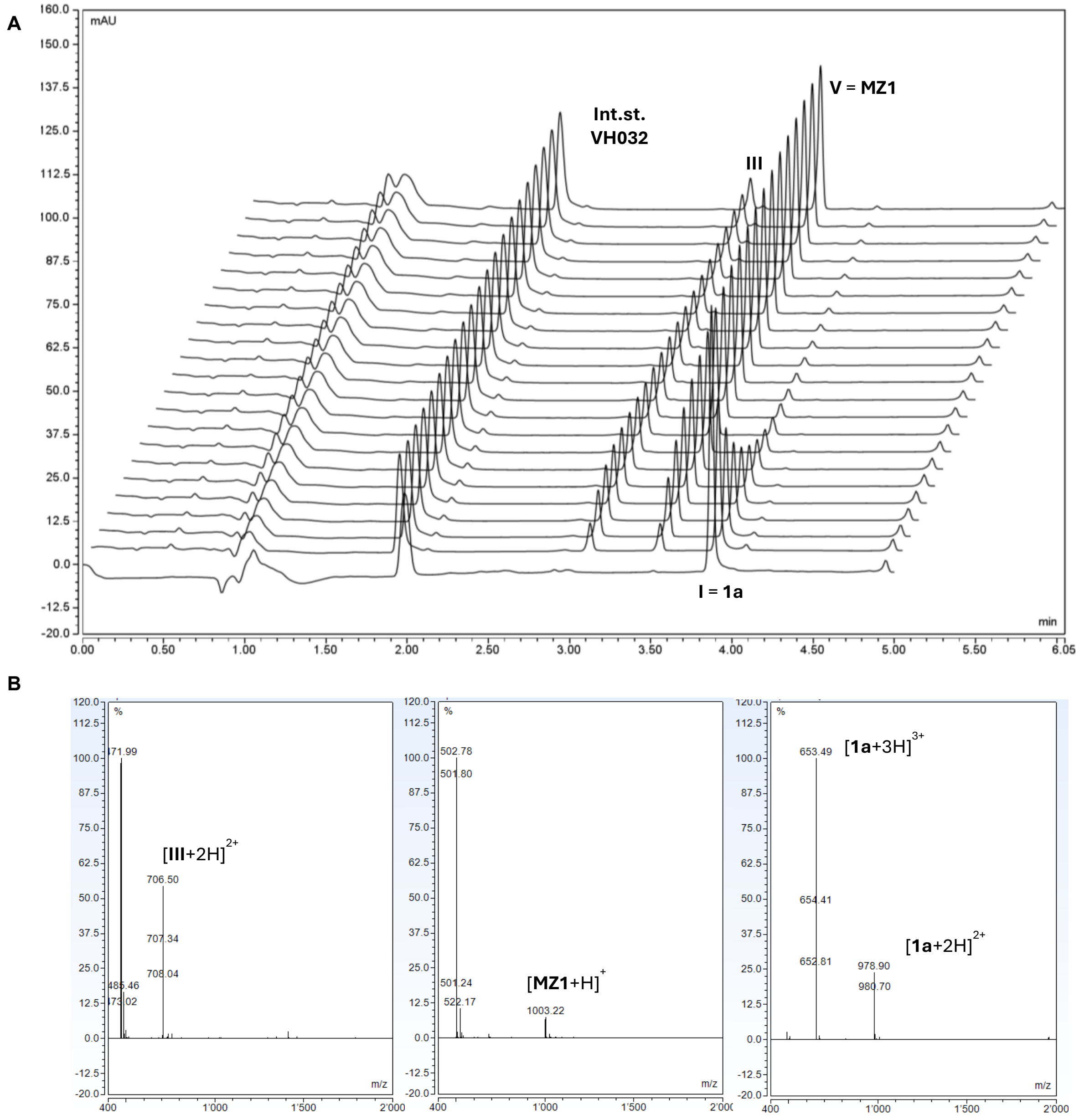

Figure S4. GSH-mediated disulfide prodrug cleavage of compound **1a**. A) UHPLC-MS traces for compound **1a** (10 µM) in the presence of GSH (2 mM) (pH 7.4, 37 °C) over 4 h time period with peaks assigned according to the MS profiles. B) MS spectra used for peak assignment.

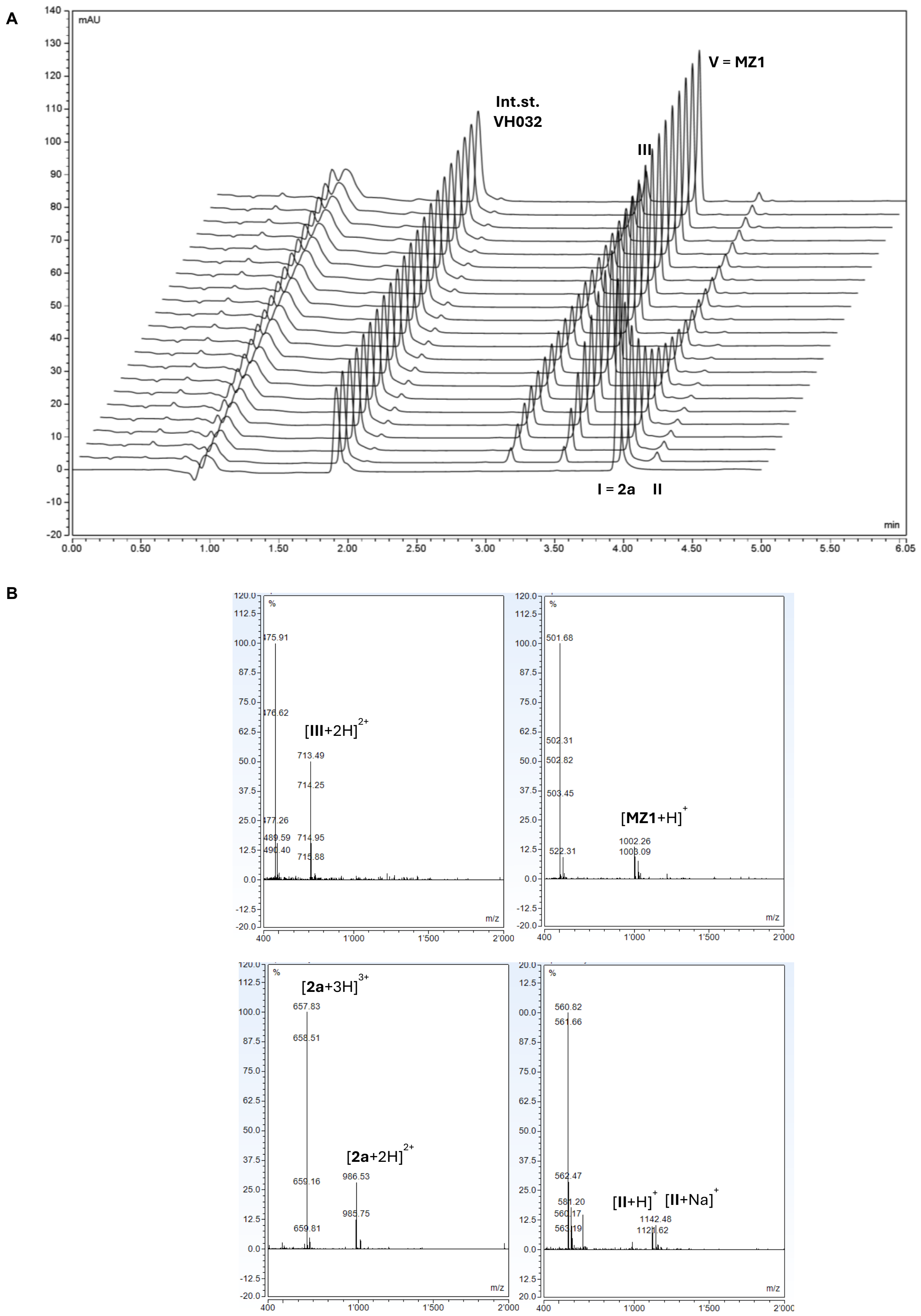

Figure S5. GSH-mediated disulfide prodrug cleavage of compound **2a**. A) UHPLC-MS traces for compound **2a** (10 µM) in the presence of GSH (2 mM) (pH 7.4, 37 °C) over 4 h time period with peaks assigned according to the MS profiles. B) MS spectra used for peak assignment.

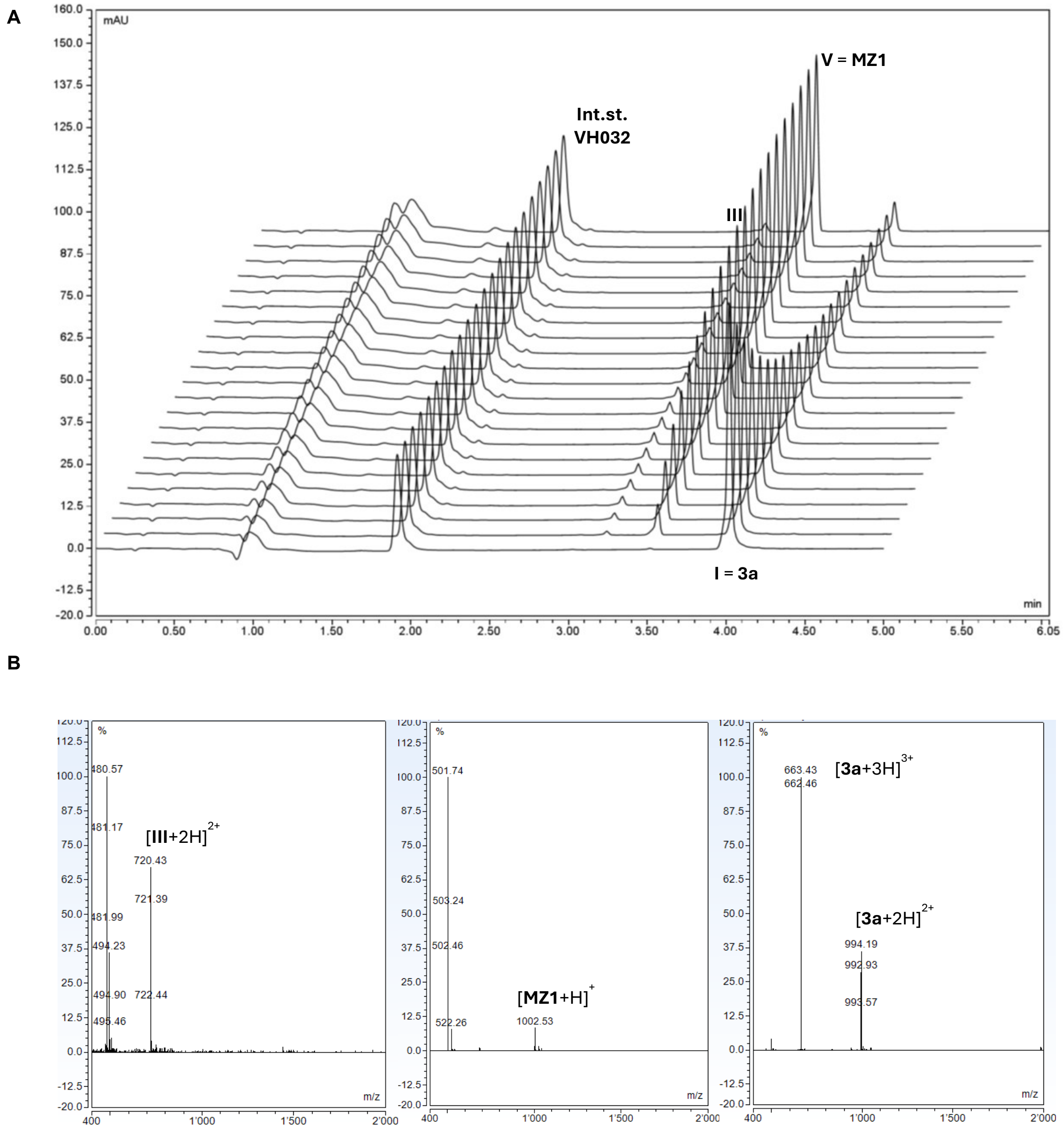

Figure S6. GSH-mediated disulfide prodrug cleavage of compound **3a**. A) UHPLC-MS traces for compound **3a** (10 µM) in the presence of GSH (2 mM) (pH 7.4, 37 °C) over 4 h time period with peaks assigned according to the MS profiles. B) MS spectra used for peak assignment.

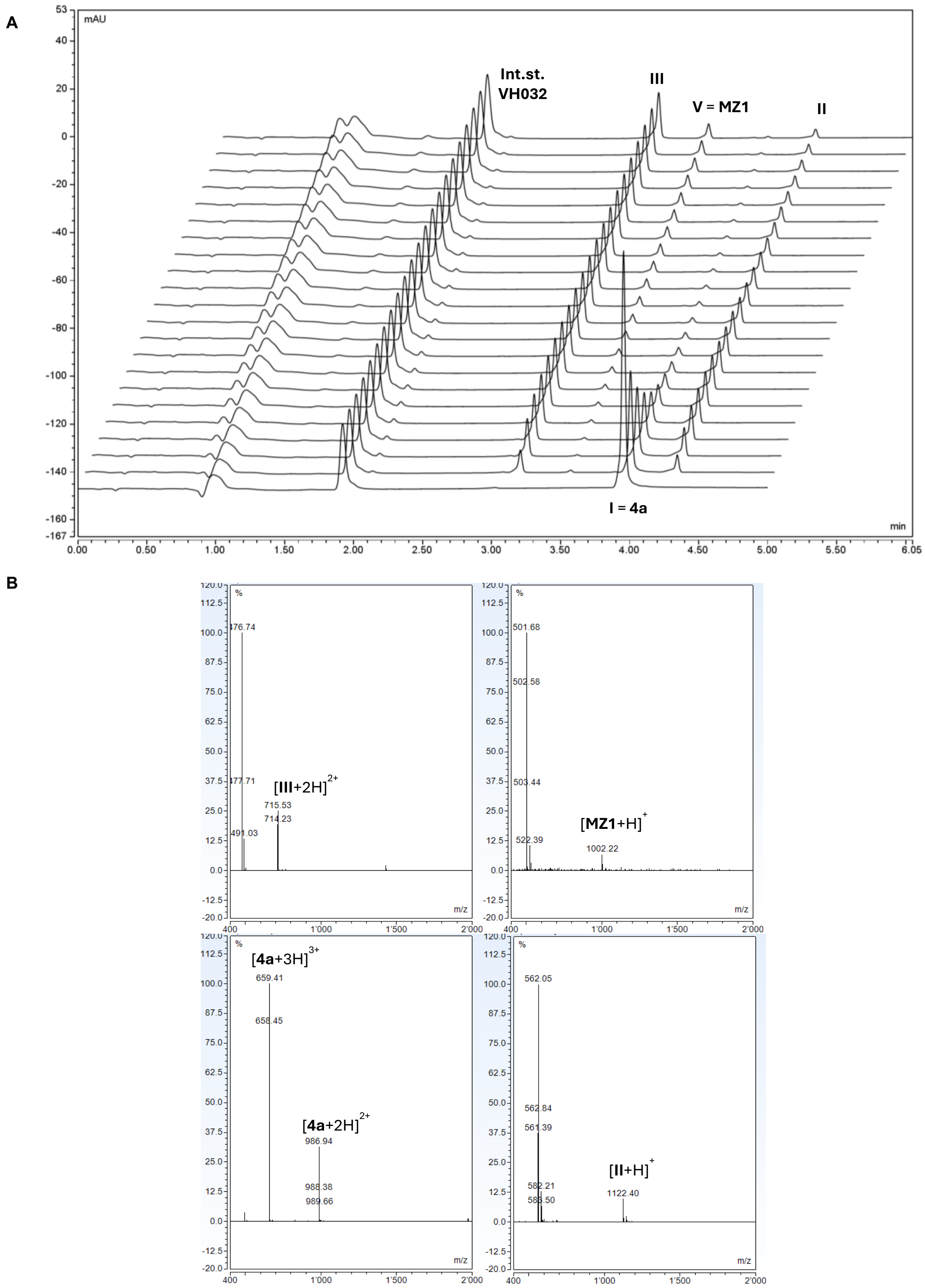

Figure S7.GSH-mediated disulfide prodrug cleavage of compound **4a**. A) UHPLC-MS traces for compound **4a** (10 µM) in the presence of GSH (2 mM) (pH 7.4, 37 °C) over 4 h time period with peaks assigned according to the MS profiles. B) MS spectra used for peak assignment.

For **Figure S8** and **Figure S9** see section 3. Mechanistic studies of the GSH-mediated release mechanism.

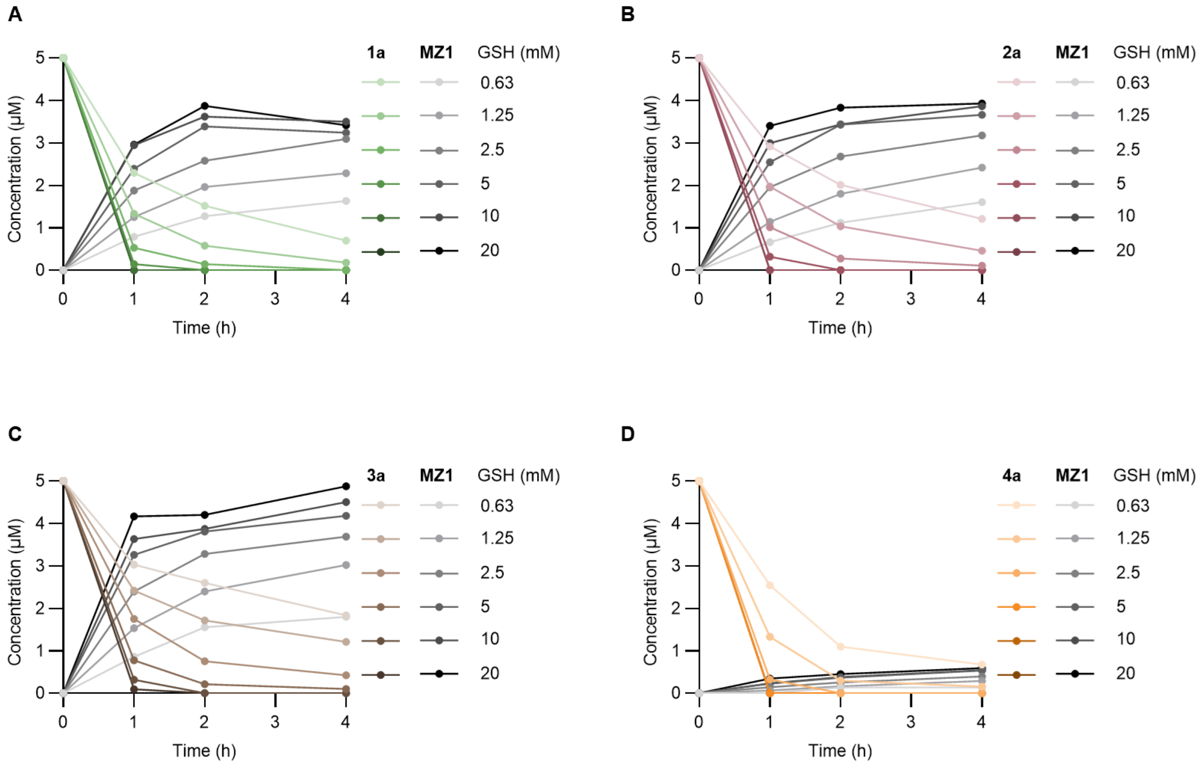

Figure S10. GSH-mediated disulfide-prodrug cleavage dependence on GSH concentration. A-D) Experimental data of GSH-mediated **MZ1** release from compounds **1a**-**4a** (10 µM) in the presence of varying concentrations of GSH (0.63-20 mM) for 1, 2 and 4 h (pH 7.4, 37 °C). After incubation, samples were diluted 2-fold by the addition of MeCN and analysed by UHPLC-MS, with an internal standard (20 µM). The intermediates present in the samples have been omitted from the figures for clarity.

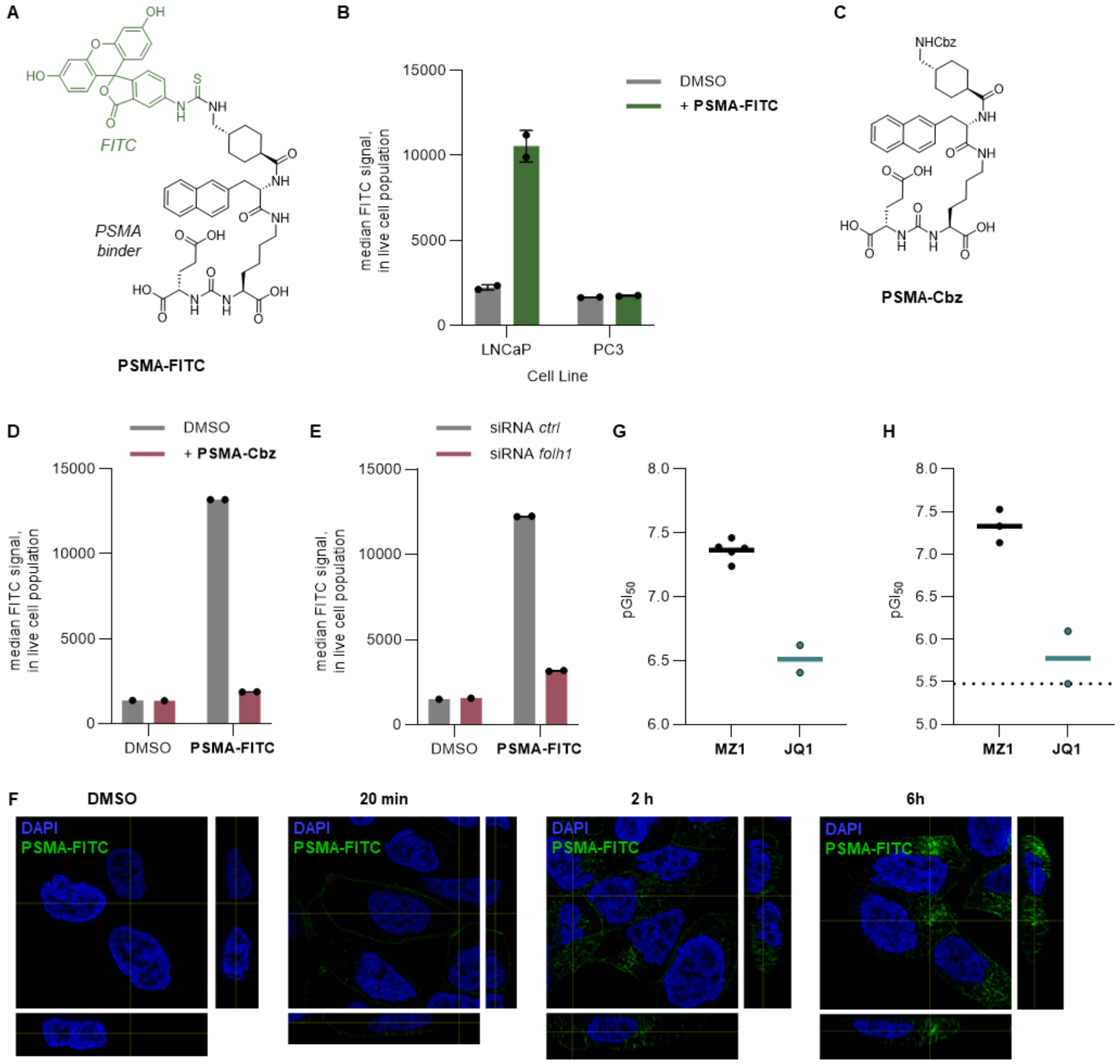

Figure S11. Validation of cell lines for prodrug testing. A) Structure of **PSMA-FITC** ligand. B) Median FITC signal in live cell population as measured by flow cytometry, following a 2 h treatment of cells with 100 nM **PSMA-FITC**. C) Structure of **PSMA-Cbz**. D) Median FITC signal in live cell population, measured by flow cytometry, of LNCaP cells pre-treated for 30 min with DMSO or 1 µM **PSMA-Cbz** then for 2 h with DMSO or 10 nM **PSMA-FITC**. E) Median FITC signal in live cell population, measured by flow cytometry, of LNCaP cells with a 72 h siRNA-mediated knockdown of *folh1* (or non-targeting siRNA = *ctrl*) treated for 2 h with DMSO or 10 nM **PSMA-FITC**. F) Confocal microscopy images of LNCaP cells following treatment with 100 nM **PSMA-FITC**. FITC signal shown in green, nucleus stained with DAPI and shown in blue. Shown is a single plane from the z-stack alongside orthogonal projections (relative positions marked with yellow lines). G-H) Anti-proliferative effect of control compounds in LNCaP (G) and PC3 (H) cells following a three-day treatment. $pGI_{50} = -\log_{10}(GI_{50})$, where GI_{50} corresponds to the compound concentration required to reduce cell viability by 50% relative to DMSO-treated cells; one data point per experimental run, bar shows geometric mean. Dotted line indicates the pGI_{50} calculated from the maximum tested compound concentration, which was the value assigned for plotting where a 50% decrease in viability was not reached.

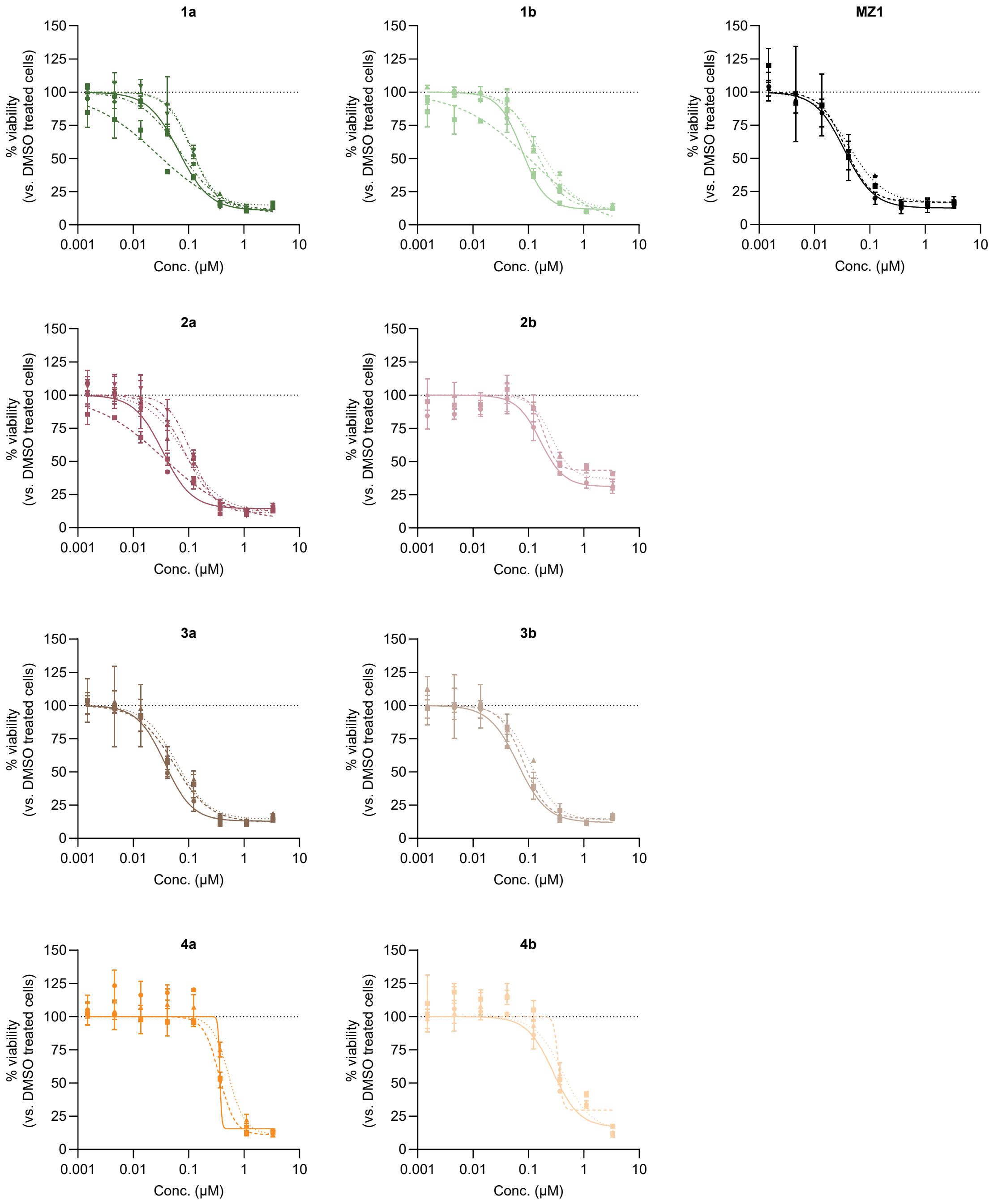

Figure S12. Antiproliferation dose-response curves used to construct Figure 3A: three-day treatment of PSMA-positive LNCaP cells. Each curve represents an independent biological replicate, and the error bars show the standard deviation for replicates within the same experiment. Viability was determined using resazurin and normalized to DMSO-treated cells on the same plate.

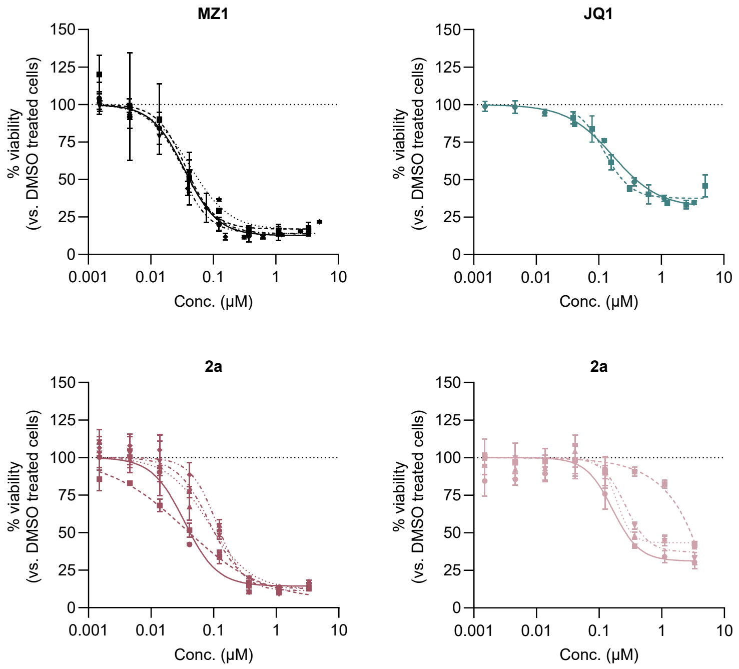

Figure S13. Antiproliferation dose-response curves used to construct Figure 3B: three-day treatment of PSMA-positive LNCaP cells. Each curve represents an independent biological replicate, and the error bars show the standard deviation for replicates within the same experiment. Viability was determined using resazurin and normalized to DMSO-treated cells on the same plate.

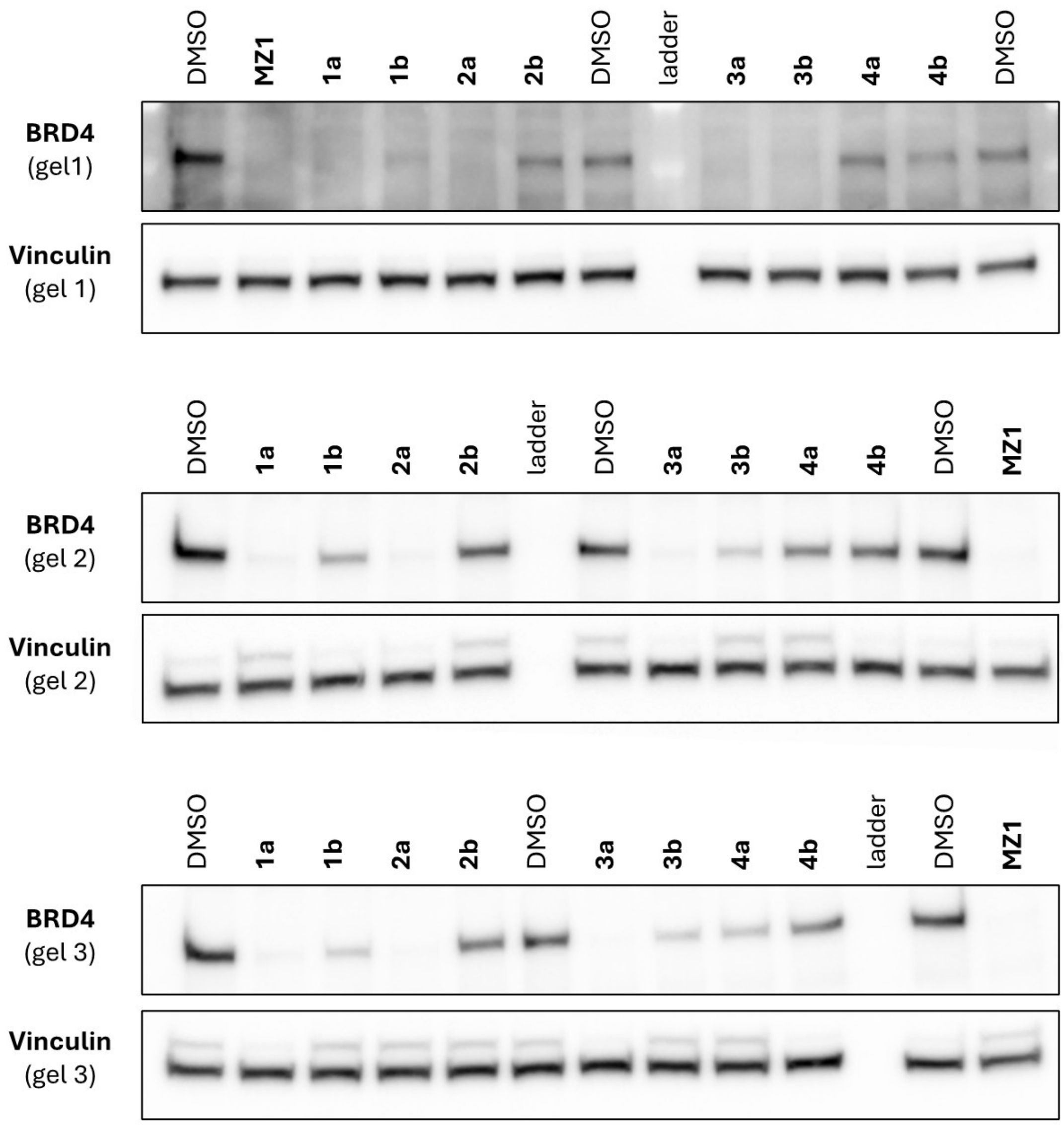

Figure S14. Images of all Western blots used for quantification in Figure 3C: single-dose measurements of BRD4 levels in LNCaP cells after 4 h treatment with 0.1 μM compounds. Solid boxes encompass bands from same membrane section.

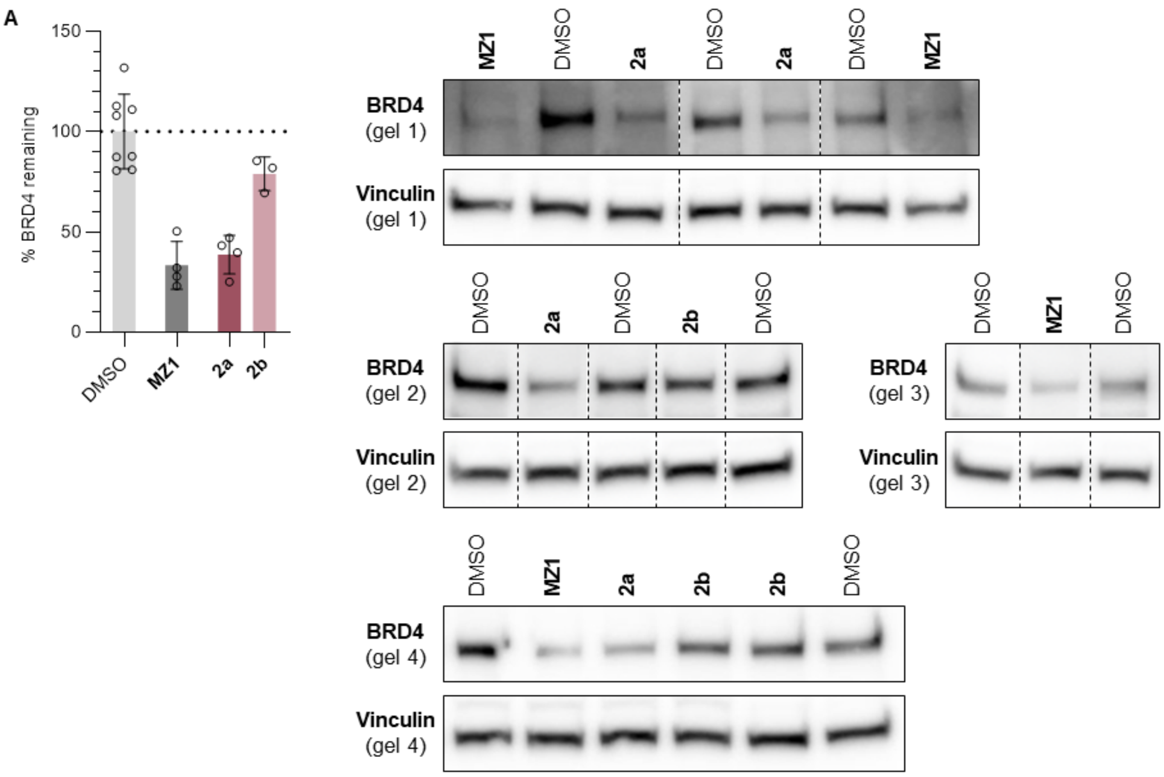

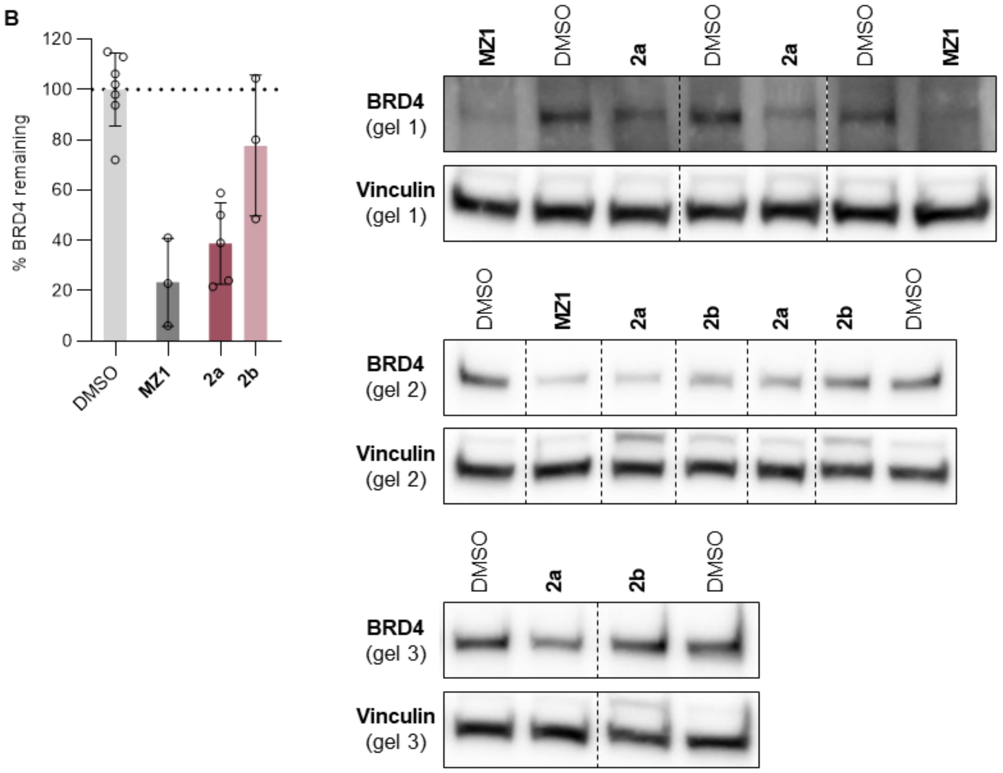

Figure S15. BRD4 degradation in LNCaP cells (A) and PC3 cells (B) following a 4 h treatment with 10 nM compounds. Degradation was determined by Western blotting, using vinculin as a loading control, and percentage remaining was calculated by comparison to DMSO-treated cells run on the same gel. Images of all blots used for quantification shown. Solid boxes encompass bands from same membrane section, dashed lines indicate where blot has been cropped to remove irrelevant lanes.

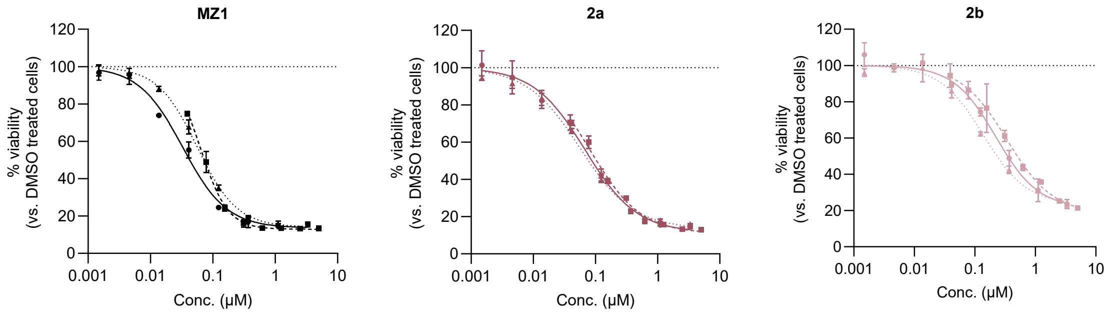

Figure S16. Antiproliferation dose-response curves used to construct Figure 3E: three-day treatment of PSMA-negative PC3 cells. Each curve represents an independent biological replicate, and the error bars show the standard deviation for replicates within the same experiment. Viability was determined using resazurin and normalized to DMSO-treated cells on the same plate.

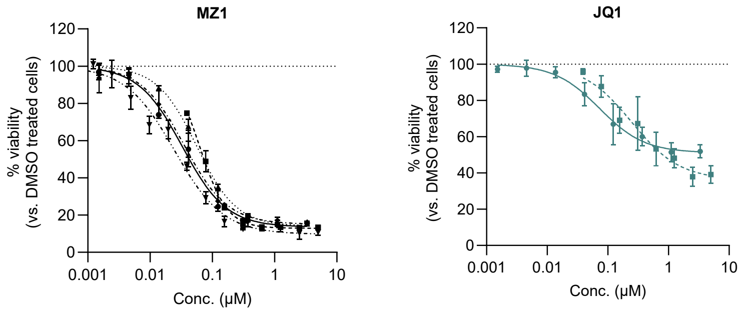

Figure S17. Antiproliferation dose-response curves used to construct Figure 3F: three-day treatment of PSMA-negative PC3 cells. Each curve represents an independent biological replicate, and the error bars show the standard deviation for replicates within the same experiment. Viability was determined using resazurin and normalized to DMSO-treated cells on the same plate. Data used for **2a** and **2b** shown in Figure S15.

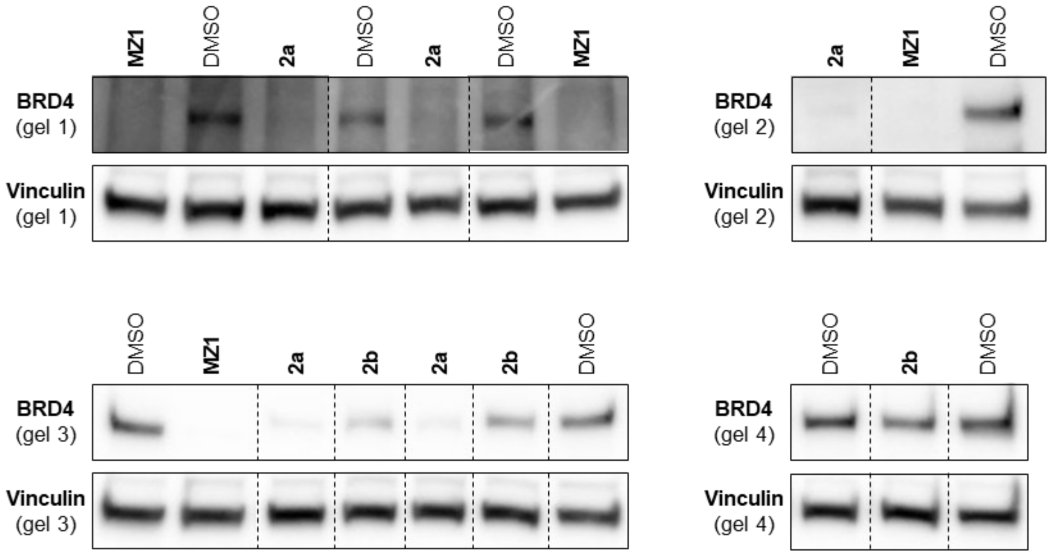

Figure S18. Images of all Western blots used for quantification in Figure 3G: single-dose measurements of BRD4 levels in PC3 cells after 4 h treatment with 0.1 μM compounds. Solid boxes encompass bands from same membrane section, dashed lines indicate where blot has been cropped to remove irrelevant lanes.

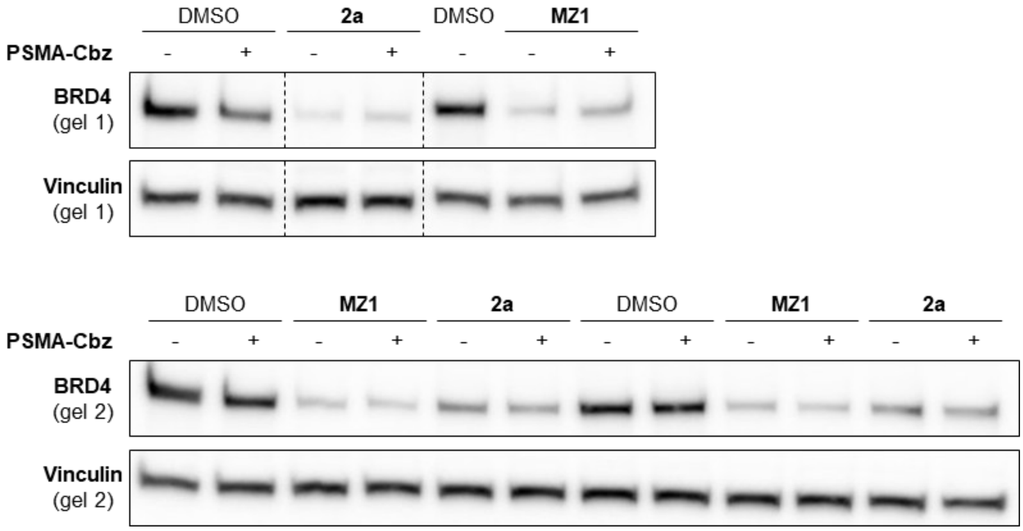

Figure S19. Images of all Western blots used for quantification in Figure 3I: BRD4 levels in LNCaP cells following 30 min pre-treatment with 10 μM **PSMA-Cbz** and 2 h treatment with 10 nM **MZ1** or **2a**. Solid boxes encompass bands from same membrane section, dashed lines indicate where blot has been cropped to remove irrelevant lanes.

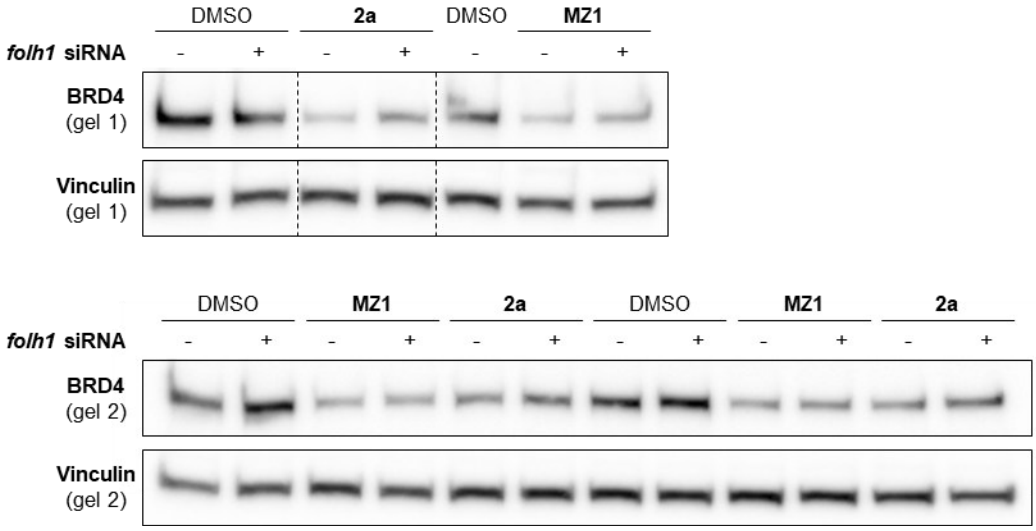

Figure S20. Images of all Western blots used for quantification in Figure 3J: BRD4 levels in LNCaP cells treated for 2 h with 10 nM **MZ1**/**2a**, 72 h after siRNA-mediated knockdown of *folh1* (or non-targeting siRNA = -). Solid boxes encompass bands from same membrane section, dashed lines indicate where blot has been cropped to remove irrelevant lanes.

Figure S21. Experimental observations consistent with intramolecular folding.

1. Although pre-cleaved prodrugs blocked **PSMA-FITC** binding to LNCaP cells as efficiently as **PSMA-Cbz**, improvements in prodrug stability reduced their blocking ability, indicating a reduction in the accessibility of the PSMA ligand in intact prodrugs (Figure S21A).
2. An **MZ1**-N_3 synthetic precursor of **3b** showed significantly blunted BRD4 degradation relative to **3b**, hinting that the presence of the PSMA delivery ligand labilises the carbonate moiety (Figure S21B).
3. The non-specific hydrolysis of the tertiary carbonate alkylic control prodrug (**3b**) was pH dependent, suggesting that the protonation state of the carboxylic acid groups affects intramolecular interactions and the aforementioned lability (Figure S21C).

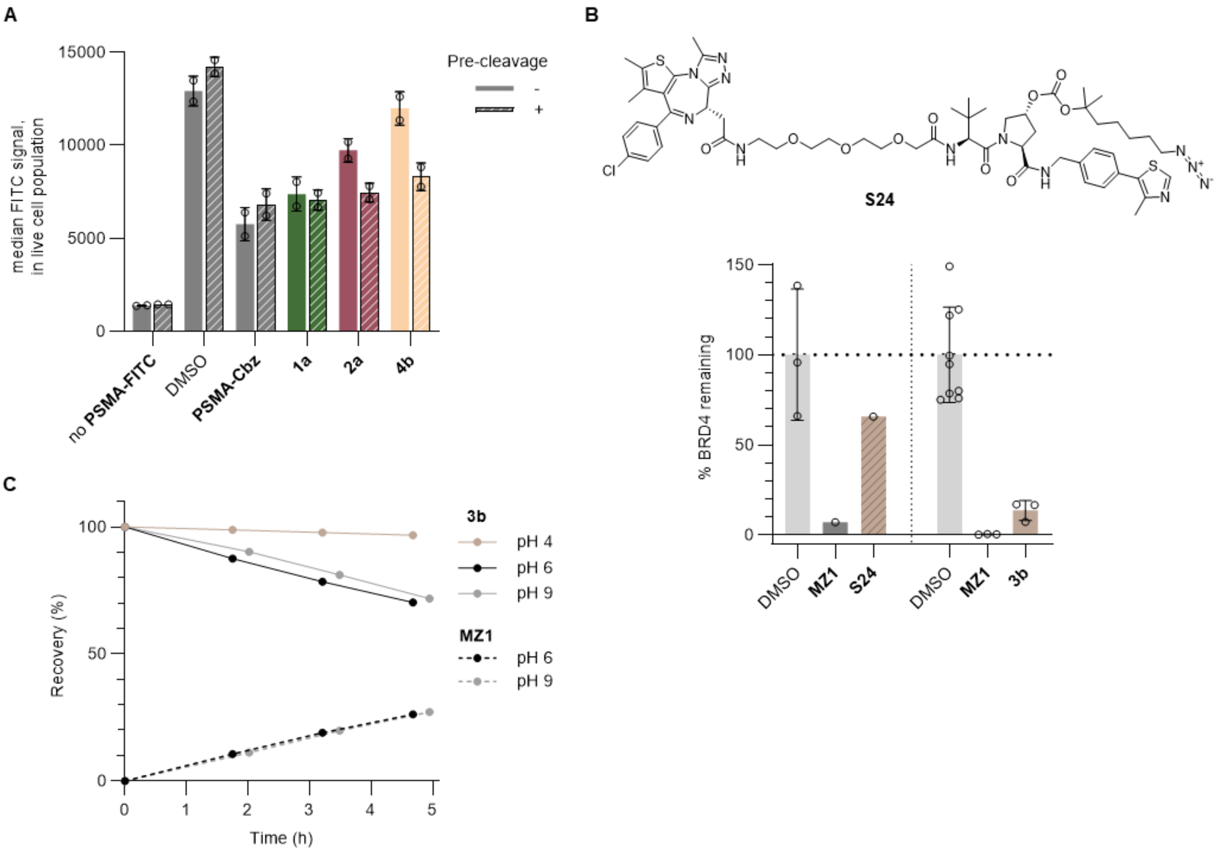

Figure S21. Experimental observations consistent with intramolecular folding of prodrugs in aqueous buffer. A) Median FITC signal in live cell population as measured by flow cytometry after pre-treatment of LNCaP cells for 30 min with 33 nM of either **PSMA-Cbz/1a/2a/4b** or the same compounds after their pre-cleavage by 16 h treatment with 5 U/ml esterase (whole reaction mixture added to LNCaP cells), followed by 2 h of 10 nM **PSMA-FITC**. B) Structure of the **MZ1**-N_3 synthetic precursor of **3b** (**S24**), and quantification of BRD4 levels in LNCaP cells following a 4 h treatment with 100 nM compounds [right side of figure, separated by dotted line, taken directly from Figure 3C for comparison]. C) Recovery of **3b** or **MZ1** upon the incubation of **3b** (10 µM) at varying pH (PBS, 37 °C), aliquots taken over 5 h and analysed by UHPLC-MS. Compound **3b** exhibited similar degradation in PBS at pH 6 and 9, but remained intact at pH 4. In comparison, **1b**, **2b**, and **4b** remained stable across pH 4-9 (data not shown).

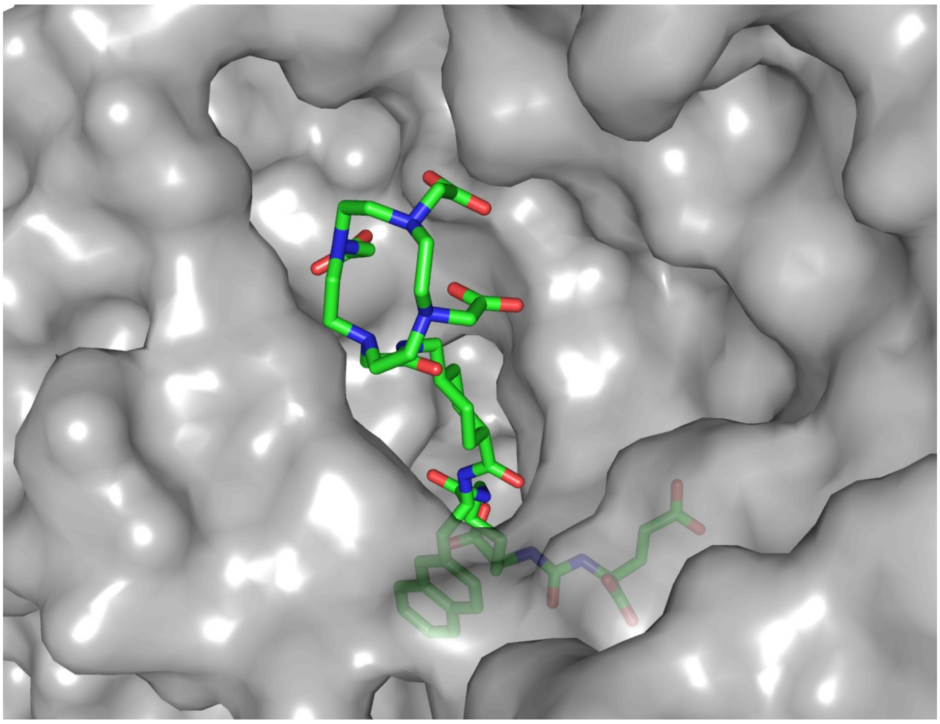

Figure S22. Crystal structure of PSMA-617 in complex with PSMA protein (PDB: 8BOW).[2] See also Novakova *et al.*[3] and Kopka *et al.*[4] for further information on tunnel-like access to ligand binding site.

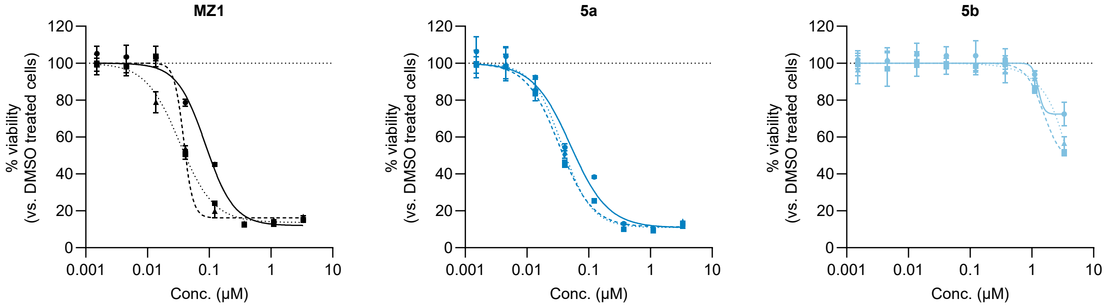

Figure S23. Antiproliferation dose-response curves used to construct Figure 6A: three-day treatment of PSMA-positive LNCaP cells. Each curve represents an independent biological replicate, and the error bars show the standard deviation for replicates within the same experiment. Viability was determined using resazurin and normalized to DMSO-treated cells on the same plate.

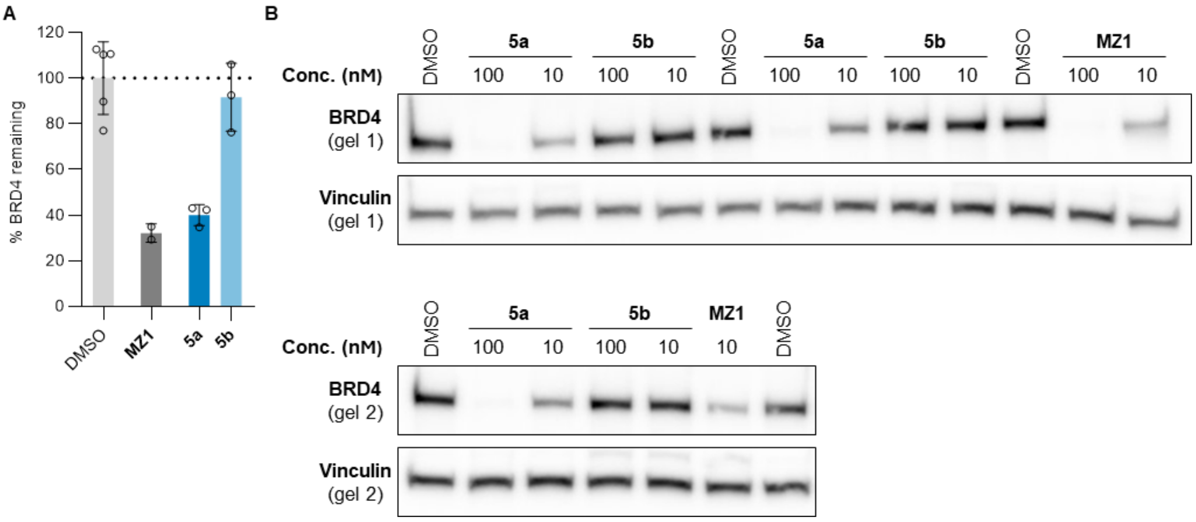

Figure S24. A) BRD4 degradation in LNCaP cells following a 4 h treatment with 10 nM compounds. Degradation was determined by Western blotting, using vinculin as a loading control, and percentage remaining was calculated by comparison to DMSO-treated cells run on the same gel. B) Images of all Western blots used for quantification in Figure 6B (100nM) and Figure S24A (10nM). Solid boxes encompass bands from same membrane section.

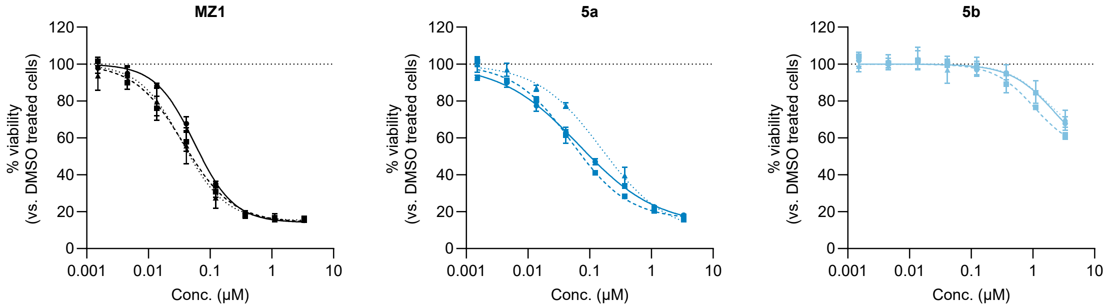

Figure S25. Antiproliferation dose-response curves used to construct Figure 6D: three-day treatment of PSMA-negative PC3 cells. Each curve represents an independent biological replicate, and the error bars show the standard deviation for replicates within the same experiment. Viability was determined using resazurin and normalized to DMSO-treated cells on the same plate.

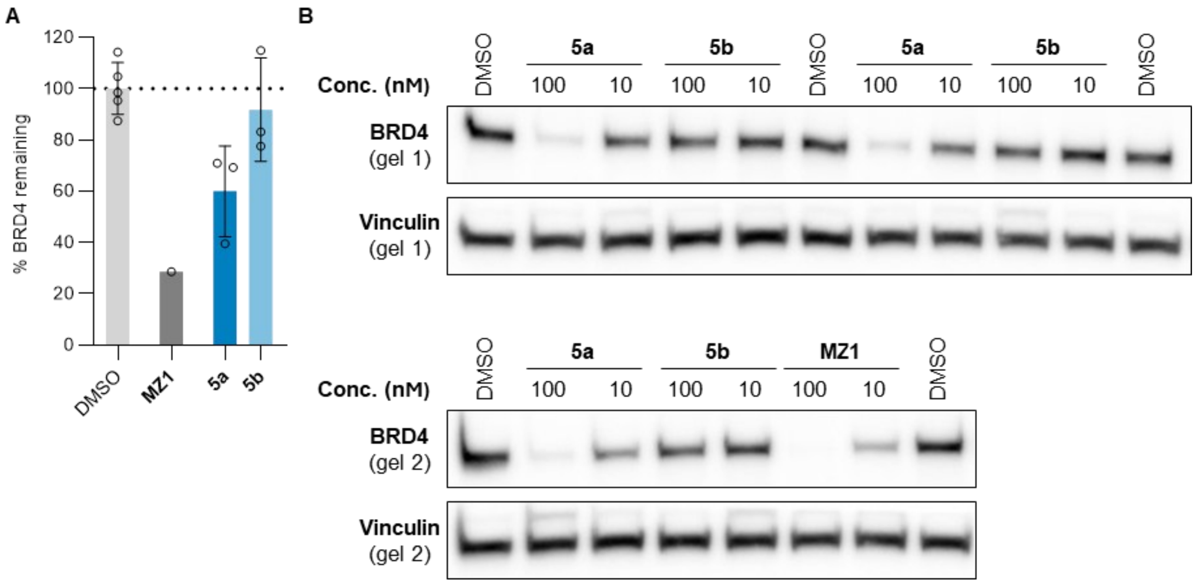

Figure S26. A) BRD4 degradation in PC3 cells following a 4 h treatment with 10 nM compounds. Degradation was determined by Western blotting, using vinculin as a loading control, and percentage remaining was calculated by comparison to DMSO-treated cells run on the same gel. B) Images of all Western blots used for quantification in Figure 6E (100nM) and Figure S26A (10nM). Solid boxes encompass bands from same membrane section.

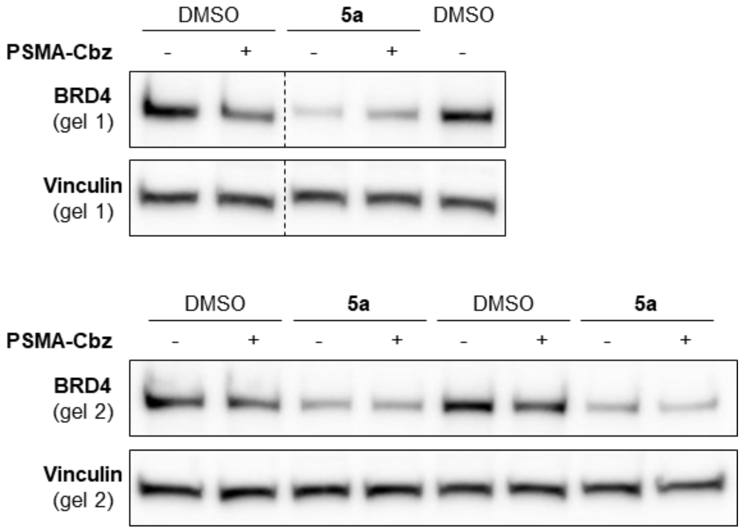

Figure S27. Images of all Western blots used for quantification in Figure 6G: BRD4 levels in LNCaP cells following 30 min pre-treatment with 10 μM **PSMA-Cbz** and 2 h treatment with 10 nM **5a**. Solid boxes encompass bands from same membrane section, dashed lines indicate where blot has been cropped to remove irrelevant lanes.

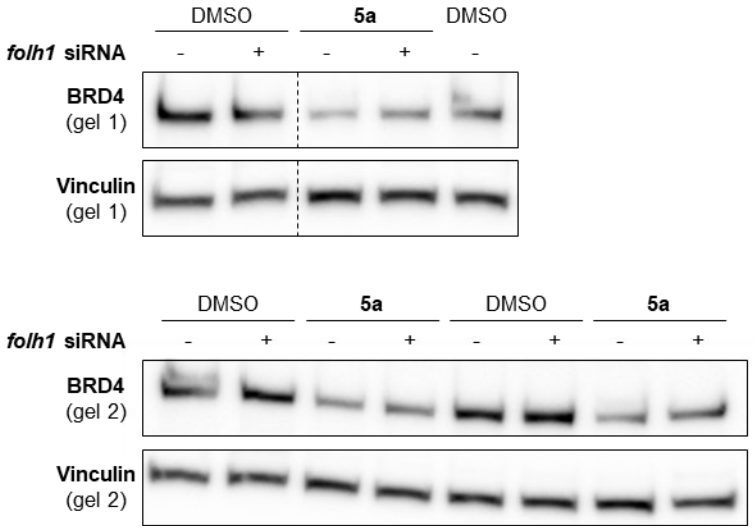

Figure S28. Images of all Western blots used for quantification in Figure 6H: BRD4 levels in LNCaP cells treated for 2 h with 10 nM **5a**, 72 h after siRNA-mediated knockdown of *folh1* (or non-targeting siRNA = -). Solid boxes encompass bands from same membrane section, dashed lines indicate where blot has been cropped to remove irrelevant lanes.

2. Materials and Methods

Materials

L-Glutathione reduced was purchased from Sigma Aldrich (G4251), esterase from porcine liver from Sigma Aldrich (E3019), **JQ1** from MedChemExpress (HY-778695), and a test amount of **MZ1** and **cis-MZ1** were kindly provided by Boehringer Ingelheim via its open innovation platform opnMe, available at https://opnme.com. All other compounds were synthesized in house: see section 4 for details on synthetic procedures and chemical characterization.

UHPLC-MS

Samples were analysed using a Vanquish™ Horizon Duo UHPLC System (Thermo Fisher Scientific) connected to a VC-D11A UV-Vis diode-array detector (Thermo Fisher Scientific) and an ISQ-EM quadrupole MS using a heated ESI source (Thermo Fisher Scientific).

Separation was performed according to methods I or II below. UV-Vis spectra were recorded between 190 and 400 nm at a bunchwidth of 4 nm and a data collection rate of 5-20 Hz. The mass spectrometer was operated in the positive and negative electrospray ionization mode at 3000 V and -2000 V source voltage, respectively (227 °C vaporizer and 300 °C sheath gas temperature, 42.9 psig (2.96 barg) N_2 sheath gas pressure). Spectra were recorded in the mass range from m/z 400 to 2000 at a rate of 2.0 Hz.

Method I: Acquity BEH C18 HPLC column (1.7 µm particle size, 2.1x150 mm; Waters) kept at 65 °C. The mobile phase consisted of A: H2O + 0.1% HCOOH and B: MeCN, with a gradient from 30 to 80% B over 5 min at a flow rate of 500 µL min^{-1}.

Method II: Acquity BEH C18 HPLC column (1.7 µm particle size, 2.1x50 mm; Waters) kept at 65 °C. The mobile phase consisted of A: H2O + 0.1% HCOOH and B: MeCN, with a gradient from 25 to 80% B over 1.3 min at a flow rate of 1000 µL min^{-1}.

Esterase stability experiment

Using a polyethylene 96 well-plate, to the compounds in DMSO was added PBS 1X (0.01 M) solution of porcine liver esterase (0, 1 or 5 U/ml) to give a final compound concentration of 20 µM and 10% DMSO. The plate was film-sealed and incubated at 37 °C for 4 h. The plate was cooled down on ice and the wells were diluted 1:1 with ice-cold MeCN containing the internal standard (**VH032**; 80 µM, final conc. 40 µM). The plate was centrifuged (4 °C, g = 3780; 15 min) after which the supernatant was transferred onto a new polyethylene plate and analysed by UHPLC-MS using method II. The quantification of **MZ1** release was carried out by measuring the peak areas (254 nm) and calculating the concentration based on a previously obtained calibration curve of **MZ1** in the presence of the internal standard.

FBS stability experiments

Using a polyethylene 96 well-plate, to the compounds in DMSO, including **MZ1**, were added PBS with or without FBS to give a final compound concentration of 10 µM with 10% DMSO and 10% FBS. The plate was film-sealed and incubated at 37 °C for 4 h. The plate was cooled down on ice, and the wells were diluted 1:1 with ice-cold MeCN. The plate was centrifuged (4 °C, g = 3780; 15 min) after which the supernatant was transferred onto a new polyethylene plate and analysed by UHPLC-MS using method II. The quantification of **MZ1** release was carried out by measuring the peak areas at 254 nm. Control samples of **MZ1** were used to normalise for the maximum concentration (5 µM) of **MZ1** recoverable in each analyte sample.

Stability experiments in varying pH

PBS 1X (0.01 M) aliquots were adjusted to pH 4, 6 and 9. To the compounds (final conc. 10 µM) and the internal standard (**VH032**; final conc. 40 µM) in DMSO (final conc. 10%) were added buffers at different pH. The samples were incubated inside the UHPLC autosampler at 37 °C for

~5 h. Every 1.7 h, an aliquot from the sample was directly injected to UHPLC-MS using method I and analysed. The quantification was carried out based on the internal standard by measuring the peak areas (254 nm), and the **MZ1** concentration was calculated based on a previously obtained calibration curve of **MZ1** in the presence of the internal standard.

GSH cleavage experiments - time course
The compounds (10 µM) were incubated in PBS 1X (0.01 M) (pH 7.4; 10% DMSO) with GSH (2 mM) and an internal standard (**VH032**; 40 µM) (total sample volume 200 µL) inside the UHPLC autosampler at 37 °C for ~4 h. Every 11 min, an aliquot from the sample was directly injected into the UHPLC-MS using method I and analysed. The quantification was carried out based on the internal standard by measuring the peak areas (254 nm), and the **MZ1** concentration was calculated based on a previously obtained calibration curve of **MZ1** in the presence of the internal standard. For details of kinetic analysis and reaction simulations, see section 3. Mechanistic studies of the GSH-mediated release mechanism.

GSH cleavage experiments - GSH concentration dependence
Using a 96 well-plate, to the compounds in DMSO (1 replicate for each time point) were added GSH solutions at varying concentrations (0.625-20 mM) in PBS 1X (0.01 M) with pH adjusted to 7.4, to give a final compound concentration of 10 µM and 10% DMSO. The plate was film-sealed and incubated at 37 °C for 4 h. At stated timepoints, the respective samples were removed from the plate and diluted 1:1 on a separate polyethylene 96 well-plate with ice-cold MeCN containing the internal standard (**VH032**; 40 µM, final conc. 20 µM). The diluted samples were analysed by UHPLC-MS using method II. The quantification was carried out based on the internal standard by measuring the peak areas (254 nm), and the **MZ1** concentration was calculated based on a previously obtained calibration curve of **MZ1** in the presence of the internal standard.

Cell culture

LNCaP cells were cultured in RPMI1640 ATCC formulation (Gibco A10491-1) and PC3 cells in RPMI1640 (Gibco 21875034). All culture media was supplemented with 10% FBS (Gibco 10270106) and penicillin-streptomycin (Gibco 15140), and cells were maintained in a humidified incubator with 5 % CO_2 at 37 °C. The identity of LNCaP and PC3 cells were authenticated by STR profiling (performed by Microsynth).

Flow cytometry

For flow cytometry experiments, $1x10^5$ LNCaP cells/well were plated (or reverse transfected with siRNAs where relevant) on 12 well plates 72 h prior to compound treatment. Following compound treatment as described at a final concentration of 0.1 % DMSO (0.2 % where two compounds added in a blocking experiment), cells were harvested by trypsinization using TrypLE Express (Gibco 12605010). Cells were washed twice in ice cold FACS buffer (PBS supplemented with 2 % FBS and 0.5 mM EDTA), resuspended in FACS buffer containing 0.4 µg/ml propidium iodide (BioLegend 421301) and stored on ice until analysis.

Cells were analyzed using a FACSymphony and FlowJo™ v10 software (BD Biosciences). Gating was performed based on FSC-A vs. SSC-A (to identify cells), FCS-A vs. FSC-H (to select single cells) and propidium iodide staining intensity (to exclude dead cells [excitation 561 nm, emission 610/20 nm]). FITC staining intensity was measured using an excitation of 488 nm and emission of 530/30 nm. The median FITC staining intensity of live single cells was calculated for cells from each well and plotted as a single data point.

Representative gating strategy used to identify live single LNCaP cells:

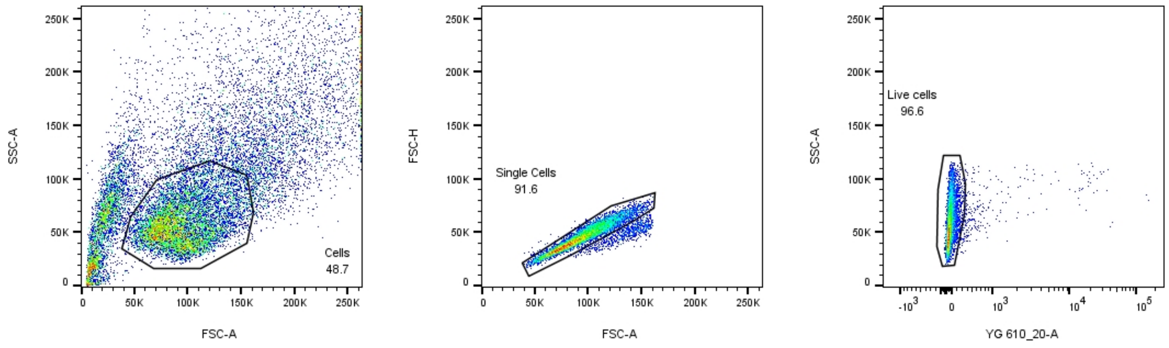

Representative gating strategy used to identify live single PC3 cells:

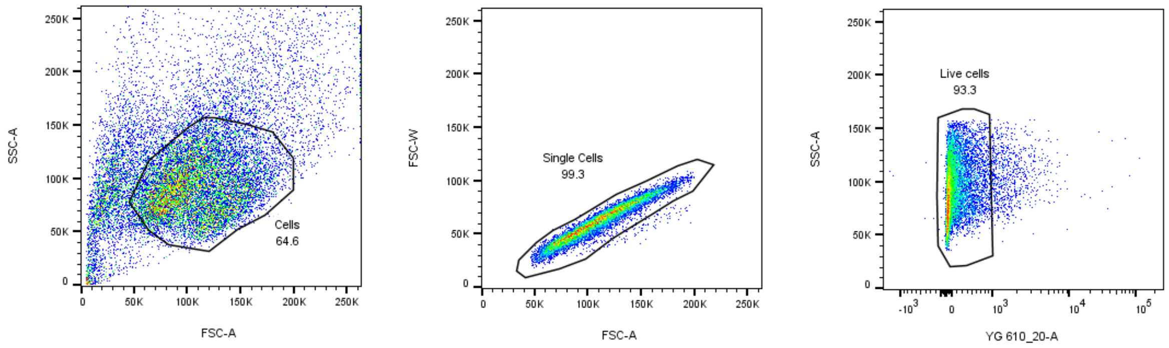

siRNA knock down (*folh1*)

For siRNA-mediated knockdown, reverse transfection was performed 72 h before compound treatment. On a 12-well plate, transfection mixtures were prepared containing 12 pmol siRNA and 1 µl Lipofectamine RNAiMax (Invitrogen 13778030) in 200 µl Opti-MEM (Gibco 31985062) according to manufacturer's instructions. For knock-down of *folh1* ON-TARGETplus human FOLH1 (2346) was used (Dharmacon J-005881-06-0002, sequence: GCAGAGCACGGUAUACUAA), and the ON-TARGETplus Non-Targeting Pool (Dharmacon D-001810-10-20) was used as a control. 1x10^5 LNCaP cells per well were plated on top of the prepared transfection mixtures in 1ml of antibiotic free medium. Shortly before compound treatment, the transfection reagents were removed through a complete media change. Cells were then treated with compounds as described at a final concentration of 0.1 % DMSO and subsequently processed for flow cytometry or Western blotting.

Confocal microscopy

For confocal microscopy, a µ-Slide 8 Well high ibiTreated microscopy chamber slide with a #1.5 Polymer cover slip (ibidi 80806) was pre-coated with 0.01 % poly-lysine solution (EMS-19320-B) for 5 min at room temperature before being washed with water and dried. 4x10^4 LNCaP cells/well were plated, and treated with compounds as described 48 h later with a final concentration of 0.1 % DMSO. Following compound treatment, cells were washed with room temperature PBS, fixed with 4 % PFA for 10 min, washed again, stained with DAPI (0.8 µg/ml) for 10 min, washed, and finally covered with mounting medium (ibidi 50001). The slide was stored in the dark at 4 °C until imaging.

Imaging was performed using a laser scanning confocal microscope (Leica SP8) using two HyD detectors: for DAPI (ex. 405 nm laser) 412-480 nm, for FITC (ex. 488 nm laser) 495-570 nm. 3 positions were chosen per well, and a z-stack acquired with 140 nm step size using a 63x oil

immersion lens. Images were acquired at a resolution of 1048x1048 pixels (pixel size of 58 nm XY), using a scan speed of 500 with 4x accumulation.

Deconvolution of z-stacks was performed using the deconvolution wizard in Huygens Professional software without modification of the point spread function (PSF). The classic maximum likelihood estimation (MLE) algorithm was used, with the background value set to 0. Using imageJ, deconvoluted images were converted to hyperstacks, the same maximum brightness applied to images from all conditions to ensure comparability, and the representative images shown in Figure S11 were generated using the orthogonal viewer.

Cell viability

For cell viability measurements, $5x10^3$ LNCaP cells or $2x10^3$ PC3 cells were plated per well on 96 well plates 24 h prior to compound treatment. Cells were treated with compounds as described at a final DMSO concentration of 0.1%. After 3 days of treatment, resazurin (Acros Organics) was added at a final concentration of 86 µM and incubated for 1h30 (LNCaP) or 2h30 (PC3) at 37 °C, before fluorescence (ex. 560 nm, em. 590 nm) was read using a SpectraMax M5 (Molecular devices). Cell viability was calculated relative to DMSO-treated cells on the same plate. Data was fitted using a four-parameter dose-response curve in GraphPad Prism v. 10.5.0, where the top plateau was fixed at 100%.

For plots showing GI_{50} values, GI_{50} values from each independent experiment were calculated as the concentration required to inhibit cell viability to 50 % relative to DMSO-treated cells, taking all replicate wells into account (curves and standard deviations of replicate wells per experiment are shown in supplementary figures). pGI_{50} values were calculated for plotting:

$$pGI_{50} = -\log_{10}(GI_{50})$$

Plots display a single point for each independent experiment, and the geometric mean of replicates is displayed as a bar. This geometric mean was exponentiated to obtain the geometric mean GI_{50} for each compound, displayed as a numerical value at the top of the plot and quoted in the text:

$$mean\ GI_{50} = 10^{-(mean\ pGI_{50})}$$

For plots showing full dose response curves, the mean of replicate wells for each compound dose from a single experiment was calculated, plotted as a single data point and these used to fit one curve per compound (curves and standard deviations of replicate wells per experiment are shown in supplementary figures).

Quantification of BRD4 by Western blot

For quantification of BRD4 protein levels by Western blotting, $4x10^5$ LNCaP cells/well or $2.4x10^5$ PC3 cells/well were plated on 6 well plates 48 h prior to compound treatment. Cells were treated with compounds as described at a final concentration of 0.1 % DMSO (0.2 % where two compounds simultaneously added in a blocking experiment). Following treatment, cells were washed with ice cold PBS and lysed on ice in RIPA buffer (50 mM tris pH 7.5, 2 mM EDTA, 150 mM NaCl, 0.5% sodium deoxycholate, 1% Triton X100) supplemented with cOmplete™ protease inhibitor cocktail (Roche), by scraping and resuspension by pipetting. Lysates were clarified by centrifugation, and protein concentrations determined using the Pierce™ BCA Protein Assay Kit (Thermo Scientific, 23227). Equal amounts were separated on 4-12% tris-glycine AccuPAGE™ PAGE gels (AP GBP-41215), then transferred to PVDF membranes. Membranes were blocked with 5% milk (Merk Millipore, 115363) in PBS-T and proteins were stained with the following antibodies at 4 °C overnight: BRD4 (Bethyl Laboratories, A301-985A50, diluted 1:2000) and vinculin (Invitrogen, 42H89L44, diluted 1:1000). For detection, membranes were incubated for 1 h at room temperature with anti-rabbit HRP-conjugated secondary antibodies (Southern

Biotech, 6415-05, 1:5000 dilution), and were imaged using SuperSignal™ West Pico PLUS Chemiluminescent Substrate (Thermo Scientific, 34580) and the Vilber Fusion-FX7 imager. Bands were quantified using ImageJ software, and BRD4 levels were normalized firstly to vinculin as a loading control, and then to protein from DMSO-treated cells run on the same gel.

Molecular dynamics simulations

Initial 3D conformations of the PROTACs were generated using OpenBabel[5] (version 3.1.0). Force field parameters for the PROTACs were obtained using the CHARMM General Force Field (CGenFF) bond-recognition and atom-typer program[6] (version 2.5.1) and version 4.6 of CGenFF.[7] Bond, angle, and dihedral parameters with high CGenFF penalty values were optimized with the help of FFParam[8] (version 1.2.0). To this end the PROTAC was manually fragmented and fragments containing high penalty values were optimized independently.

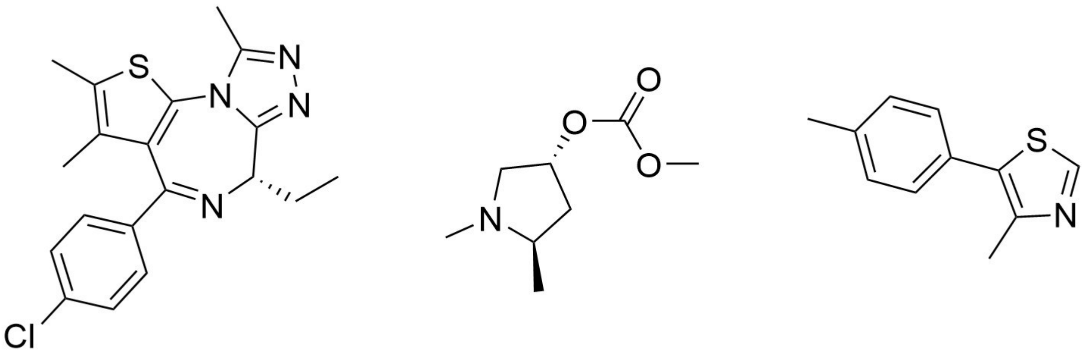

Figure S29. The three fragments of the PROTAC whose bond, angle, and/or dihedral parameters were optimized.

Each of the three fragments was subjected to molecular mechanics (MM) energy minimization using the CHARMM[9] (version 48b1) software and the unoptimized CGenFF parameters followed by quantum mechanical (QM) energy minimization at the MP2 theory level using the 6-31G* basis set in the Gaussian16[10] software. For each internal coordinate with high CGenFF penalty values or high disagreement between the MM and QM optimized geometries an MM and a QM potential energy surface (PES) scan was performed. The MM parameters were then iteratively optimized to match the QM PES as closely as possible. The optimized parameters were converted to GROMACS format with the cgenff_charmm2gmx_py3_nx2[11] python script using the CHARMM36[12] and CGenFF[7] 4.6 force fields.

The following steps were performed with GROMACS[13] (version 2024.2). Each PROTAC was placed in a cubic box and solvated with either TIP3P water[12] or with chloroform[14]. All systems solvated in water were neutralized with Na^+ ions and 150 mM of NaCl salt was added. After steepest descent energy minimization the systems were equilibrated at 300 K for 1 ns using the canonical ensemble with a time step of 2 fs and utilizing the LINCS algorithm[15] to constrain hydrogen atoms. A velocity-rescaling thermostat[16] with a coupling time of 0.1 ps was employed. During equilibration the PROTAC position was restrained. After releasing the PROTAC position restraints an initial simulation with a wall time of 20 h (simulated time is system specific, at least 100 ns) was performed. After discarding the first 10th of the simulation, 10 equally spaced snapshots were taken from this trajectory as starting configurations for the production runs. After reinitializing the velocities of each selected snapshot, the 10 independent production runs were started with a wall time of 72 h for compound **2a**, and 72 h or 48 h for water and chloroform respectively for compound **5a**. The simulated times are system specific, and are between 400 and 1000 ns. The cumulative sampling was 10.4 and 26.2 μs for **2a** in water and chloroform, respectively, and 4.4 and 9.2 μs for **5a**. To check for convergence the 10 independent simulations of each system were divided into two groups, analysed independently and compared before the combined analysis with all 10 trajectories was performed. The analysis for convergence by block averaging over two blocks of runs is depicted in Figures S30 – S31. The simulations in water and

in chloroform without ions showed acceptable convergence. The sampled PROTAC configurations were clustered using the GROMACS single linkage clustering algorithm applied to non-hydrogen atoms of the PROTAC with the RMSD cutoff as indicated in the main text.

For the simulations of **5a** in chloroform with seven Na^+ ions to neutralize the system, the electrostatic interaction between the ions and the negatively charged carboxylates of the PROTAC was very strong. To get diverse starting structures, the initial simulation was done at 500 K instead of 300 K. After discarding the first 10th of the simulation and selecting 10 equally spaced snapshots, the production simulations were performed at 300 K and 360 K. The elevated temperature (360 K) is used solely to improve sampling[17] as the force field does not have any explicit temperature dependence. The cumulative sampling at 300 K was 5.3 μs and 8.7 μs at 360 K. For the simulations at 300 K, sampling was poorer and the PROTAC did not deviate far from the initial configuration. The simulations at 360 K showed much better convergence while the PROTAC properties were similar to the simulations at 300 K.

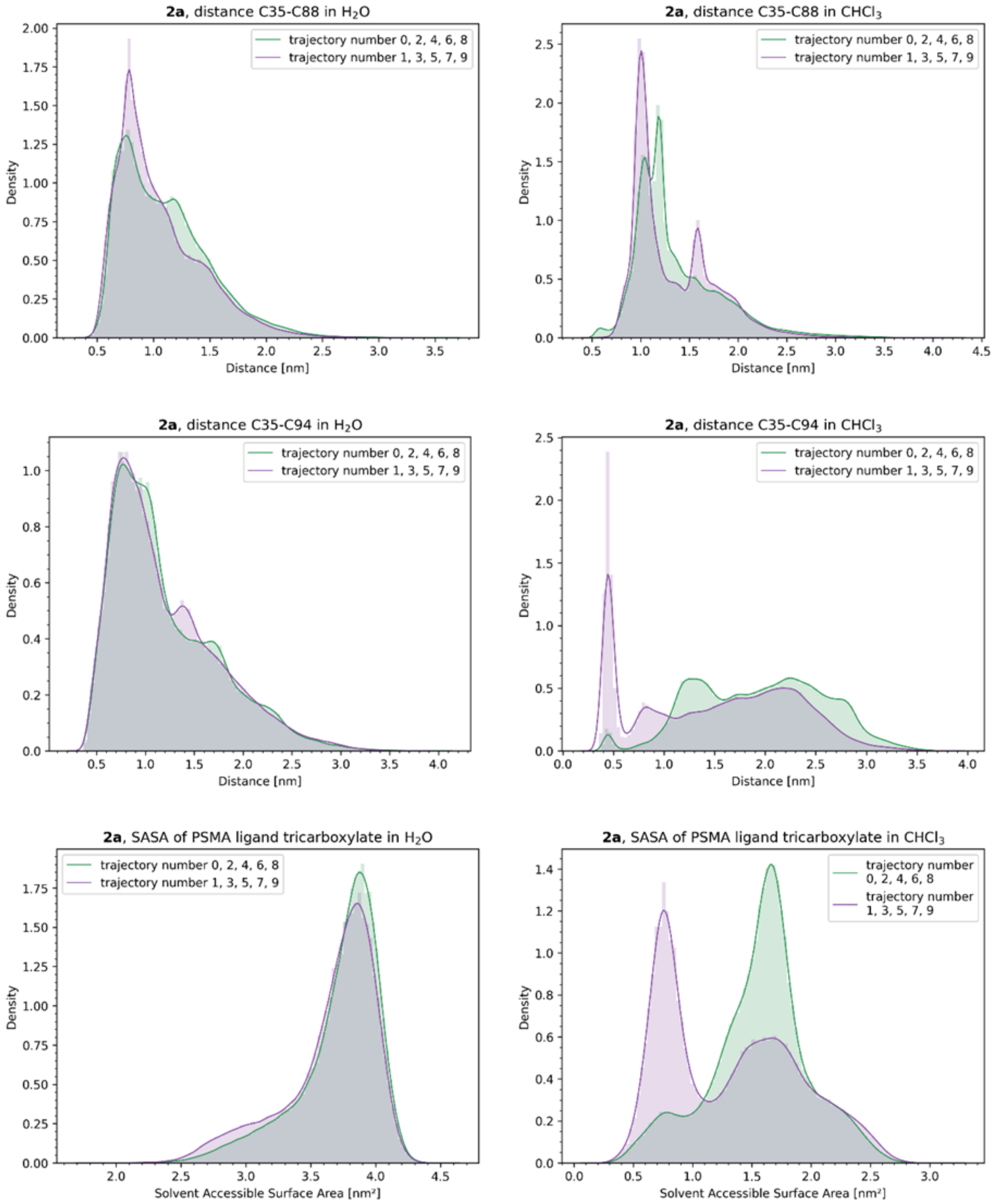

Figure S30. Distance and solvent accessible surface area (SASA) for MD simulations of compound **2a** with the 10 trajectories at 300 K split into two groups.

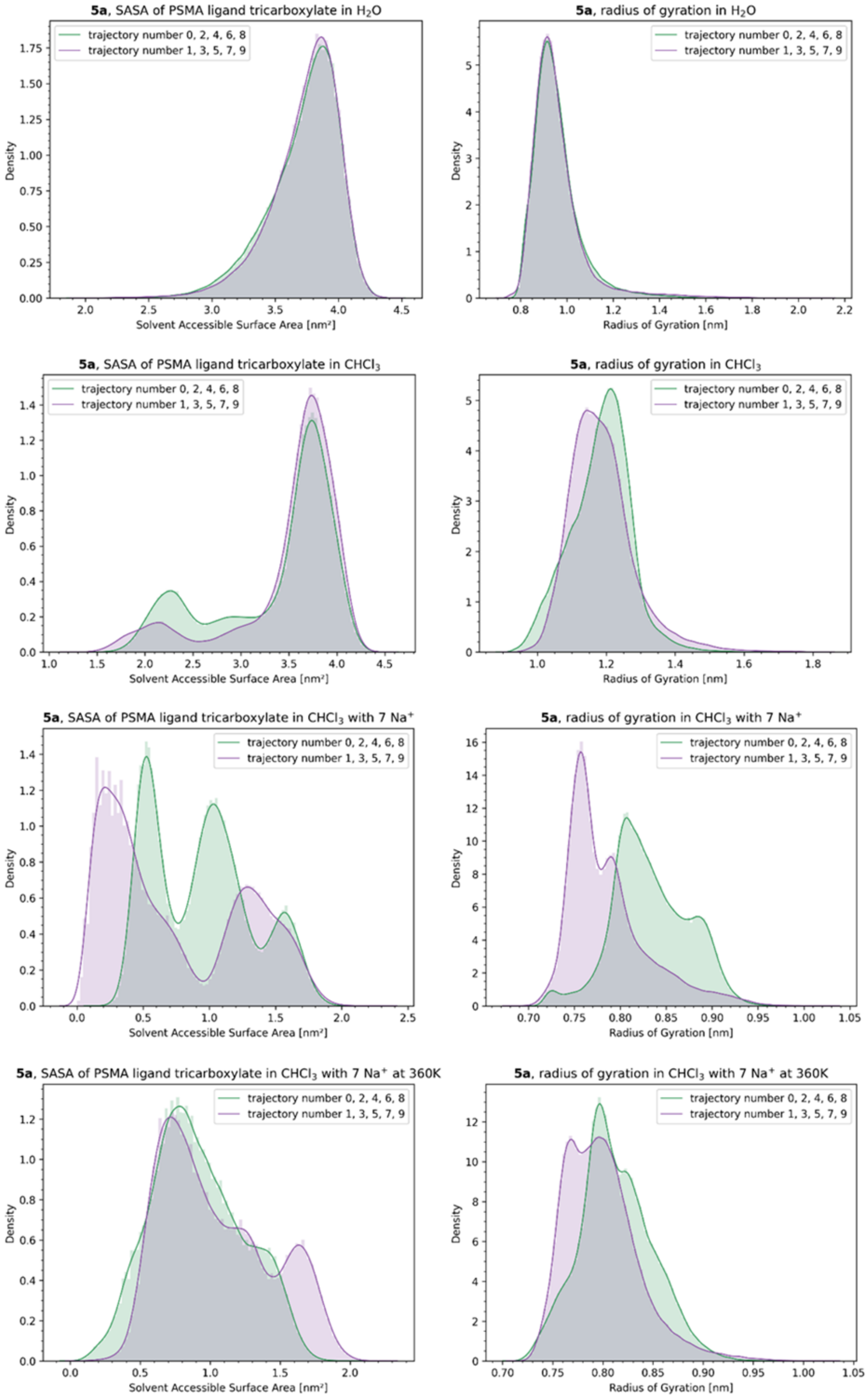

Figure S31. Solvent accessible surface area (SASA) and radius of gyration for MD simulations of compound **5a** at 300 K (top 3 rows) and 360 K (last row) with the 10 trajectories split into two groups.

Data analysis

Data was analysed using excel, ImageJ and R, and graphs prepared using GraphPad Prism v. 10.5.0 or R. Error bars show ± standard deviation, individual data points are shown to indicate the n number. PyMOL was used for the visualization of protein structures and compound conformations resulting from the molecular dynamics simulations.

3. Mechanistic Studies of the GSH-Mediated Release Mechanism

Data collection and analysis

Samples were prepared and measured according to the method described in Materials and Methods section. Chromatograms from UHPLC-MS were analysed using Chromeleon 7. The peaks were identified by MS and integrated (254 nm). Peaks areas were compared to the internal standard to calculate the temporal concentrations of the analytes and intermediates. Peak areas of final product **MZ1** were compared to the internal standard and a previously obtained calibration curve, to determine the temporal concentration.

Observed rate constant (k_{obs})

Temporal concentrations of starting material prodrugs were plotted by $\ln([I]/[I]_0)$ vs time (Figure S6). Based on the plots, it was assessed that all compounds follow a pseudo-first-order kinetics up to at least 60% decay, as these data points fall within the linear section of the curve. Thus, a linear fit was obtained for data points covering 50-60% of decay, where the negative slopes represent the observed rate constant k_{obs}.

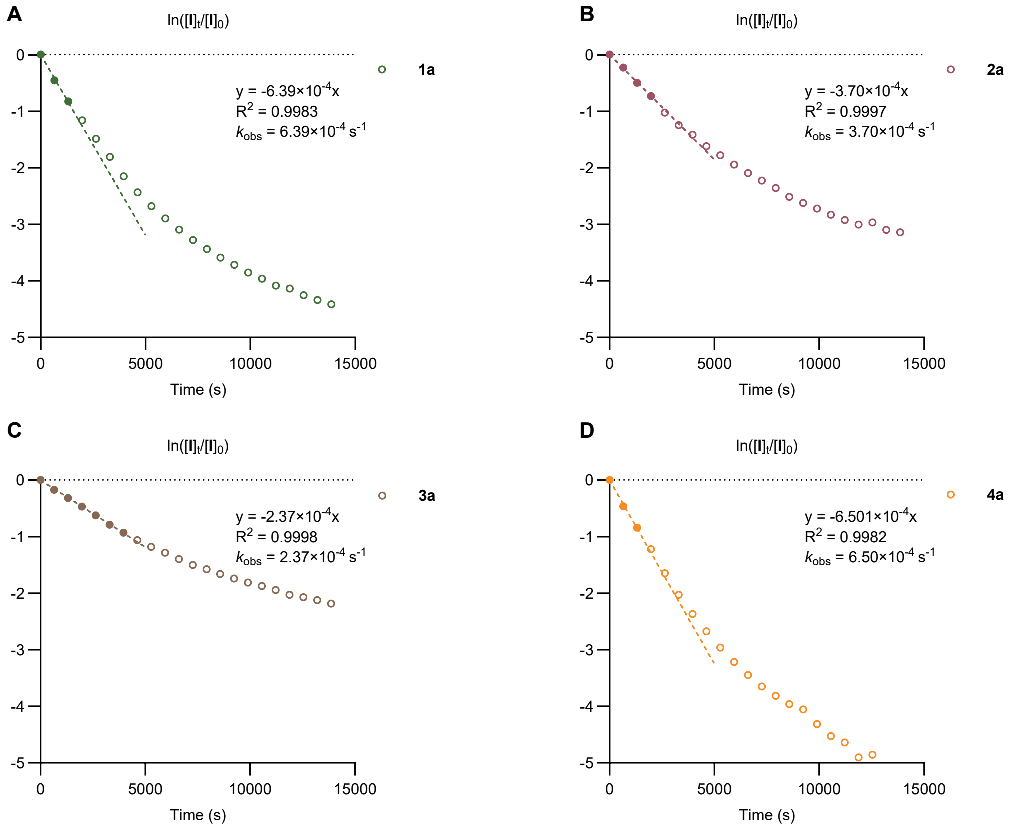

Figure S8. Plots of $\ln([I]/[I]_0)$ vs time for compounds **1a**-**4a** in the presence of GSH (2 mM), indicating a pseudo-first-order decay up to 50-60% of decay. The equations display the observed rate constants (-slope) and correlation factor (R^2).

Initial rate of MZ1 release (v_0)

Temporal concentrations of **MZ1** were plotted vs time (Figure S7). The linear sections of the curves were used to obtain a fit for each compound. The slope of the resulting equations corresponds to the initial rate (v_0).

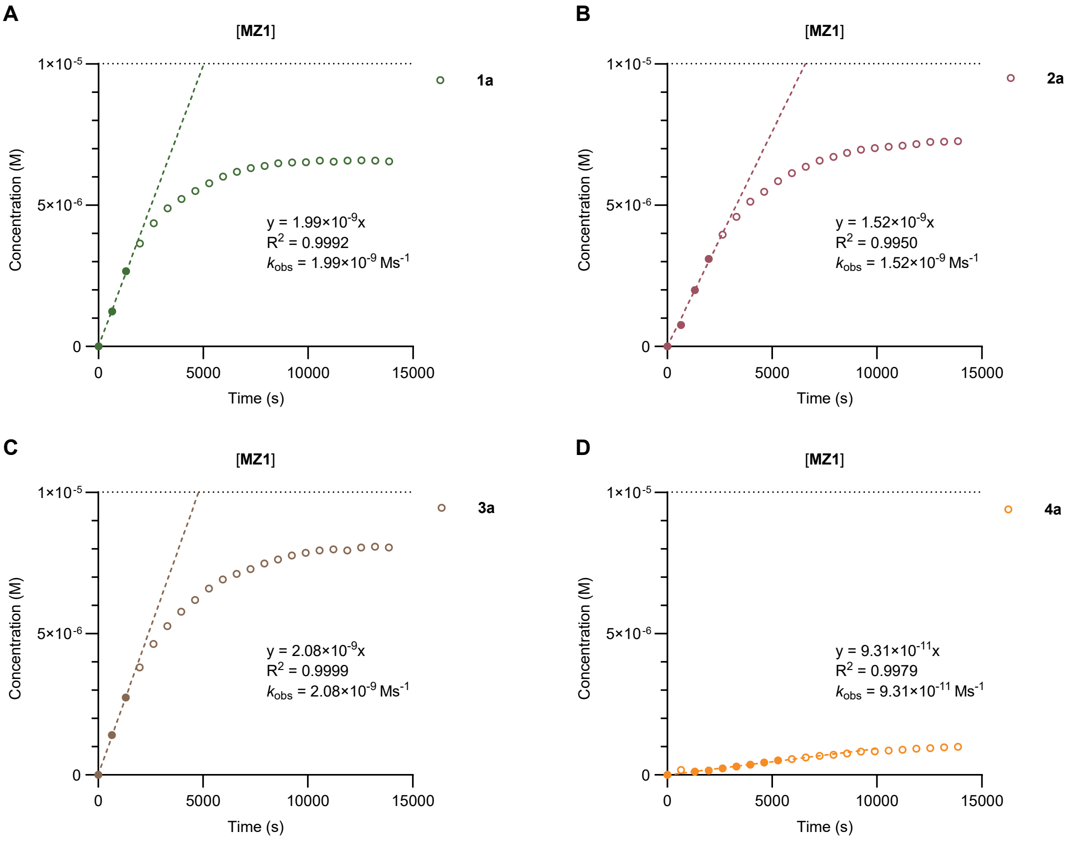

Figure S9. Plots of [**MZ1**] vs time for compounds **1a**-**4a** in the presence of GSH (2 mM). A fit was obtained in the linear section of the curve (for **1a**-**3a** 27-30% conversion; for **4a** 5% conversion, one outlier datapoint within the linear section not included in the fit). The equations display the observed rate constants (slope) and correlation factor (R^2).

Kinetic simulations

The experimentally calculated temporal concentrations of the observed species were used to simulate the kinetic profiles with Dynochem 6 ™.

The model was built by defining:

1) All elementary steps involved in the reaction mechanism for each compound (Schemes S1-S4). Beyond the basic elementary steps discussed in the manuscript:
   a. GSH oxidation to GSSG was incorporated for each simulation.
   b. for **3a** an additional non-specific degradation to **MZ1** was incorporated based on experimental evidence (Figure 1B and C in the article main text).
   c. for **4a** a step from the reactive intermediate **II** to an undetected degradation product **X*** was incorporated, to account for a clear loss of mass balance in the experiment.
2) All reaction components present in the defined elementary steps and their molecular weights.
3) The reaction conditions: 37 °C, 200 μL volume.
4) Initial reagent concentrations for **I** (1×10^{-5} M) and GSH (2×10^{-3} M)
5) As an initial starting point, rate constants (*k*) for each elementary step were assigned as 1 $M^{-1} \cdot s^{-1}$ or s^{-1}, and equilibrium constants (K_{eq}) for largely irreversible steps were assigned as 10 and for irreversible steps as 1000 in appropriate units.

Iterations of the simulation:

1) The rate and equilibrium constants were modified for each step in small increments, and the quality of the simulation was assessed by eye.
2) The same was repeated for the equilibrium constants.
3) Reaction step involving GSH oxidation to GSSG was optimised for compound **4a**, and the same constants were applied in the simulations of other compounds.
4) As a last stage, equilibrium constants were altered iteratively to assess the range where the simulation sensitivity is low.

The rate and equilibrium constants that were used in plotting the simulation results on Figure 2A-D, and the low-sensitivity ranges for equilibrium constants are represented in Table S1.

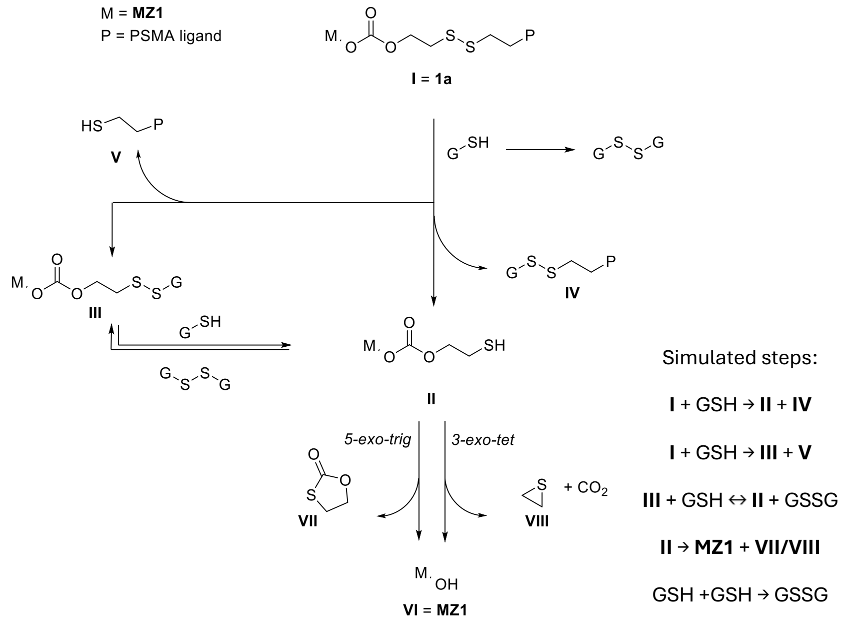

Scheme S1 GSH-mediated cleavage mechanism elementary steps included in the Dynochem simulation for primary carbonate **1a**.

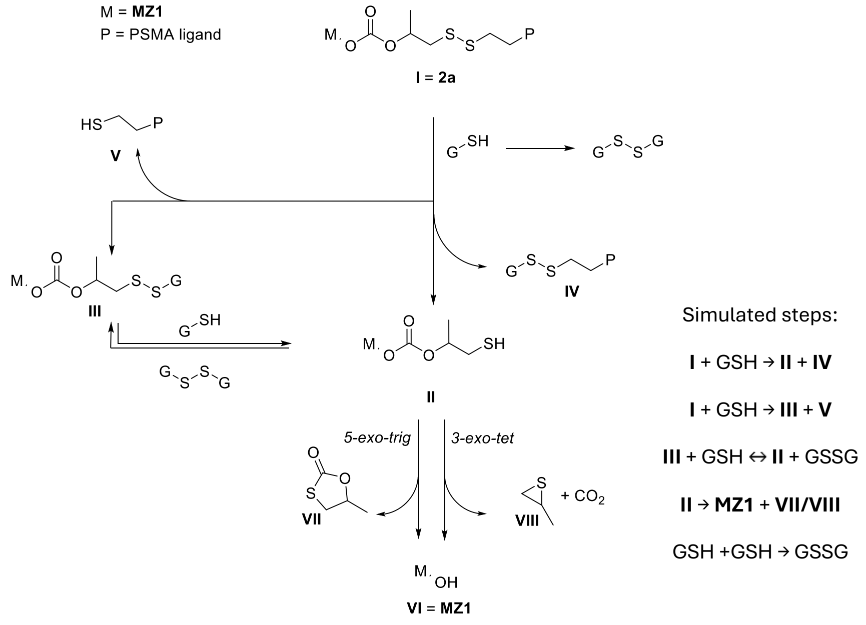

Scheme S2 GSH-mediated cleavage mechanism elementary steps included in the Dynochem simulation for secondary carbonate **2a**.

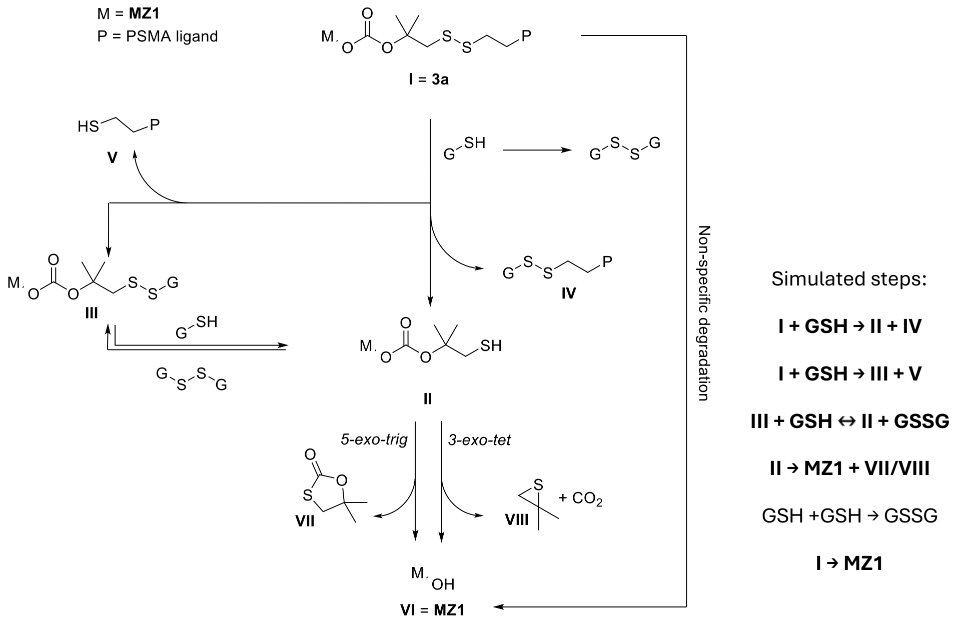

Scheme S3 GSH-mediated cleavage mechanism elementary steps included in the Dynochem simulation for tertiary carbonate **3a**.

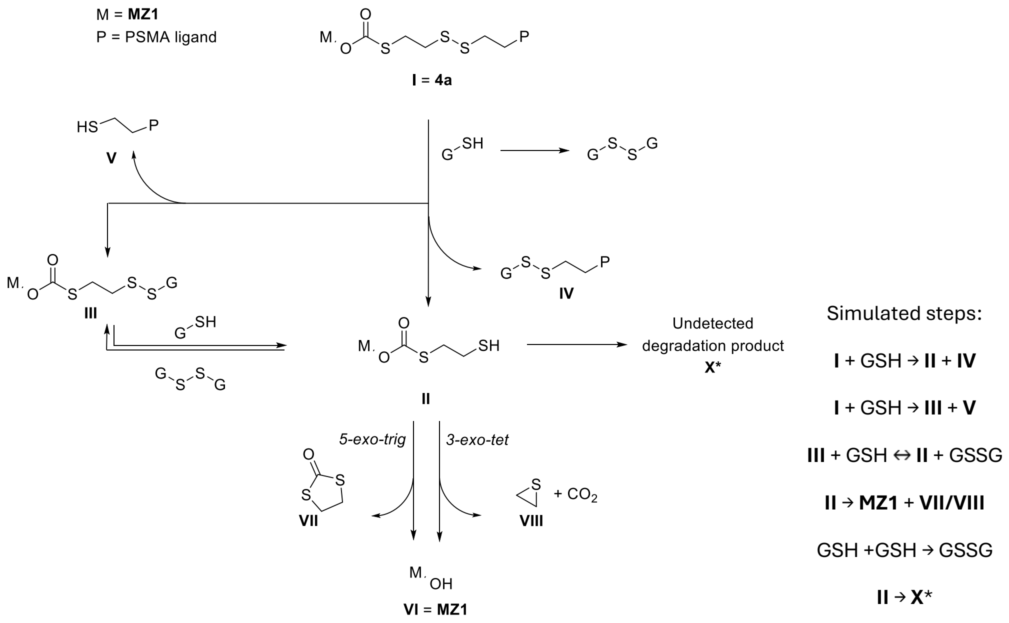

Scheme S4 GSH-mediated cleavage mechanism elementary steps included in the Dynochem simulation for thiocarbonate **4a**.

	1a	2a	3a	4a	
**I** + GSH → **II** + **IV**	0.15	0.15	0.125	0.15	*k* ($M^{-1}·s^{-1}$)
	10<br>*(5×10⁻⁴-1000)**	10<br>*(0.02-1000)**	10<br>*(0.001-1000)**	10<br>*(0.1-1000)**	K_{eq}
**I** + GSH → **III** + **V**	0.18	0.1	0.02	0.22	*k* ($M^{-1}·s^{-1}$)
	10<br>*(0.5-1000)**	10<br>*(0.05-1000)**	10<br>*(0.002-1000)**	10<br>*(0.5-1000)**	K_{eq}
**III** + GSH ↔ **II** + GSSG**	0.1	0.08	0.1	0.15	*k* ($M^{-1}·s^{-1}$)
	1<br>*(0.05-1000)**	1<br>*(1-1000)**	1<br>*(0.05-1000)**	0.3	K_{eq}
**II** → **MZ1** + **VII/VIII**	0.1	0.0003	0.1	4.5×10^{-5}	*k* (s^{-1})
	1000<br>*(0.05-1000)**	1000<br>*(0.05-1000)**	1000<br>*(0.005-1000)**	1000<br>*(5×10⁻⁶-1000)**	K_{eq} (M)
GSH + GSH → GSSG	0.05	0.05	0.05	0.05	*k* ($M^{-1}·s^{-1}$)
	1000	1000	1000	1000	K_{eq} (M^{-1})
**I** → **MZ1**			0.0005		*k* (s^{-1})
			1000<br>*(50-1000)**		K_{eq}
**II** → **X***				0.0003	*k* (s^{-1})
				6	K_{eq}

** Range where the simulation showed low sensitivity to the value.*

***When considering the reversibility of each elementary step, defined by the equilibrium constant (K_{eq}), due to the fast reaction kinetics the simulated kinetic profiles of almost all substrate-involving steps generally showed low sensitivity towards their reversibility. However, for the thiocarbonate **4a** we obtained a defined constant for the interconversion between **III** and **II** ($K_{eq\ III→II}$ = 0.3), indicating an equilibrium shift towards intermediate **III** that was indeed observed to be accumulating at a higher concentration in the reaction mixture than **II** (Figure 2D in the article main text). Although here it was possible to more precisely define the equilibrium (K_{eq}) due to the greatly reduced rate of the downstream cyclization of **II**, it is clear that the cyclization controls the rate of **MZ1** release for this compound.*

Table S1 Simulated rate and equilibrium constants for each elementary step in the GSH-mediated release mechanism (Schemes S1-S4) of compounds **1a**-**4a**, which were used in the plotted simulation results in Figures 2A-D. Additionally, ranges given for values where the simulation showed low sensitivity.

4. Synthetic Experimental Procedures, Schemes and Compound Characterisation

General experimental procedures

Characterisation of compounds

NMR spectra were recorded on AV 300, AV2 400 or AV2 500 MHz Bruker spectrometers. The spectra are calibrated to the residual ^1H and ^{13}C signals of the solvents. Chemical shifts are reported in ppm and the spectra are calibrated using the residual chloroform signals (7.26 ppm for ^1H NMR and 77.16 ppm for ^{13}C NMR), the residual DMSO signals (2.50 ppm for ^1H NMR and 39.52 ppm for ^{13}C NMR), the residual methanol signals (3.31 ppm for ^1H NMR and 49.00 ppm for ^{13}C NMR), the residual acetone signals (2.05 ppm for ^1H NMR and 29.84 ppm for ^{13}C NMR) and the residual dichloromethane signals (5.32 ppm for ^1H NMR and 53.84 ppm for ^{13}C NMR). Multiplicities are abbreviated as follows: singlet (s), doublet (d), triplet (t), quartet (q), doublet-doublet (dd), quintet (quint), multiplet (m), and broad (br). High resolution electrospray ionization mass spectrometry (HRMS (ESI)): Dionex Ultimate 3000 UHPLC system (ThermoFischer Scientifics, Germering, Germany) connected to a QExactive MS with a heated ESI source (ThermoFisher Scientific, Bremen, Germany); mass calibration to <2 ppm accuracy with Pierce® ESI calibration solns. (ThermoFisher Scientific, Rockford, USA).

Synthetic and purification methods

Reactions were carried out under ambient atmosphere unless anhydrous conditions are explicitly mentioned; in that case, reactions were carried out under a nitrogen atmosphere using standard Schlenk techniques. All reagents were used as received unless otherwise noted. Solvents were purchased in the best quality available. For anhydrous solvents, the solvents were purged with argon and passed through alumina columns in a solvent purification system (Innovative Technology). Reactions were monitored by thin layer chromatography (TLC) using Merck TLC silica gel 60 F_{254} and using UV light (254 nm) as a visualizing agent and acidic ceric ammonium molybdate/ phosphomolybdic acid, potassium permanganate or vanillin solutions with heat as developing agents. Column chromatography was performed over silica gel (230-400 mesh) under nitrogen or air pressure.

Cu(I)-catalysed azide-alkyne cycloaddition (CuAAC)

General Procedure A:

The azide (1 equiv), alkyne **S32** (prepared according to the previously reported procedure[18])(1.5 equiv) and tris((1-benzyl-4-triazolyl)methyl)amine (TBTA) (0.2 equiv) were combined and dissolved in *N*-methyl-2-pyrrolidone (NMP) (final concentration 0.02 M). To the solution $Cu_2SO_4{\cdot}5H_2O$ (50 mM solution in NMP; 1.5 equiv) and sodium ascorbate (100 mM suspension in NMP; 1.5 equiv) were sequentially added. The reaction was stirred for 16 h and diluted with ice water (2 mL) to precipitate the product. The mother liquor was removed, and the trituration was repeated once more. The solids were dissolved in a mixture of MeCN and water (1:1) with 0.1% formic acid, filtered and purified by reverse-phase HPLC using a Shimadzu Nexera system, equipped with an Agilent Zorbax 300SB-C18 semi-preparative column (9.4 x 250 mm 5-micron) using 25 to 55% MeCN in water buffered with 0.1% formic acid as eluent (4 mL/min).

General Procedure B:

The azide (1 equiv), alkyne **S36** (1.5 equiv) and TBTA (0.2 equiv) were combined and dissolved in NMP (final concentration 0.0017 M). To the solution $Cu_2SO_4{\cdot}5H_2O$ (100 mM solution in NMP; 3 equiv) and sodium ascorbate (100 mM suspension in NMP; 3 equiv) were sequentially added. The reaction was stirred for 16 h and diluted with ice water (3 mL) to precipitate the product. The solids were pelleted by centrifugation and the mother liquor was removed. The solids were dissolved in aqueous GnHCl (6M) and further diluted with MeCN (up to 30%), filtered and purified

by reverse-phase HPLC using a Shimadzu Nexera system, equipped with an Agilent Zorbax 300SB-C18 semi-preparative column (9.4 x 250 mm 5-micron) using 30 to 60% MeCN in water buffered with 0.1% trifluoroacetic acid (TFA) as eluent (4 mL/min).

Solid Phase Peptide Synthesis (SPPS)

Reagents and solvents for SPPS

Tentagel XV HMPA resin was kindly provided by Prof. Dr. N. Hartrampf Lab, University of Zurich. Dimethylformamide (DMF) used in SPPS was treated with AldraAmine trapping packets (volume 1000-4000 mL; Sigma-Aldrich) at least 24 h prior to use.

Coupling of the first amino acid

SPPS was performed on Tentagel XV HMPA resin (0.29 mmol/g, 1.0 g, 0.29 mmol, 1 equiv). After swelling the resin in a 20 mL fritted syringe for 15 min with DMF, the first amino acid (Fmoc-Lys(Alloc)-OH) was coupled using conditions: Fmoc-Lys(Alloc)-OH (10 equiv), was dissolved in DMF (7 mL). *N,N'*-Diisopropylcarbodiimide (DIC) (5 equiv) and 4-dimethylaminopyridine (DMAP) in DMF (0.2 M, 0.1 equiv) were added and the solution was added to the resin. The reaction was allowed to agitate for 18 h at ambient temperature, after which, the resin was washed with DMF (3 x 15 mL).

General Procedure C: Fmoc-coupling strategy

The Fmoc-group was deprotected after coupling steps by treating the swollen resin with piperdine solution (20% in DMF; 7 ml; cont. 1% TFA) for 5 min while being gently agitated with a spatula, after which the resin was washed with DMF (1 x 15 mL). The procedure was repeated once more ending with a wash with DMF (3 x 15 mL).

To couple a new amino acid (AA), the AA (5 equiv) was dissolved in Hexafluorophosphate Azabenzotriazole Tetramethyl Uronium (HATU) solution (0.38 M in DMF; 4.8 equiv), the solution was made up to ~7 mL with DMF and *N,N*-Diisopropylethylamine (DIPEA) (10 equiv) was added. The solution was allowed to react for 2 min and then added to the swollen resin. The reaction mixture was agitated for 1-1.5 h at ambient temperature, after which, the resin was washed with DMF (3 x 15 mL).

Alloc-sidechain deprotection

The DMF-swelled resin was washed with CH_2Cl_2 (3 x 15 mL) and split equally between two reactor vessels. $Pd(PPh_3)_4$ (3 equiv) was dissolved in CH_2Cl_2 (30 mL), phenylsilane (20 equiv) was added, after which the solution was split and added to the resin in two vessels in parallel. During the reaction, gas development was observed and the resin was gently agitated with a spatula over 60 min at ambient temperature. The resin washed in each vessel with CH_2Cl_2 (3 x 20 mL), DMF (3 x 20 mL) and CH_2Cl_2 (3 x 20 mL). The procedure was repeated once more and finally the deprotected resin from both vessels were combined.

Cleavage from resin, global deprotection and purification

Clevage from resin and global deproctection was carried out using a mixture TFA (94%), triisopropylsilane (TIPS) (1%), H_2O (2.5%) and 2,2 -(Ethylenedioxy)diethanethiol (DODT) (2.5%) (15 mL) for 2 h at room temperature with gentle agitation. The supernatant was then collected by filtration and the resin further washed with TFA (10 mL). The supernatant and wash solution were combined and concentrated under a weak stream of N_2 until 5 mL remained. The peptides were precipitated from ice-cold diethyl ether (-20 °C) (45 mL) and the solids were collected by centrifugation. The volatiles were removed from the pellet under a weak stream of N_2, after which the solids were dissolved in aqueous GnHCl (6M) and further diluted with MeCN (up to 5%), filtered and purified by reverse-phase HPLC using a Shimadzu Nexera system, equipped with an Agilent Zorbax 300SB-C18 semi-preparative column (9.4 x 250 mm 5-micron) using 5 to 35% MeCN in water buffered with 0.1% trifluoroacetic acid (TFA) as eluent (4 mL/min).

Experimental procedures and characterisation

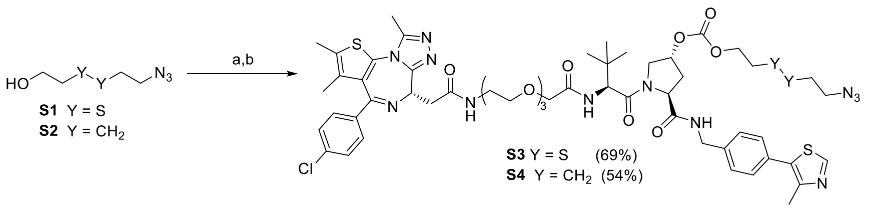

Scheme S5 Synthetic route to obtain the primary carbonate **MZ1**-N_3 intermediates **S3** and **S4**; a) Triphosgene (0.8 or 0.7 equiv), DMAP (6 equiv), CH_2Cl_2 (0.02 M), 0 to 20 °C, 1h; b) **MZ1** (1 equiv), CH_2Cl_2 (0.01 M), 0 to 20 °C, 15 h, 69 & 54% over two steps.

Compound S3 – *2-((2-Azidoethyl)disulfaneyl)ethyl((3R,5S)-1-((S)-2-(tert-butyl)-17-((S)-4-(4-chlorophenyl)-2,3,9-trimethyl-6H-thieno[3,2-f][1,2,4]triazolo[4,3-a][1,4]diazepin-6-yl)-4,16-dioxo-6,9,12-trioxa-3,15-diazaheptadecanoyl)-5-((4-(4-methylthiazol-5-yl)benzyl)carbamoyl)pyrrolidin-3-yl) carbonate*

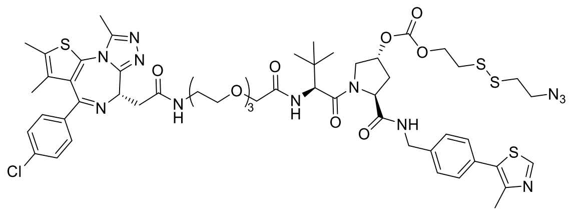

In an oven-dried flask, triphosgene (8.29 mg, 0.028 mmol) was dissolved in anhydrous CH_2Cl_2 (1.7 mL) and cooled to 0 °C. While stirring, dropwise, an ice-cold solution of **S1**[19] (12.5 mg, 0.07 mmol) and 4-dimethylaminopyridine DMAP (25.6 mg, 0.209 mmol) in anhydrous CH_2Cl_2 (1.7 mL) was added over 5 min. The ice bath was removed, and the reaction was allowed to warm up to 20 °C and stirred for 1 h. The solution was cooled to 0 °C and **MZ1** (prepared according to the previously reported procedure[20]) (35 mg, 0.035 mmol) was added in one portion. The ice bath was removed, and the reaction was allowed to warm up to 20 °C and was stirred for 15 h. The reaction mixture was concentrated under reduced pressure, and the residue was purified by preparative TLC (CH_2Cl_2:MeOH = 95:5) and triturated with pentane to obtain **S3** as a colourless solid (29 mg, 0.024 mmol, 69% yield). ^{1}H NMR (400 MHz, Methylene Chloride-d_2) δ = 8.66 (s, 1H), 7.90 (t, *J* = 6.1 Hz, 1H), 7.44 – 7.38 (m, 2H), 7.35 – 7.29 (m, 7H), 7.22 (d, *J* = 9.1 Hz, 1H), 5.26 (br s, 1H), 4.84 (t, *J* = 7.9 Hz, 1H), 4.59 (dd, *J* = 7.7, 6.2 Hz, 1H), 4.56 – 4.48 (m, 2H), 4.37 (t, *J* = 6.5 Hz, 2H), 4.32 – 4.21 (m, 2H), 4.11 – 3.99 (m, 2H), 3.87 (dd, *J* = 11.9, 4.3 Hz, 1H), 3.73 – 3.43 (m, 13H), 3.42 – 3.25 (m, 3H), 2.96 (t, *J* = 6.5 Hz, 2H), 2.87 (t, *J* = 6.7 Hz, 2H), 2.61 – 2.49 (m, 4H), 2.47 (s, 3H), 2.41 – 2.31 (m, 4H), 1.67 (s, 3H), 0.98 (s, 9H); ^{13}C NMR (101 MHz, Methylene Chloride-d_2) δ = 171.2, 171.1, 170.8, 170.3, 164.1, 156.2, 154.6, 150.5, 150.4, 148.9, 139.0, 137.3, 136.8, 132.6, 132.0, 131.4, 131.2, 131.1, 130.9, 130.4 (2C), 129.6 (2C), 128.9 (2C), 128.2 (2C), 77.3, 71.3, 71.0, 70.9 (2C), 70.6, 70.4, 66.2, 59.1, 57.1, 54.7, 54.1, 50.3, 43.3, 40.0, 39.1, 38.0, 37.4, 35.6, 34.2, 26.5 (3C), 16.3, 14.6, 13.2, 12.0; HRMS (ESI), m/z: $[M+2H]^{2+}$ calcd for $C_{54}H_{69}O_{10}N_{12}ClS_4^{2+}$: 604.1910 found: 604.1901.

Compound S4 – *6-Azidohexyl((3R,5S)-1-((S)-2-(tert-butyl)-17-((S)-4-(4-chlorophenyl)-2,3,9-trimethyl-6H-thieno[3,2-f][1,2,4]triazolo[4,3-a][1,4]diazepin-6-yl)-4,16-dioxo-6,9,12-trioxa-3,15-diazaheptadecanoyl)-5-((4-(4-methylthiazol-5-yl)benzyl)carbamoyl)pyrrolidin-3-yl) carbonate*

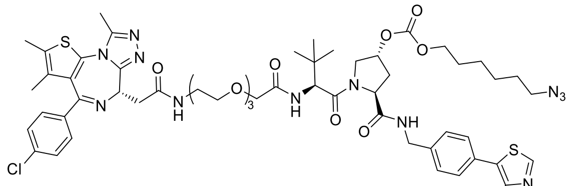

In an oven-dried flask, triphosgene (7.25 mg, 0.024 mmol) was dissolved in anhydrous CH_2Cl_2 (1.6 mL) and cooled to 0 °C. While stirring, dropwise, an ice-cold solution of **S2**[21] (10 mg, 0.070 mmol) and DMAP (25.6 mg, 0.209 mmol) in anhydrous CH_2Cl_2 (1.6 mL) was added over 5 min. The ice bath was removed, and the reaction was allowed to warm up to 20 °C and stirred for 1 h. The solution was cooled to 0 °C and **MZ1**[20] (35 mg, 0.035 mmol) was added in one portion. Again, the ice bath was removed, and the reaction was allowed to warm up to 20 °C and stirred for 15 h. The reaction mixture was concentrated under reduced pressure, and the residue was purified by flash column chromatography (CH_2Cl_2:MeOH = 9:3 to 85:15) and triturated with pentane to obtain **S4** as a colourless solid (22 mg, 0.188 mmol, 54% yield). ^{1}H NMR (500 MHz, Methylene Chloride-*d*$_2$) δ = 8.66 (s, 1H), 7.81 (t, *J* = 6.1 Hz, 1H), 7.44 – 7.39 (m, 2H), 7.36 – 7.29 (m, 6H), 7.24 (p, *J* = 5.6 Hz, 2H), 5.26 (dp, *J* = 4.9, 2.3 Hz, 1H), 4.79 (t, *J* = 7.8 Hz, 1H), 4.59 (dd, *J* = 7.6, 6.4 Hz, 1H), 4.56 – 4.48 (m, 2H), 4.27 (dd, *J* = 15.2, 5.5 Hz, 1H), 4.16 (dt, *J* = 12.0, 1.9 Hz, 1H), 4.11 (td, *J* = 6.7, 1.1 Hz, 2H), 4.08 – 3.97 (m, 2H), 3.88 (dd, *J* = 11.8, 4.5 Hz, 1H), 3.74 – 3.58 (m, 8H), 3.58 – 3.50 (m, 2H), 3.50 – 3.42 (m, 1H), 3.42 – 3.28 (m, 3H), 3.25 (t, *J* = 6.9 Hz, 2H), 2.58 (s, 3H), 2.55 – 2.46 (m, 4H), 2.39 (s, 3H), 2.36 – 2.29 (m, 1H), 1.67 (s, 3H), 1.61 – 1.55 (m, 2H), 1.44 – 1.33 (m, 4H), 0.98 (s, 9H), (Note: two signals missing due to overlapping with water); ^{13}C NMR (126 MHz, Methylene Chloride-*d*$_2$) δ = 171.2, 171.1, 170.8, 170.1, 164.1, 156.2, 154.9, 150.5, 150.4, 148.9, 139.0, 137.2, 136.8, 132.6, 132.0, 131.4, 131.2, 131.1, 130.9, 130.4 (2C), 129.6 (2C), 128.9 (2C), 128.2 (2C), 76.8, 71.4, 71.0, 70.9, 70.8, 70.5, 70.3, 68.6, 59.1, 57.0, 54.7, 54.1, 51.8, 43.3, 39.9, 39.0, 35.8, 34.3, 29.1, 28.8, 26.7, 26.5 (3C), 25.7, 16.3, 14.6, 13.2, 12.0; HRMS (ESI), m/z: $[M+H]^+$ calcd for $C_{56}H_{72}O_{10}N_{12}ClS_2^+$: 1171.4619 found: 1171.4618.

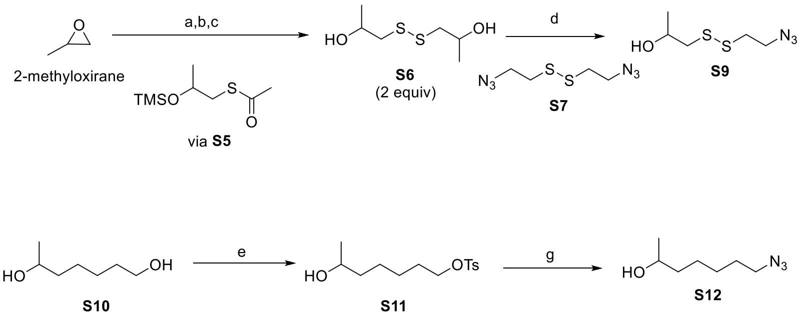

Scheme S6 Synthetic route to obtain the secondary alcohol linkers **S9** and **S12**. a) Thiolacetic acid (1.1 equiv), SiO_2, 25 °C, 18 h; b) HMDS (1 equiv), 25 °C, 2 h; c) NH_3 (10 equiv), MeOH (0.2 M), air, 0 to 20 °C, 72 h, 45% over three steps; d) **S7** (1 equiv), DTT (0.4 equiv), CH_2Cl_2/PBS (1:1; 0.05 M), pH 8, 20 °C, 48 h, 60%; e) TsCl (1.3 equiv), Et_3N (1.5 equiv), DMF (0.15 M), 0 °C, 2 h, 67%; g) NaN_3 (1 equiv), DMF (0.1 M), 60 °C, 18 h, 87%.

Compound S5 – *S-(2-((Trimethylsilyl)oxy)propyl) ethanethioate*

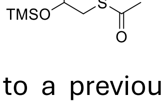

Compound **S5** was prepared according to a previously reported procedure starting with 2-metyloxirane (0.58 g, 10 mmol)).[22] The crude product (1.25 g, ~6.06 mmol) was used in the next

step without further purification. ^{1}H NMR of the crude product was in correlation with the literature[22].

Compound S6 *– 1,1'-Disulfanediylbis(propan-2-ol)*

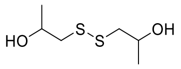

Crude material of **S5** (731 mg, ~3.54 mmol) was dissolved in MeOH (10 mL) and the solution was cooled to 0 °C. NH_3 (7M in MeOH; 5.06 mL, 35.4 mmol) was added dropwise. The reaction was fitted with a balloon of air and allowed to slowly warm up to 20 °C and stirred for 72 h. The reaction mixture was bubbled through with N_2 for 15 min and the resulting solution was concentrated under reduced pressure. The crude material was purified by flash column chromatography (CH_2Cl_2:MeOH = 995:5 to 965:35) to afford the desired product **S6** as a colourless oil (239 mg, 1.31 mmol, 45% yield over three steps). Spectral data match with previously published data.[23] HRMS (ESI), m/z: $[M+Na]^+$ calcd for $C_6H_{14}O_2NaS_2^+$: 205.0327 found: 205.0328.

Compound S9 *– 1-((2-Azidoethyl)disulfaneyl)propan-2-ol*

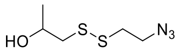

S6 (250 mg, 1.37 mmol) and **S7**[24] (140 mg, 0.686 mmol) were dissolved in CH_2Cl_2 (16.5 mL). Phosphate buffer (16.5 mL) was added, and the pH was adjusted to 8 with NaOH (1 M). 1,4-Dithiothreitol (DTT) (21.2 mg, 0.137 mmol) was added in one portion, and the flask was tightly sealed. The reaction mixture was vigorously stirred at 20 °C for 24 h. The aqueous layer was adjusted to pH 8 and a supplement of additional DTT (21.2 mg, 0.137 mmol) was added to the reaction mixture, which was vigorously stirred for another 24 h. The reaction mixture was diluted with brine and extracted with CH_2Cl_2. The organic layers were combined, dried over $MgSO_4$ and concentrated under reduced pressure. The crude material was purified by flash column chromatography (CH_2Cl_2:Et_2O:MeOH = 100:0:0 to 50:50:0 to 0:97:3) to afford **S9** as a pale-yellow oil (160 mg, 0.828 mmol, 60% yield). ^{1}H NMR (400 MHz, Methylene Chloride-d_2) δ = 4.07 – 3.99 (m, 1H), 3.60 (t, J = 6.7 Hz, 2H), 2.89 (m, 3H), 2.69 (dd, J = 13.7, 8.1 Hz, 1H), 2.16 (d, J = 3.4 Hz, 1H), 1.25 (d, J = 6.3 Hz, 3H); ^{13}C NMR (101 MHz, Methylene Chloride-d_2) δ = 66.3, 50.4, 48.5, 37.9, 22.3; HRMS (ESI), m/z: $[M+Na]^+$ calcd for $C_5H_{11}ON_3NaS_2^+$: 216.0236 found: 216.0237.

Compound S11 *– 6-Hydroxyheptyl 4-methylbenzenesulfonate*

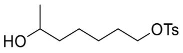

In an oven-dried flask, **S10**[25] (160 mg, 1.21 mmol) was dissolved in anhydrous CH_2Cl_2 (8 mL). Dry triethylamine (183 mg, 0.25 mL, 1.81 mmol) was added, and the solution was cooled down to 0 °C. TsCl (300 mg, 1.57 mmol) was added portion-wise. The reaction mixture was stirred at 0 °C for 2 h, after which it was quenched by the addition of $NaHCO_3$ (sat.) until pH ~8-9 and then diluted with brine. The phases were separated, and the aqueous layer was extracted with CH_2Cl_2. The combined organic layers were washed with brine, dried over $MgSO_4$ and concentrated under reduced pressure. The crude material was purified by flash column chromatography (CH_2Cl_2:EtOAc = 99:1 to 90:10) to afford the desired product **S11** as a colourless oil (231 mg, 0.807 mmol, 67%). Spectral data match with previously published data.[26]

Compound S12 *– 7-Azidoheptan-2-ol*

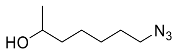

In a teflon flask, **S11** (209 mg, 0.73 mmol) was dissolved in anhydrous *N,N*-dimethylformamide (DMF) (7 mL). NaN_3 (47.5 mg, 0.73 mmol) was added, in one portion. The flask was sealed, and the reaction mixture was heated to 60 °C and stirred for 20 h. The reaction mixture was cooled to 0 °C and quenched with $NaHCO_3$ (sat.) until pH ~8-9. It was diluted with brine and extracted with

CH_2Cl_2. The combined organic layers were washed with ice-water, dried over $MgSO_4$ and concentrated under reduced pressure. The crude material was purified by flash column chromatography (pentane:CH_2Cl_2:Et_2O = 40:60:0 to 0:100:0 to 0:85:15) to afford the desired product **S12** as a colourless oil (100 mg, 0.636, 87% yield. ^{1}H NMR (500 MHz, Chloroform-*d*) δ = 3.84 – 3.76 (m, 1H), 3.27 (t, *J* = 6.9 Hz, 2H), 1.62 (p, *J* = 7.0 Hz, 2H), 1.51 – 1.31 (m, 6H, overlapping with water), 1.19 (d, *J* = 6.1 Hz, 3H); ^{13}C NMR (126 MHz, Chloroform-*d*) δ = 68.1, 51.5, 39.2, 29.0, 26.9, 25.5, 23.7; HRMS (ESI), m/z: $[M+Na]^+$ calcd for $C_7H_{15}N_3NaO^+$: 180.1107 found: 180.1106.

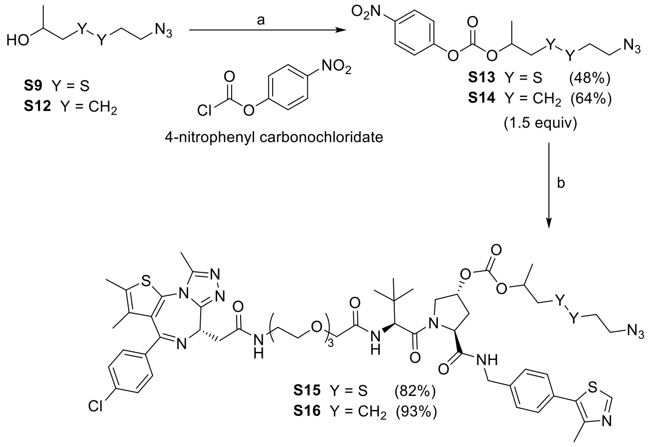

Scheme S7 Synthetic route to obtain the secondary carbonate **MZ1**-N_3 intermediates **S15** and **S16**. a) 4-Nitropenyl carbonochloridate (1.1 or 2 equiv), DMAP (1.1 or 2 equiv), THF or CH_2Cl_2 (0.1 M), 0 to 20 °C, 18 or 2 h, 48 & 64%; b) **MZ1** (1 equiv), DMAP (1.5 equiv), CH_2Cl_2 (0.1 or 0.05 M), 20 °C, 18 h, 82 & 93%.

Compound S13 *– 1-((2-Azidoethyl)disulfaneyl)propan-2-yl (4-nitrophenyl) carbonate*

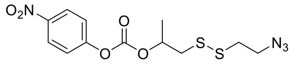

In an oven-dried flask, **S9** (90 mg, 0.466 mmol) and DMAP (62.6 mg, 0.512 mmol) were dissolved in anhydrous tetrahydrofurane (THF) (5 mL) and cooled to 0 °C and 4-nitropenyl carbonochloridate (103 mg, 0.512 mmol) was added in one portion. The reaction mixture was allowed to slowly warm up to 20 °C and stirred for 18 h. The reaction mixture was filtered through a plug of silica and washed with CH_2Cl_2. The filtrate was concentrated under reduced pressure, and the residue was purified by flash column chromatography (Hexane:EtOAc = 95:5 to 50:50) to afford the desired compound **S13** as a colourless waxy solid (80 mg, 0.223 mmol, 48% yield). ^{1}H NMR (500 MHz, Chloroform-*d*) δ = 8.32 – 8.25 (m, 2H), 7.43 – 7.37 (m, 2H), 5.19 – 5.09 (m, 1H), 3.60 (t, *J* = 6.8 Hz, 2H), 3.05 (dd, *J* = 14.1, 6.9 Hz, 1H), 2.99 – 2.85 (m, 3H), 1.50 (d, *J* = 6.3 Hz, 3H); ^{13}C NMR (126 MHz, Chloroform-*d*) δ = 155.6, 152.0, 145.6, 125.5 (2C), 121.9 (2C), 75.2, 50.1, 44.4, 37.7, 19.2; HRMS (ESI), m/z: $[M+Na]^+$ calcd for $C_{12}H_{14}O_5N_4NaS_2^+$: 381.0298 found: 381.0299.

Compound S14 *– 7-Azidoheptan-2-yl (4-nitrophenyl) carbonate*

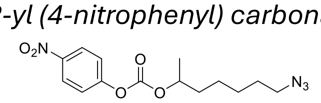

In an oven-dried flask, **S12** (62.9 mg, 0.4 mmol) and DMAP (97.7 mg, 0.8 mmol) were dissolved in anhydrous CH_2Cl_2 (4 mL) and cooled to 0 °C and 4-nitropenyl carbonochloridate (161 mg, 0.8 mmol) was added in one portion. The reaction mixture was allowed to warm up to 20 °C and stirred for 2 h. The reaction mixture was quenched by the addition of brine and diluted with CH_2Cl_2. The layers were separated, and the aqueous layer was extracted with CH_2Cl_2. The organic layers were combined and washed with brine, dried over $MgSO_4$ and concentrated under reduced pressure. The crude material was purified by flash column chromatography (cyclohexane:CH_2Cl_2

= 100:0 to 0:100) to afford the desired compound **S14** as a colourless oil (83 mg, 0.258 mmol, 64% yield). ^{1}H NMR (400 MHz, Chloroform-*d*) δ = 8.31 – 8.25 (m, 2H), 7.41 – 7.36 (m, 2H), 4.93 – 4.84 (m, 1H), 3.28 (t, *J* = 6.8 Hz, 2H), 1.83 – 1.71 (m, 1H), 1.69 – 1.58 (m, 3H), 1.51 – 1.40 (m, 4H), 1.39 (d, *J* = 6.2 Hz, 3H); ^{13}C NMR (101 MHz, Chloroform-*d*) δ = 155.8, 152.3, 145.5, 125.4 (2C), 121.9 (2C), 77.5, 51.4, 35.7, 28.9, 26.7, 25.0, 19.9; HRMS (ESI), m/z: $[M+Na]^+$ calcd for $C_{14}H_{18}O_5N_4Na^+$: 345.1169 found: 345.1170.

Compound S15 – *1-((2-Azidoethyl)disulfaneyl)propan-2-yl((3R,5S)-1-((S)-2-(tert-butyl)-17-((S)-4-(4-chlorophenyl)-2,3,9-trimethyl-6H-thieno[3,2-f][1,2,4]triazolo[4,3-a][1,4]diazepin-6-yl)-4,16-dioxo-6,9,12-trioxa-3,15-diazaheptadecanoyl)-5-((4-(4-methylthiazol-5-yl)benzyl)carbamoyl)pyrrolidin-3-yl) carbonate*

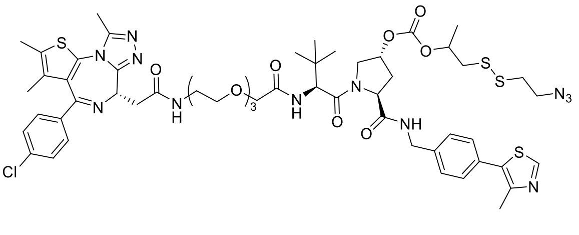

In an oven-dried flask, **S13** (21.4 mg, 0.06 mmol) was dissolved in anhydrous CH_2Cl_2 (0.4 mL). DMAP (7.31 mg, 0.06 mmol) and **MZ1**[20] (40 mg, 0.04 mmol) were added in one portion. The reaction mixture was stirred at 20 °C for 18 h, after which it was concentrated under reduced pressure. The residue was purified by flash column chromatography (CH_2Cl_2:MeOH = 95:5 to 92:8) and triturated with pentane to afford **S15** as a colourless solid (40 mg, 0.033 mmol, 82% yield). ^{1}H NMR (500 MHz, Chloroform-*d*) δ = 8.67 (s, 1H), 7.89 (dt, *J* = 11.6, 6.0 Hz, 1H), 7.44 – 7.37 (m, 2H), 7.34 – 7.27 (m, 6H), 7.25 – 7.16 (m, 2H), 5.35 – 5.29 (m, 1H), 5.01 (hept, *J* = 6.2 Hz, 1H), 4.88 (dt, *J* = 12.6, 7.7 Hz, 1H), 4.63 (t, *J* = 6.9 Hz, 1H), 4.56 (dd, *J* = 9.3, 5.9 Hz, 1H), 4.49 (ddd, *J* = 15.1, 6.3, 3.5 Hz, 1H), 4.35 (ddd, *J* = 15.0, 5.7, 2.9 Hz, 1H), 4.28 – 4.20 (m, 1H), 4.14 – 3.99 (m, 2H), 3.91 (dt, *J* = 11.8, 4.3 Hz, 1H), 3.76 – 3.59 (m, 8H), 3.62 – 3.49 (m, 5H), 3.49 – 3.32 (m, 3H), 3.09 – 2.76 (m, 4H), 2.69 – 2.55 (m, 4H), 2.50 (s, 3H), 2.42 – 2.32 (m, 4H), 1.67 (s, 3H), 1.39 (dd, *J* = 6.3, 5.1 Hz, 3H), 0.98 (s, 9H); ^{13}C NMR (126 MHz, Chloroform-*d*) δ = 170.9, 170.9, 170.9, 170.9, 170.1, 170.0, 163.7, 163.7, 155.9, 155.9, 154.0, 153.9, 150.4, 149.9, 148.6, 138.4, 138.4, 136.8, 136.8, 136.8, 136.8, 132.1, 131.8, 131.1, 130.9, 130.9, 130.8, 130.0 (2C), 129.5 (2C), 128.8 (2C), 128.2 (2C), 76.7, 76.7, 74.0, 73.8, 71.1, 71.1, 70.9, 70.8, 70.8, 70.7, 70.7, 70.4, 70.2, 70.2, 58.8, 56.9, 56.8, 54.4, 53.9, 53.9, 50.1, 44.7, 44.2, 43.3, 43.3, 39.8, 38.9, 37.6, 37.5, 35.6, 35.5, 33.8, 33.8, 26.5 (3C), 19.2, 19.0, 16.2, 14.6, 13.2, 11.9, (Note: due to the presence of two diastereomers, several peaks are duplicated, but not all); HRMS (ESI), m/z: $[M+H]^+$ calcd for $C_{55}H_{70}O_{10}N_{12}ClS_4^+$: 1221.3904 found: 1221.3901.

Compound S16 – *7-Azidoheptan-2-yl ((3R,5S)-1-((S)-2-(tert-butyl)-17-((S)-4-(4-chlorophenyl)-2,3,9-trimethyl-6H-thieno[3,2-f][1,2,4]triazolo[4,3-a][1,4]diazepin-6-yl)-4,16-dioxo-6,9,12-trioxa-3,15-diazaheptadecanoyl)-5-((4-(4-methylthiazol-5-yl)benzyl)carbamoyl)pyrrolidin-3-yl) carbonate*

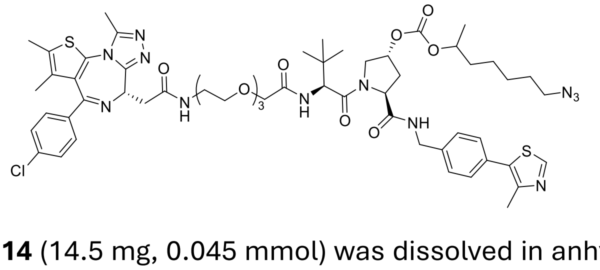

In an oven-dried flask, **S14** (14.5 mg, 0.045 mmol) was dissolved in anhydrous CH_2Cl_2 (0.6 mL). DMAP (5.48 mg, 0.045 mmol) and **MZ1**[20] (30 mg, 0.03 mmol) were added in one portion. The reaction mixture was stirred at 20 °C for 18 h, after which it was concentrated under reduced pressure. The residue was purified by flash column chromatography (CH_2Cl_2:MeOH = 95:5 to

90:10) and by preparative TLC (CH_2Cl_2:MeOH = 95:5) and triturated with pentane to afford **S16** as a colourless solid (33 mg, 0.028 mmol, 93% yield). ^{1}H NMR (500 MHz, Methylene Chloride-d_2) δ = 8.66 (s, 1H), 7.78 (t, J = 6.0 Hz, 1H), 7.45 – 7.37 (m, 2H), 7.36 – 7.28 (m, 6H), 7.26 – 7.17 (m, 2H), 5.29 – 5.21 (m, 1H), 4.82 – 4.68 (m, 2H), 4.64 – 4.45 (m, 3H), 4.28 (dd, J = 15.2, 5.5 Hz, 1H), 4.14 (dd, J = 18.6, 11.7 Hz, 1H), 4.08 – 3.96 (m, 2H), 3.94 – 3.85 (m, 1H), 3.71 – 3.59 (m, 8H), 3.58 – 3.43 (m, 3H), 3.42 – 3.22 (m, 5H), 2.59 (s, 3H), 2.55 – 2.45 (m, 4H), 2.39 (s, 3H), 2.36 – 2.28 (m, 1H), 1.67 (s, 3H), 1.63 – 1.46 (m, 4H), 1.42 – 1.29 (m, 4H), 1.25 (t, J = 6.2 Hz, 3H), 0.97 (s, 9H); ^{13}C NMR (126 MHz, Methylene Chloride-d_2) δ = 171.3, 171.2, 171.1, 171.1, 170.8, 170.1, 170.0, 164.1, 156.2, 154.6, 154.5, 150.5, 150.4, 148.9, 139.0, 137.2, 136.8, 132.6, 132.0, 131.4, 131.2, 131.1, 130.8, 130.4 (2C), 129.6 (2C), 128.9 (2C), 128.2 (2C), 128.2 (2C), 76.6, 76.6, 76.1, 76.0, 71.4, 71.4, 71.1, 71.0, 70.9, 70.8, 70.8, 70.6, 70.5, 70.3, 70.3, 59.2, 57.0, 57.0, 54.7, 54.2, 54.1, 51.8, 51.8, 43.3, 43.3, 39.9, 39.1, 36.0, 36.0, 35.8, 35.8, 34.4, 34.3, 29.1, 27.0, 26.9, 26.5 (3C), 25.2, 25.2, 19.9, 19.9, 16.3, 14.6, 13.2, 12.0, (Note: due to the presence of two diastereomers, several peaks are duplicated, but not all); HRMS (ESI), m/z: $[M+2H]^{2+}$ calcd for $C_{57}H_{75}O_{10}N_{12}ClS_2^{2+}$: 593.2424 found: 593.2424.

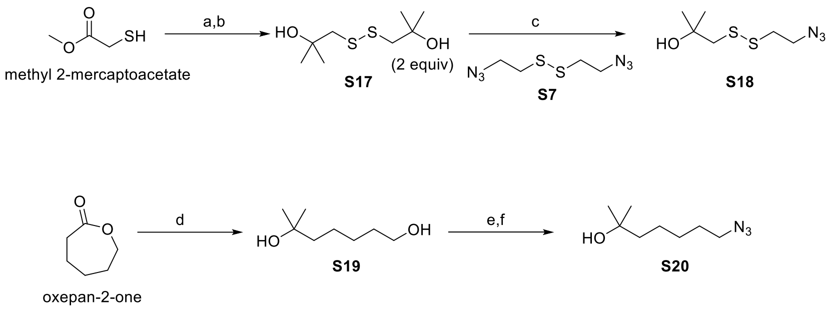

Scheme S8 Synthetic route to obtain the tertiary alcohol linkers **S18** and **S20**. a) MeMgBr (3.5 equiv), Et_2O (0.3 M), 0 to 20 °C, 45 min; b) I_2 (1.1 equiv), Et_3N (1.1 equiv), THF (0.8 M), 0 to 20 °C, 1 h, 53% over two steps; c) **S7** (1 equiv), DTT (0.3 equiv), CH_2Cl_2/PBS (1:1; 0.05 M), pH 8, 20 °C, 24 h, 26%; d) MeMgBr (3.5 equiv), Et_2O (0.4 M), 0 to 20 °C, 45 min, 41%; e) MsCl (2 equiv), Et_3N (2 equiv), CH_2Cl_2 (0.5 M), 0 to 20 °C, 24 h; f) NaN_3 (1 equiv), DMF (0.1), 60 °C, 18 h, 69% over two steps.

***Compound S17** – 1,1'-Disulfanediylbis(2-methylpropan-2-ol)*

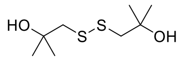

In an oven-dried flask, MeMgBr (3M in Et_2O; 46.7 mL, 140 mmol) was cooled to 0 °C. A solution of methyl 2-mercaptoacetate (4.25 g, 3.6 mL, 40 mmol) in anhydrous Et_2O (53 mL) was added dropwise over 15 min. The reaction was allowed to warm up to 20 °C and stirred for 30 min. The reaction mixture was cooled to 0 °C and quenched slowly with ice-water. Then NH_4Cl (sat.) was added, and the mixture was neutralised with HCl (32%). The phases were separated, and the aqueous layer was extracted with Et_2O. The combined organic layers were dried over $MgSO_4$ and concentrated under reduced pressure, to provide a crude colourless oil (3.92 g, ~36.9 mmol) which was used in the next step without further purification. In an oven-dried flask, the oil (1.96 g, ~18.5 mmol) was dissolved in anhydrous THF (45 mL). Dry triethylamine (2.05 g, 2.86 mL, 20.3 mmol) was added, and the solution was cooled down to 0 °C. Iodine (5.17 g, 20.3 mmol) was added to the reaction mixture portion-wise. The reaction mixture was allowed to warm up to 20 °C and stirred for 1 h, after which the reaction was quenched with $Na_2S_2O_3$ (sat.) and extracted with CH_2Cl_2. The combined organic layers were washed with brine, dried over $MgSO_4$ and concentrated under reduced pressure. The crude material was purified by flash column chromatography (hexane:EtOAc = 3:1 to 1:1) to afford the desired product **S17** as a colourless oil (2.26 g, 10.8 mmol, 53% yield over two steps). ^{1}H NMR (400 MHz, Chloroform-d) δ = 3.01 (s, 4H),

2.16 (s, 2H), 1.31 (s, 12H); ^{13}C NMR (101 MHz, Chloroform-*d*) δ = 71.2 (2C), 54.3 (2C), 28.9 (4C); HRMS (ESI), m/z: [M+Na]$^+$ calcd for $C_8H_{18}O_2NaS_2^+$: 233.0645 found: 233.0643.

Compound S18 *– 1-((2-Azidoethyl)disulfaneyl)-2-methylpropan-2-ol*

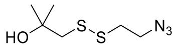

S17 (316 mg, 1.5 mmol) and **S7**[24] (153 mg, 0.75 mmol) were dissolved in CH_2Cl_2 (7.5 mL). Phosphate buffered saline (PBS) (7.5 mL) was added to the solution, and the pH was adjusted to ~8 with $NaHCO_3$ (sat.). DTT (34.7 mg, 0.225 mmol) was added in one portion and the flask was tightly sealed and stirred at 20 °C for 24 h and stored at 4 °C for 2 days. The reaction mixture was diluted with CH_2Cl_2 and brine, and the aqueous phase was saturated with solid NaCl. The phases were separated, and the aqueous layer was extracted with CH_2Cl_2. The organic layers were combined, dried over $MgSO_4$ and concentrated under reduced pressure. The crude material was purified by flash column chromatography (CH_2Cl_2:Et_2O = 100:0 to 50:50) to afford the desired product **S18** as a brown oil (80 mg, 0.386 mmol, 26% yield). ^{1}H NMR (400 MHz, Chloroform-*d*) δ = 3.61 (t, *J* = 6.8 Hz, 2H), 2.97 (s, 2H), 2.90 (t, *J* = 6.8 Hz, 2H), 1.96 (s, 1H), 1.32 (s, 6H); ^{13}C NMR (101 MHz, Chloroform-*d*) δ = 71.1, 54.7, 50.1, 37.7, 28.8 (2C); HRMS (ESI), m/z: [M+Na]$^+$ calcd for $C_6H_{13}ON_3NaS_2^+$: 230.0392 found: 230.0393.

Compound S19 *– 6-Methylheptane-1,6-diol*

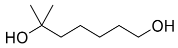

In a flamed-dried flask, MeMgBr (3M in Et_2O; 40.9 mL, 123 mmol) was cooled to 0 °C. A solution of the oxepan-2-one (4.0 g, 35 mmol) in anhydrous Et_2O (47 mL) was added dropwise over 10 min. The reaction was allowed to warm up to 20 °C and stirred for 45 min. The reaction mixture was cooled to 0 °C and quenched slowly with ice-water and then diluted with NH_4Cl (sat.). The mixture was neutralised with HCl (32%). The phases were separated, and the aqueous layer was extracted with Et_2O. The combined organic layers were dried over $MgSO_4$ and concentrated under reduced pressure. The crude material was purified by flash column chromatography (CH_2Cl_2:MeOH = 100:0 to 90:10) to afford the desired product **S19** as a colourless oil (2.1 g, 14.4 mmol, 41% yield). Spectral data match with previously published data.[27]

Compound S20 *– 7-Azido-2-methylheptan-2-ol*

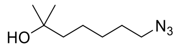

In an oven-dried flask, **S19** (1.9 g, 13 mmol) was dissolved in anhydrous CH_2Cl_2 (26 mL). The solution was cooled to 0 °C, and dry triethylamine (1.97 g, 2.7 mL, 19.5 mmol) was added in one portion, followed by a dropwise addition of MsCl (1.79 g, 1.21 mL, 15.6 mmol). The solution was allowed to slowly warm up to 20 °C, and it was stirred for 23 h. The reaction mixture was supplemented with additional dry triethylamine (0.66 g, 0.90 mL, 6.5 mmol) and MsCl (1.19 g, 0.81 mL, 10.4 mmol) and stirred for an additional 1 h at 20 °C. The reaction mixture was quenched with a dropwise addition of $NaHCO_3$ (sat.) until pH 8-9. The biphasic mixture was diluted with brine and extracted with CH_2Cl_2. The combined organic phases were washed with brine, dried over $MgSO_4$ and concentrated under reduced pressure, to provide the crude intermediate as a colourless oil (2.91 g, ~13 mmol), which was used in the next step without further purification. In a teflon flask, the intermediate oil (1.46 g, ~6.5 mmol) was dissolved in anhydrous DMF (65 mL), and NaN_3 (0.423 g, 6.5 mmol) was added in one portion. The reaction vessel was sealed, heated to 60 °C and stirred for 18 h. The reaction mixture was then allowed to cool down to 20 °C and quenched with $NaHCO_3$ (sat.) until pH 8-9. The solution was extracted with Et_2O, the organic layers were combined and washed with ice-cold water and brine. The organic layers were dried over $MgSO_4$ and concentrated under reduced pressure. The crude material was purified by flash column chromatography (hexane:EtOAc = 4:1 to 1:1) to afford the desired product **S20** as a colourless oil (770 mg, 4.50 mmol, 69% yield over two steps). ^{1}H NMR (500 MHz, Chloroform-*d*)

δ = 3.27 (t, *J* = 6.9 Hz, 2H), 1.66 – 1.58 (m, 2H), 1.50 – 1.36 (m, 6H; overlapping with water signal), 1.22 (s, 6H); ^{13}C NMR (126 MHz, Chloroform-*d*) δ = 71.1, 51.6, 43.9, 29.4 (2C), 29.0, 27.4, 24.0; HRMS (ESI), m/z: [M+Na]$^+$ calcd for $C_8H_{17}ON_3Na^+$: 194.1264 found: 194.1263.

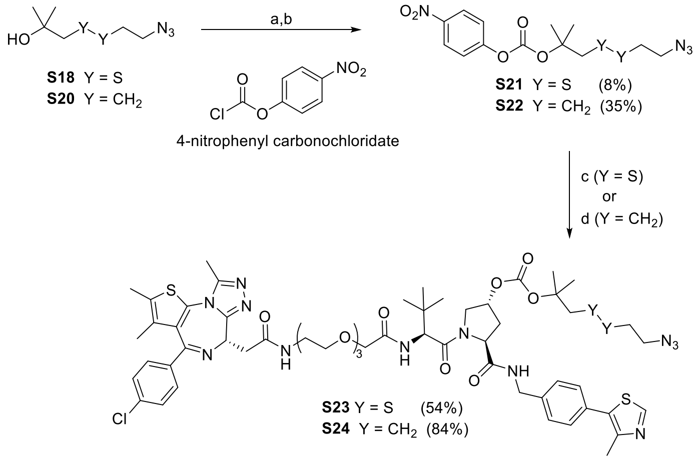

Scheme S9 Synthetic route to obtain the tertiary carbonate **MZ1**-N_3 intermediates **S23** and **S24**. a) NaHMDS (1 equiv), THF (0.1 M), 0 °C, 15 min; b) 4-Nitrophenyl carbonochloridate (1.5 equiv), DMAP (0.1 equiv), THF (0.05 M), 0 to 20 °C, 2 h, 8 & 35% over two steps; c) **S21** (1 equiv), **MZ1** (1.3 equiv), DMAP (2 equiv), CH_2Cl_2 (0.05 M), 20 °C, 48 h, 54%; d) **S22** (2.2 equiv), **MZ1** (1 equiv), DMAP (3 equiv), CH_2Cl_2 (0.1 M), 20 °C, 72 h, 84%.

Compound S21 *– 1-((2-Azidoethyl)disulfaneyl)-2-methylpropan-2-yl (4-nitrophenyl) carbonate*

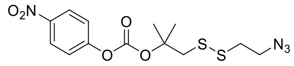

In an oven-dried flask, **S18** (50 mg, 0.241 mmol) was dissolved in anhydrous THF (2.4 mL) and cooled to 0 °C. NaHMDS (1 M in THF; 0.241 ml, 0.241 mmol) was added dropwise to the solution, and the solution was stirred at 0 °C for 15 min. It was added to a freshly prepared solution of 4-nitropenyl carbonochloridate (72.9 mg, 0.362 mmol) and DMAP (2.95 mg, 0.024 mmol) in anhydrous THF (2.4 mL) at 0 °C. The reaction mixture was allowed to slowly warm up to 20 °C and was stirred for 2 h, after which it was quenched by the addition of brine. The aqueous solution was extracted with CH_2Cl_2 and the combined organic layers were washed with brine, dried over $MgSO_4$ and concentrated under reduced pressure. The crude material was purified by flash column chromatography (cyclohexane:CH_2Cl_2 = 100:0 to 0:100) to afford the desired product **S21** as a colourless oil (7 mg, 0.019 mmol, 8% yield). ^{1}H NMR (400 MHz, Chloroform-*d*) δ = 8.31 – 8.23 (m, 2H), 7.40 – 7.34 (m, 2H), 3.60 (t, *J* = 6.8 Hz, 2H), 3.33 (s, 2H), 2.90 (t, *J* = 6.8 Hz, 2H), 1.64 (s, 6H); ^{13}C NMR (101 MHz, Chloroform-*d*) δ = 155.6, 150.5, 145.5, 125.4 (2C), 122.1 (2C), 85.3, 50.0, 49.4, 37.7, 25.6 (2C); HRMS (ESI), m/z: [M]$^+$ calcd for $C_{13}H_{16}O_5N_4S_2^+$: 372.0557 found: 372.0560.

Compound S22 *– 7-Azido-2-methylheptan-2-yl (4-nitrophenyl) carbonate*

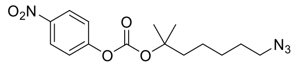

In an oven-dried flask, **S20** (85.6 mg, 0.5 mmol) was dissolved in anhydrous THF (5 mL) and cooled to 0 °C. NaHMDS (1 M in THF; 0.5 ml, 0.5 mmol) was added dropwise to the solution, and the solution was stirred at 0 °C for 15 min. It was added to a freshly prepared solution of 4-nitropenyl carbonochloridate (151 mg, 0.75 mmol) and DMAP (6.11 mg, 0.05 mmol) in anhydrous THF (5 mL) at 0 °C. The reaction mixture was allowed to slowly warm up to 20 °C and was stirred for 2 h, after which it was quenched by the addition of brine. The aqueous solution was extracted with CH_2Cl_2 , and the combined organic layers were washed with brine, dried over $MgSO_4$ and concentrated under reduced pressure. The crude material was purified by flash column

chromatography (cyclohexane:CH_2Cl_2 = 100:0 to 0:100) to afford the desired product **S22** as a colourless oil (58 mg, 0.172 mmol, 35% yield). ^{1}H NMR (500 MHz, Chloroform-*d*) δ = 8.29 – 8.24 (m, 2H), 7.38 – 7.33 (m, 2H), 3.28 (t, *J* = 6.8 Hz, 2H), 1.87 – 1.81 (m, 2H), 1.68 – 1.59 (m, 2H), 1.55 (s, 6H), 1.47 – 1.38 (m, 4H); ^{13}C NMR (126 MHz, Chloroform-*d*) δ = 155.8, 150.6, 145.3, 125.4 (2C), 122.0 (2C), 87.0, 51.5, 40.5, 28.9, 27.1, 25.7 (2C), 23.6; HRMS (ESI), m/z: [M+Na]$^+$ calcd for $C_{15}H_{20}O_5N_4Na^+$: 359.1326 found: 359.1327.

Compound S23 *– 1-((2-Azidoethyl)disulfaneyl)-2-methylpropan-2-yl ((3R,5S)-1-((S)-2-(tert-butyl)-17-((S)-4-(4-chlorophenyl)-2,3,9-trimethyl-6H-thieno[3,2-f][1,2,4]triazolo[4,3-a][1,4]diazepin-6-yl)-4,16-dioxo-6,9,12-trioxa-3,15-diazaheptadecanoyl)-5-((4-(4-methylthiazol-5-yl)benzyl)carbamoyl)pyrrolidin-3-yl) carbonate*

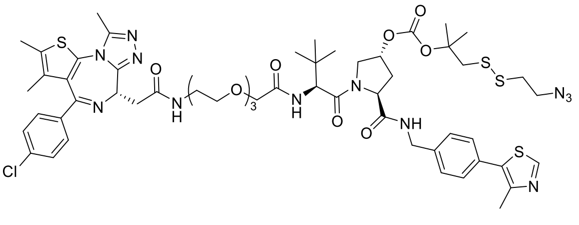

In an oven-dried flask, **S21** (7.2 mg, 0.019 mmol) was dissolved in anhydrous CH_2Cl_2 (0.4 mL). DMAP (4.7 mg, 0.039 mmol) and **MZ1**[20] (25.2 mg, 0.025 mmol) were added in one portion. The reaction mixture was stirred at 20 °C for 48 h. The reaction mixture was directly loaded on silica and purified by flash column chromatography (CH_2Cl_2:MeOH = 95:5 to 90:10) and by preparative TLC (CH_2Cl_2:MeOH =95:5) and triturated with pentane to afford **S23** as a colourless solid (13 mg, 0.0105 mmol, 54% yield). ^{1}H NMR (400 MHz, Methylene Chloride-d_2) δ = 8.66 (s, 1H), 7.85 (t, *J* = 6.1 Hz, 1H), 7.44 – 7.38 (m, 2H), 7.36 – 7.29 (m, 6H), 7.29 – 7.20 (m, 2H), 5.26 – 5.19 (m, 1H), 4.79 (t, *J* = 7.8 Hz, 1H), 4.64 – 4.46 (m, 3H), 4.27 (dd, *J* = 15.3, 5.4 Hz, 1H), 4.13 (d, *J* = 11.8 Hz, 1H), 4.04 (d, *J* = 1.7 Hz, 2H), 3.89 (dd, *J* = 11.7, 4.5 Hz, 1H), 3.71 – 3.62 (m, 8H), 3.61 – 3.52 (m, 4H), 3.52 – 3.44 (m, 1H), 3.43 – 3.24 (m, 4H), 3.20 (d, *J* = 13.9 Hz, 1H), 2.87 (t, *J* = 6.7 Hz, 2H), 2.58 (s, 3H), 2.53 – 2.43 (m, 4H), 2.39 (s, 3H), 2.36 – 2.27 (m, 1H), 1.67 (s, 3H), 1.54 (s, 3H), 1.52 (s, 3H), 0.98 (s, 9H); ^{13}C NMR (101 MHz, Methylene Chloride-d_2) δ = 171.2, 171.1, 170.8, 170.1, 164.1, 156.2, 152.9, 150.5, 150.4, 148.8, 139.0, 137.3, 136.8, 132.6, 132.0, 131.4, 131.2, 131.1, 130.9, 130.4 (2C), 129.7 (2C), 128.9 (2C), 128.3 (2C), 83.7, 76.4, 71.4, 71.1, 70.9, 70.8, 70.6, 70.3, 59.2, 57.0, 54.7, 50.3, 50.2, 43.3, 40.0, 39.1, 37.9, 35.8, 34.4, 26.5 (3C), 25.7, 25.6, 16.3, 14.6, 13.2, 12.0, (Note: one signal missing due to overlapping with the solvent); HRMS (ESI), m/z: [M+H]$^+$ calcd for $C_{56}H_{72}O_{10}N_{12}ClS_4^+$: 1235.4060 found: 1235.4073.

Compound S24 *– 7-Azido-2-methylheptan-2-yl ((3R,5S)-1-((S)-2-(tert-butyl)-17-((S)-4-(4-chlorophenyl)-2,3,9-trimethyl-6H-thieno[3,2-f][1,2,4]triazolo[4,3-a][1,4]diazepin-6-yl)-4,16-dioxo-6,9,12-trioxa-3,15-diazaheptadecanoyl)-5-((4-(4-methylthiazol-5-yl)benzyl)carbamoyl)pyrrolidin-3-yl) carbonate*

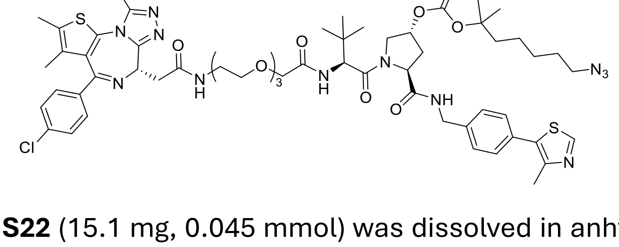

In an oven-dried flask, **S22** (15.1 mg, 0.045 mmol) was dissolved in anhydrous CH_2Cl_2 (0.3 mL). DMAP (5.48 mg, 0.045 mmol) and **MZ1**[20] (30 mg, 0.03 mmol) were added in one portion. The reaction mixture was stirred at 20 °C for 48 h, then supplemented with an additional **S22** (7.04 mg, 0.021 mmol) and DMAP (5.48 mg, 0.045 mmol) and stirred at 20 °C for an additional 24 h. The reaction mixture was directly loaded on silica and purified by flash column chromatography (CH_2Cl_2:MeOH = 95:5 to 90:10) and by preparative TLC (CH_2Cl_2:MeOH = 90:10) and triturated with

pentane to afford **S24** as a colourless solid (30 mg, 0.025 mmol, 84% yield); ^{1}H NMR (500 MHz, Methylene Chloride-d_2) δ = 8.66 (s, 1H), 7.77 (t, *J* = 6.2 Hz, 1H), 7.45 – 7.38 (m, 2H), 7.38 – 7.28 (m, 6H), 7.27 – 7.17 (m, 2H), 5.24 – 5.18 (m, 1H), 4.76 (t, *J* = 7.7 Hz, 1H), 4.62 – 4.54 (m, 2H), 4.51 (dd, *J* = 15.2, 6.6 Hz, 1H), 4.28 (dd, *J* = 15.3, 5.5 Hz, 1H), 4.11 – 3.99 (m, 3H), 3.89 (dd, *J* = 11.7, 4.7 Hz, 1H), 3.72 – 3.58 (m, 8H), 3.58 – 3.50 (m, 2H), 3.50 – 3.43 (m, 1H), 3.42 – 3.28 (m, 3H), 3.25 (t, *J* = 7.1 Hz, 2H), 2.58 (s, 3H), 2.54 – 2.43 (m, 4H), 2.39 (s, 3H), 2.33 – 2.24 (m, 1H), 1.67 (s, 3H), 1.63 – 1.55 (m, 2H), 1.44 (s, 3H), 1.42 (s, 3H), 1.40 – 1.30 (m, 4H), 0.98 (s, 9H), (Note: two protons overlapping with water); ^{13}C NMR (126 MHz, Methylene Chloride-d_2) δ = 171.3, 171.1, 170.8, 170.0, 164.1, 156.2, 153.1, 150.5, 150.4, 148.9, 139.0, 137.2, 136.8, 132.6, 132.0, 131.4, 131.2, 131.1, 130.8, 130.4 (2C), 129.6 (2C), 128.9 (2C), 128.2 (2C), 85.1, 75.9, 71.4, 71.1, 70.9, 70.8, 70.6, 70.3, 59.2, 56.9, 54.7, 54.2, 51.8, 43.3, 40.7, 39.9, 39.1, 35.9, 34.4, 29.1, 27.4, 26.5 (3C), 25.8, 25.7, 23.8, 16.3, 14.6, 13.2, 12.0; HRMS (ESI), m/z: [M+H]$^{+}$ calcd for $C_{58}H_{76}O_{10}N_{12}ClS_2^{+}$: 1199.4932 found: 1199.4921.

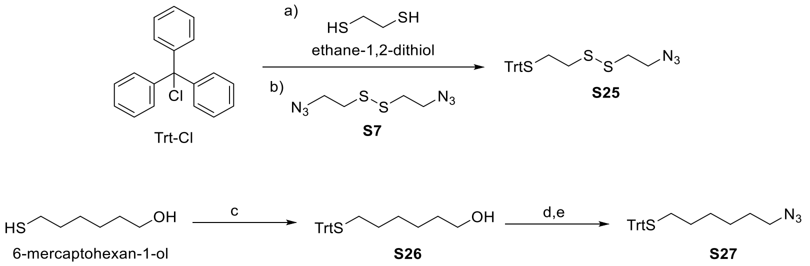

Scheme S10 Synthetic route to obtain the protected thiol linkers **S25** and **S27**. a) Ethane-1,2-dithiol (1.5 equiv), THF (1 M), 65 °C, 18 h; b) **S7** (0.33 equiv), Et_3N (1 equiv), CH_2Cl_2 (0.1 M), 20 °C, 40 h, 42% over two steps; c) Trt-Cl (1.1 equiv), THF (1 M), 65 °C, 24 h, 89%; d) MsCl (1.5 equiv), Et_3N (1.7 equiv), CH_2Cl_2 (0.5 M), 0 °C, 18 h; e) NaN_3 (1 equiv), DMF (0.1 M), 60 °C, 18 h, 71% over two steps.

***Compound S25** – 1-(2-Azidoethyl)-2-(2-(tritylthio)ethyl)disulfane*

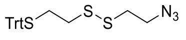

In an oven-dried Schlenk tube trityl chloride (1.00 g, 3.59 mmol) was dissolved in anhydrous THF (3.6 mL). Ethane-1,2-dithiol (0.51 g, 5.38 mmol) was added in one portion, and the reaction mixture was heated to 65 °C and stirred for 18 h. The reaction mixture was diluted with brine and extracted with CH_2Cl_2. The combined organic layers were washed with brine, dried over $MgSO_4$ and concentrated under reduced pressure to afford the crude mono-trityl ethanedithiol intermediate as a white solid (960 mg, ~2.85 mmol). **S7**[24] (109 mg, 0.54 mmol) was dissolved in CH_2Cl_2 (1.3 mL) and triethylamine (162 mg, 0.23 mL, 1.60 mmol) was added in one portion. To the stirred reaction mixture, a solution of the mono-trityl ethanedithiol intermediate (600 mg, ~1.78 mmol) in CH_2Cl_2 (5.0 mL) was added dropwise over 3 h. The reaction mixture was stirred at 20 °C for 40 h, after which it was diluted with CH_2Cl_2 and washed with NH_4Cl (sat.), dried over $MgSO_4$ and concentrated under reduced pressure. The crude material was purified by flash column chromatography (cyclohexane:CH_2Cl_2 = 90:10 to 70:30) to afford the desired compound **S25** as a yellow oil (196 mg, 0.45 mmol, 42% yield). ^{1}H NMR (400 MHz, Methylene Chloride-d_2) δ = 7.45 – 7.40 (m, 6H), 7.33 – 7.27 (m, 6H), 7.27 – 7.21 (m, 3H), 3.47 (t, *J* = 6.8 Hz, 2H), 2.67 (t, *J* = 6.8 Hz, 2H), 2.58 – 2.51 (m, 2H), 2.50 – 2.45 (m, 2H); ^{13}C NMR (101 MHz, Methylene Chloride-d_2) δ = 145.1 (3C), 130.0 (6C), 128.4 (6C), 127.2 (3C), 67.5, 50.3, 38.0, 37.8, 31.9; HRMS (ESI), m/z: [M+Na]$^{+}$ calcd for $C_{23}H_{23}N_3NaS_3^{+}$: 460.0946 found: 460.0945.

***Compound S26** – 6-(Tritylthio)hexan-1-ol*

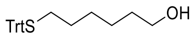

In an oven-dried flask, Trt-Cl (1.75 g, 6.27 mmol) was dissolved in anhydrous THF (6.3 mL). 6-Mercaptohexan-1-ol (0.72 g, 5.33 mmol) was added in one portion and the reaction mixture was heated to 65°C and stirred for 24 h. The reaction mixture was concentrated under reduced pressure and purified by flash column chromatography (hexane:EtOAc = 90:10 to 70:30) to afford the desired compound **S26** as a white solid (1.80 g, 4.78 mmol, 90% yield). ^{1}H NMR (400 MHz, Methylene Chloride-d_2) δ = 7.43 – 7.38 (m, 6H), 7.28 (ddd, *J* = 7.8, 6.7, 1.4 Hz, 6H), 7.24 – 7.19 (m, 3H), 3.53 (t, *J* = 6.6 Hz, 2H), 2.13 (t, *J* = 7.3 Hz, 2H), 1.48 – 1.37 (m, 4H, overlapping with water), 1.30 – 1.16 (m, 4H); ^{13}C NMR (101 MHz, Methylene Chloride-d_2) δ = 145.5 (3C), 130.0 (6C), 128.2 (6C), 126.9 (3C), 66.7, 63.0, 33.0, 32.2, 29.2, 28.9, 25.7; HRMS (ESI), m/z: [M+Na]$^+$ calcd for $C_{25}H_{28}ONaS^+$: 399.1753 found: 399.1753.

***Compound S27** – (6-Azidohexyl)(trityl)sulfane*

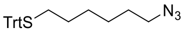

In an oven-dried flask, **S26** (1.32 g, 3.50 mmol) was dissolved in anhydrous CH_2Cl_2 (7 mL). The solution was cooled to 0 °C and dry triethylamine (0.60 g, 0.83 mL, 5.95 mmol) was added in one portion, followed by a dropwise addition of MsCl (0.60 g, 0.41 mL, 5.25 mmol). The solution was allowed to slowly warm up to 20 °C, and stirred for 18 h. The reaction mixture was cooled to 0 °C and quenched with a dropwise addition of $NaHCO_3$ (sat.) until pH 8-9. The biphasic mixture was diluted with brine and extracted with CH_2Cl_2. The combined organic phases were washed with brine, dried over $MgSO_4$ and concentrated under reduced pressure, to provide the crude intermediate as a brown oil, which was used in the next step without further purification. In a teflon flask, the oil was dissolved in anhydrous DMF (35 mL), and NaN_3 (226 mg, 3.47 mmol) was added in one portion. The reaction vessel was sealed, heated to 60 °C and stirred for 18 h. The reaction mixture was allowed to cool down to 0 °C and quenched with $NaHCO_3$ (sat.) until pH 8-9. The solution was extracted with Et_2O, the organic layers were combined and washed with ice-cold water. The organic layers were dried over $MgSO_4$ and concentrated under reduced pressure. The crude material was purified by flash column chromatography (hexane:CH_2Cl_2 = 90:10 to 70:30) to afford the desired product **S27** as a yellow oil (0.99 g, 2.46 mmol, 70% yield over two steps). ^{1}H NMR (400 MHz, Methylene Chloride-d_2) δ = 7.43 – 7.39 (m, 6H), 7.32 – 7.26 (m, 6H), 7.24 – 7.19 (m, 3H), 3.20 (t, *J* = 7.0 Hz, 2H), 2.14 (t, *J* = 7.3 Hz, 2H), 1.54 – 1.45 (m, 2H, overlapping with water), 1.42 – 1.33 (m, 2H), 1.30 – 1.18 (m, 4H). ^{13}C NMR (101 MHz, Methylene Chloride-d_2) δ = 145.5 (3C), 130.0 (6C), 128.2 (6C), 126.9 (3C), 66.8, 51.8, 32.1, 29.0, 28.8, 28.8, 26.6; HRMS (ESI), m/z: [M+Na]$^+$ calcd for $C_{25}H_{27}N_3NaS^+$: 424.1818 found: 424.1817.

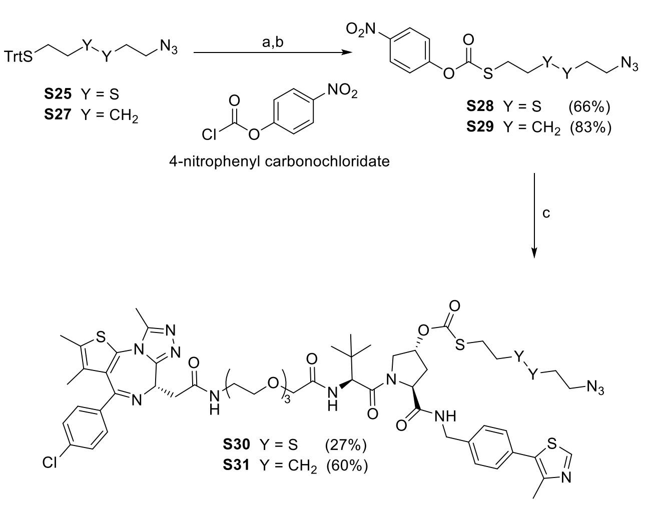

Scheme S11 Synthetic route to obtain the thiocarbonate **MZ1**-N_3 intermediates **S30** and **S31**. a) TFA (15%), TES (5%), CH_2Cl_2 (0.1 M), 0 °C, 10 min; b) 4-Nitropenyl carbonochloridate (2 equiv), DMAP (2 equiv), CH_2Cl_2 (0.1 M), 20 °C, 1 h, 66

& 83% over two steps; c) **S28** (2 equiv) or **S29** (1.5 equiv) **MZ1** (1 equiv), DMAP (2 or 1.5 equiv), CH_2Cl_2 (0.05 M), 20 °C, 72 or 96 h, 27 & 60%.

Compound S28 *– S-(2-((2-Azidoethyl)disulfaneyl)ethyl) O-(4-nitrophenyl) carbonothioate*

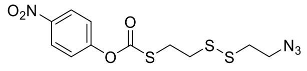

In an oven dried flask, **S25** (90 mg, 0.206 mmol) was dissolved in anhydrous CH_2Cl_2 (0.1 mL) and cooled to 0 °C. An ice-cold mixture of CH_2Cl_2:TFA:TES (80:15:5) (2 mL) was added and stirred at 0 °C for 10 min. The reaction was diluted with brine and CH_2Cl_2. The aqueous layer was carefully neutralised on ice to pH 7 by the addition of $NaHCO_3$ (sat.), and the aqueous layer was extracted with CH_2Cl_2. The organic layers were combined and washed with brine, over $MgSO_4$ and concentrated under reduced pressure (the rotary evaporator water batch was kept at 20 °C). The residue was used immediately in a new oven-dried flask, where it was dissolved in anhydrous CH_2Cl_2 (2 mL). In one portion, 4-nitrophenyl carbonochloridate (82.9 mg, 0.411 mmol) and DMAP (50.2 mg, 0.411 mmol) were added to the solution. The reaction mixture was stirred at 20 °C for 1 h, after which the reaction was quenched with brine and diluted with CH_2Cl_2. The phases were separated, and the aqueous layer was extracted with CH_2Cl_2. The organic layers were combined, washed with brine, dried over $MgSO_4$ and concentrated under reduced pressure. The crude material was loaded on fluorosil and purified by flash column chromatography (Cyclohexane:CH_2Cl_2 = 80:20 to 0:100) to afford the desired product **S28** as a colourless oil (49 mg, 0.136 mmol, 66% yield). ^{1}H NMR (400 MHz, Chloroform-*d*) δ = 8.32 – 8.24 (m, 2H), 7.39 – 7.34 (m, 2H), 3.61 (t, *J* = 6.8 Hz, 2H), 3.35 – 3.27 (m, 2H), 3.04 – 2.96 (m, 2H), 2.88 (t, *J* = 6.8 Hz, 2H); ^{13}C NMR (101 MHz, Chloroform-*d*) δ = 169.2, 155.6, 145.7, 125.5 (2C), 122.1 (2C), 50.1, 37.7,37.7, 30.9; HRMS (ESI), m/z: $[M+Cl]^-$ calcd for $C_{11}H_{12}O_4N_4ClS_3^-$: 394.9715 found: 394.9711.

Compound S29 *– S-(6-Azidohexyl) O-(4-nitrophenyl) carbonothioate*

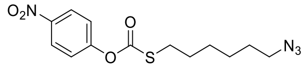

In an oven-dried flask, **S27** (301 mg, 0.75 mmol) was dissolved in anhydrous CH_2Cl_2 (0.5 mL) and cooled to 0 °C. An ice-cold mixture of CH_2Cl_2:TFA:TES (80:15:5) (7.5 mL) was added and stirred at 0 °C for 10 min. The reaction was diluted with brine and CH_2Cl_2. The aqueous layer was carefully neutralised on ice to pH 7 by the addition of $NaHCO_3$ (sat.), and the aqueous layer was extracted with CH_2Cl_2. The organic layers were combined and washed with brine, over $MgSO_4$ and concentrated under reduced pressure (the rotary evaporator water batch was kept at 20 °C). The residue was used immediately in a new oven-dried flask, where it was dissolved in anhydrous CH_2Cl_2 (7.5 mL). In one portion, 4-nitrophenyl carbonochloridate (302 mg, 1.5 mmol) and DMAP (183 mg, 1.5 mmol) were added to the solution. The reaction mixture was stirred at 20 °C for 1 h, after which the reaction was quenched with brine and diluted with CH_2Cl_2. The phases were separated, and the aqueous layer was extracted with CH_2Cl_2. The organic layers were combined, washed with brine, dried over $MgSO_4$ and concentrated under reduced pressure. The crude material was loaded on fluorosil and purified by flash column chromatography (Cyclohexane:CH_2Cl_2 = 80:20 to 0:100) to afford the desired product **S29** as a colourless oil (202 mg, 0.623 mmol, 83% yield). ^{1}H NMR (400 MHz, Chloroform-*d*) δ = 8.30 – 8.25 (m, 2H), 7.39 – 7.32 (m, 2H), 3.28 (t, *J* = 6.8 Hz, 2H), 2.97 (t, *J* = 7.3 Hz, 2H), 1.78 – 1.68 (m, 2H), 1.68 – 1.56 (m, 2H), 1.53 – 1.36 (m, 4H); ^{13}C NMR (101 MHz, Chloroform-*d*) δ = 169.8, 155.8, 145.6, 125.4 (2C), 122.2 (2C), 51.5, 31.4, 29.5, 28.8, 28.3, 26.3; HRMS (ESI), m/z: $[M+H]^+$ calcd for $C_{13}H_{17}O_4N_2S^+$: 297.0904 found: 297.0905.

Compound S30 – *S-(2-((2-Azidoethyl)disulfaneyl)ethyl)O-((3R,5S)-1-((S)-2-(tert-butyl)-17-((S)-4-(4-chlorophenyl)-2,3,9-trimethyl-6H-thieno[3,2-f][1,2,4]triazolo[4,3-a][1,4]diazepin-6-yl)-4,16-dioxo-6,9,12-trioxa-3,15-diazaheptadecanoyl)-5-((4-(4-methylthiazol-5-yl)benzyl)carbamoyl)pyrrolidin-3-yl) carbonothioate*

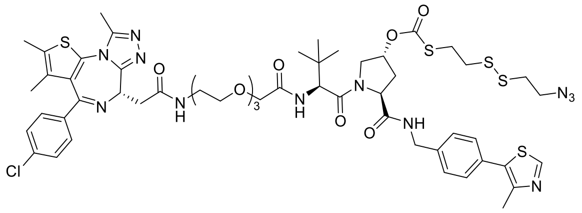

In an oven-dried flask, **S28** (28.8 mg, 0.08 mmol) was dissolved in dry CH_2Cl_2 (0.8 mL), in one portion DMAP (9.75 mg, 0.08 mmol) and **MZ1**[20] (40 mg, 0.04 mmol) were added. The reaction was stirred at 20 °C for 72 h. The reaction mixture was concentrated under reduced pressure and purified by flash column chromatography (CH_2Cl_2:MeOH = 95:5 to 90:10), by preparative TLC (CH_2Cl_2:MeOH = 95:5) and eventually by reverse-phase HPLC using a Shimadzu Nexera system, equipped with an Agilent Zorbax 300SB-C18 semi-preparative column (9.4 x 250 mm 5-micron) using 25 to 55% MeCN in water buffered with 0.1% formic acid as eluent (4 mL/min) to afford **S30** as an off-white solid (13 mg. 0.011 mmol, 27% yield). ^{1}H NMR (500 MHz, Methylene Chloride-d_2) δ = 8.65 (s, 1H), 7.82 (t, *J* = 6.2 Hz, 1H), 7.41 (d, *J* = 8.4 Hz, 2H), 7.37 – 7.29 (m, 6H), 7.28 – 7.19 (m, 2H), 5.49 (p, *J* = 2.6 Hz, 1H), 4.78 (t, *J* = 7.6 Hz, 1H), 4.63 – 4.54 (m, 2H), 4.51 (dd, *J* = 15.2, 6.6 Hz, 1H), 4.28 (dd, *J* = 15.2, 5.4 Hz, 1H), 4.15 (dt, *J* = 12.1, 1.9 Hz, 1H), 4.04 (s, 2H), 3.90 (dd, *J* = 11.8, 4.7 Hz, 1H), 3.72 – 3.58 (m, 10H), 3.56 – 3.51 (m, 2H), 3.50 – 3.42 (m, 1H), 3.42 – 3.32 (m, 2H), 3.29 (dd, *J* = 14.8, 7.5 Hz, 1H), 3.23 – 3.11 (m, 2H), 2.94 (dt, *J* = 9.2, 6.2 Hz, 2H), 2.87 (t, *J* = 6.7 Hz, 2H), 2.59 (s, 3H), 2.57 – 2.49 (m, 1H), 2.47 (s, 3H), 2.39 (s, 3H), 2.35 – 2.27 (m, 1H), 1.67 (s, 3H), 0.97 (s, 9H); ^{13}C NMR (126 MHz, Methylene Chloride-d_2) δ = 171.2, 171.1, 170.8, 170.5, 170.1, 164.1, 156.2, 150.5, 150.4, 148.9, 139.0, 137.2, 136.9, 132.6, 132.0, 131.5, 131.2, 131.1, 130.8, 130.4 (2C), 129.7 (2C), 129.0 (2C), 128.2 (2C), 76.8, 71.4, 71.0, 70.9, 70.8, 70.5, 70.3, 59.1, 56.9, 54.7, 54.0 (visible in DEPT135), 50.4, 43.3, 39.9, 39.1, 38.3, 38.0, 35.8, 34.3, 30.8, 26.5 (3C), 16.3, 14.6, 13.3, 12.0; HRMS (ESI), m/z: $[M+H]^+$ calcd for $C_{54}H_{68}O_9N_{12}ClS_5^+$: 1223.3519 found: 1223.3519.

Compound S31 – *S-(6-Azidohexyl)O-((3R,5S)-1-((S)-2-(tert-butyl)-17-((S)-4-(4-chlorophenyl)-2,3,9-trimethyl-6H-thieno[3,2-f][1,2,4]triazolo[4,3-a][1,4]diazepin-6-yl)-4,16-dioxo-6,9,12-trioxa-3,15-diazaheptadecanoyl)-5-((4-(4-methylthiazol-5-yl)benzyl)carbamoyl)pyrrolidin-3-yl) carbonothioate*

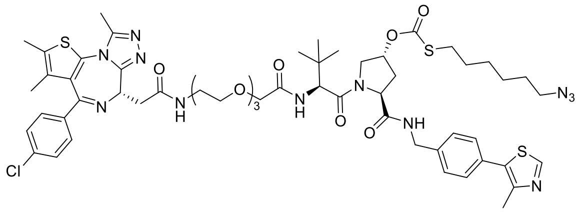

In an oven-dried flask, **S29** (17 mg, 0.052 mmol) was dissolved in dry CH_2Cl_2 (0.7 mL). In one portion, DMAP (6.4 mg, 0.052 mmol) and **MZ1**[20] (35 mg, 0.035 mmol) were added. The reaction was stirred at 20 °C for 96 h. The reaction mixture was concentrated under reduced pressure and purified by flash column chromatography (CH_2Cl_2:MeOH = 95:5 to 90:10) and by preparative TLC (CH_2Cl_2:MeOH = 95:5) and triturated with pentane to afford **S31** as a colourless solid (25 mg. 0.210 mmol, 60% yield). ^{1}H NMR (500 MHz, Methylene Chloride-d_2) δ = 8.66 (s, 1H), 7.79 (t, *J* = 6.1 Hz, 1H), 7.44 – 7.38 (m, 2H), 7.36 – 7.29 (m, 6H), 7.26 – 7.20 (m, 2H), 5.49 – 5.47 (m, 1H), 4.77 (t, *J* = 7.5 Hz, 1H), 4.62 – 4.54 (m, 2H), 4.51 (dd, *J* = 15.2, 6.7 Hz, 1H), 4.28 (dd, *J* = 15.2, 5.4 Hz, 1H), 4.10 – 4.05 (m, 1H), 4.04 (d, *J* = 1.9 Hz, 2H), 3.91 (dd, *J* = 11.7, 4.9 Hz, 1H), 3.74 – 3.58 (m, 8H), 3.57 – 3.51 (m, 2H), 3.51 – 3.42 (m, 1H), 3.42 – 3.32 (m, 2H), 3.32 – 3.22 (m, 3H), 2.84 (t, *J* = 7.3 Hz, 2H), 2.59 (s, 3H), 2.55 – 2.49 (m, 1H), 2.48 (s, 3H), 2.39 (s, 3H), 2.34 – 2.25 (m, 1H), 1.67 (s, 3H), 1.61 –

1.55 (m, 4H), 1.39 (tt, J = 5.2, 2.2 Hz, 4H), 0.97 (s, 9H); ^{13}C NMR (126 MHz, Methylene Chloride-d_2) δ = 171.2, 171.2, 171.1, 170.8, 170.0, 164.1, 156.2, 150.5, 150.4, 148.9, 139.0, 137.2, 136.9, 132.6, 132.0, 131.4, 131.2, 131.1, 130.8, 130.4 (2C), 129.7 (2C), 128.9 (2C), 128.2 (2C), 76.2, 71.4, 71.0, 70.9, 70.8, 70.6, 70.3, 59.1, 56.9, 54.7, 54.0, 51.8, 43.3, 39.9, 39.1, 35.9, 34.3, 31.3, 29.9, 29.1, 28.5, 26.6, 26.5 (3C), 16.3, 14.6, 13.2, 12.0; HRMS (ESI), m/z: $[M+H]^+$ calcd for $C_{56}H_{72}O_9N_{12}ClS_3^+$: 1187.4390 found: 1187.4390.

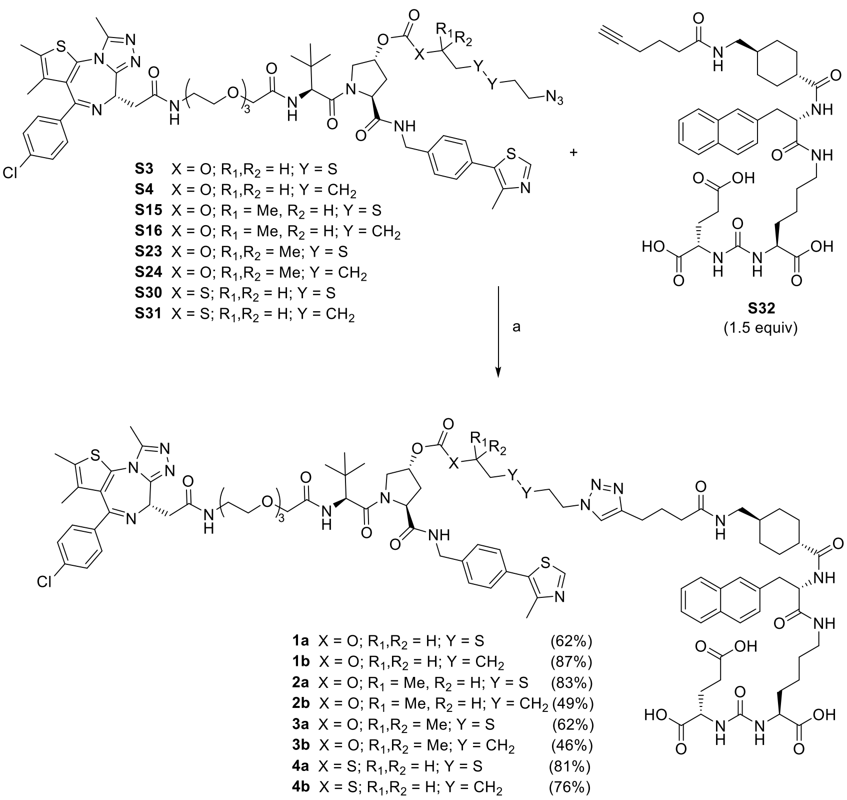

Scheme S12 The final CuAAC to form the PCa-targeting SelecPROTACs **1a**-**4b**. a) $CuSO_4 \cdot 5H_2O$ (1.5 equiv), sodium ascorbate (1.5 equiv), TBTA (0.2 equiv), NMP (0.02 M), 20 °C, 16 h, 46–87%.

Compound 1a *– (((1S)-1-Carboxy-5-((2S)-2-(4-((4-(1-(2-((2-(((((3R,5S)-1-((S)-2-(1-((S)-4-(4-chlorophenyl)-2,3,9-trimethyl-6H-thieno[3,2-f][1,2,4]triazolo[4,3-a][1,4]diazepin-6-yl)-2-oxo-6,9,12-trioxa-3-azatetradecan-14-amido)-3,3-dimethylbutanoyl)-5-((4-(4-methylthiazol-5-yl)benzyl)-carbamoyl)pyrrolidin-3-yl)oxy)carbonyl)oxy)ethyl)disulfaneyl)ethyl)-1H-1,2,3-triazol-4-yl)-butanamido)methyl)cyclohexane-1-carboxamido)-3-(naphthalen-2-yl)propanamido)pentyl)-carbamoyl)-L-glutamic acid*

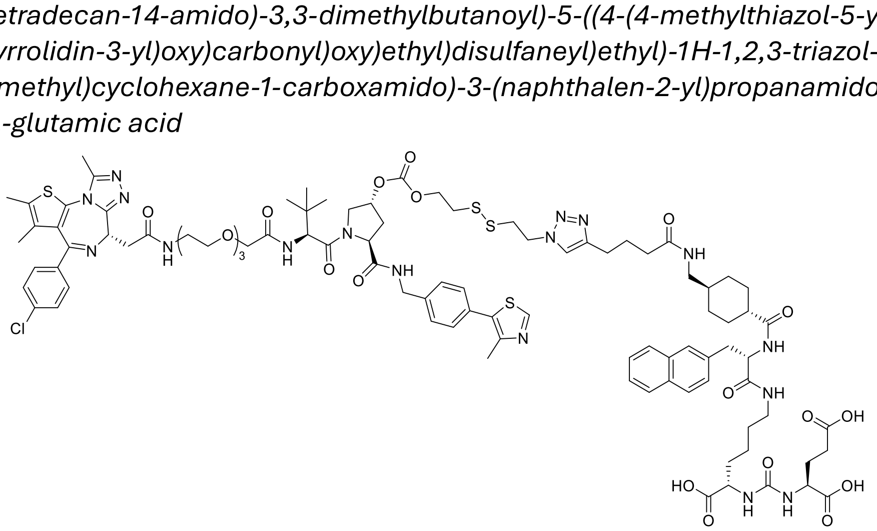

1a was prepared according to the general procedure A, from **S3** (7 mg, 5.8 µmol) to afford **1a** as a white solid (7 mg, 3.6 µmol, 62% yield). ^{1}H NMR (500 MHz, DMSO-d_6) δ = 8.97 (s, 1H), 8.64 (t, *J* = 6.1 Hz, 1H), 8.27 (t, *J* = 5.7 Hz, 1H), 7.98 – 7.93 (m, 2H), 7.88 (s, 1H), 7.83 (dd, *J* = 7.7, 1.6 Hz, 1H), 7.80 – 7.75 (m, 2H), 7.71 (t, *J* = 5.8 Hz, 1H), 7.68 (d, *J* = 1.6 Hz, 1H), 7.49 – 7.36 (m, 12H), 6.36 (d, *J* = 8.0 Hz, 1H), 6.25 (d, *J* = 7.6 Hz, 1H), 5.22 (t, *J* = 4.9 Hz, 1H), 4.61 – 4.56 (m, 2H), 4.56 – 4.43 (m, 4H), 4.40 (dd, *J* = 15.7, 6.3 Hz, 1H), 4.35 – 4.25 (m, 3H), 4.08 – 3.93 (m, 5H), 3.85 (dd, *J* = 12.0, 3.9 Hz, 1H), 3.62 – 3.52 (m, 9H), 3.10 (dd, *J* = 13.6, 5.1 Hz, 1H), 3.06 – 2.96 (m, 4H), 2.92 (dd, *J* = 13.6, 9.5 Hz, 1H), 2.84 (t, *J* = 6.2 Hz, 2H), 2.60 – 2.54 (m, 5H), 2.43 (s, 3H), 2.41 – 2.39 (m, 3H), 2.37 – 2.30 (m, 1H), 2.29 – 2.02 (m, 6H), 1.82 – 1.70 (m, 4H), 1.68 – 1.54 (m, 7H), 1.50 – 1.40 (m, 2H), 1.35 – 1.14 (m, 6H), 1.08 – 0.88 (m, 10H), 0.85 – 0.73 (m, 2H), (Note: three OH protons not detectable, seven proton signals overlapping with water); ^{13}C NMR (126 MHz, DMSO-d_6) δ = 174.9, 174.7, 174.3, 174.1, 171.6, 171.0, 170.9, 169.7, 169.3, 169.0, 163.0, 157.2, 155.1, 153.6, 151.5, 149.8, 147.8, 146.5, 139.2, 136.7, 135.8, 135.2, 132.9, 132.3, 131.7, 131.1, 130.7, 130.1 (2C), 129.8, 129.8, 129.5, 128.7 (2C), 128.4 (2C), 127.9, 127.5 (2C), 127.4 (2C), 127.3, 127.3, 125.9, 125.3, 122.1, 77.0, 70.4, 69.8, 69.6 (2C), 69.5, 69.2, 65.3, 58.2, 56.1, 53.8, 53.7, 53.5, 52.4, 52.2, 47.9, 44.7, 43.6, 41.7, 38.6, 38.3, 38.2, 37.5, 37.1, 37.0, 36.0, 35.1, 34.8, 34.7, 31.7, 29.7, 29.5, 28.8, 28.7, 28.3, 26.1 (3C), 25.3, 24.7, 22.6, 15.9, 14.0, 12.7, 11.3, (Note: two signals missing, likely due to overlapping with the solvent); HRMS (ESI), m/z: [M+2H]$^{2+}$ calcd for $C_{93}H_{120}O_{20}N_{17}ClS_4^{2+}$: 978.8728 found: 978.8740.

***Compound 1b** – (((1S)-1-Carboxy-5-((2S)-2-(4-((4-(1-(6-(((((3R,5S)-1-((S)-2-(1-((S)-4-(4-chlorophenyl)-2,3,9-trimethyl-6H-thieno[3,2-f][1,2,4]triazolo[4,3-a][1,4]diazepin-6-yl)-2-oxo-6,9,12-trioxa-3-azatetradecan-14-amido)-3,3-dimethylbutanoyl)-5-((4-(4-methylthiazol-5-yl)benzyl)carbamoyl)pyrrolidin-3-yl)oxy)carbonyl)oxy)hexyl)-1H-1,2,3-triazol-4-yl)butanamido)methyl)cyclohexane-1-carboxamido)-3-(naphthalen-2-yl)propanamido)pentyl)carbamoyl)-L-glutamic acid*

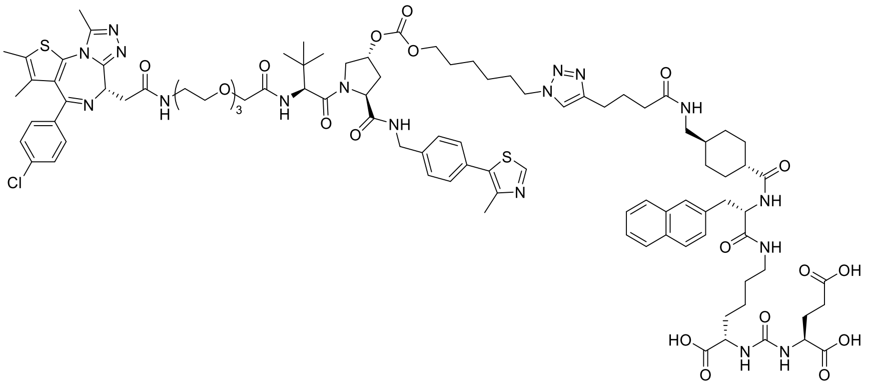

1b was prepared according to the general procedure A, from **S4** (7 mg, 6.0 µmol) to afford **1b** as a white solid (10 mg, 5.2 µmol, 87% yield). ^{1}H NMR (500 MHz, DMSO-d_6) δ = 8.97 (s, 1H), 8.63 (t, *J* = 6.1 Hz, 1H), 8.27 (t, *J* = 5.7 Hz, 1H), 7.99 – 7.92 (m, 2H), 7.85 – 7.82 (m, 2H), 7.80 – 7.75 (m, 2H), 7.71 (t, *J* = 5.8 Hz, 1H), 7.68 (d, *J* = 1.6 Hz, 1H), 7.50 – 7.35 (m, 12H), 6.36 (d, *J* = 8.0 Hz, 1H), 6.24 (d, *J* = 5.1 Hz, 1H), 5.20 (s, 1H), 4.55 – 4.42 (m, 4H), 4.39 (dd, *J* = 15.7, 6.3 Hz, 1H), 4.30 – 4.24 (m, 3H), 4.09 – 3.93 (m, 7H), 3.85 (dd, *J* = 11.9, 3.9 Hz, 1H), 3.62 – 3.52 (m, 9H), 3.10 (dd, *J* = 13.6, 5.1 Hz, 1H), 3.06 – 2.96 (m, 2H), 2.92 (dd, *J* = 13.6, 9.5 Hz, 1H), 2.84 (t, *J* = 6.2 Hz, 2H), 2.58 (s, 3H), 2.55 (t, *J* = 7.7 Hz, 2H), 2.43 (s, 3H), 2.40 (s, 3H), 2.35 – 2.23 (m, 2H), 2.23 – 2.03 (m, 5H), 1.83 – 1.72 (m, 5H), 1.68 – 1.52 (m, 10H), 1.51 – 1.39 (m, 2H), 1.35 – 1.27 (m, 4H), 1.27 – 1.16 (m, 6H), 1.07 – 0.97 (m, 1H), 0.95 (s, 9H), 0.86 – 0.73 (m, 2H), (Note: three OH protons not detectable, five proton signals overlapping with water); ^{13}C NMR (126 MHz, DMSO-d_6) δ = 174.9, 174.7, 174.2, 174.1, 171.6, 171.0, 170.9, 169.7, 169.2, 168.9, 163.0, 157.2, 155.1, 153.9, 151.5, 149.8, 147.8, 146.4, 139.2, 136.7, 135.8, 135.2, 132.9, 132.3, 131.7, 131.1, 130.7, 130.1 (2C), 129.8, 129.8, 129.5, 128.7 (2C), 128.4 (2C), 127.9, 127.5 (2C), 127.4 (2C), 127.3 (2C), 125.9, 125.3, 121.7, 76.6,

70.4, 69.8, 69.6 (2C), 69.5, 69.2, 67.6, 58.3, 56.0, 53.8, 53.7, 53.6, 52.3, 52.2, 49.0, 44.6, 43.6, 41.7, 38.6, 38.3, 38.2, 37.5, 37.0, 35.1, 34.8, 34.7, 31.7, 29.7, 29.5 (2C), 28.8, 28.7, 28.3, 27.8, 26.1 (3C), 25.5, 25.3, 24.7, 24.5, 22.6, 15.9, 14.0, 12.7, 11.3, (Note: two signals missing, likely due to overlapping with the solvent); HRMS (ESI), m/z: $[M-H]^-$ calcd for $C_{95}H_{121}O_{20}N_{17}ClS_2^-$: 1918.8109 found: 1918.8091.

Compound 2a - *(((1S)-1-Carboxy-5-((2S)-2-(4-((4-(1-(2-((2-(((((3R,5S)-1-((S)-2-(1-((S)-4-(4-chlorophenyl)-2,3,9-trimethyl-6H-thieno[3,2-f][1,2,4]triazolo[4,3-a][1,4]diazepin-6-yl)-2-oxo-6,9,12-trioxa-3-azatetradecan-14-amido)-3,3-dimethylbutanoyl)-5-((4-(4-methylthiazol-5-yl)benzyl)carbamoyl)pyrrolidin-3-yl)oxy)carbonyl)oxy)propyl)disulfaneyl)ethyl)-1H-1,2,3-triazol-4-yl)butanamido)methyl)cyclohexane-1-carboxamido)-3-(naphthalen-2-yl)propanamido)pentyl)carbamoyl)-L-glutamic acid*

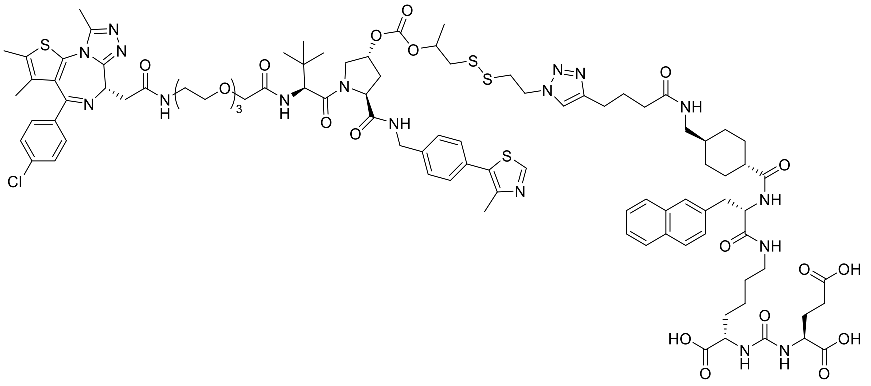

2a was prepared according to the general procedure A, from **S15** (7.3 mg, 5.9 µmol) to afford **2a** as a white solid (9.7 mg, 4.9 µmol, 83% yield). ^{1}H NMR (500 MHz, DMSO-d_6) δ = 8.97 (d, *J* = 1.0 Hz, 1H), 8.65 (dt, *J* = 16.3, 6.0 Hz, 1H), 8.27 (t, *J* = 5.7 Hz, 1H), 8.03 – 7.93 (m, 2H), 7.88 (d, *J* = 2.9 Hz, 1H), 7.86 – 7.81 (m, 1H), 7.80 – 7.76 (m, 2H), 7.71 (t, *J* = 5.7 Hz, 1H), 7.68 (d, *J* = 1.6 Hz, 1H), 7.50 – 7.35 (m, 12H), 6.38 (s, 1H), 6.22 (s, 1H), 5.21 (s, 1H), 4.94 – 4.82 (m, 1H), 4.64 – 4.55 (m, 2H), 4.55 – 4.35 (m, 5H), 4.27 (ddd, *J* = 15.7, 5.7, 2.9 Hz, 1H), 4.10 – 3.92 (m, 5H), 3.89 – 3.80 (m, 1H), 3.63 – 3.51 (m, 9H), 3.13 – 2.89 (m, 6H), 2.84 (t, *J* = 6.2 Hz, 2H), 2.60 – 2.54 (m, 5H), 2.43 (s, 3H), 2.40 (s, 3H), 2.35 – 2.24 (m, 2H), 2.21 – 2.02 (m, 5H), 1.87 – 1.72 (m, 3H), 1.68 – 1.54 (m, 8H), 1.50 – 1.39 (m, 2H), 1.35 – 1.13 (m, 9H), 1.04 – 0.89 (m, 10H), 0.87 – 0.72 (m, 2H), (Note: three OH protons not detectable, seven proton signals overlapping with water); ^{13}C NMR (126 MHz, DMSO-d_6) δ = 174.9, 174.9, 174.5, 174.4, 171.6, 171.0, 171.0, 170.9, 169.7, 169.3, 169.2, 169.0, 168.9, 163.0, 157.1, 155.1, 153.3, 153.2, 151.5, 149.8, 147.7, 146.6, 146.5, 139.2, 136.7, 135.8, 135.2, 132.9, 132.3, 131.7, 131.1 (2C), 130.7, 130.1, 129.8, 129.8, 129.5, 128.7 (2C), 128.4 (2C), 127.9, 127.5 (2C), 127.4 (2C), 127.3 (2C), 125.9, 125.3, 122.1, 76.8, 73.3, 73.1, 70.4, 70.4, 69.8, 69.6 (2C), 69.5, 69.5, 69.2, 58.2, 56.1, 56.0, 53.8, 53.8, 53.6, 52.7, 52.7, 52.6, 47.9, 44.7, 43.6, 43.1, 42.8, 41.7, 38.6, 38.3, 38.1, 37.5, 37.1, 37.0, 35.2, 35.1, 34.8, 34.8, 34.8, 34.7, 31.7, 29.7, 29.6, 29.5, 28.8, 28.3, 26.1 (3C), 25.3, 24.7, 24.7, 22.6, 18.8, 18.6, 15.9, 14.0, 12.7, 11.3. (Note: due to the presence of two diastereomers, several peaks are duplicated, but not all; two signals are missing, likely due to overlapping with the solvent); HRMS (ESI), m/z: $[M+2H]^{2+}$ calcd for $C_{94}H_{122}O_{20}N_{17}ClS_4^{2+}$: 985.8806 found: 985.8806.

Compound 2b – *(((1S)-1-Carboxy-5-((2S)-2-(4-((4-(1-(6-(((((3R,5S)-1-((S)-2-(1-((S)-4-(4-chlorophenyl)-2,3,9-trimethyl-6H-thieno[3,2-f][1,2,4]triazolo[4,3-a][1,4]diazepin-6-yl)-2-oxo-6,9,12-trioxa-3-azatetradecan-14-amido)-3,3-dimethylbutanoyl)-5-((4-(4-methylthiazol-5-yl)benzyl)carbamoyl)pyrrolidin-3-yl)oxy)carbonyl)oxy)heptyl)-1H-1,2,3-triazol-4-yl)butanamido)methyl)cyclohexane-1-carboxamido)-3-(naphthalen-2-yl)propanamido)pentyl)carbamoyl)-L-glutamic acid*

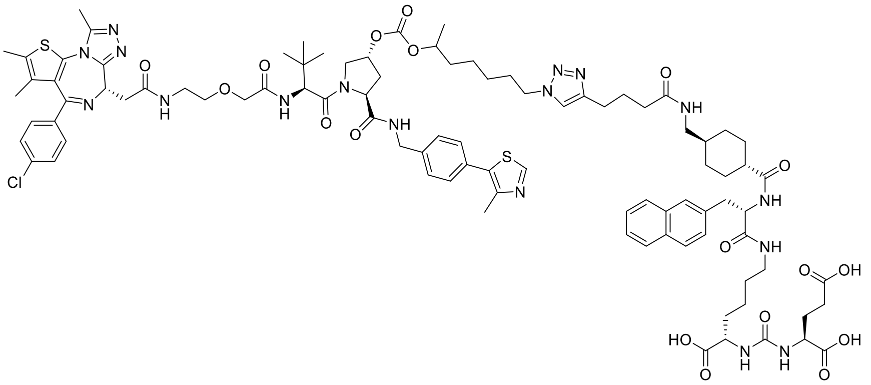

2b was prepared according to the general procedure A, from **S16** (10.1 mg, 8.5 μmol) to afford **2b** as a white solid (8 mg, 4.1 μmol, 49% yield). ^{1}H NMR (500 MHz, DMSO-d_6) δ = 8.97 (s, 1H), 8.63 (q, *J* = 5.9 Hz, 1H), 8.27 (t, *J* = 5.7 Hz, 1H), 7.97 – 7.91 (m, 2H), 7.86 – 7.81 (m, 2H), 7.80 – 7.75 (m, 2H), 7.71 (t, *J* = 5.8 Hz, 1H), 7.68 (s, 1H), 7.48 – 7.36 (m, 12H), 6.34 (d, *J* = 8.1 Hz, 1H), 6.26 (d, *J* = 7.8 Hz, 1H), 5.23 – 5.17 (m, 1H), 4.64 (h, *J* = 6.1 Hz, 1H), 4.46 (dtdd, *J* = 24.9, 21.2, 10.1, 5.4 Hz, 5H), 4.27 (qd, *J* = 7.4, 3.8 Hz, 3H), 4.10 – 3.91 (m, 5H), 3.88 – 3.81 (m, 1H), 3.65 – 3.52 (m, 9H), 3.10 (dd, *J* = 13.7, 5.0 Hz, 1H), 3.01 (dp, *J* = 24.6, 6.4 Hz, 2H), 2.92 (dd, *J* = 13.6, 9.5 Hz, 1H), 2.84 (t, *J* = 6.3 Hz, 2H), 2.58 (s, 3H), 2.55 (td, *J* = 7.7, 2.3 Hz, 2H), 2.43 (s, 3H), 2.40 (s, 3H), 2.34 – 2.17 (m, 3H), 2.16 – 2.02 (m, 4H), 1.82 – 1.72 (m, 6H), 1.69 – 1.52 (m, 8H), 1.51 – 1.41 (m, 3H), 1.36 – 1.14 (m, 13H), 1.09 – 0.91 (m, 10H), 0.85 – 0.73 (m, 2H), (Note: three OH protons not detectable, five proton signals overlapping with water); ^{13}C NMR (126 MHz, DMSO-d_6) δ = 174.9, 174.7, 174.2, 174.0, 171.6, 171.0, 171.0, 171.0, 169.7, 169.2, 169.2, 168.9, 168.9, 163.0, 157.2, 155.1, 153.5, 153.5, 151.5, 149.8, 147.8, 146.4, 146.4, 139.2, 136.7, 135.8, 135.2, 132.9, 132.3, 131.7, 131.1, 130.7, 130.1 (2C), 129.8, 129.8, 129.5, 128.7 (2C), 128.4 (2C), 127.9, 127.5 (2C), 127.4 (2C), 127.3, 127.2, 125.9, 125.3, 121.6, 76.4, 75.0, 70.4, 70.4, 69.8, 69.6 (2C), 69.5, 69.2, 58.3, 56.0, 53.8, 53.7, 53.7, 52.3, 52.0, 49.1, 49.0, 44.6, 43.6, 41.7, 38.6, 38.3, 38.2, 37.5, 37.0, 35.2, 35.1, 34.9, 34.9, 34.9, 34.8, 34.7, 31.7, 29.7, 29.5 (2C), 28.8, 28.7, 28.3, 26.1 (2C), 25.7, 25.6, 25.3, 24.7, 24.1, 24.0, 22.6, 19.5, 19.5, 15.9, 14.0, 12.7, 11.3, (Note: due to the presence of two diastereomers, several peaks are duplicated, but not all; two signals missing, likely due to overlapping with the solvent); HRMS (ESI), m/z: $[M+2H]^{2+}$ calcd for $C_{96}H_{126}O_{20}N_{17}ClS_2^{2+}$: 967.9242 found: 967.9244.

Compound 3a – *(((S)-1-Carboxy-5-((S)-2-((1r,4S)-4-((4-(1-(2-((2-(((((3R,5S)-1-((S)-2-(1-((S)-4-(4-chlorophenyl)-2,3,9-trimethyl-6H-thieno[3,2-f][1,2,4]triazolo[4,3-a][1,4]diazepin-6-yl)-2-oxo-6,9,12-trioxa-3-azatetradecan-14-amido)-3,3-dimethylbutanoyl)-5-((4-(4-methylthiazol-5-yl)benzyl)carbamoyl)pyrrolidin-3-yl)oxy)carbonyl)oxy)-2-methylpropyl)disulfaneyl)ethyl)-1H-1,2,3-triazol-4-yl)butanamido)methyl)cyclohexane-1-carboxamido)-3-(naphthalen-2-yl)propanamido)pentyl)carbamoyl)-L-glutamic acid*

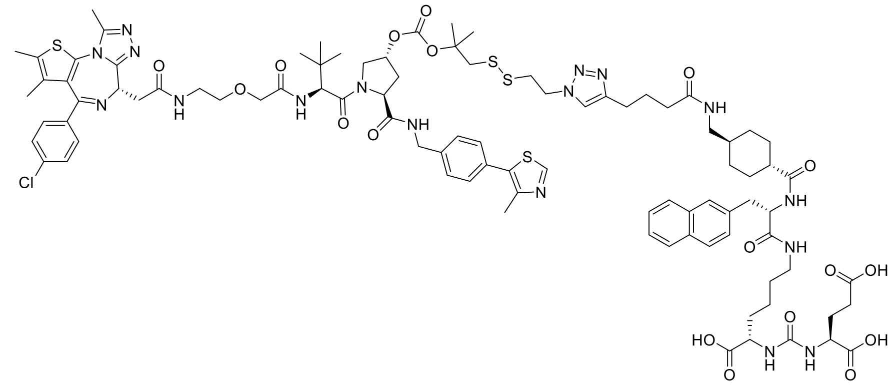

3a was prepared according to the general procedure A, from **S23** (7.5 mg, 6.1 μmol) to afford **3a** as a white solid (7.5 mg, 3.8 μmol, 62% yield). ^{1}H NMR (500 MHz, DMSO-d_6) δ = 8.97 (s, 1H), 8.65 (t, *J* = 6.0 Hz, 1H), 8.27 (t, *J* = 5.7 Hz, 1H), 8.00 – 7.93 (m, 2H), 7.88 (s, 1H), 7.83 (dd, *J* = 7.4, 1.5 Hz, 1H), 7.80 – 7.75 (m, 2H), 7.71 (t, *J* = 5.8 Hz, 1H), 7.68 (d, *J* = 1.6 Hz, 1H), 7.48 – 7.38 (m, 12H), 6.38 (d, *J* = 8.0 Hz, 1H), 6.23 (d, *J* = 4.9 Hz, 1H), 5.16 (s, 1H), 4.62 – 4.57 (m, 2H), 4.55 – 4.36 (m, 5H), 4.27 (dd, *J* = 15.8, 5.6 Hz, 1H), 4.06 – 3.92 (m, 5H), 3.84 (dd, *J* = 11.8, 3.9 Hz, 1H), 3.62 – 3.52 (m, 9H), 3.10 (dd, *J* = 13.6, 5.1 Hz, 1H), 3.01 (dp, *J* = 19.2, 6.4 Hz, 2H), 2.92 (dd, *J* = 13.6, 9.5 Hz, 1H), 2.84 (t, *J* = 6.3 Hz, 2H), 2.60 – 2.54 (m, 5H), 2.43 (s, 3H), 2.40 (s, 3H), 2.33 – 2.24 (m, 2H), 2.21 – 2.02 (m, 5H), 1.86 – 1.73 (m, 3H), 1.70 – 1.54 (m, 8H), 1.49 – 1.42 (m, 8H), 1.34 – 1.14 (m, 6H), 1.08 – 0.92 (m, 10H), 0.86 – 0.72 (m, 2H), (Note: three OH protons not detectable, nine proton signals overlapping with water); ^{13}C NMR (126 MHz, DMSO-d_6) δ = 174.9, 174.7, 174.3, 174.2, 171.6, 171.0, 171.0, 169.7, 169.2, 168.9, 163.0, 157.2, 155.1, 152.0, 151.5, 149.8, 147.7, 146.5, 139.2, 136.7, 135.8, 135.2, 132.9, 132.3, 131.7, 131.1, 130.7 (2C), 130.1, 129.8, 129.8, 129.5, 128.7 (2C), 128.4 (2C), 127.9, 127.5 (2C), 127.4 (2C), 127.3 (2C), 125.9, 125.3, 122.1, 83.0, 76.2, 70.4, 69.8 (2C), 69.6, 69.5, 69.2, 58.3, 56.0, 53.8, 53.7, 53.6, 52.4, 52.4, 48.4, 47.8, 44.7, 43.6, 41.7, 38.6, 38.3, 38.1, 37.5, 37.3, 37.0, 35.2, 34.8, 34.8, 31.6, 29.7, 29.5, 28.8, 28.7, 28.3, 26.1 (3C), 25.3, 25.0, 25.0, 24.7, 22.6, 15.9, 14.0, 12.7, 11.3, (Note: two signals missing, likely due to overlapping with the solvent); HRMS (ESI), m/z: $[M+2H]^{2+}$ calcd for $C_{95}H_{124}O_{20}N_{17}ClS_4^{2+}$: 992.8885 found: 992.8888.

***Compound 3b** - (((S)-1-Carboxy-5-((S)-2-((1r,4S)-4-((4-(1-(6-(((((3R,5S)-1-((S)-2-(1-((S)-4-(4-chlorophenyl)-2,3,9-trimethyl-6H-thieno[3,2-f][1,2,4]triazolo[4,3-a][1,4]diazepin-6-yl)-2-oxo-6,9,12-trioxa-3-azatetradecan-14-amido)-3,3-dimethylbutanoyl)-5-((4-(4-methylthiazol-5-yl)benzyl)carbamoyl)pyrrolidin-3-yl)oxy)carbonyl)oxy)-6-methylheptyl)-1H-1,2,3-triazol-4-yl)butanamido)methyl)cyclohexane-1-carboxamido)-3-(naphthalen-2-yl)propanamido)pentyl)carbamoyl)-L-glutamic acid*

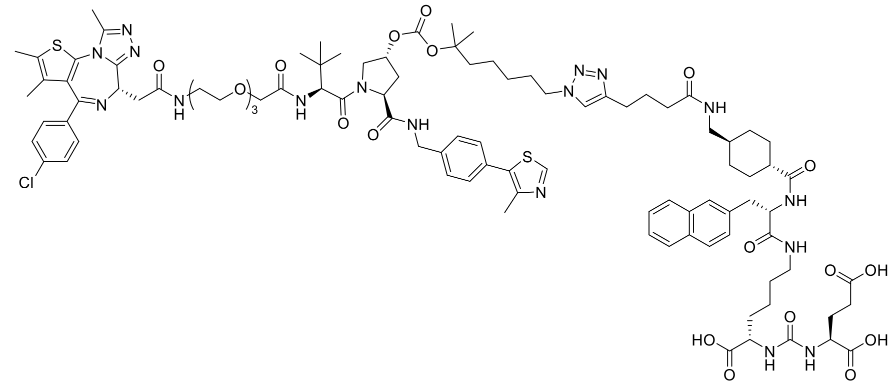

3b was prepared according to the general procedure A, from **S24** (10.2 mg, 8.5 µmol) to afford **3b** as a white solid (7.7 mg, 4.0 µmol, 46% yield). ^{1}H NMR (500 MHz, DMSO-d_6) δ = 8.97 (s, 1H), 8.63 (t, *J* = 6.1 Hz, 1H), 8.27 (t, *J* = 5.7 Hz, 1H), 7.99 – 7.92 (m, 2H), 7.86 – 7.81 (m, 2H), 7.80 – 7.75 (m, 2H), 7.71 (t, *J* = 5.8 Hz, 1H), 7.68 (s, 1H), 7.49 – 7.37 (m, 12H), 6.38 (d, *J* = 7.9 Hz, 1H), 6.23 (br s, 1H), 5.14 (s, 1H), 4.55 – 4.36 (m, 5H), 4.31 – 4.23 (m, 3H), 4.08 – 3.90 (m, 5H), 3.84 (dd, *J* = 11.9, 4.0 Hz, 1H), 3.64 – 3.51 (m, 9H), 3.10 (dd, *J* = 13.6, 5.1 Hz, 1H), 3.00 (dq, *J* = 19.1, 6.5 Hz, 2H), 2.92 (dd, *J* = 13.6, 9.5 Hz, 1H), 2.84 (t, *J* = 6.3 Hz, 2H), 2.58 (s, 3H), 2.55 (t, *J* = 7.7 Hz, 2H), 2.43 (s, 3H), 2.40 (s, 3H), 2.32 – 2.23 (m, 2H), 2.22 – 2.14 (m, 1H), 2.14 – 2.03 (m, 4H), 1.85 – 1.73 (m, 5H), 1.71 – 1.54 (m, 10H), 1.51 – 1.40 (m, 2H), 1.37 (s, 3H), 1.36 (s, 3H), 1.35 – 1.13 (m, 10H), 1.07 – 0.92 (m, 10H), 0.85 – 0.73 (m, 2H), (Note: three OH protons not detectable, five proton signals overlapping with water); ^{13}C NMR (126 MHz, DMSO-d_6) δ = 174.9, 174.7, 174.3, 174.2, 171.6, 171.0, 171.0, 169.7, 169.2, 168.9, 163.0, 157.2, 155.1, 152.1, 151.5, 149.8, 147.7, 146.4, 139.2, 136.7, 135.8, 135.2, 132.9, 132.3, 131.7, 131.1, 130.7, 130.1 (2C), 129.8, 129.8, 129.5, 128.7 (2C), 128.4 (2C), 127.9, 127.5 (2C), 127.4 (2C), 127.3 (2C), 125.9, 125.3, 121.7, 84.2, 75.8, 70.4, 69.8, 69.6 (2C), 69.5, 69.2, 58.3, 55.9, 53.8, 53.7 (2C), 52.3, 52.3, 49.1, 44.6, 43.6, 41.7, 38.6, 38.3, 38.1, 37.5, 37.0, 35.2, 34.9, 34.8, 31.7, 29.7, 29.5 (2C), 28.8, 28.7, 28.3, 26.2, 26.1 (3C), 25.4, 25.3 (2C), 25.3, 24.7, 22.7, 22.6, 15.9, 14.0, 12.7, 11.3, (Note: two signals missing, likely due to overlapping with the solvent); HRMS (ESI), m/z: [M+2H]$^{2+}$ calcd for $C_{97}H_{128}O_{20}N_{17}ClS_2^{2+}$: 974.9320 found: 974.9326.

Compound 4a – *(((1S)-1-Carboxy-5-((2S)-2-(4-((4-(1-(2-((2-(((((3R,5S)-1-((S)-2-(1-((S)-4-(4-chlorophenyl)-2,3,9-trimethyl-6H-thieno[3,2-f][1,2,4]triazolo[4,3-a][1,4]diazepin-6-yl)-2-oxo-6,9,12-trioxa-3-azatetradecan-14-amido)-3,3-dimethylbutanoyl)-5-((4-(4-methylthiazol-5-yl)benzyl)carbamoyl)pyrrolidin-3-yl)oxy)carbonyl)thio)ethyl)disulfaneyl)ethyl)-1H-1,2,3-triazol-4-yl)butanamido)methyl)cyclohexane-1-carboxamido)-3-(naphthalen-2-yl)propanamido)pentyl)carbamoyl)-L-glutamic acid*

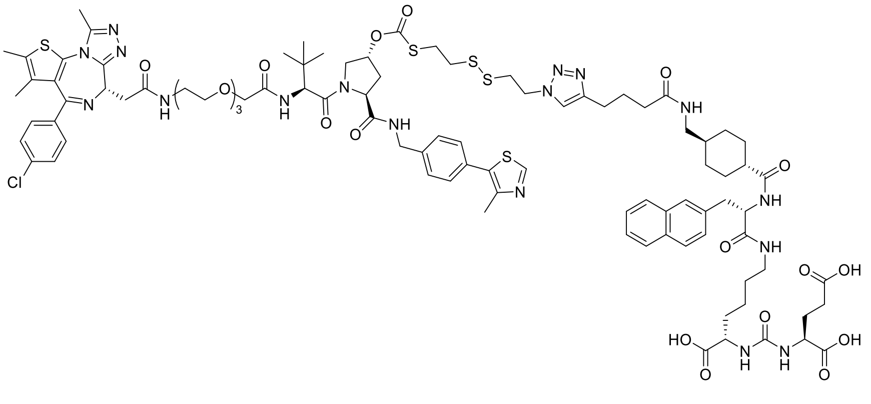

4a was prepared according to the general procedure A, from **S30** (7.1 mg, 5.8 µmol) to afford **4a** as a white solid (9.3 mg, 4.7 µmol, 81% yield). ^{1}H NMR (500 MHz, DMSO-d_6) δ = 8.97 (s, 1H), 8.63 (t, *J* = 6.1 Hz, 1H), 8.27 (t, *J* = 5.7 Hz, 1H), 8.00 – 7.93 (m, 2H), 7.89 (s, 1H), 7.83 (dd, *J* = 7.7, 1.7 Hz, 1H), 7.80 – 7.75 (m, 2H), 7.71 (t, *J* = 5.7 Hz, 1H), 7.68 (s, 1H), 7.48 – 7.38 (m, 12H), 6.39 (d, *J* = 8.0 Hz, 1H), 6.22 (d, *J* = 4.8 Hz, 1H), 5.46 (s, 1H), 4.62 – 4.57 (m, 2H), 4.55 – 4.43 (m, 4H), 4.39 (dd, *J* = 15.7, 6.3 Hz, 1H), 4.27 (dd, *J* = 15.7, 5.7 Hz, 1H), 4.05 – 3.93 (m, 5H), 3.88 (dd, *J* = 12.0, 4.0 Hz, 1H), 3.62 – 3.52 (m, 9H), 3.05 – 2.89 (m, 6H), 2.84 (t, *J* = 6.3 Hz, 2H), 2.60 – 2.55 (m, 5H), 2.43 (s, 3H), 2.40 (s, 3H), 2.34 – 2.25 (m, 2H), 2.21 – 2.02 (m, 5H), 1.87 – 1.72 (m, 3H), 1.69 – 1.54 (m, 8H), 1.51 – 1.39 (m, 2H), 1.36 – 1.27 (m, 2H), 1.27 – 1.16 (m, 4H), 1.06 – 0.92 (m, 10H), 0.85 – 0.73 (m, 2H), (Note: three OH protons not detectable, nine proton signals overlapping with water); ^{13}C NMR (126 MHz, DMSO-d_6) δ = 174.9, 174.7, 174.3 (2C), 171.6, 171.0, 170.9, 169.7, 169.4, 169.3, 169.0, 163.0, 157.2, 155.1, 151.5, 149.8, 147.8, 146.5, 139.2, 136.7, 135.8, 135.2, 132.9, 132.3, 131.7, 131.1, 130.7, 130.1 (2C), 129.8, 129.8, 129.6, 128.7 (2C), 128.4 (2C), 127.9, 127.5 (2C), 127.4 (2C), 127.3 (2C), 125.9, 125.3, 122.1, 76.9, 70.4, 69.8, 69.6 (2C), 69.5, 69.2, 58.2, 56.0, 53.8, 53.7, 53.5, 52.4, 52.4, 47.9, 44.7, 43.6, 41.7, 38.6, 38.3, 38.1, 37.5, 37.2, 37.0, 37.0, 35.1, 34.8, 34.8, 31.7, 30.1, 29.7, 29.5, 28.8, 28.7, 28.3, 26.1 (3C), 25.3, 24.7, 22.6, 15.9, 14.0, 12.7, 11.3, (Note: two signals missing, likely due to overlapping with the solvent); HRMS (ESI), m/z: $[M+2H]^{2+}$ calcd for $C_{93}H_{120}O_{19}N_{17}ClS_5^{2+}$: 986.8614 found: 986.8619.

Compound 4b – *(((1S)-1-Carboxy-5-((2S)-2-(4-((4-(1-(6-(((((3R,5S)-1-((S)-2-(1-((S)-4-(4-chlorophenyl)-2,3,9-trimethyl-6H-thieno[3,2-f][1,2,4]triazolo[4,3-a][1,4]diazepin-6-yl)-2-oxo-6,9,12-trioxa-3-azatetradecan-14-amido)-3,3-dimethylbutanoyl)-5-((4-(4-methylthiazol-5-yl)benzyl)carbamoyl)pyrrolidin-3-yl)oxy)carbonyl)thio)hexyl)-1H-1,2,3-triazol-4-yl)butanamido)methyl)cyclohexane-1-carboxamido)-3-(naphthalen-2-yl)propanamido)pentyl)carbamoyl)-L-glutamic acid*

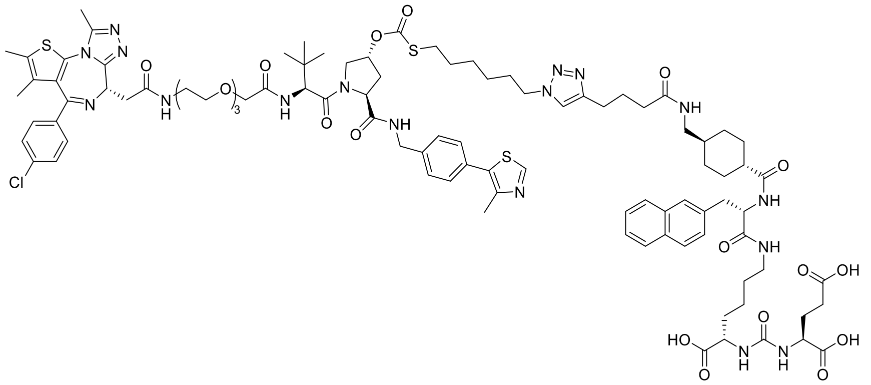

4b was prepared according to the general procedure A, from **S31** (10.1 mg, 8.5 µmol) to afford **4b** as a white solid (12.5 mg, 6.5 µmol, 76% yield). ^{1}H NMR (500 MHz, DMSO-d_6) δ = 8.97 (s, 1H), 8.63 (t, *J* = 6.0 Hz, 1H), 8.27 (t, *J* = 5.7 Hz, 1H), 8.01 – 7.93 (m, 2H), 7.85 – 7.81 (m, 2H), 7.80 – 7.75 (m, 2H), 7.71 (t, *J* = 5.8 Hz, 1H), 7.68 (s, 1H), 7.48 – 7.37 (m, 12H), 6.38 (d, *J* = 8.2 Hz, 1H), 6.23 (d, *J* = 6.0 Hz, 1H), 5.45 (s, 1H), 4.56 – 4.42 (m, 4H), 4.39 (dd, *J* = 15.7, 6.3 Hz, 1H), 4.31 – 4.24 (m, 3H), 4.09 – 3.92 (m, 5H), 3.88 (dd, *J* = 12.0, 4.1 Hz, 1H), 3.62 – 3.52 (m, 9H), 3.10 (dd, *J* = 13.6, 5.1 Hz, 1H), 3.01 (dp, *J* = 19.5, 6.4 Hz, 2H), 2.92 (dd, *J* = 13.6, 9.5 Hz, 1H), 2.87 – 2.79 (m, 4H), 2.58 (s, 3H), 2.55 (t, *J* = 7.7 Hz, 2H), 2.43 (s, 3H), 2.40 (s, 3H), 2.34 – 2.24 (m, 2H), 2.21 – 2.13 (m, 2H), 2.13 – 2.03 (m, 3H), 1.86 – 1.73 (m, 5H), 1.69 – 1.51 (m, 10H), 1.50 – 1.39 (m, 2H), 1.36 – 1.28 (m, 4H), 1.27 – 1.13 (m, 6H), 1.06 – 0.97 (m, 1H), 0.95 (s, 9H), 0.85 – 0.73 (m, 2H), (Note: three OH protons not detectable, five proton signals overlapping with water); ^{13}C NMR (126 MHz, DMSO-d_6) δ = 174.9, 174.7, 174.2, 174.2, 171.6, 171.0, 170.9, 169.9, 169.7, 169.2, 168.9, 163.0, 157.2, 155.1, 151.5, 149.8, 147.8, 146.4, 139.2, 136.7, 135.8, 135.2, 132.9, 132.3, 131.7, 131.1, 130.7, 130.1 (2C), 129.8, 129.8, 129.5, 128.7 (2C), 128.4 (2C), 127.9, 127.5 (2C), 127.4 (2C), 127.3 (2C), 125.9, 125.3, 121.7, 76.5, 70.4, 69.8, 69.6 (2C), 69.5, 69.2, 58.3, 56.0, 53.8, 53.7, 53.5, 52.3, 52.3, 49.0, 44.6, 43.6, 41.7, 38.6, 38.3, 38.1, 37.5, 37.0, 35.2, 34.8 (2C), 31.6, 30.3, 29.7, 29.5 (2C), 29.1, 28.8, 28.7, 28.3, 27.3, 26.1 (3C), 25.3, 25.3, 24.7, 22.6, 15.9, 14.0, 12.7, 11.3, (Note: two signals missing, likely due to overlapping with the solvent); HRMS (ESI), m/z: $[M+2H]^{2+}$ calcd for $C_{95}H_{124}O_{19}N_{17}ClS_3^{2+}$: 968.9050 found: 968.9052.

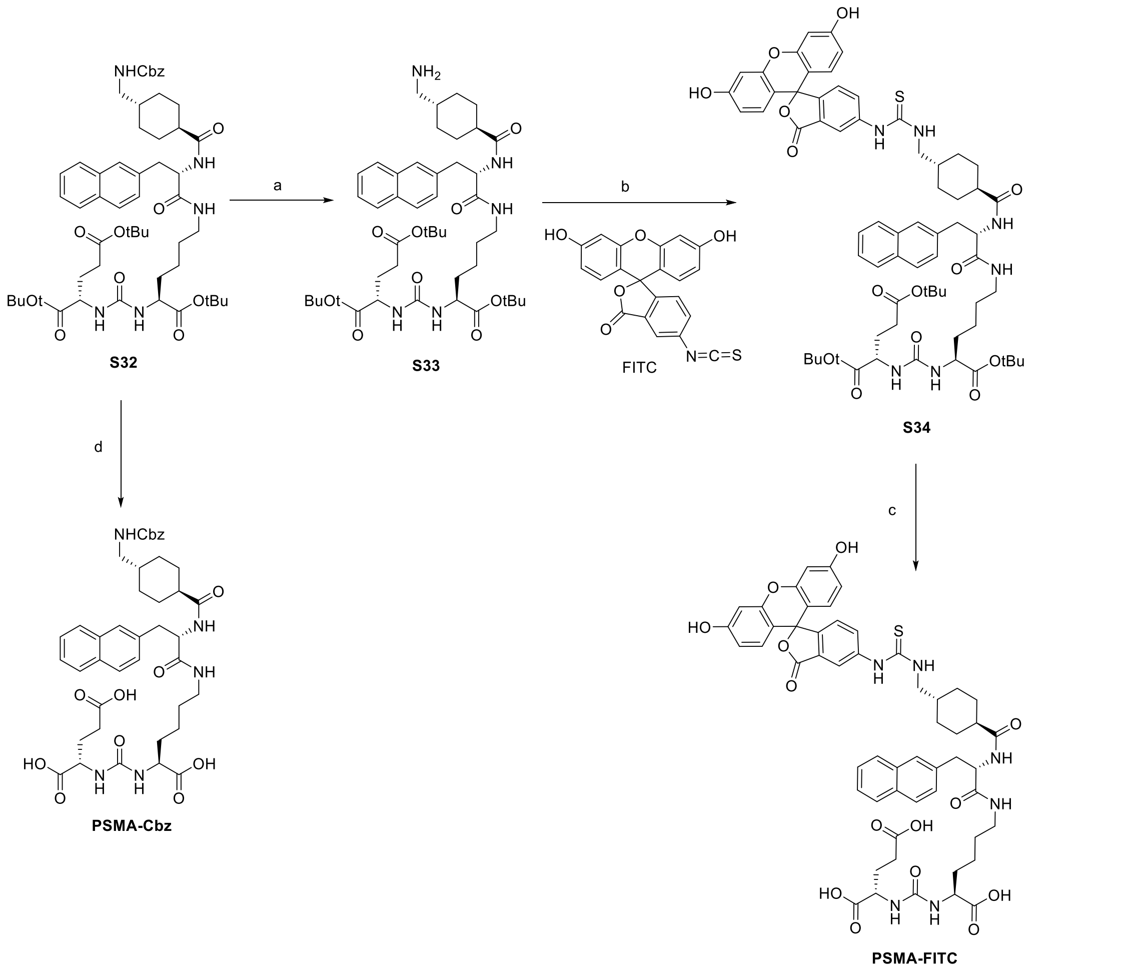

Scheme S13 Synthetic route to obtain **PSMA-FITC** and a competing non-fluorescent ligand **PSMA-Cbz**. a) NH_4HCO_2 (45 equiv), Pd/C (12 mol%), MeOH/THF (1:1; 0.05 M), 31 h, 58%; b) FITC (1 equiv), DIPEA (1.5 equiv), DMF (0.1 M), 20 °C, darkness, 2 h, 75%; c) TFA (500 equiv), CH_2Cl_2 (0.01M), 0 to 20 °C, darkness, 4 h, 31%; d) TFA (500 equiv), CH_2Cl_2 (0.03 M)), 0 to 20 °C, 1.5 h, 20%.

***Compound S32** – di-tert-butyl (((S)-6-((S)-2-((1r,4S)-4-((((benzyloxy)carbonyl)amino)methyl)cyclo-hexane-1-carboxamido)-3-(naphthalen-2-yl)propanamido)-1-(tert-butoxy)-1-oxohexan-2-yl)carbamoyl)-L-glutamate*

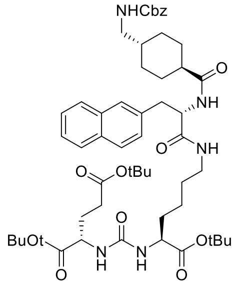

S32 was synthesised according to a previously reported procedure and the spectral data matched with previously published data.[18]

Compound S33 – *di-tert-butyl (((S)-6-((S)-2-((1r,4S)-4-(aminomethyl)cyclohexane-1-carboxamido)-3-(naphthalen-2-yl)propanamido)-1-(tert-butoxy)-1-oxohexan-2-yl)carbamoyl)-L-glutamate*

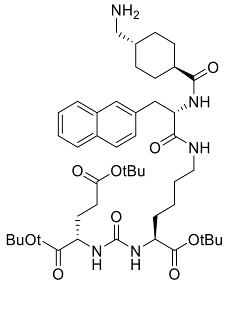

S33 was synthesised similarly to a previously reported procedure[18]: In an oven dried flask and under a N_2 environment, **S32** (60 mg, 0.063 mmol) was dissolved in MeOH (0.6 mL) and THF (0.6 mL). NH_4HCO_2 (59.2 mg, 0.939 mmol) was added in one portion. Upon the dissolution of most of the solid, Pd/C (5%; 8.66 mg, 0.004 mmol) was added in one portion, upon which, the reaction mixture was heated to 50 °C for 10 minutes and then left to stir at 20 °C for 7 h. The reaction mixture was re-supplemented with Pd/C (5%; 8.66 mg, 0.004 mmol) and NH_4HCO_2 (59.2 mg, 0.939 mmol), heated to 50 °C for 10 minutes, and allowed to stir at 20 °C for 18 h. An additional re-supplement of NH_4HCO_2 (59.2 mg, 0.939 mmol) with heating to 50 °C for 10 minutes was carried out and the reaction was allowed to stir at 20 °C for another 6 h. The mixture was filtered over celite, and the filtrate concentrated under reduced pressure. The crude material was purified by flash column chromatography (amino derivatised silica; CH_2Cl_2:MeOH = 99:1 to 90:10) to afford the product **S33** as a colourless waxy solid (30 mg, 0.036 mmol, 58% yield). The spectral data matched with previously published data.[18]

Compound S34 – *di-tert-butyl (((S)-1-(tert-butoxy)-6-((S)-2-((1r,4S)-4-((3-(3',6'-dihydroxy-3-oxo-3H-spiro[isobenzofuran-1,9'-xanthen]-5-yl)thioureido)methyl)cyclohexane-1-carboxamido)-3-(naphthalen-2-yl)propanamido)-1-oxohexan-2-yl)carbamoyl)-L-glutamate*

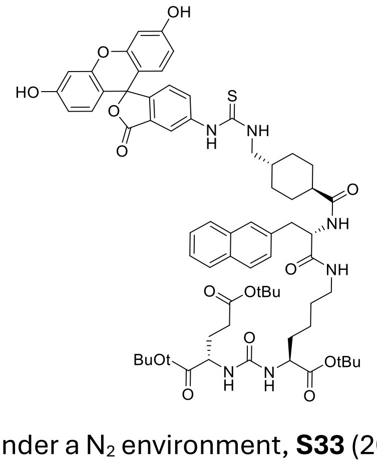

In an oven-dried amber vial and under a N_2 environment, **S33** (20 mg, 0.024 mmol) was dissolved in anhydrous DMF (0.24 mL). Dry DIPEA (6.4 µl, 0.036 mmol) and Fluorescein isothiocyanate Isomer I (90% purity; 10.5 mg, 0.024 mmol) were added. The reaction was allowed to stir at 20 °C for 2 h after which the vial was stored at -20 °C for 18 h. The reaction mixture was concentrated under reduced pressure and the crude material was purified in the dark by preparative TLC (EtOAc:DCM= 1:1 with 6% MEOH and 0.5% DMF) to afford the desired compound **S34** as an orange solid (22 mg, 0.18 mmol, 75% yield). ^{1}H NMR (400 MHz, Methanol-d_4) δ 8.11 (s, 1H), 7.85 – 7.72 (m, 4H), 7.69 (d, J = 1.7 Hz, 1H), 7.49 – 7.34 (m, 3H), 7.15 (d, J = 8.3 Hz, 1H), 6.76 – 6.63 (m, 4H), 6.55 (dd, J = 8.7, 2.4 Hz, 2H), 4.66 (dd, J = 8.6, 6.6 Hz, 1H), 4.19 (dd, J = 8.7, 5.0 Hz, 1H), 4.06 (dd, J = 8.4, 4.9 Hz, 1H), 3.53 – 3.44 (m, 2H), 3.25 (dd, J = 13.7, 6.7 Hz, 1H), 3.18 – 3.02 (m, 3H), 2.40 – 2.26 (m, 2H), 2.25 – 2.13 (m, 1H), 2.10 – 1.96 (m, 1H), 1.92 – 1.73 (m, 4H), 1.72 – 1.56 (m, 3H), 1.55 – 1.19 (m, 34H), 1.04 (dq, J = 12.8, 10.2, 9.5 Hz, 2H), (Note: eight OH/NH signals missing

due to proton-deuterium exchange); ^{13}C NMR (126 MHz, Methanol-d_4) δ 178.8, 174.0, 173.8, 173.7, 173.6, 171.2, 160.0, 154.3, 142.5, 136.0, 134.9, 133.9, 131.7, 130.4, 129.0, 129.0, 128.6, 128.6, 128.5, 127.1, 126.6, 125.8, 119.7, 113.7, 111.6, 103.5, 82.9, 82.5, 81.8, 56.0, 54.8, 54.2, 51.7, 45.9, 39.8, 39.3, 38.3, 32.8, 32.5, 31.1, 31.0, 30.3, 29.8, 29.6, 29.1, 28.4 (3C), 28.3 (3C), 28.3 (3C), 23.5, (Note: many signals broad and eleven signals missing in the aromatic region due to the possible ring opening and closing of the FITC fluorophore; pentane and ethanol-d_6 impurities present). HRMS (ESI), m/z: $[M+H]^+$ calcd for $C_{66}H_{81}O_{14}N_6S^+$: 1213.5526 found: 1213.5535.

Compound PSMA-FITC – *(((S)-1-carboxy-5-((S)-2-((1r,4S)-4-((3-(3',6'-dihydroxy-3-oxo-3H-spiro[isobenzofuran-1,9'-xanthen]-5-yl)thioureido)methyl)cyclohexane-1-carboxamido)-3-(naphthalen-2-yl)propanamido)pentyl)carbamoyl)-L-glutamic acid*

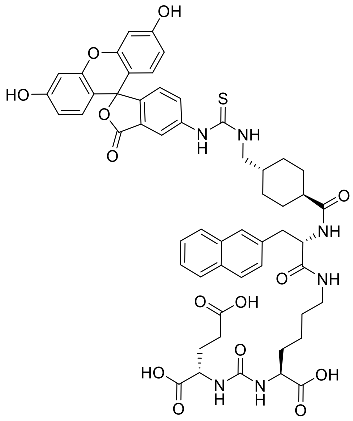

In an oven-dried flask and under a N_2 environment, **S34** (13 mg, 0.011 mmol) was suspended in anhydrous CH_2Cl_2 (0.8 mL) and the reaction mixture was cooled to 0 °C and covered from light with foil. TFA (0.398 ml, 5.36 mmol) was slowly added. The reaction mixture was followed by reverse phase TLC and allowed to stir at 0 °C for 2 h. It was allowed to warm up and stirred at 20 °C for 2 h, upon which toluene (3 mL) was added, and the solution was concentrated under reduced pressure in the darkness (using the rotary evaporator bath at no higher than 20 °C). The crude material was diluted with PBS and basified with NaOH (1M) to pH 10 to dissolve the material and then neutralised with HCl (1M). The solution was filtered and purified by reverse-phase HPLC using a Shimadzu Nexera system, equipped with an Agilent Zorbax 300SB-C18 semi-preparative column (9.4 x 250 mm 5-micron) using 20 to 70% MeCN in water buffered with 0.1% TFA as eluent (8 mL/min) to afford the desired product **PSMA-FITC** as an orange solid (3.5 mg, 0.0034 mmol, 31% yield). ^{1}H NMR (500 MHz, DMSO-d_6) δ 12.60 (s, 1H), 12.48 (s, 1H), 12.14 (s, 1H), 10.11 (s, 2H), 9.82 (s, 1H), 8.29 (s, 1H), 8.04 (s, 1H), 7.98 – 7.92 (m, 2H), 7.84 (dd, *J* = 7.8, 0.9 Hz, 1H), 7.83 – 7.76 (m, 2H), 7.72 (d, *J* = 8.2 Hz, 1H), 7.69 (d, *J* = 1.6 Hz, 1H), 7.45 (dqd, *J* = 8.3, 6.8, 1.5 Hz, 2H), 7.40 (dd, *J* = 8.5, 1.7 Hz, 1H), 7.16 (d, *J* = 8.3 Hz, 1H), 6.67 (d, *J* = 2.3 Hz, 2H), 6.60 (d, *J* = 8.7 Hz, 2H), 6.56 (dd, *J* = 8.7, 2.3 Hz, 2H), 6.32 (d, *J* = 8.3 Hz, 1H), 6.28 (d, *J* = 8.3 Hz, 1H), 4.54 (td, *J* = 8.9, 5.2 Hz, 1H), 4.10 (td, *J* = 8.3, 5.4 Hz, 1H), 4.02 (td, *J* = 8.1, 5.3 Hz, 1H), 3.14 – 3.02 (m, 2H), 2.95 (ddd, *J* = 23.0, 13.4, 7.8 Hz, 2H), 2.32 – 2.16 (m, 2H), 2.16 – 2.06 (m, 1H), 1.97 – 1.87 (m, 1H), 1.81 – 1.65 (m, 4H), 1.60 (h, *J* = 7.6 Hz, 1H), 1.56 – 1.42 (m, 3H), 1.33 (p, *J* = 7.1 Hz, 2H), 1.24 (tt, *J* = 8.1, 3.3 Hz, 3H), 1.14 – 1.02 (m, 1H), 0.92 (p, *J* = 12.8 Hz, 2H), (Note: one signal for two protons missing due to overlapping with solvent); ^{13}C NMR (126 MHz, DMSO-d_6) δ 174.8, 174.5, 174.2, 173.7, 171.0, 168.5, 159.4, 157.3, 151.9, 135.8, 132.9, 131.8, 129.0, 129.0, 127.9, 127.4, 127.4, 127.3, 127.3, 125.9, 125.3, 112.6, 109.7, 102.2, 53.6, 52.2, 51.5, 43.6, 38.4, 38.2, 36.4, 31.7, 29.9 (2C), 29.7, 29.6, 28.7 (2C), 28.3, 27.5, 22.5 (Note: thirteen high-field signals missing in due to the possible ring opening and closing of the FITC fluorophore); HRMS (ESI), m/z: $[M-H]^-$ calcd for $C_{54}H_{55}O_{14}N_6S$: 1043.3503 found: 1043.3480.

Compound PSMA-Cbz – *(((S)-5-((S)-2-((1r,4S)-4-((((benzyloxy)carbonyl)amino)methyl)cyclohexane-1-carboxamido)-3-(naphthalen-2-yl)propanamido)-1-carboxypentyl)carbamoyl)-L-glutamic acid*

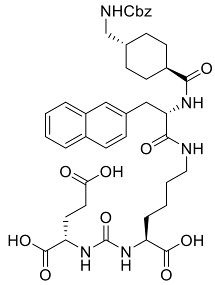

In an oven-dried flask and under a N_2 environment, **S32** (15 mg, 0.016 mmol) was dissolved in anhydrous CH_2Cl_2 (0.6 mL) and the solution was cooled to 0 °C. TFA (0.58 ml, 7.83 mmol) was slowly added. The reaction mixture was allowed to warm up to 20 °C and stirred for 30 min. Toluene (3 mL) was added and the reaction mixture was concentrated under reduced pressure (using the rotary evaporator bath at no higher than 20 °C). The residue was dissolved in anhydrous CH_2Cl_2 (0.6 mL) and the same process was repeated twice. The dry residue was suspended in acetone and filtered to afford the desired compound **PSMA-Cbz** as a white solid (5 mg, 0.0032 mmol, 20% yield). ^{1}H NMR (500 MHz, DMSO-d_6) δ 12.47 (s, 3H), 7.98 – 7.91 (m, 2H), 7.85 (dd, *J* = 7.6, 1.8 Hz, 1H), 7.81 – 7.76 (m, 2H), 7.68 (d, *J* = 1.6 Hz, 1H), 7.49 – 7.41 (m, 2H), 7.41 – 7.28 (m, 6H), 7.21 (t, *J* = 5.9 Hz, 1H), 6.34 (d, *J* = 8.1 Hz, 1H), 6.26 (d, *J* = 7.8 Hz, 1H), 4.99 (s, 2H), 4.52 (td, *J* = 9.0, 5.2 Hz, 1H), 4.06 (q, *J* = 7.1, 6.5 Hz, 1H), 4.00 (td, *J* = 7.9, 5.0 Hz, 1H), 3.10 (dd, *J* = 13.6, 5.1 Hz, 1H), 3.07 – 2.96 (m, 2H), 2.96 – 2.87 (m, 1H), 2.80 (t, *J* = 6.2 Hz, 2H), 2.23 (qt, *J* = 14.7, 7.2 Hz, 2H), 2.08 – 2.02 (m, 1H), 1.84 – 1.70 (m, 1H), 1.71 – 1.54 (m, 4H), 1.52 – 1.40 (m, 2H), 1.32 (p, *J* = 7.0 Hz, 2H), 1.28 – 1.13 (m, 5H), 1.02 (qd, *J* = 12.9, 3.7 Hz, 1H), 0.80 (qt, *J* = 14.0, 8.2 Hz, 2H). (Note: Acetone impurity present); ^{13}C NMR (126 MHz, DMSO-d_6) δ 174.9, 174.6, 174.2, 174.0, 171.0, 157.2, 156.2, 137.3, 135.8, 132.9, 131.8, 128.3 (2C), 127.9, 127.7, 127.7 (2C), 127.4, 127.4, 127.3, 127.3, 125.9, 125.3, 65.1, 53.7, 52.3, 52.2, 46.5, 43.6, 38.3, 38.2, 37.2, 31.6, 30.7, 29.5, 29.5, 29.4, 28.7, 28.7, 28.3, 22.5; HRMS (ESI), m/z: $[M+H]^+$ calcd for $C_{41}H_{52}O_{11}N_5^+$: 790.3658 found: 790.3662.

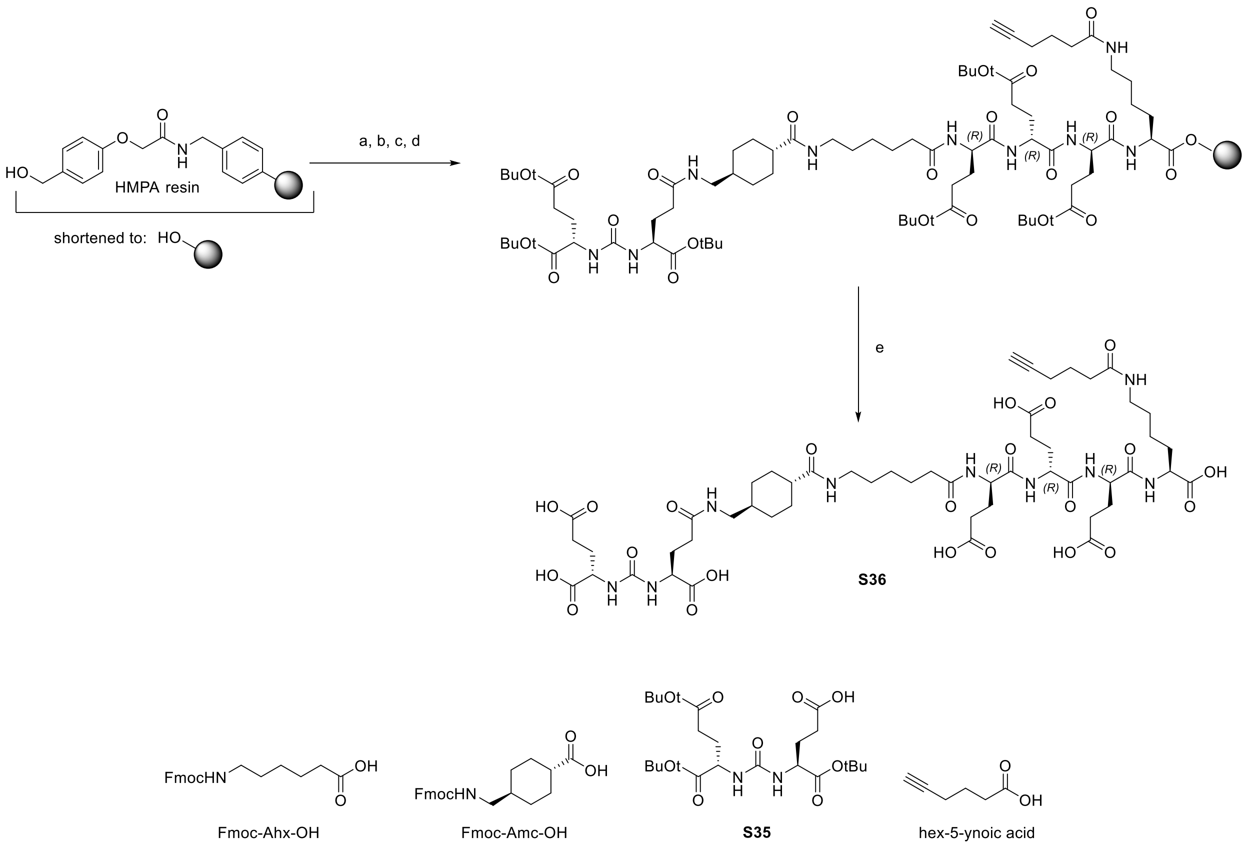

Scheme S14 The synthesis of the key PSMA-targeting alkyne intermediate **S36**. a) First coupling with DIC, DMAP: Lys(alloc)-OH; b) Standard fmoc-strategy amide couplings with HATU, DIPEA: 3x Fmoc-(D)Glu(OtBu)-OH, Fmoc-Ahx-OH, Fmoc-Amc-OH, **S35**; c) Alloc-deprotection; d) Standard fmoc-strategy amide coupling: hex-5-ynoic acid; e) Cleavage and global deprotection; overall yield 38%.

***Compound S36** – (((S)-1-carboxy-4-((((1R,4S)-4-((6-(((R)-4-carboxy-1-(((R)-4-carboxy-1-(((R)-4-carboxy-1-(((S)-1-carboxy-5-(hex-5-ynamido)pentyl)amino)-1-oxobutan-2-yl)amino)-1-oxobutan-2-yl)amino)-1-oxobutan-2-yl)amino)-6-oxohexyl)carbamoyl)cyclohexyl)methyl)amino)-4-oxobutyl)carbamoyl)-L-glutamic acid*

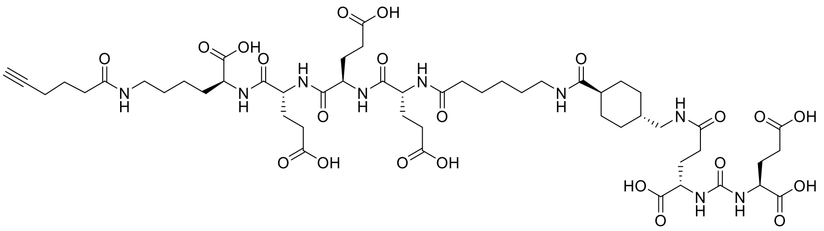

S36 was prepared according to the solid phase peptide synthesis (SPPS) general procedures to incorporate Fmoc-Lys(Alloc)-OH, 3 x Fmoc-(D)Glu(OtBu)-OH, Fmoc-Ahx-OH, Fmoc-Amc-OH, **S35** (prepared according to the previously reported procedure)[28], hex-5-ynoic acid (Scheme S14). After the cleavage, global deprotection, and purification **S36** was obtained as a white solid (130 mg, 0.110 mmol, 38% yield). ^{1}H NMR (500 MHz, DMSO-d_6) δ 12.30 (s, 7H), 8.06 (d, *J* = 7.8 Hz, 1H), 8.01 – 7.95 (m, 2H), 7.94 (d, *J* = 7.9 Hz, 1H), 7.82 – 7.72 (m, 2H), 7.62 (t, *J* = 5.6 Hz, 1H), 6.34 (t, *J* = 8.0 Hz, 2H), 4.30 (td, *J* = 8.3, 5.2 Hz, 1H), 4.26 – 4.19 (m, 2H), 4.17 – 4.11 (m, 1H), 4.11 – 4.08 (m, 1H), 4.05 (td, *J* = 8.1, 5.1 Hz, 1H), 3.06 – 2.92 (m, 4H), 2.87 (t, *J* = 6.2 Hz, 2H), 2.76 (t, *J* = 2.7 Hz, 1H), 2.29 – 2.17 (m, 8H), 2.17 – 2.04 (m, 8H), 1.98 (tq, *J* = 9.9, 3.3 Hz, 1H), 1.94 – 1.82 (m, 5H), 1.81 – 1.52 (m, 13H), 1.47 (p, *J* = 7.7 Hz, 2H), 1.40 – 1.21 (m, 11H), 0.84 (qd, *J* = 13.3, 4.0 Hz, 2H); (Note: an isopropyl-derivative impurity present(< 2.5%)). ^{13}C NMR (126 MHz, DMSO-d_6) δ 174.92, 174.25, 174.14, 173.99, 173.97, 173.89, 173.74, 173.37, 172.54, 171.51, 171.25, 171.18, 170.98,

170.90, 157.27, 84.14, 71.44, 52.12, 51.95, 51.92, 51.90, 51.65 (2C), 44.75, 44.11, 38.22 (2C), 37.04, 35.11, 34.15, 31.64, 30.79, 30.21, 30.13, 30.07, 29.88, 29.70 (2C), 28.96, 28.83 (2C), 28.62, 28.42, 27.64, 27.55, 27.14, 27.11, 26.11, 24.96, 24.28, 22.73, 17.42, (Note: an isopropyl-derivative impurity present(< 2.5%)). HRMS (ESI), m/z: $[M+H]^+$ calcd for $C_{52}H_{80}O_{22}N_9^+$: 1182.54125 found: 1182.54131

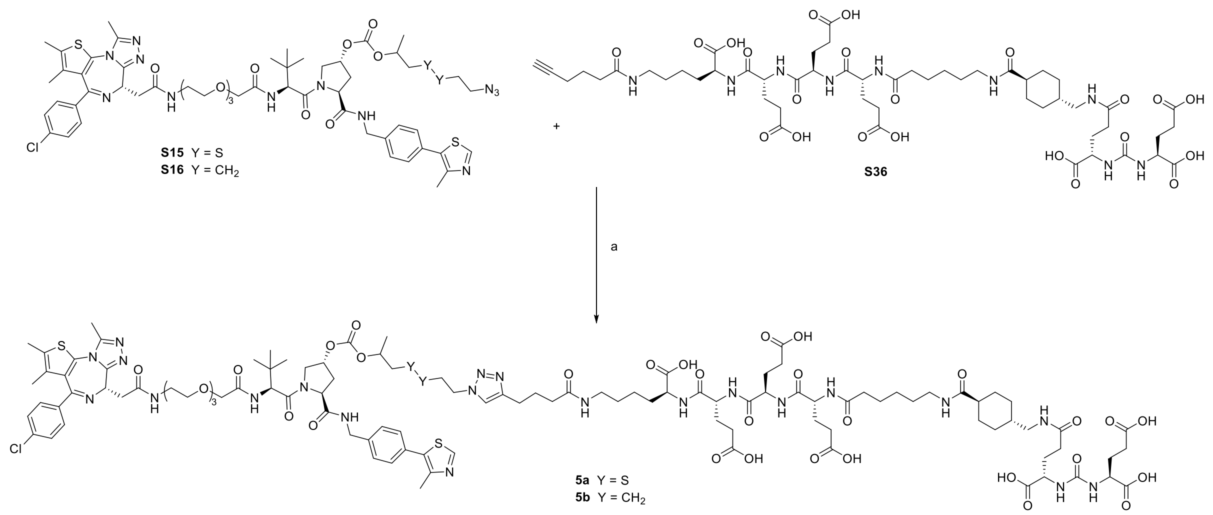

Scheme S15 The final CuAAC to form the highly negatively charged PCa-targeting SelecPROTACs **5a** and **5b**. a) **S36** (1.5 equiv), $CuSO_4 \cdot 5H_2O$ (3 equiv), sodium ascorbate (3 equiv), TBTA (0.2 equiv), NMP (0.0017 M), 20 °C, 16 h, 83-85%.

Compound 5a

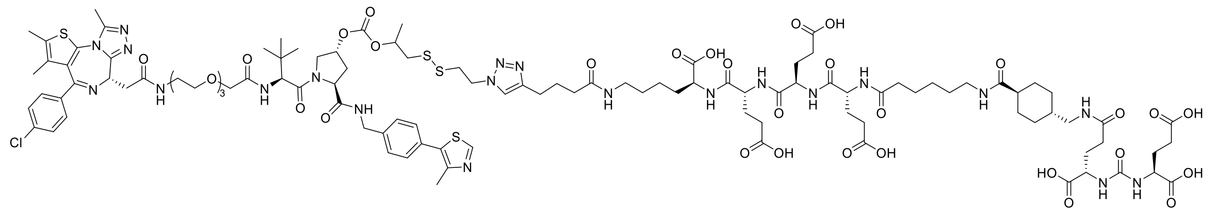

5a was prepared according to the general procedure B, from **S15** (9.8 mg, 8 μmol) to afford **5a** as a white solid (16 mg, 6.7 μmol, 83% yield). ^1H NMR (500 MHz, DMSO-d_6) δ 12.31 (s, 7H), 8.97 (s, 1H), 8.64 (dt, *J* = 16.2, 6.0 Hz, 1H), 8.27 (t, *J* = 5.7 Hz, 1H), 8.07 (d, *J* = 7.8 Hz, 1H), 7.98 (dd, *J* = 7.6, 4.0 Hz, 2H), 7.94 (d, *J* = 8.1 Hz, 1H), 7.88 (d, *J* = 3.2 Hz, 1H), 7.79 (t, *J* = 5.8 Hz, 1H), 7.74 (t, *J* = 5.6 Hz, 1H), 7.62 (t, *J* = 5.6 Hz, 1H), 7.50 – 7.37 (m, 9H), 6.34 (t, *J* = 8.0 Hz, 2H), 5.21 (q, *J* = 4.9 Hz, 1H), 4.88 (h, *J* = 6.3 Hz, 1H), 4.59 (q, *J* = 7.0 Hz, 2H), 4.53 – 4.35 (m, 4H), 4.34 – 4.18 (m, 4H), 4.18 – 3.92 (m, 7H), 3.89 – 3.81 (m, 1H), 3.08 – 2.91 (m, 6H), 2.87 (t, *J* = 6.3 Hz, 2H), 2.61 – 2.55 (m, 5H), 2.43 (s, 3H), 2.40 (s, 3H), 2.36 – 2.28 (m, 1H), 2.30 – 2.16 (m, 8H), 2.18 – 2.04 (m, 7H), 1.98 (tq, *J* = 9.4, 3.2 Hz, 1H), 1.96 – 1.81 (m, 5H), 1.83 – 1.64 (m, 12H), 1.61 (s, 4H), 1.46 (q, *J* = 7.8 Hz, 2H), 1.41 – 1.16 (m, 14H), 0.95 (d, *J* = 2.7 Hz, 9H), 0.84 (qd, *J* = 13.4, 4.1 Hz, 2H), (Note: fifteen proton signals overlapping with water; CH_2Cl_2 impurity present); ^{13}C NMR (126 MHz, DMSO-d_6) δ 174.9, 174.2, 174.1, 174.0, 174.0, 173.9, 173.7, 173.4, 172.5, 171.6, 171.5, 171.2, 171.0, 170.9, 170.9, 169.7, 169.3, 169.2, 169.0, 168.9, 163.0, 157.3, 155.1, 153.3, 153.2, 151.5, 149.8, 147.7, 146.6, 146.5, 139.2, 136.7, 135.2, 132.2, 131.1, 130.7, 130.2 (2C), 129.8, 129.8, 129.6, 128.7 (2C), 128.5 (2C), 127.5 (2C), 122.1, 76.8, 73.3, 73.2, 70.4, 70.4, 69.8, 69.6 (2C), 69.5, 69.5, 69.2, 58.3, 56.1, 56.0, 53.8, 53.6, 53.6, 53.6, 52.1, 51.9, 51.9, 51.6 (2C), 47.9, 44.7, 44.1, 43.0, 42.8, 41.7, 38.6, 38.2, 37.5, 37.1, 37.0, 35.2, 35.1, 35.1, 34.9, 34.8, 34.8, 34.7, 31.6, 30.8, 30.7, 30.2, 30.1, 30.1, 29.9, 29.7 (2C), 29.0, 28.8 (2C), 28.7, 28.4, 27.6, 27.5, 27.1, 27.1, 26.1 (3C), 25.2, 25.2, 25.0, 24.7, 24.7, 22.7, 18.8, 18.6, 15.9, 14.1, 12.7, 11.3, (Note: due to the presence of two diastereomers, several

peaks are duplicated, but not all; CH_2Cl_2 impurity present); HRMS (ESI), m/z: $[M+2H]^{2+}$ calcd for $C_{107}H_{150}O_{32}N_{21}{}^{35}Cl^{32}S_4{}^{2+}$: 1201.96581 found: 1201.96484.

Compound 5b

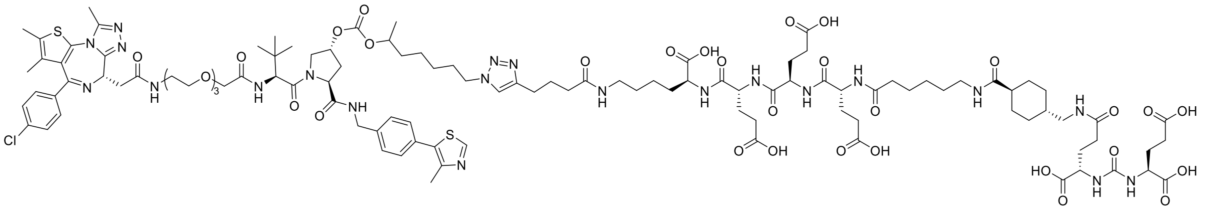

5b was prepared according to the general procedure A, from **S16** (11.9 mg, 10 μmol) to afford **5b** as a white solid (20 mg, 8.5 μmol, 85% yield). ^{1}H NMR (500 MHz, DMSO-d_6) δ 12.30 (s, 7H), 8.97 (s, 1H), 8.63 (q, *J* = 6.0 Hz, 1H), 8.27 (t, *J* = 5.7 Hz, 1H), 8.07 (d, *J* = 7.9 Hz, 1H), 7.98 (dd, *J* = 7.7, 4.1 Hz, 2H), 7.94 (d, *J* = 7.9 Hz, 1H), 7.83 (d, *J* = 2.1 Hz, 1H), 7.79 (t, *J* = 5.8 Hz, 1H), 7.74 (t, *J* = 5.8 Hz, 1H), 7.62 (t, *J* = 5.6 Hz, 1H), 7.49 – 7.37 (m, 9H), 6.34 (t, *J* = 8.0 Hz, 2H), 5.19 (q, *J* = 5.5 Hz, 1H), 4.65 (h, *J* = 6.2 Hz, 1H), 4.53 – 4.36 (m, 4H), 4.33 – 4.19 (m, 6H), 4.17 – 3.92 (m, 7H), 3.84 (dt, *J* = 11.9, 4.0 Hz, 1H), 3.00 (dq, *J* = 19.7, 6.4 Hz, 4H), 2.87 (t, *J* = 6.3 Hz, 2H), 2.60 – 2.53 (m, 5H), 2.43 (s, 3H), 2.40 (s, 3H), 2.34 – 2.28 (m, 1H), 2.27 – 2.18 (m, 8H), 2.17 – 2.05 (m, 7H), 1.99 (tt, *J* = 11.9, 3.3 Hz, 1H), 1.95 – 1.82 (m, 5H), 1.83 – 1.64 (m, 14H), 1.63 – 1.51 (m, 5H), 1.50 – 1.42 (m, 3H), 1.40 – 1.14 (m, 18H), 0.95 (s, 9H), 0.84 (qd, *J* = 13.8, 13.4, 3.9 Hz, 2H), (Note: thirteen proton signals overlapping with water); ^{13}C NMR (126 MHz, DMSO-d_6) δ 174.9, 174.2, 174.1, 174.0, 174.0, 173.9, 173.7, 173.4, 172.5, 171.6, 171.5, 171.2, 171.0, 170.9, 169.6, 169.2, 169.2, 168.9, 168.9, 163.0, 157.3, 155.1, 153.5, 153.5, 151.5, 149.9, 149.8, 147.7, 146.4, 139.2, 136.7, 135.2, 132.2, 131.1, 130.8, 130.7, 130.2 (2C), 129.8, 129.8, 129.6, 128.7 (2C), 128.4 (2C), 127.5 (2C), 121.7, 76.4, 75.1, 70.4, 70.4, 69.8, 69.6 (2C), 69.5, 69.5, 69.2, 58.3, 56.0, 53.8, 53.7, 52.1, 51.9, 51.9, 51.6 (2C), 49.1, 49.0, 44.7, 44.1, 41.7, 38.6, 38.2, 37.5, 37.0, 35.2, 35.1, 35.1, 34.9, 34.9, 34.9, 34.8, 34.7, 31.6, 30.8, 30.7, 30.2, 30.1, 30.1, 29.9, 29.7 (2C), 29.5, 29.0, 28.8 (2C), 28.7, 28.4, 27.6, 27.5, 27.1, 26.1 (3C), 25.7, 25.7, 25.2, 24.9, 24.7, 24.1, 24.1, 22.7, 19.5, 19.5, 15.9, 14.0, 12.7, 11.3 (Note: due to the presence of two diastereomers, several peaks are duplicated, but not all); HRMS (ESI), m/z: $[M+2H]^{2+}$ calcd for $C_{109}H_{154}O_{32}N_{21}{}^{35}Cl^{32}S_2{}^{2+}$: 1184.00939 found: 1184.01020.

5. ^{1}H and ^{13}C NMR Spectra

*Compound **S3**. ^{1}H NMR (400 MHz, Methylene Chloride-d_2) and ^{13}C NMR (101 MHz, Methylene Chloride-d_2)*

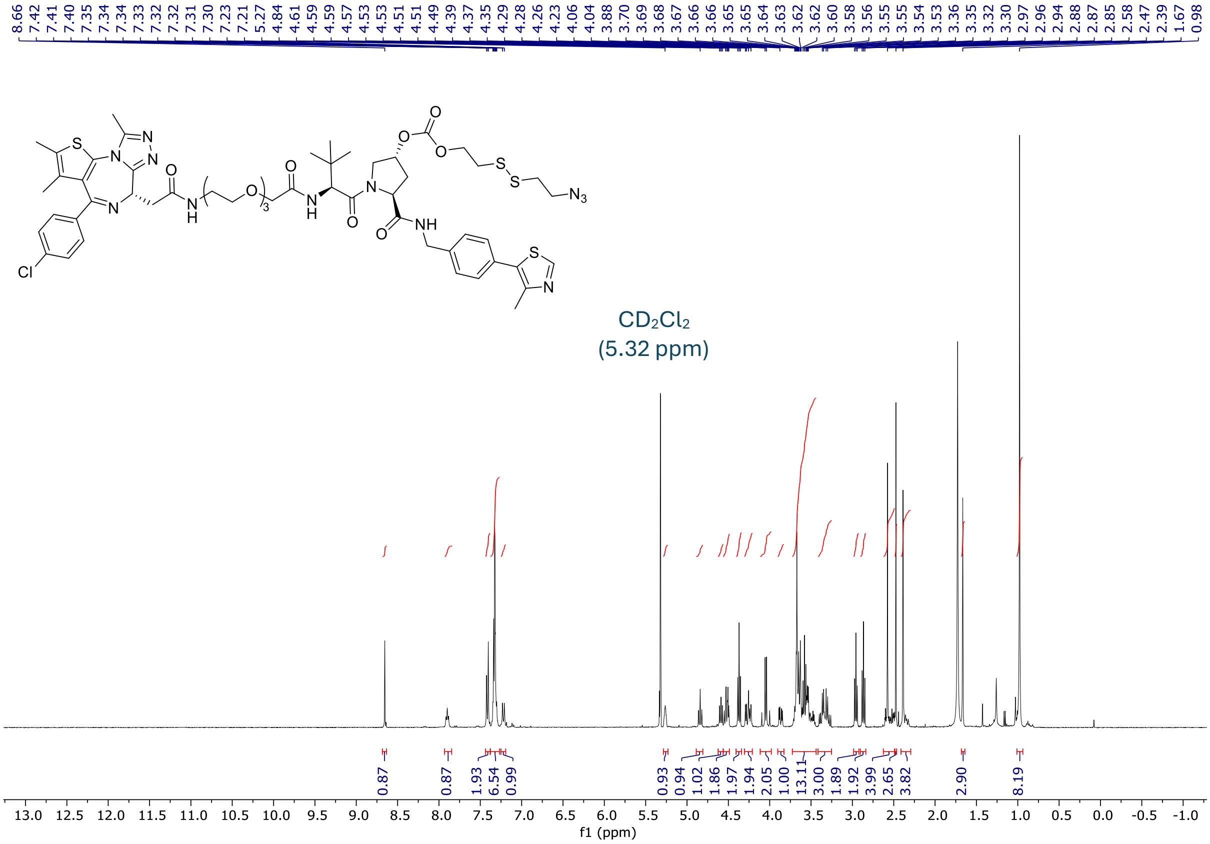

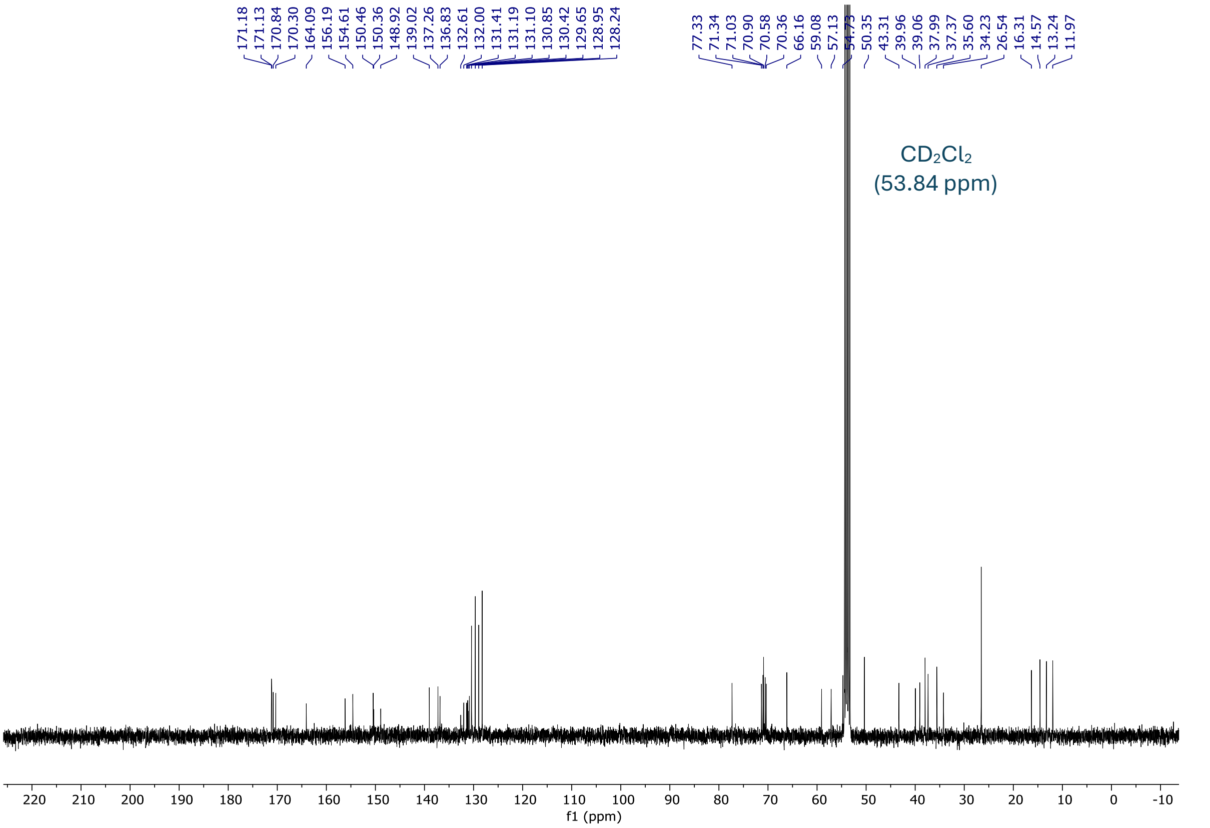

*Compound **S4**. ^{1}H NMR (500 MHz, Methylene Chloride-d_2) and ^{13}C NMR (126 MHz, Methylene Chloride-d_2)*

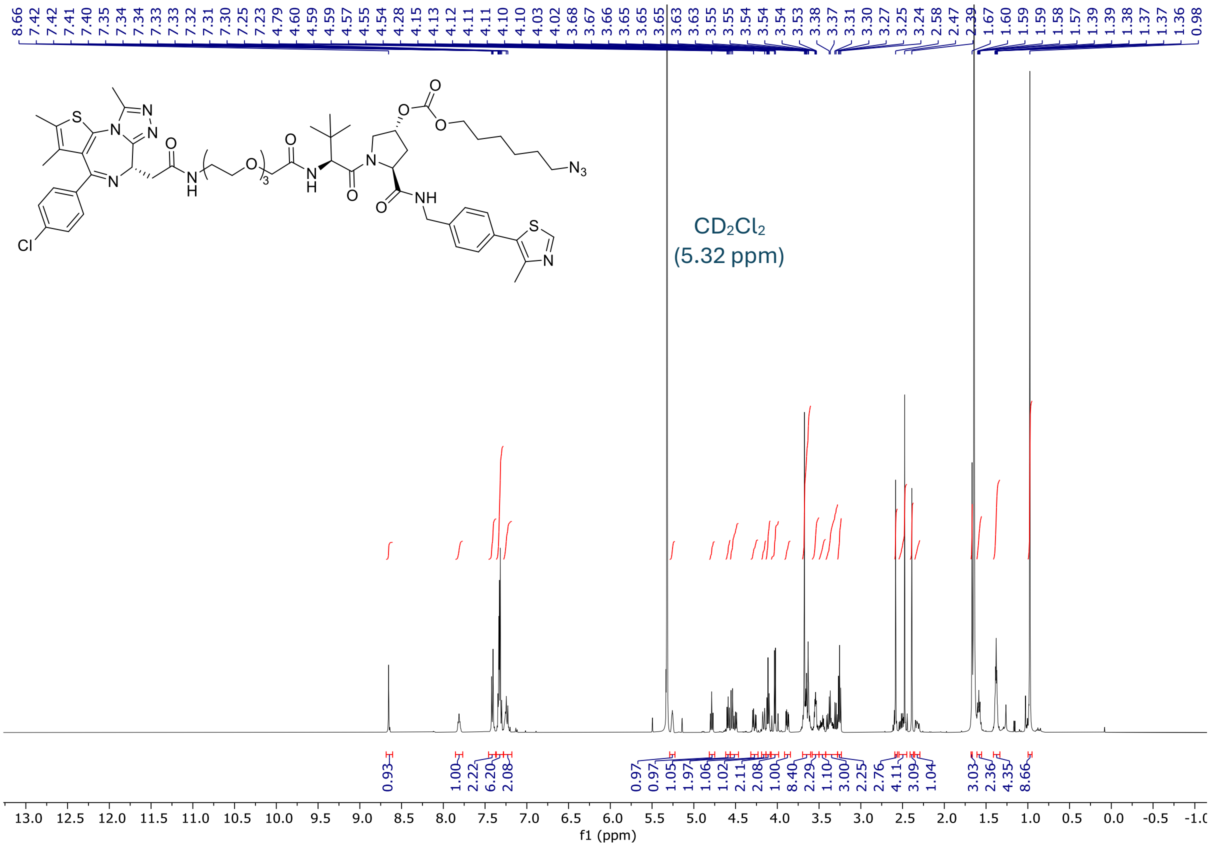

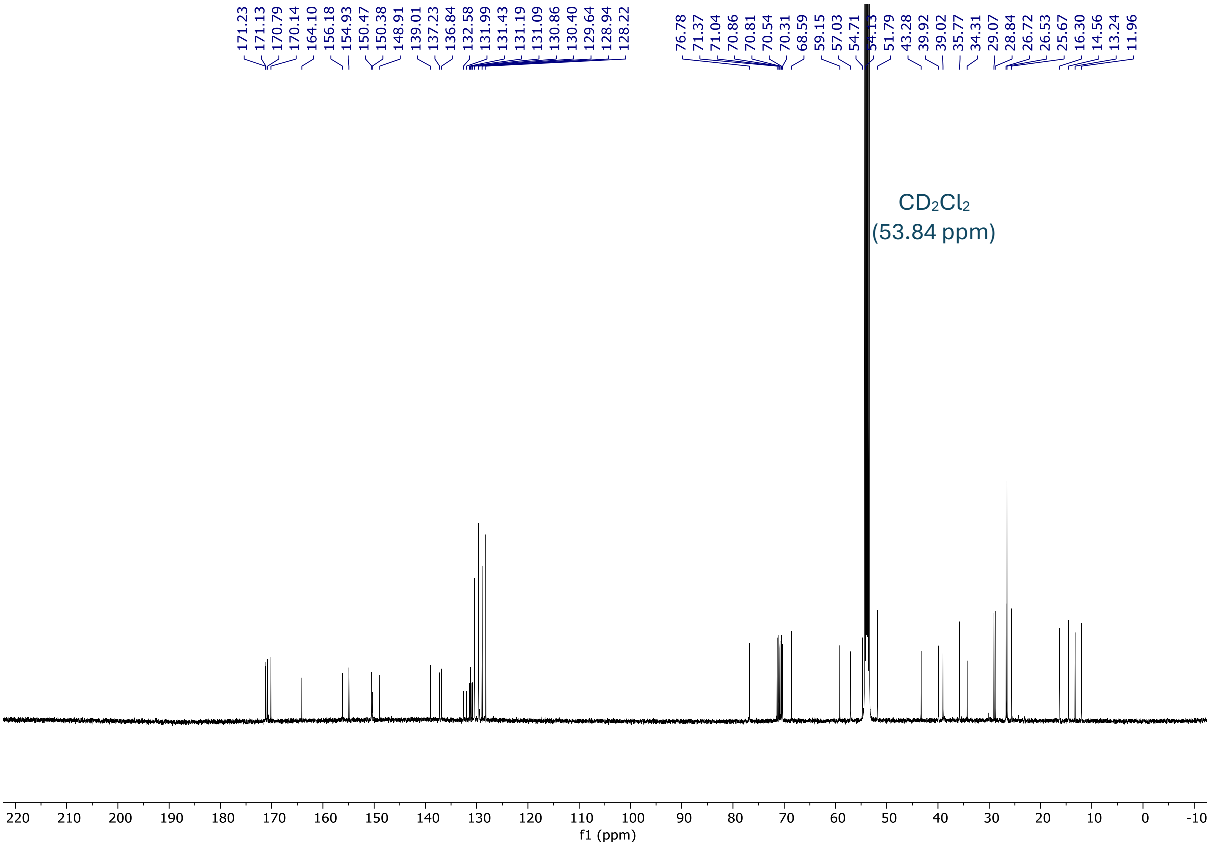

*Compound **S9**. ^{1}H NMR (400 MHz, Methylene Chloride-d_2) and ^{13}C NMR (101 MHz, Methylene Chloride-d_2)*

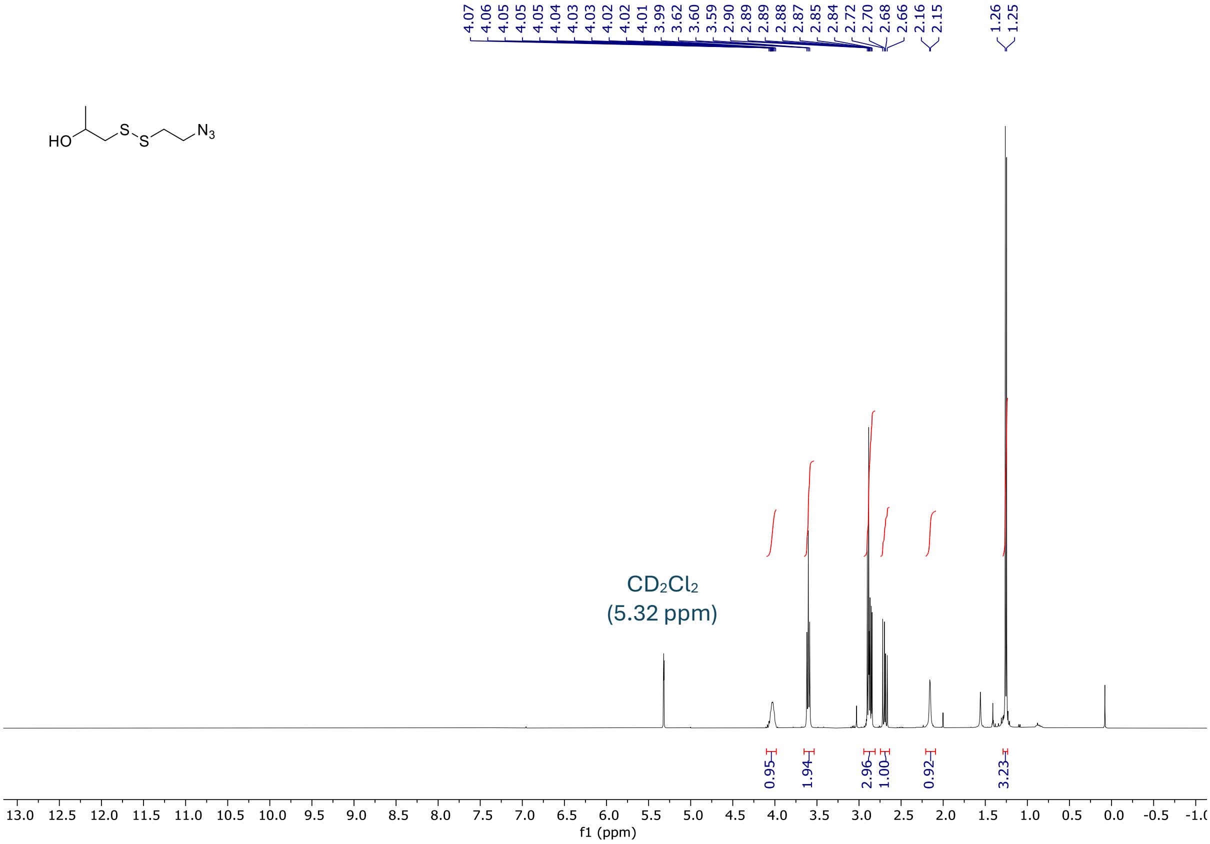

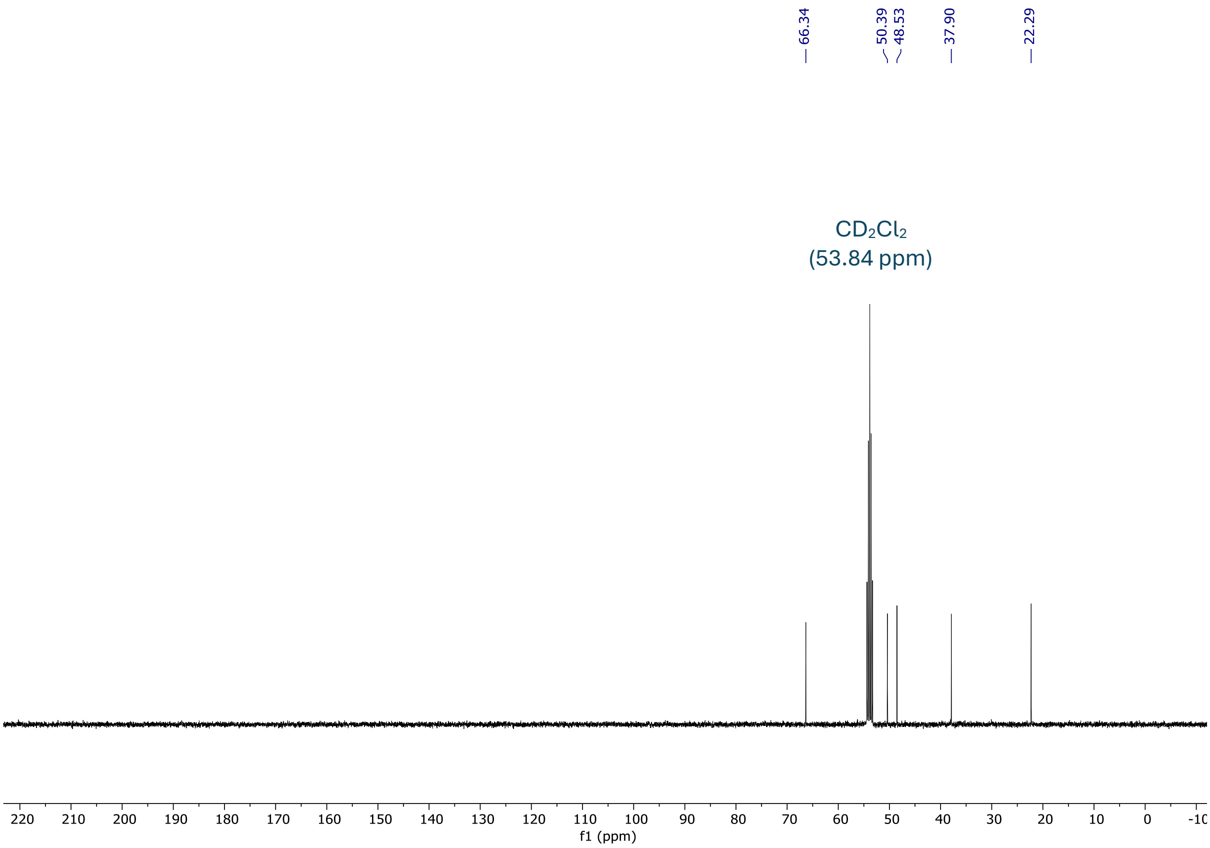

Compound ***S12****. ^{1}H NMR (500 MHz, Chloroform-d) and ^{13}C NMR (126 MHz, Chloroform-d)*

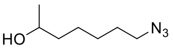

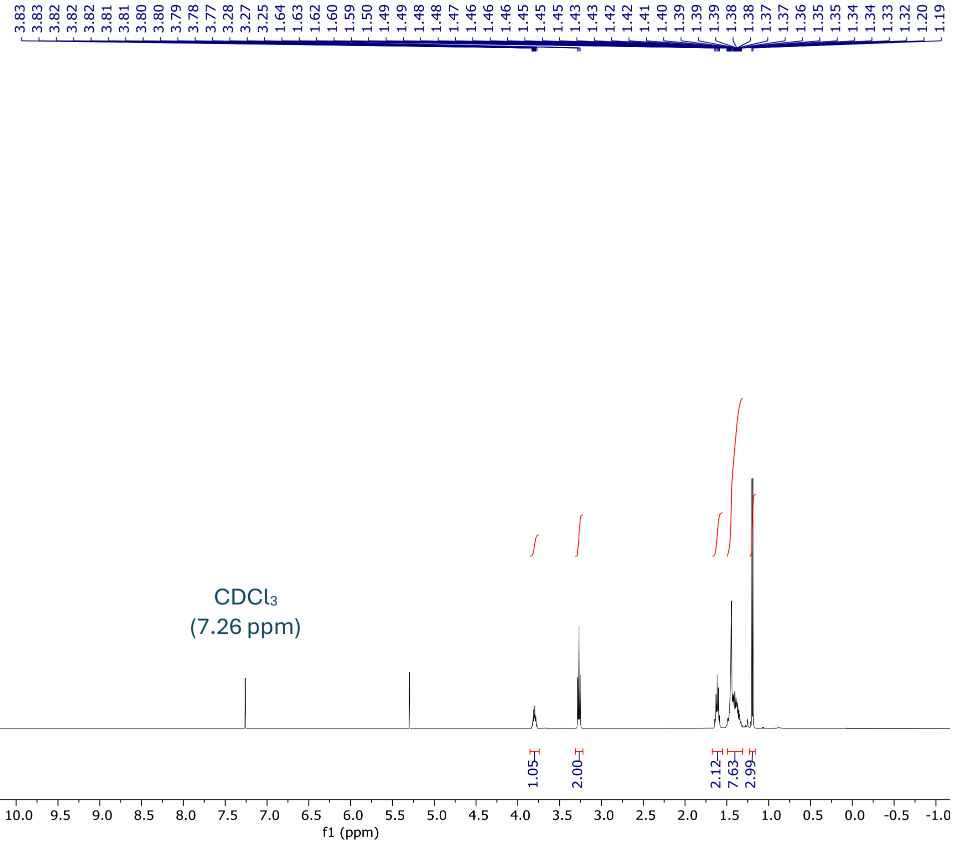

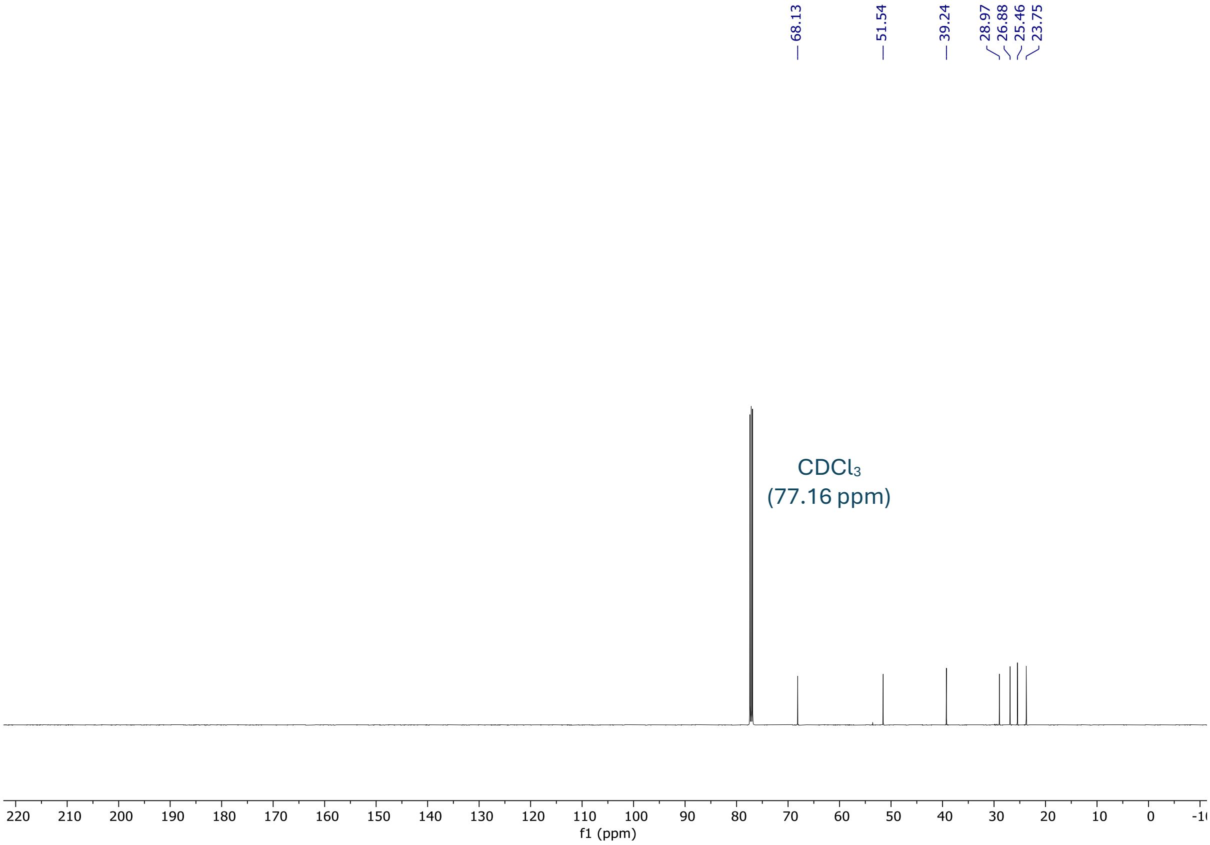

*Compound **S13**. ^{1}H NMR (500 MHz, Chloroform-d) and ^{13}C NMR (126 MHz, Chloroform-d)*

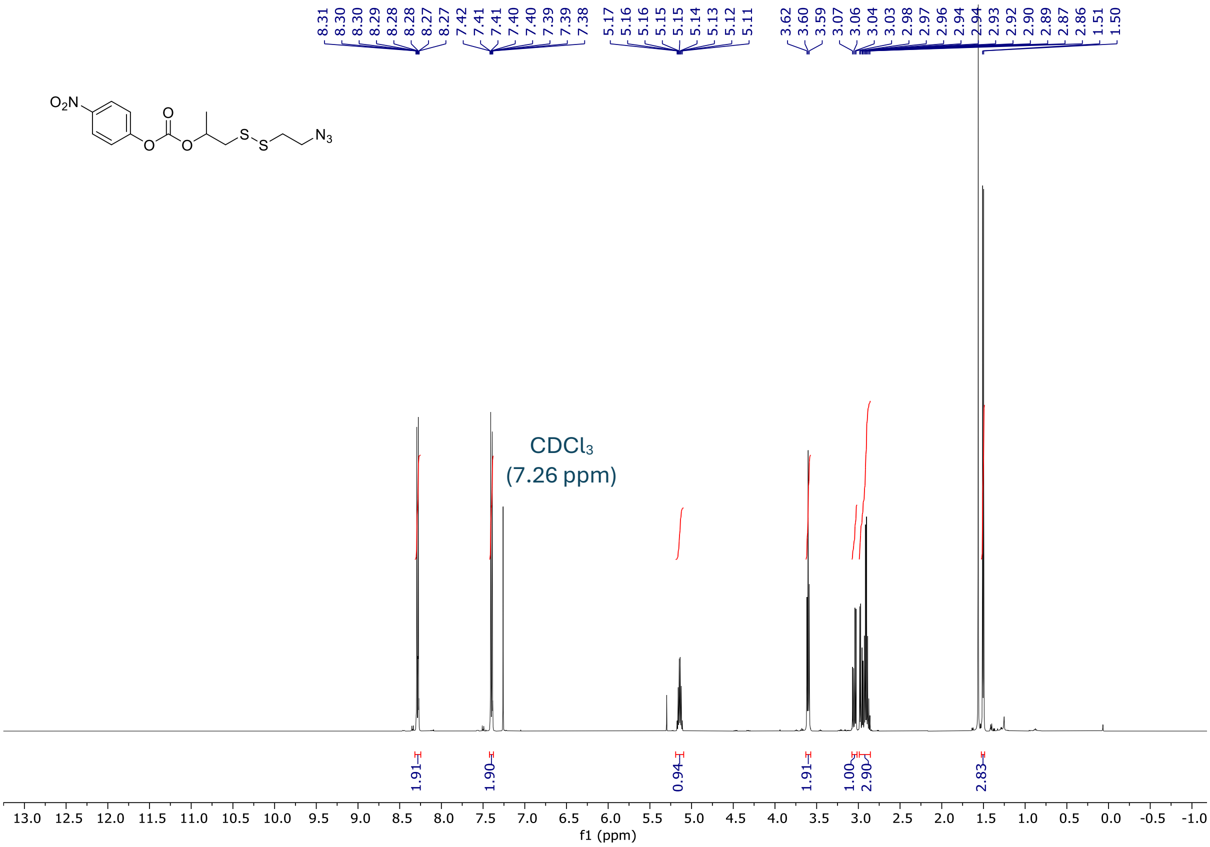

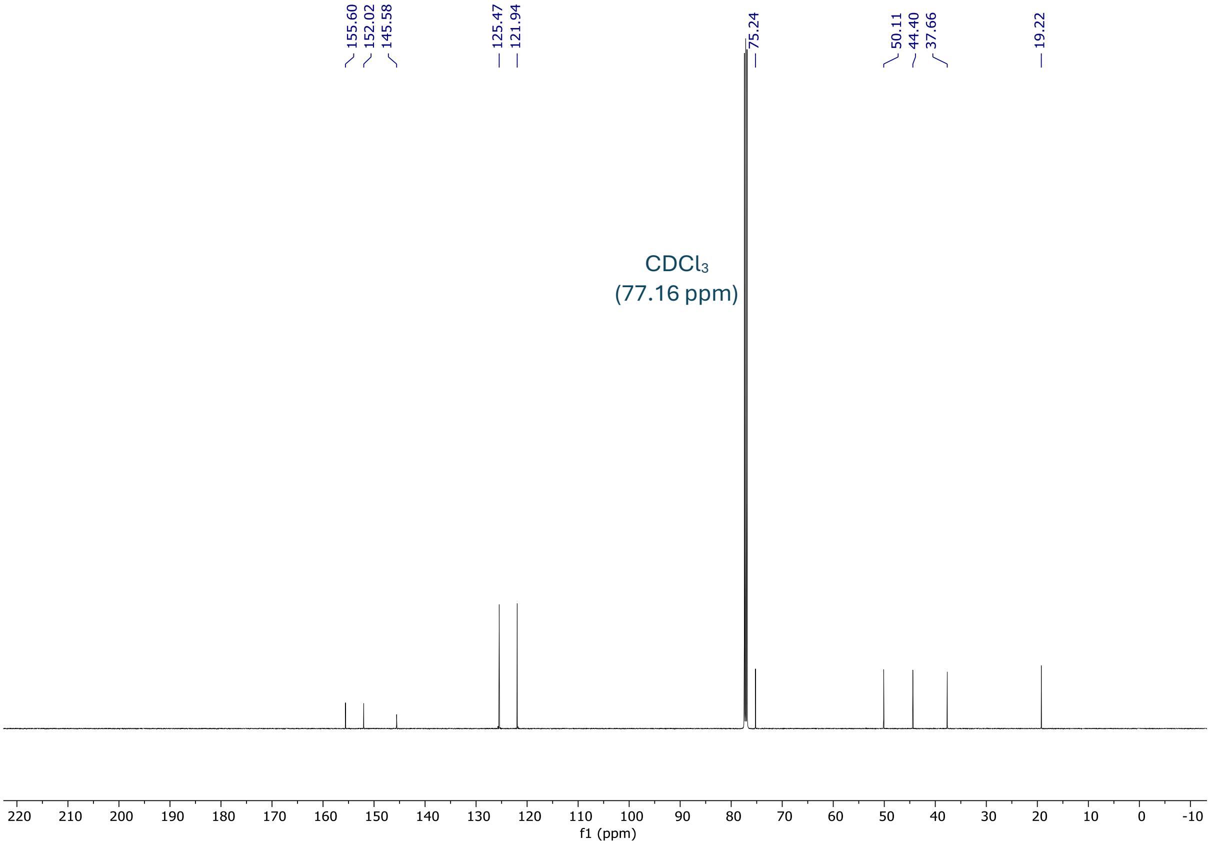

*Compound **S14**. ^{1}H NMR (400 MHz, Chloroform-d) and ^{13}C NMR (101 MHz, Chloroform-d)*

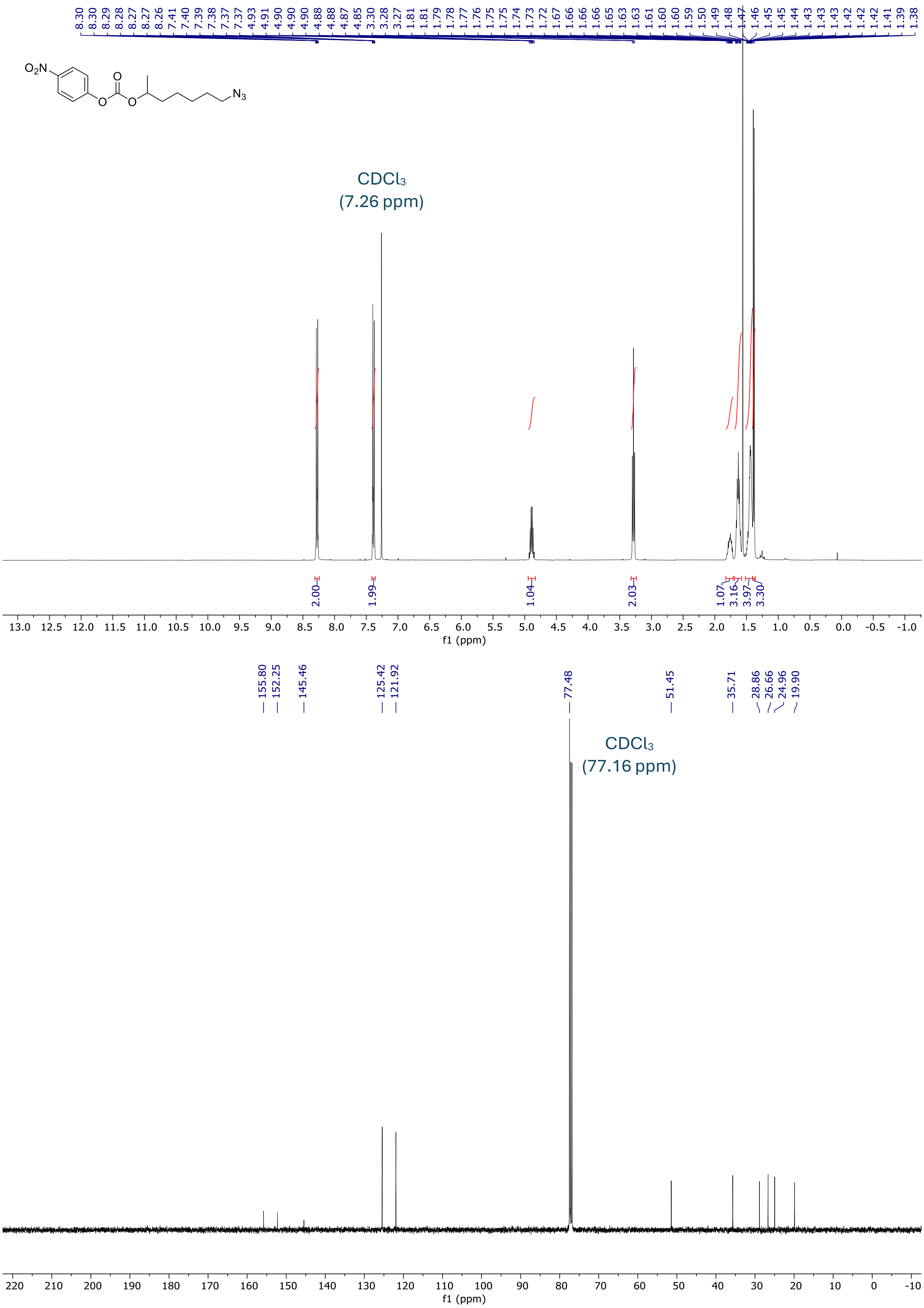

Compound ***S15***. *^{1}H NMR (500 MHz, Chloroform-d) and ^{13}C NMR (126 MHz, Chloroform-d)*

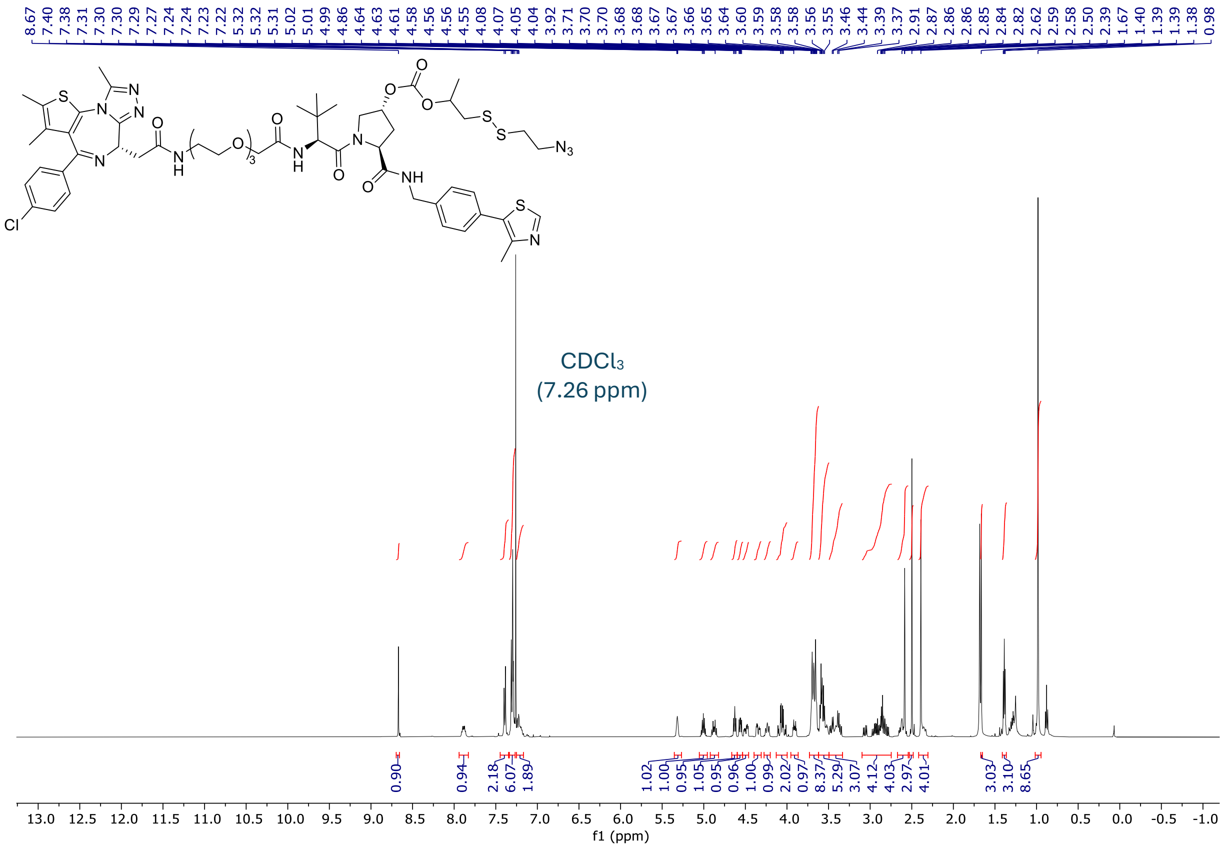

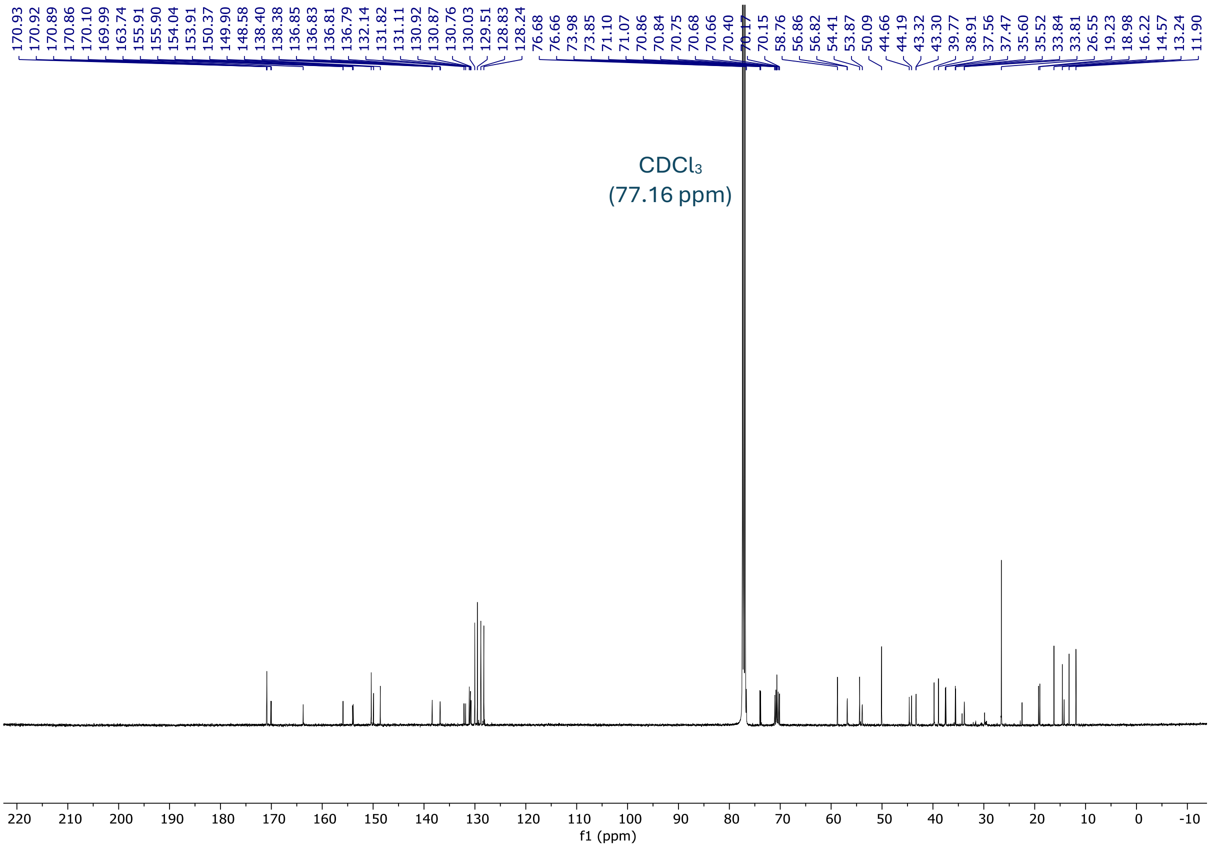

*Compound **S16**. ^{1}H NMR (500 MHz, Methylene Chloride-d_2) and ^{13}C NMR (126 MHz, Methylene Chloride-d_2)*

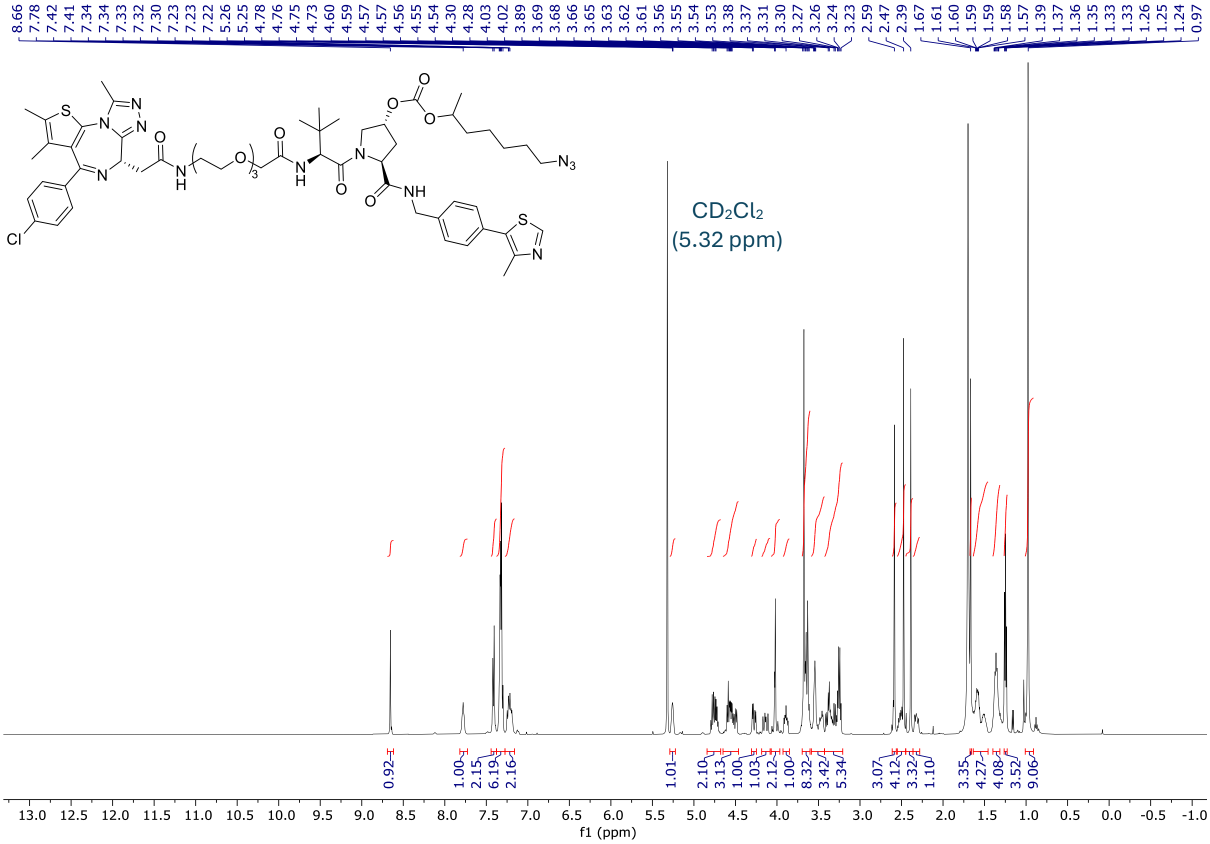

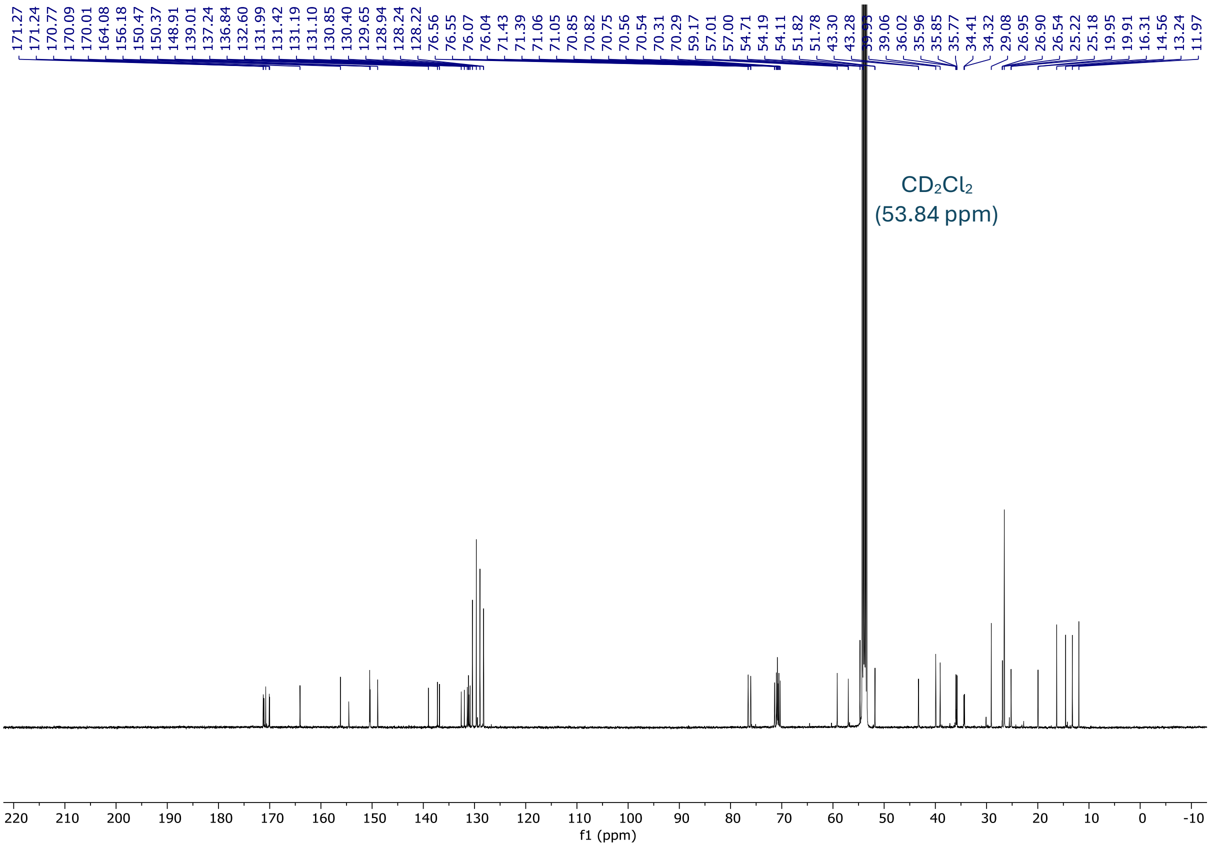

*Compound **S17**. ^{1}H NMR (400 MHz, Chloroform-d) and ^{13}C NMR 101 MHz, Chloroform-d)*

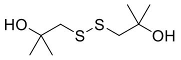

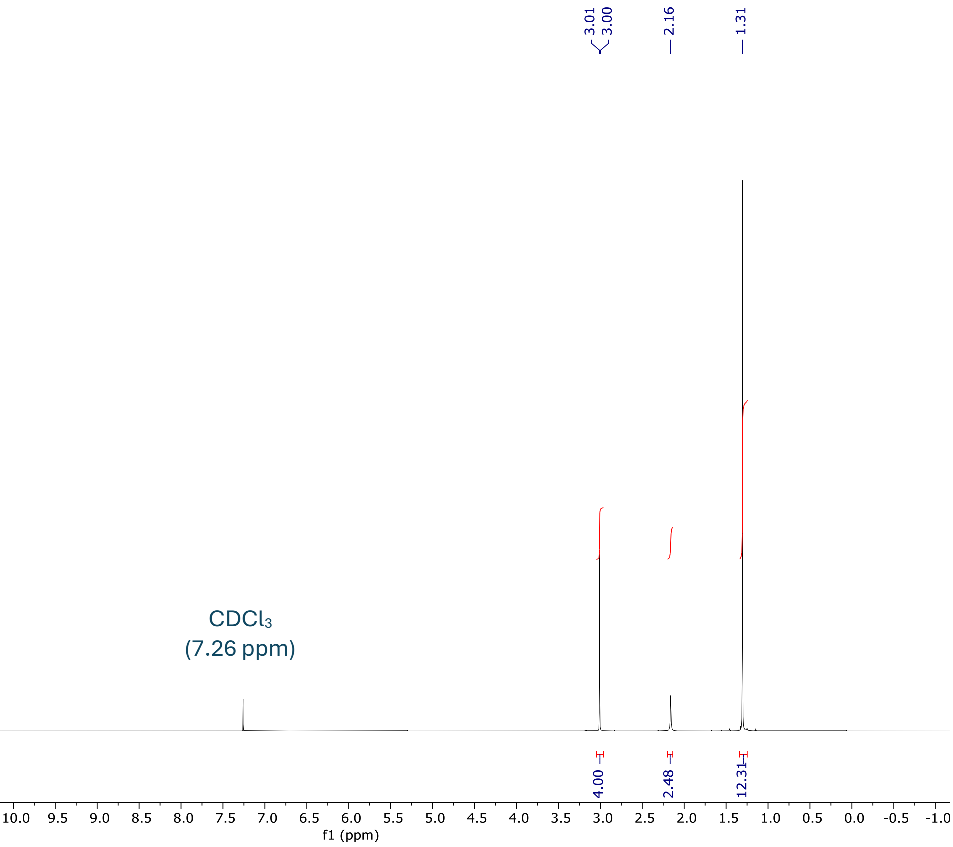

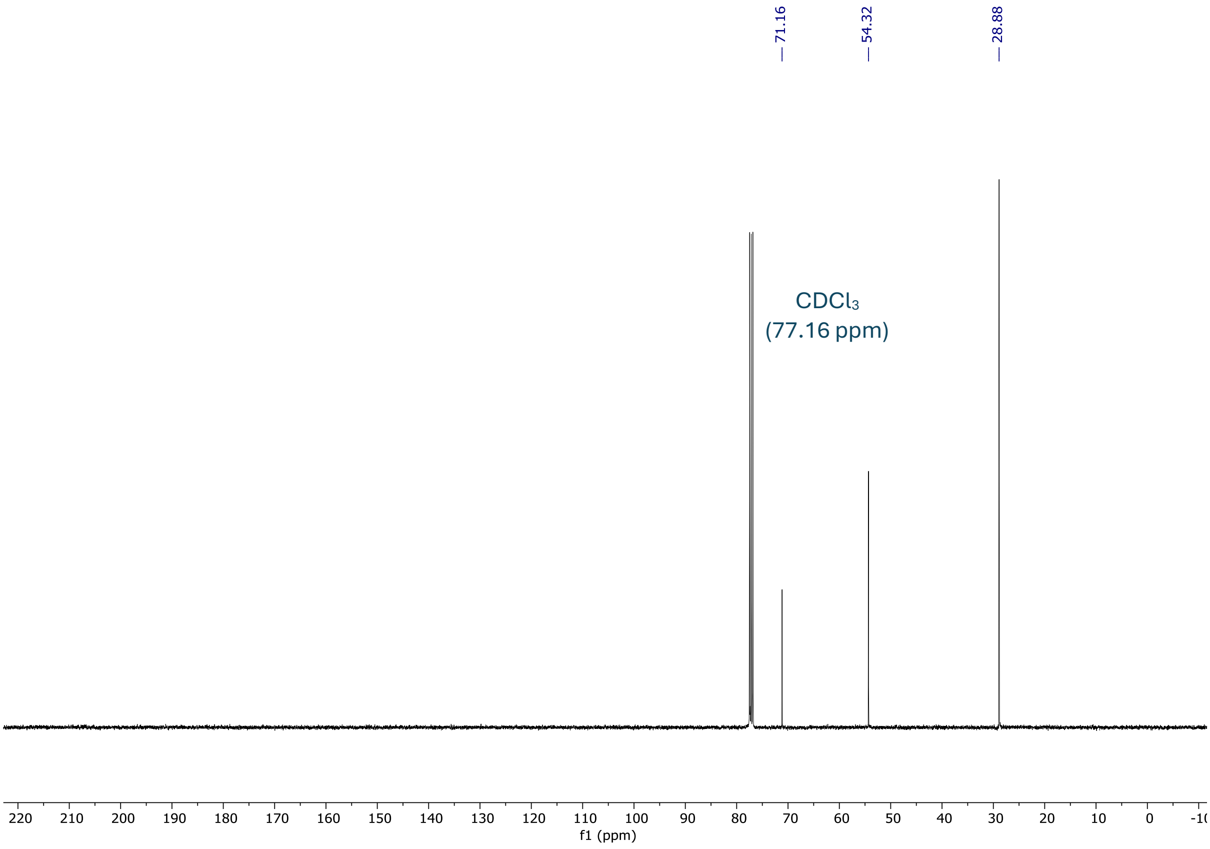

*Compound **S18**. ^{1}H NMR (400 MHz, Chloroform-d) and ^{13}C NMR (101 MHz, Chloroform-d)*

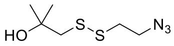

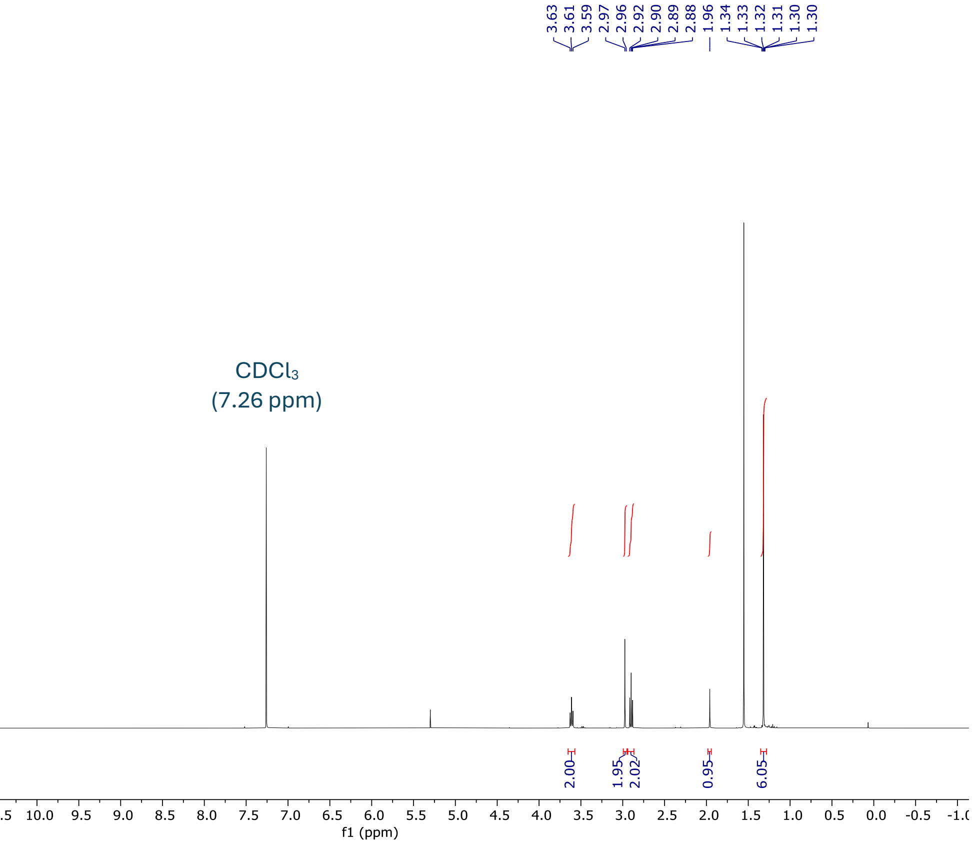

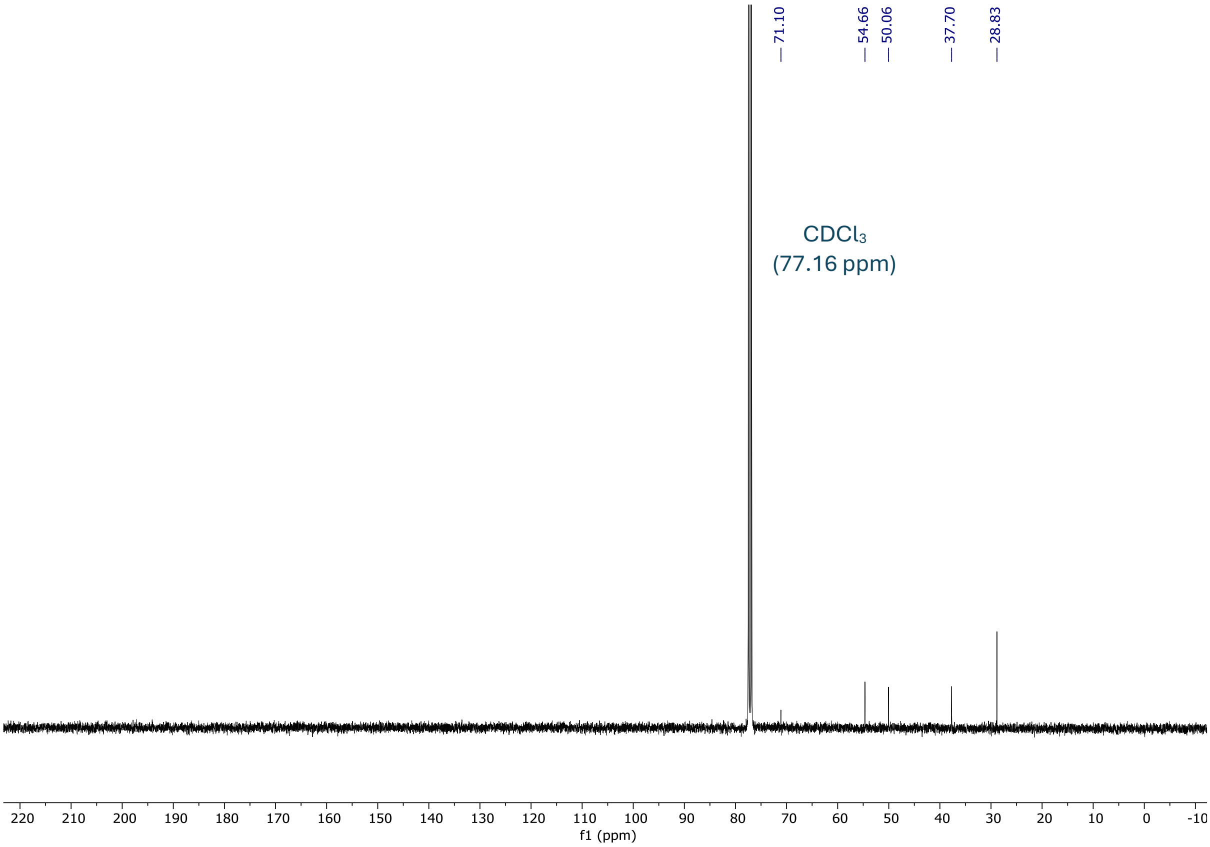

*Compound **S20**. ^{1}H NMR (500 MHz, Chloroform-d) and ^{13}C NMR (126 MHz, Chloroform-d)*

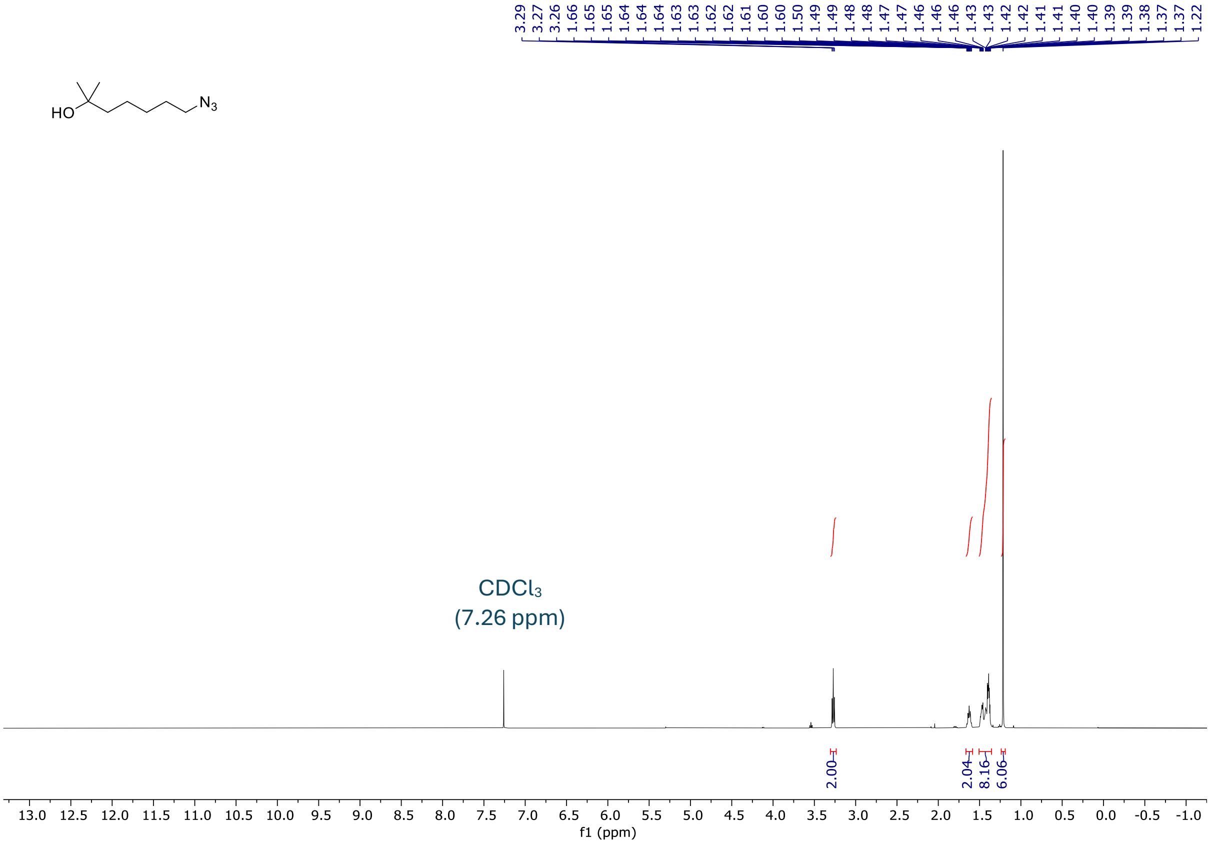

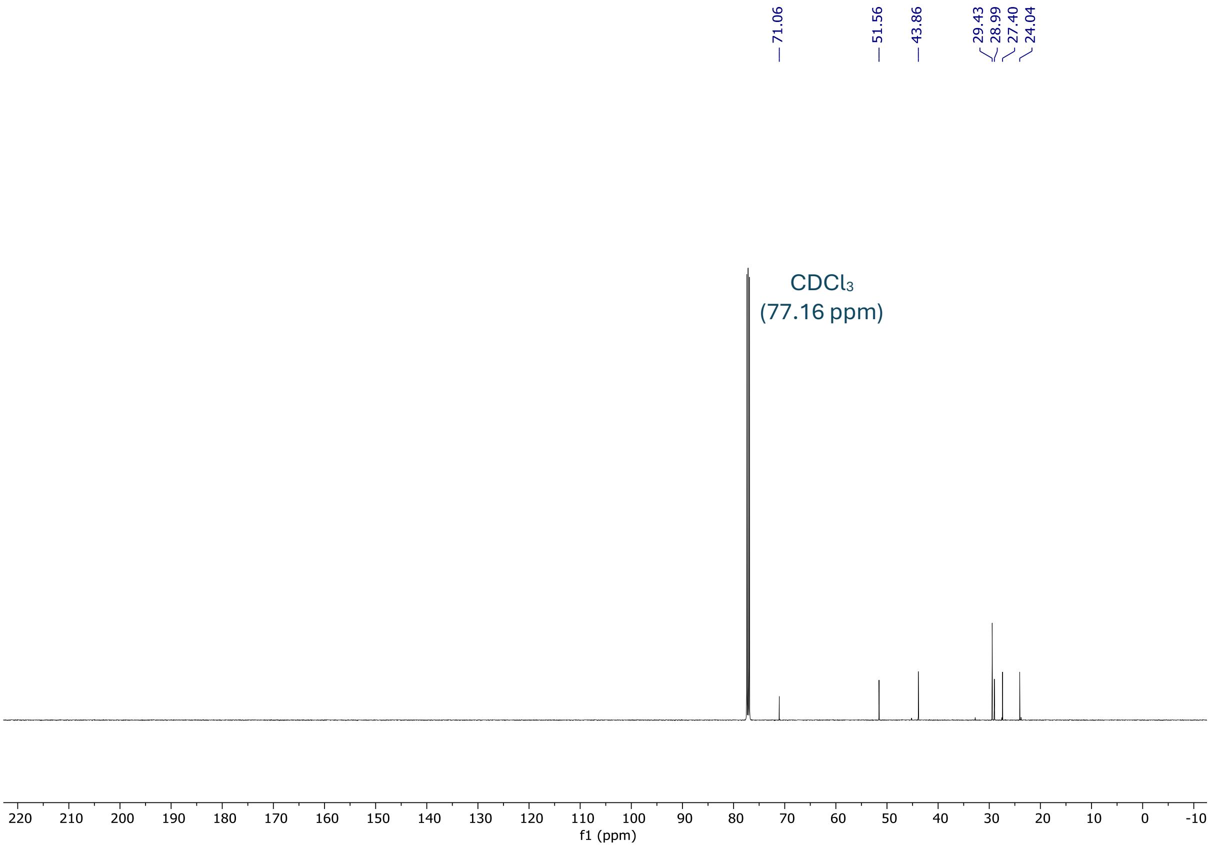

*Compound **S21**. ^1H NMR (400 MHz, Chloroform-d) and ^{13}C NMR (101 MHz, Chloroform-d)*

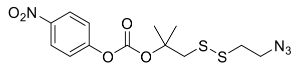

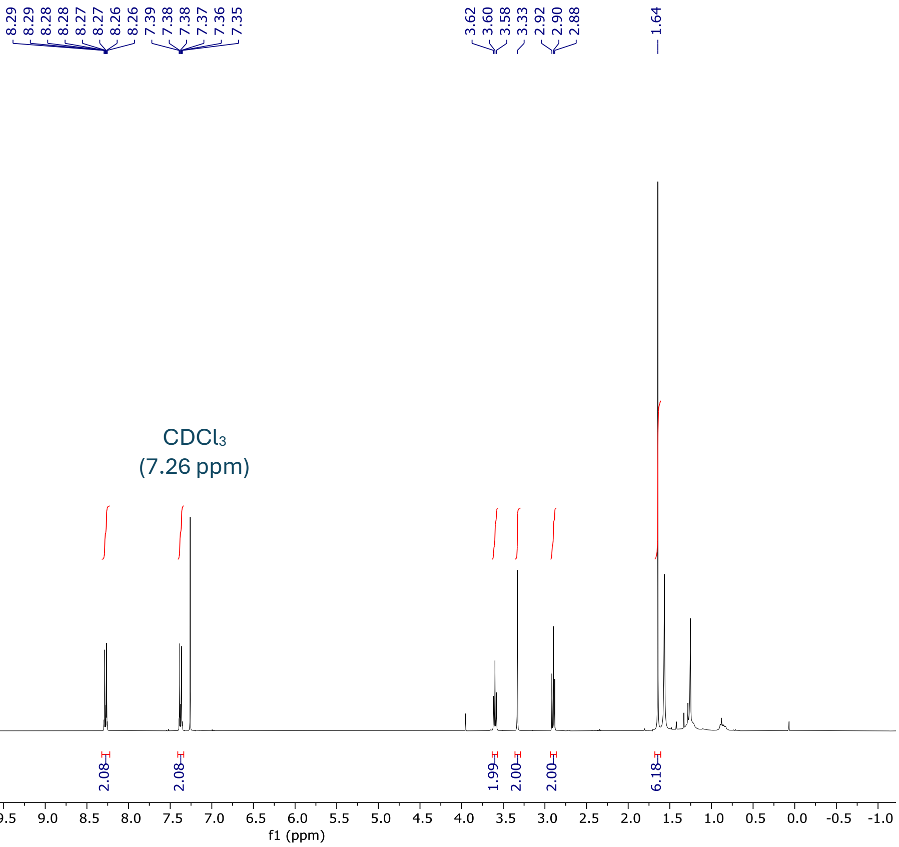

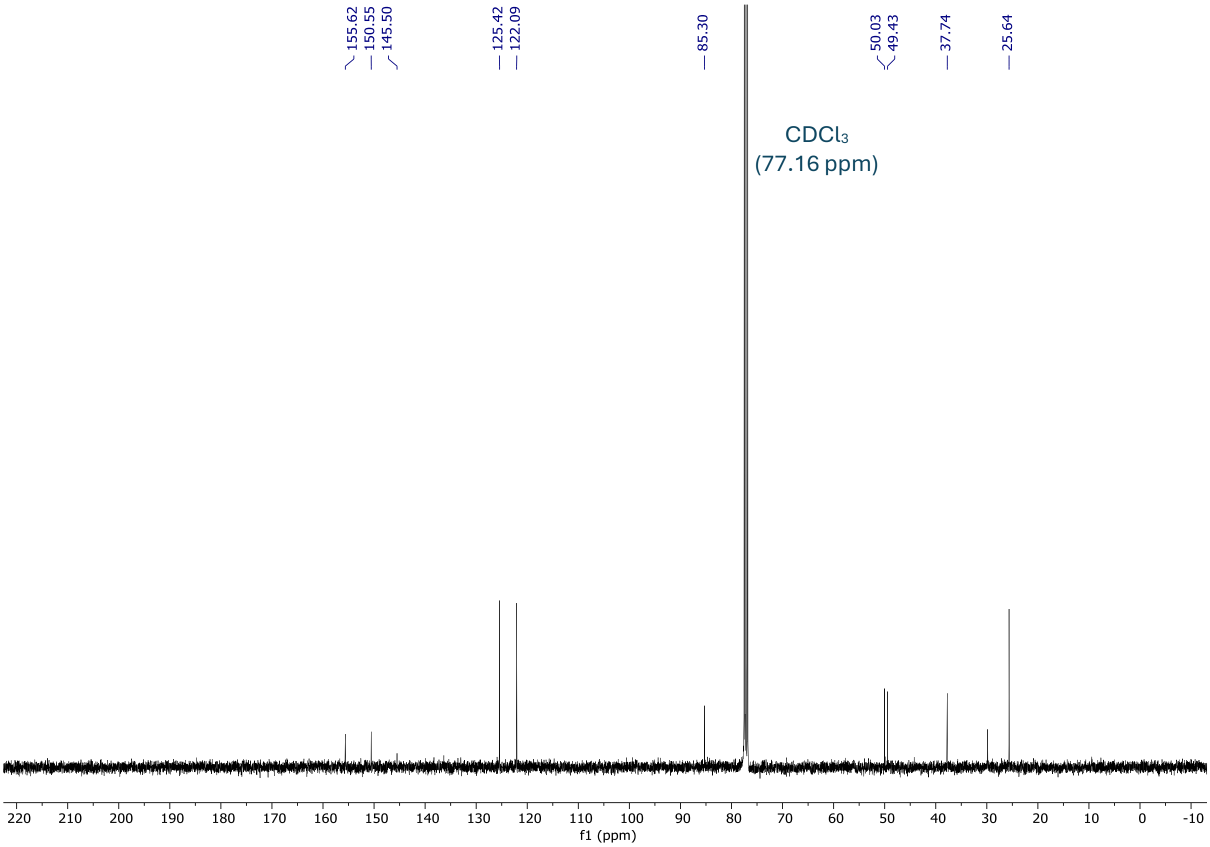

*Compound **S22**. ^{1}H NMR (500 MHz, Chloroform-d) and ^{13}C NMR (126 MHz, Chloroform-d)*

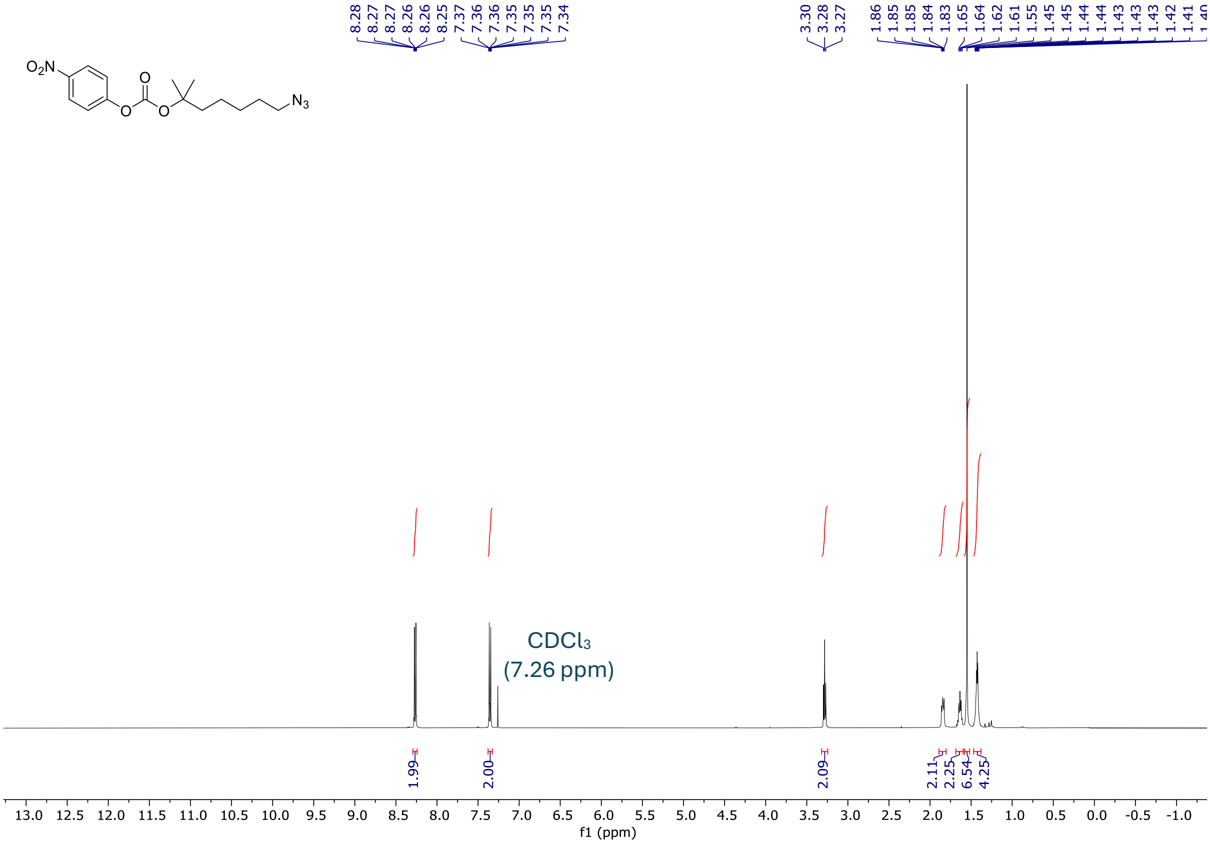

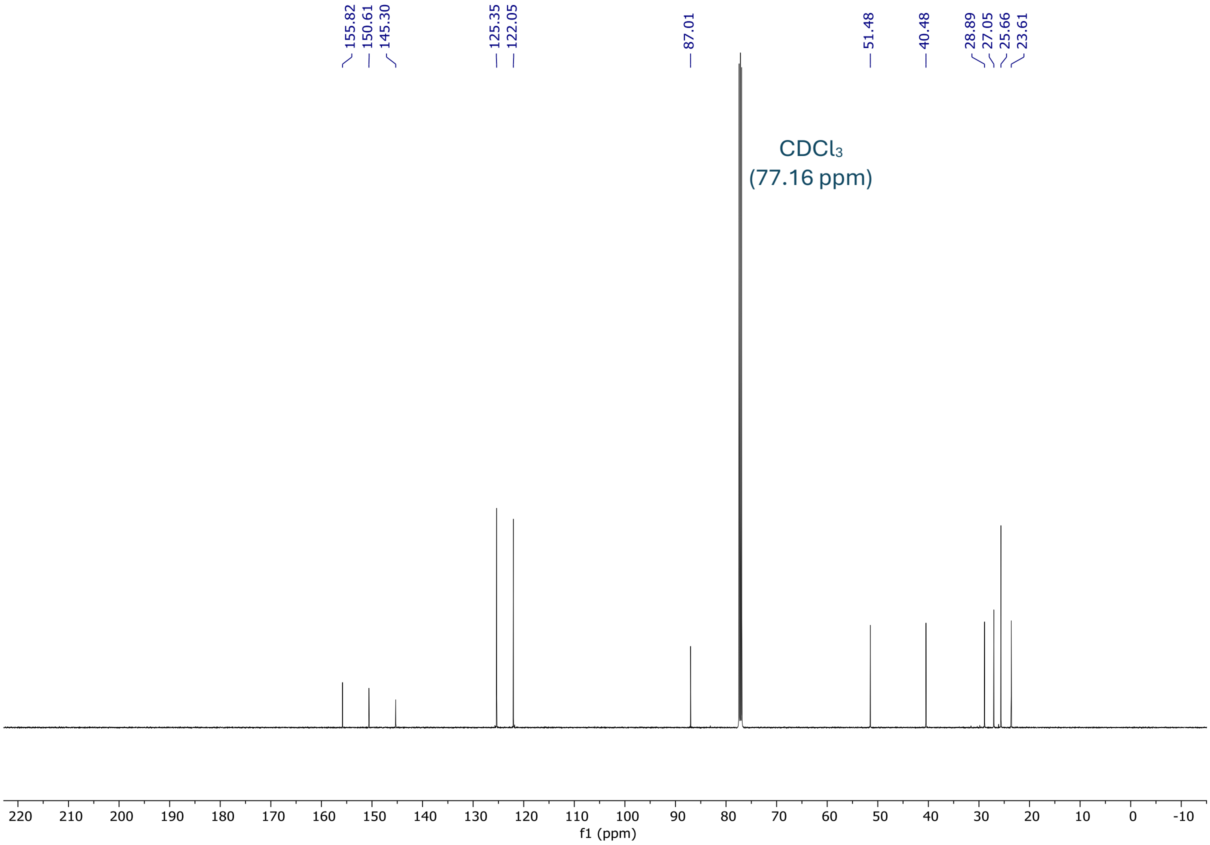

*Compound **S23**. ^{1}H NMR (400 MHz, Methylene Chloride-d$_2$) and ^{13}C NMR (101 MHz, Methylene Chloride-d$_2$)*

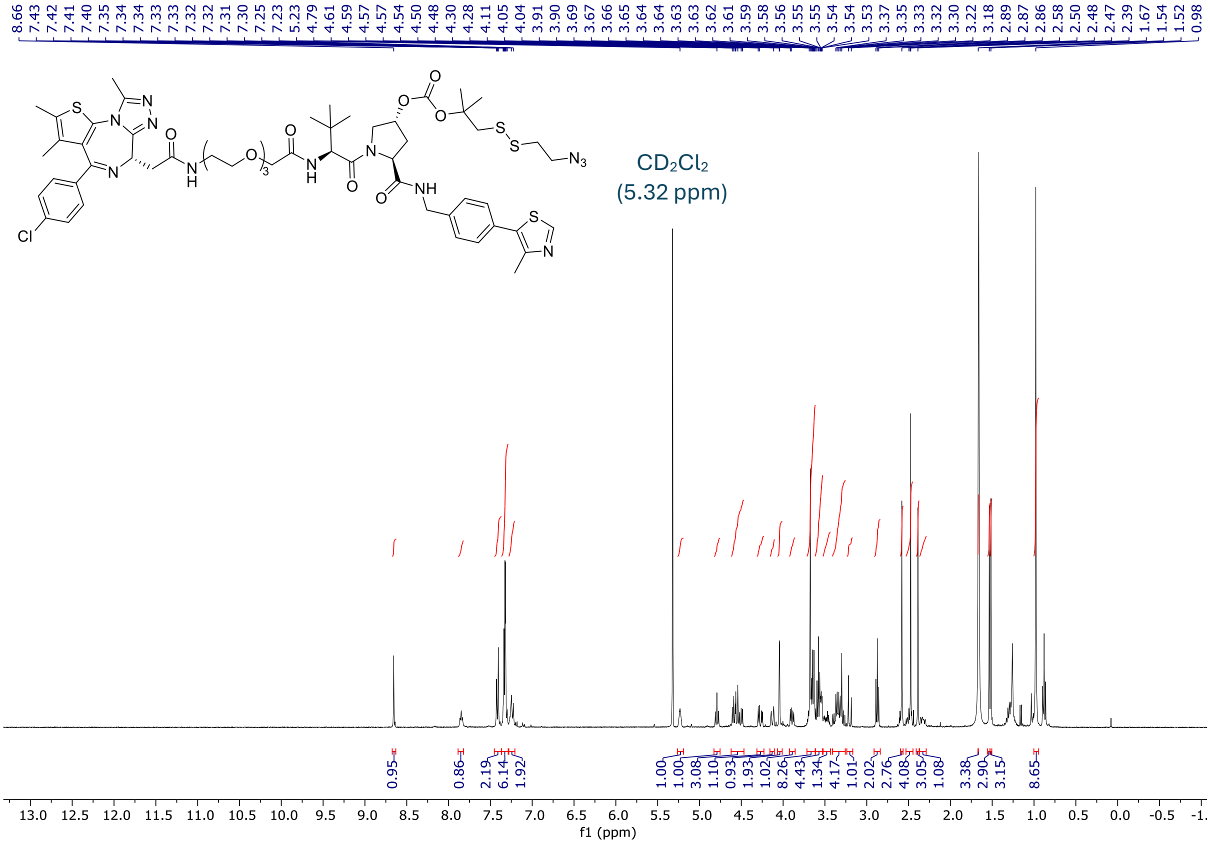

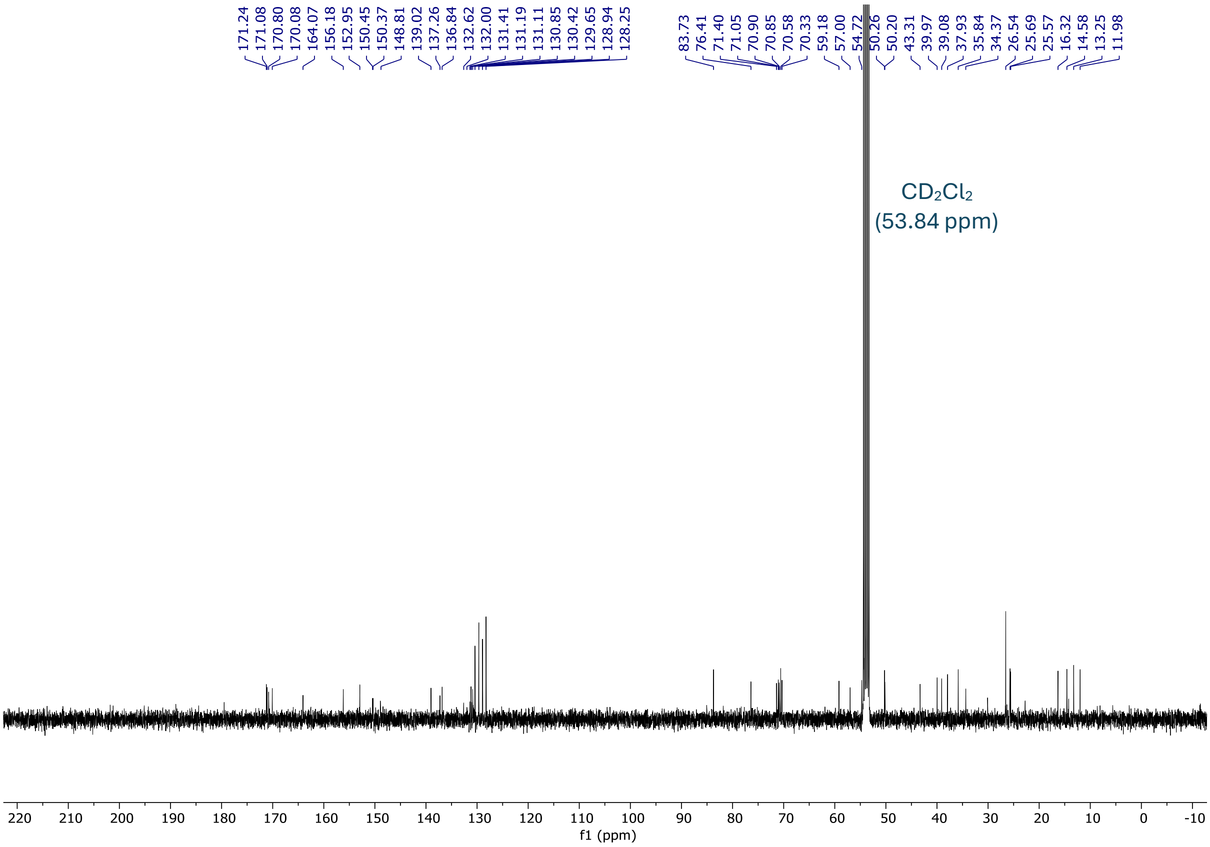

*Compound **S24***. ^{1}H NMR (500 MHz, Methylene Chloride-d_2) and ^{13}C NMR (126 MHz, Methylene Chloride-d_2)

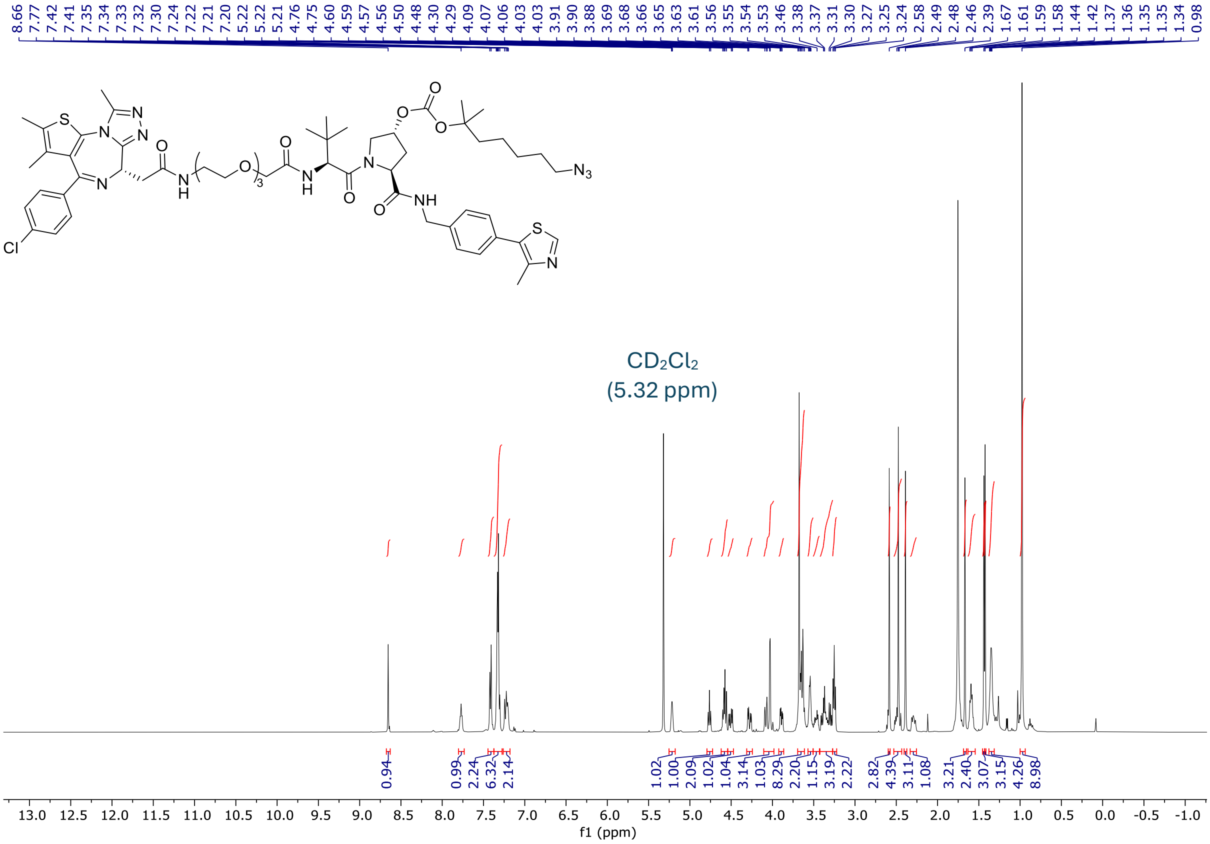

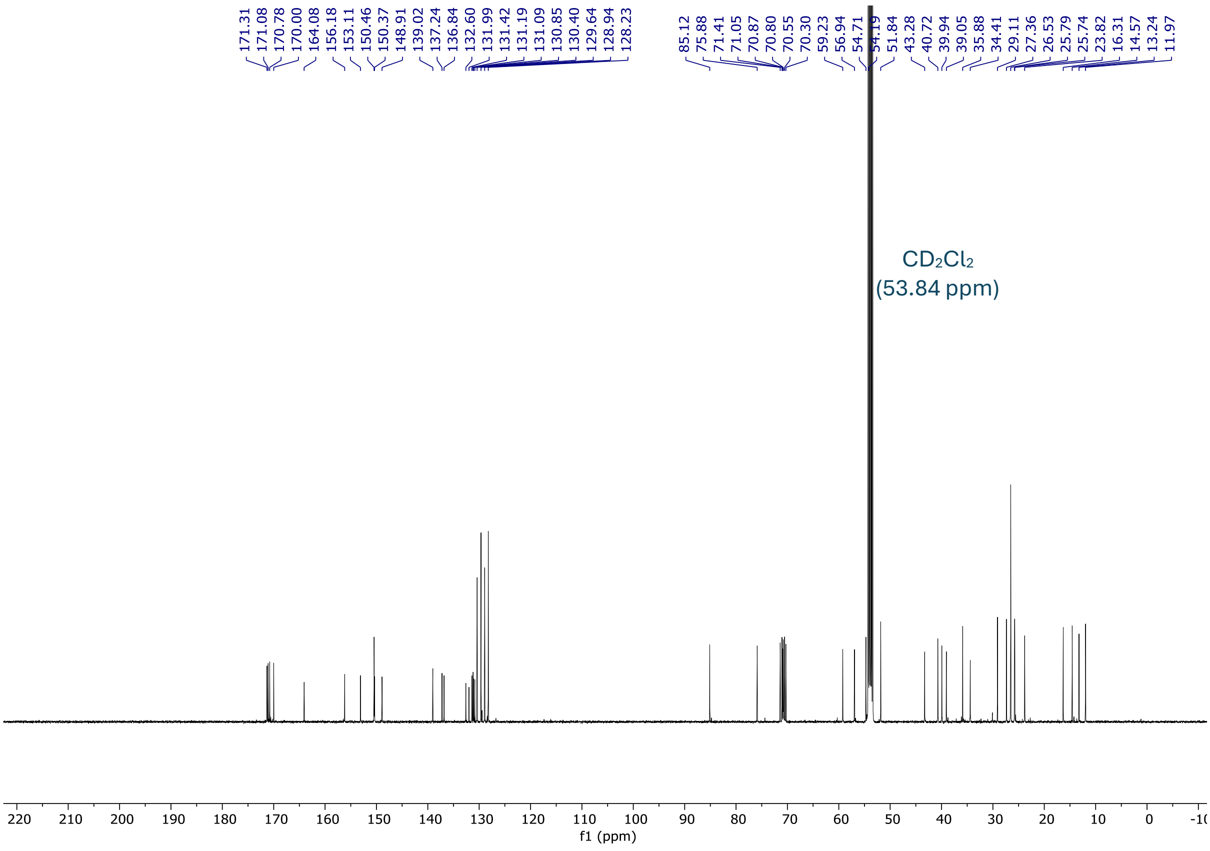

*Compound **S25**. ^{1}H NMR (400 MHz, Methylene Chloride-d_2) and ^{13}C NMR (101 MHz, Methylene Chloride-d_2)*

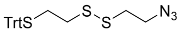

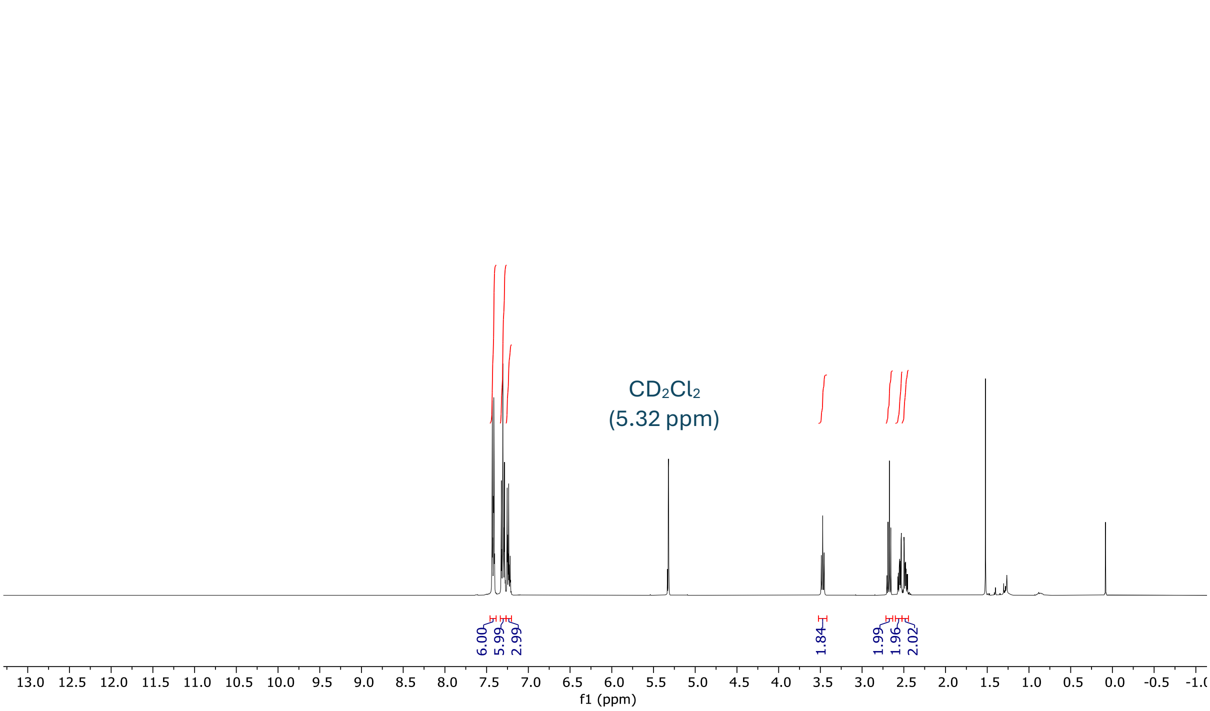

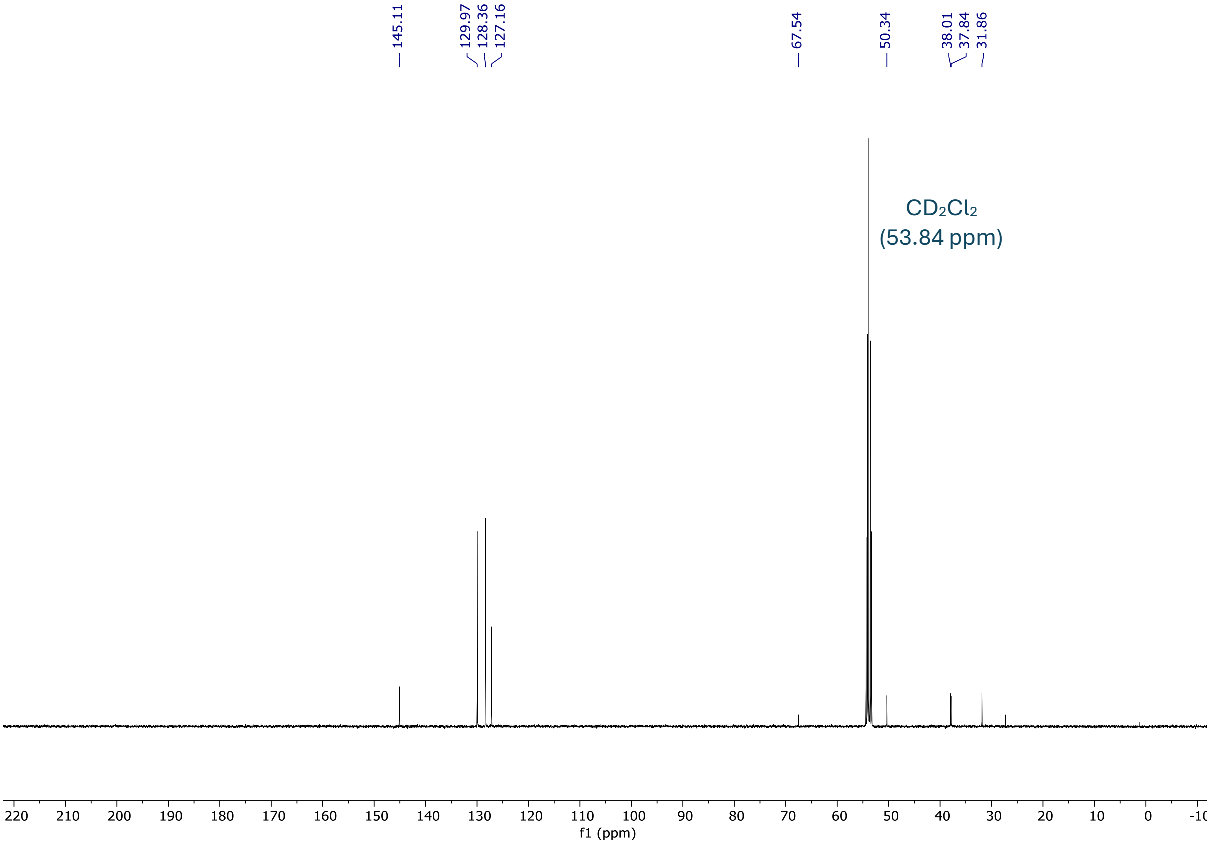

*Compound **S26**. ^{1}H NMR (400 MHz, Methylene Chloride-d_2) and ^{13}C NMR (101 MHz, Methylene Chloride-d_2)*

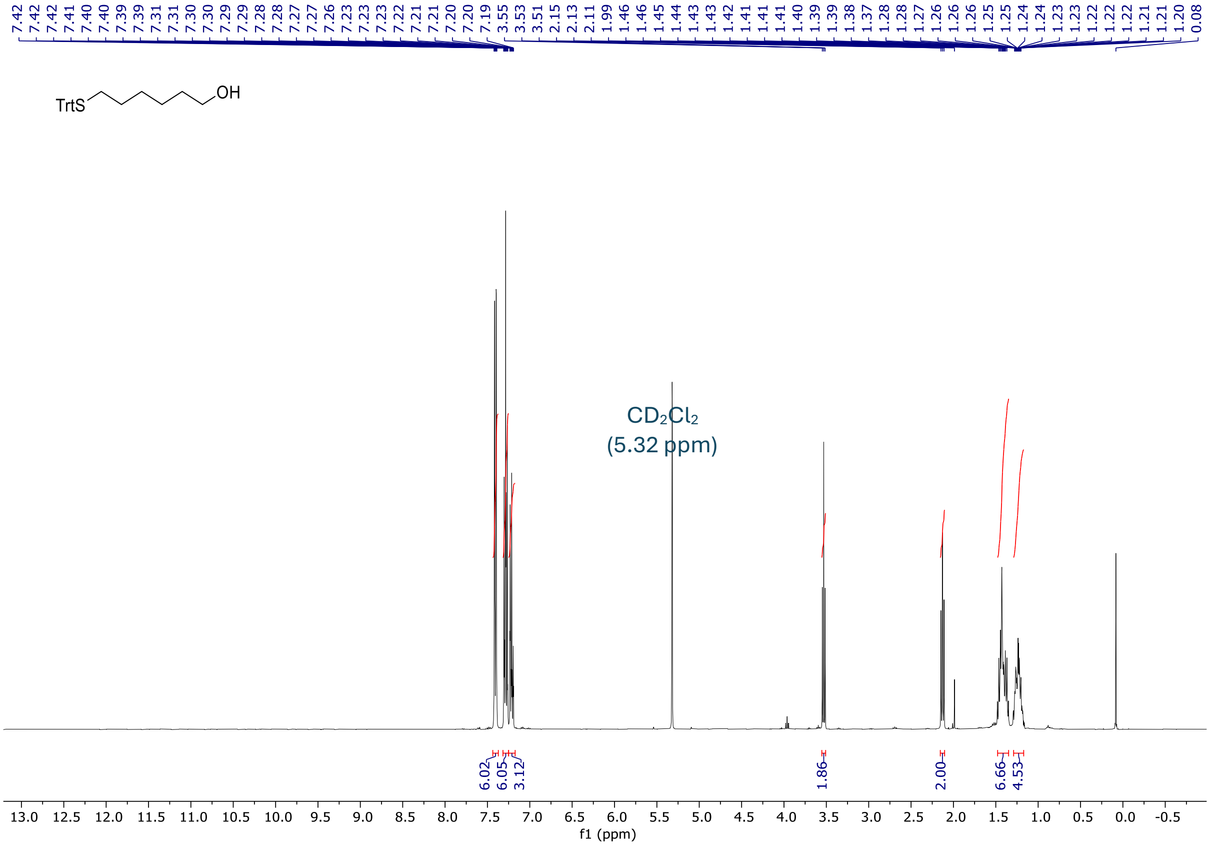

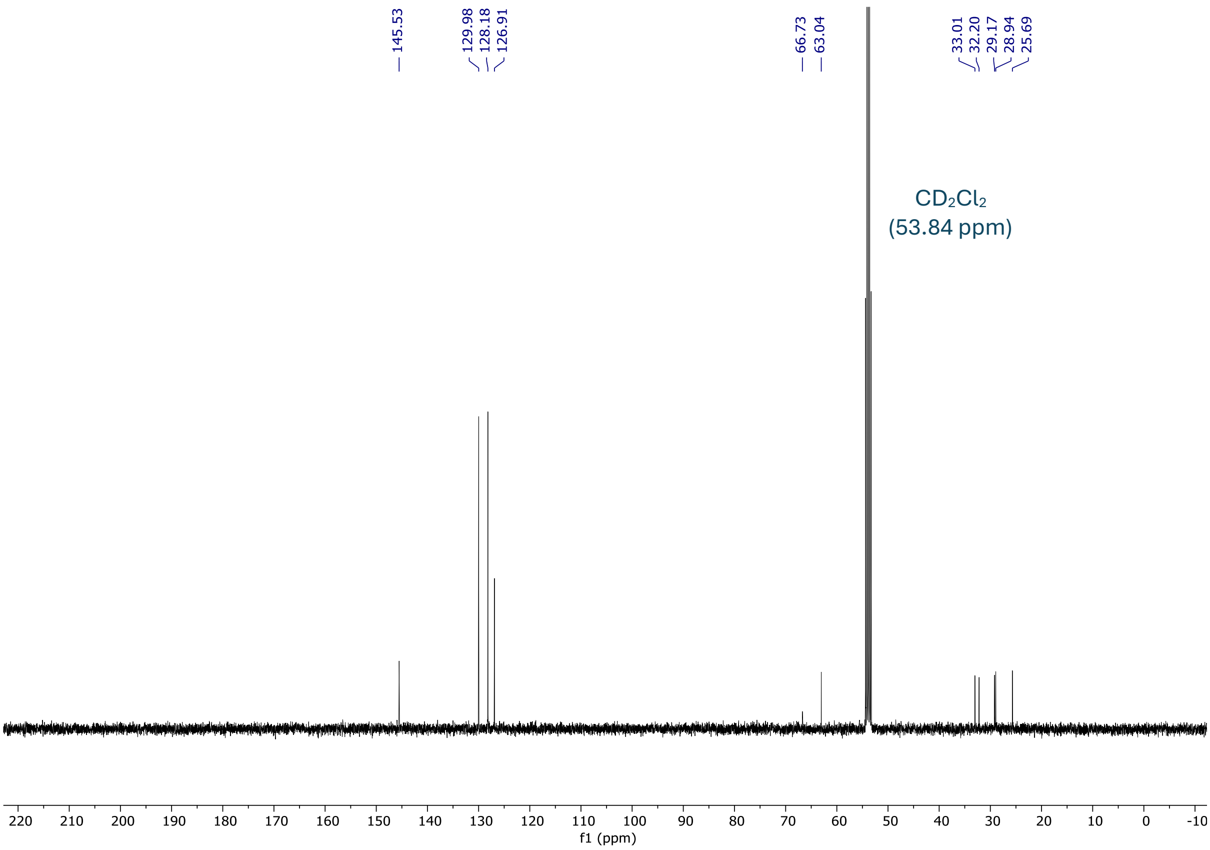

*Compound **S27**. ^{1}H NMR (400 MHz, Methylene Chloride-d_2) and ^{13}C NMR (101 MHz, Methylene Chloride-d_2)*

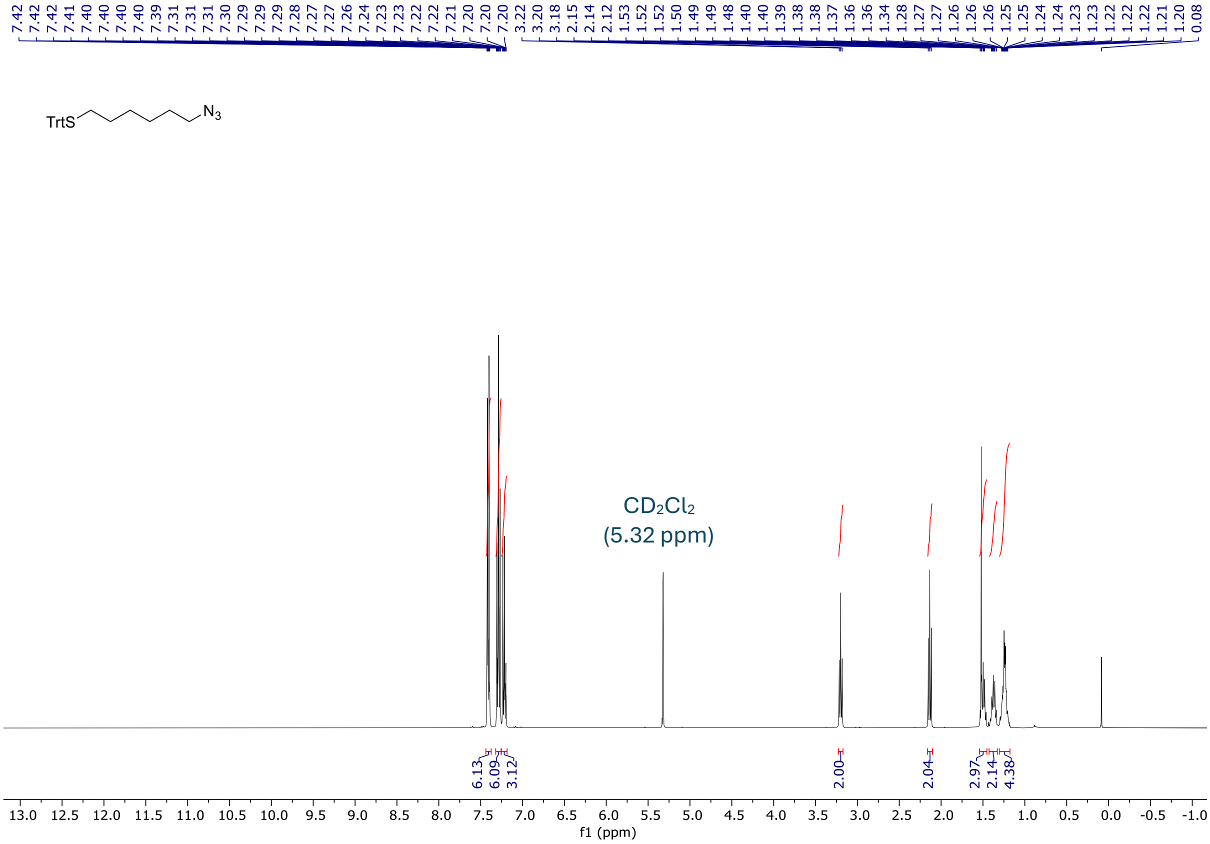

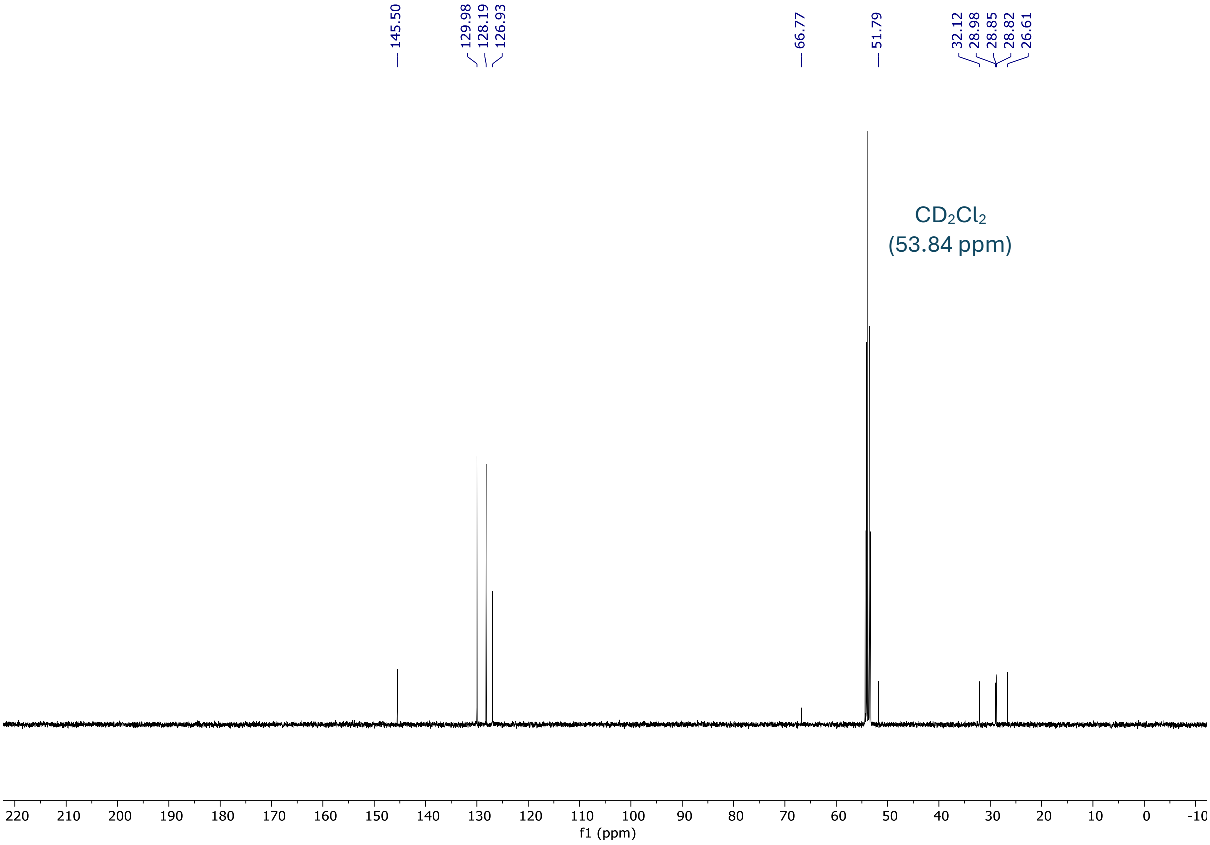

Compound **S28**. ^{1}H NMR (*400 MHz, Chloroform-d*) and ^{13}C NMR (*101 MHz, Chloroform-d*)

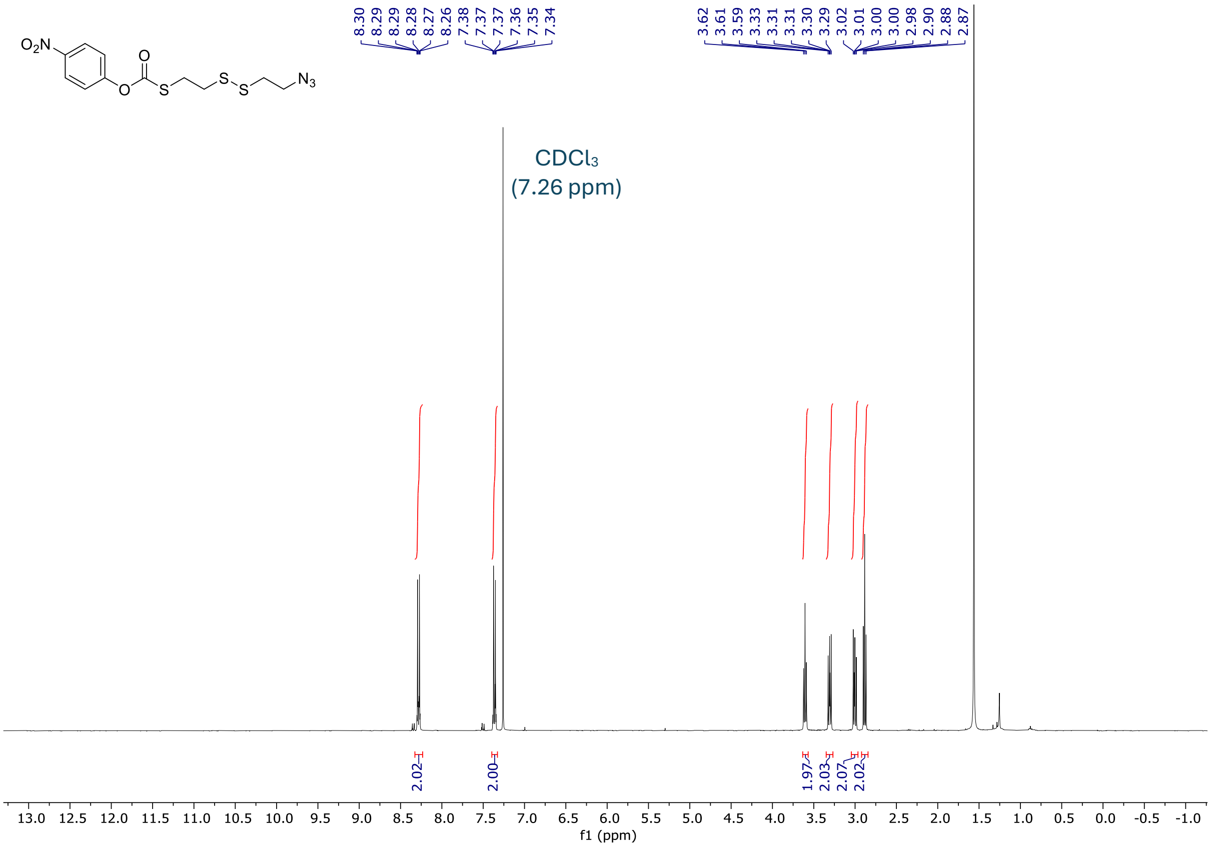

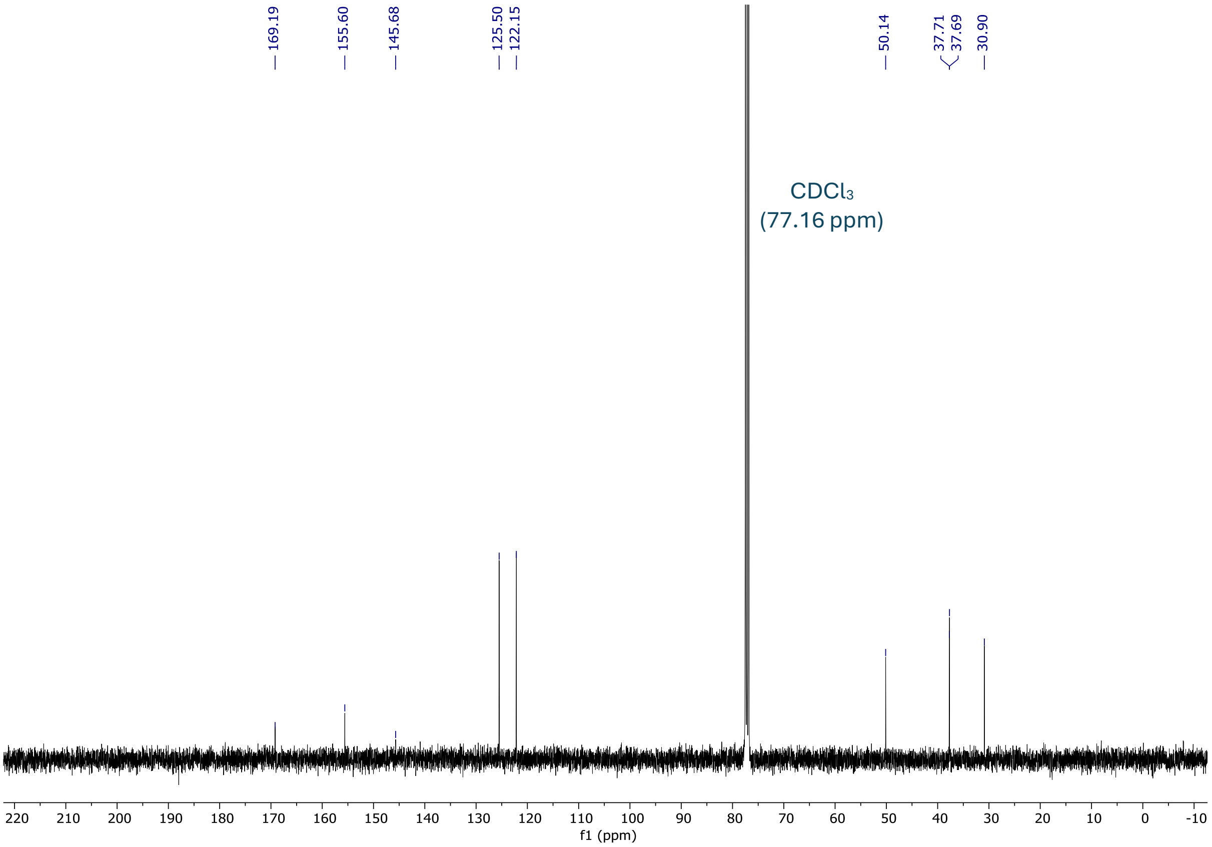

*Compound **S29**. ^{1}H NMR (400 MHz, Chloroform-d) and ^{13}C NMR (101 MHz, Chloroform-d)*

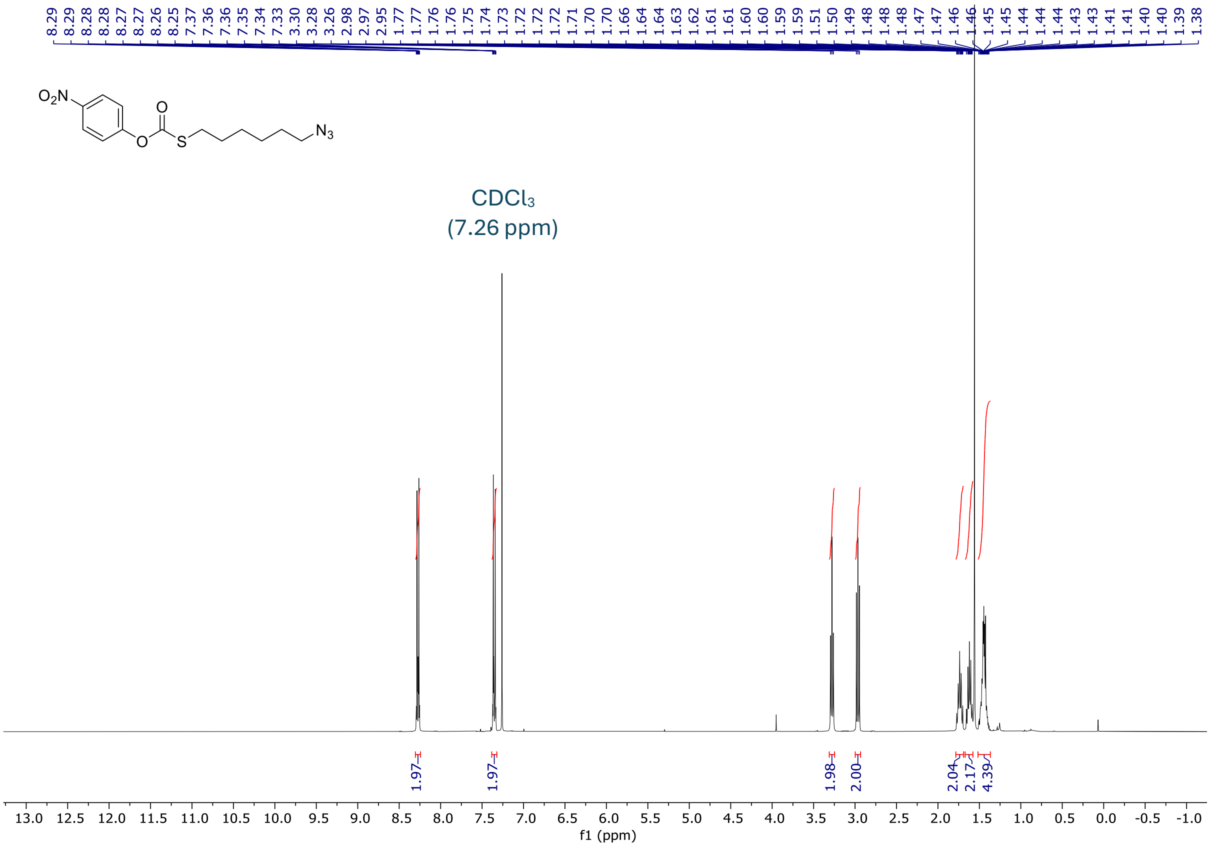

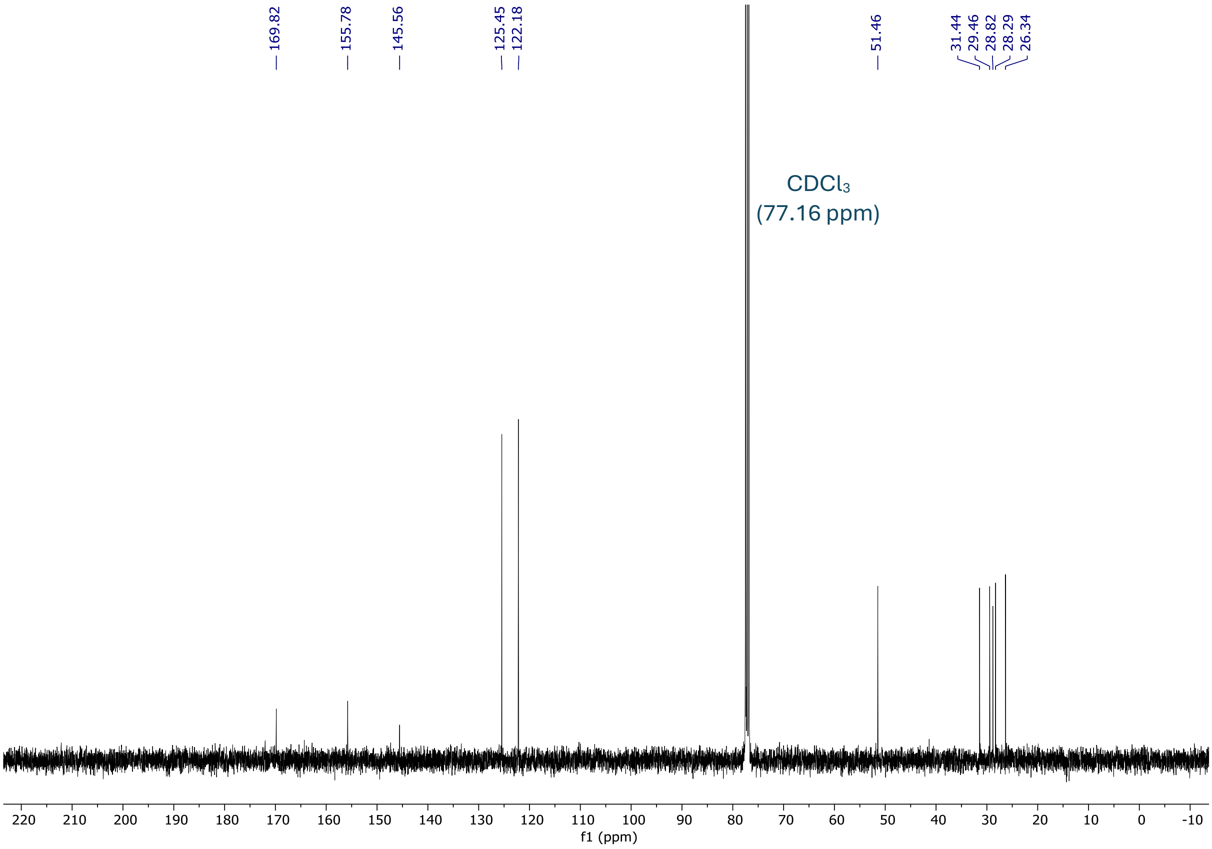

*Compound **S30**. ^{1}H NMR (500 MHz, Methylene Chloride-d_2), ^{13}C NMR (126 MHz, Methylene Chloride-d_2) and DEPT135 NMR (126 MHz, Methylene Chloride-d_2)*

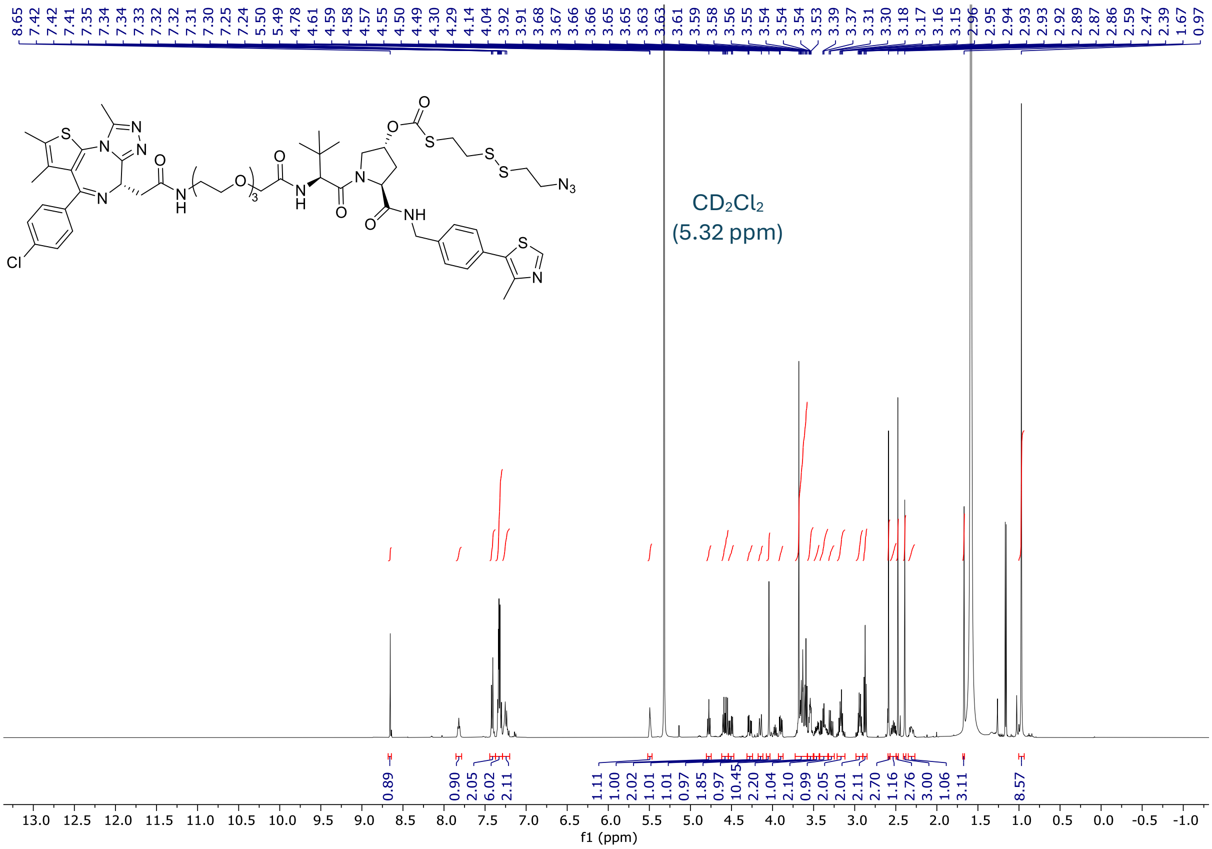

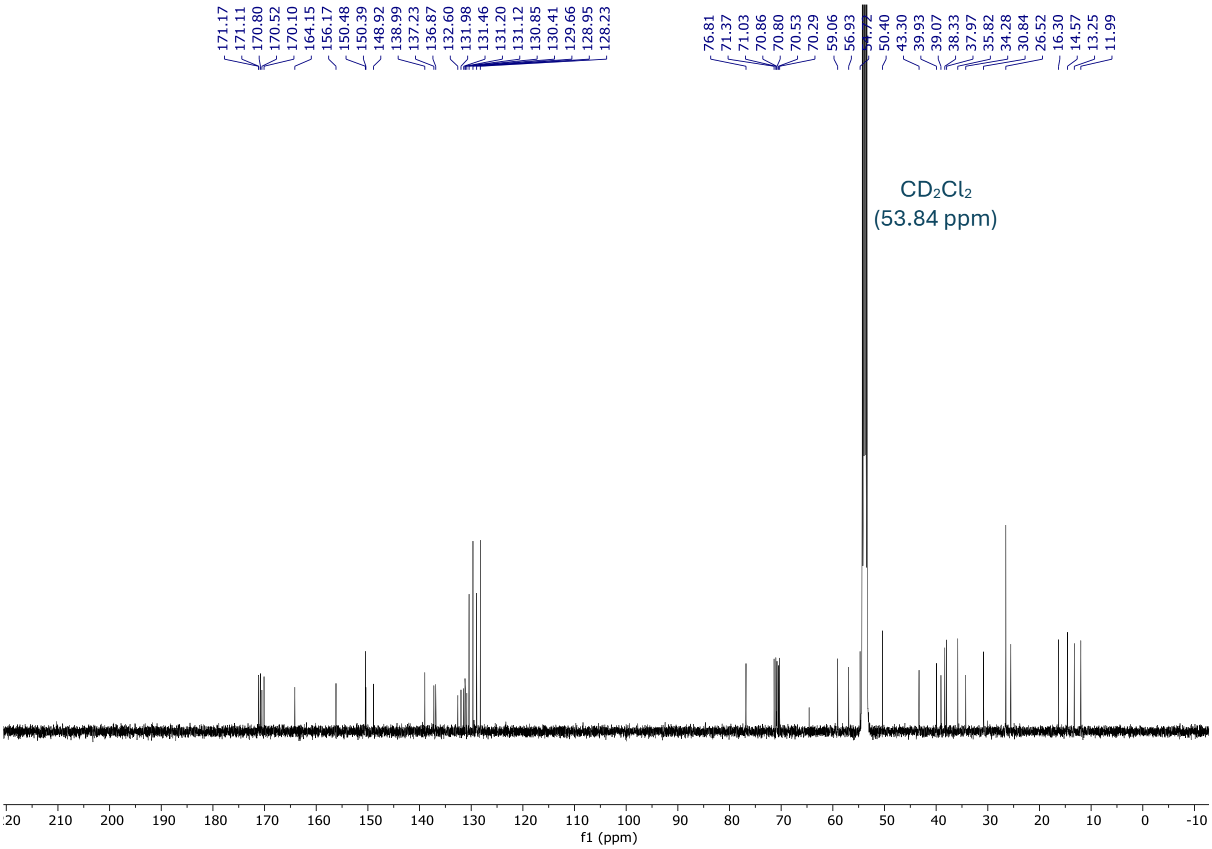

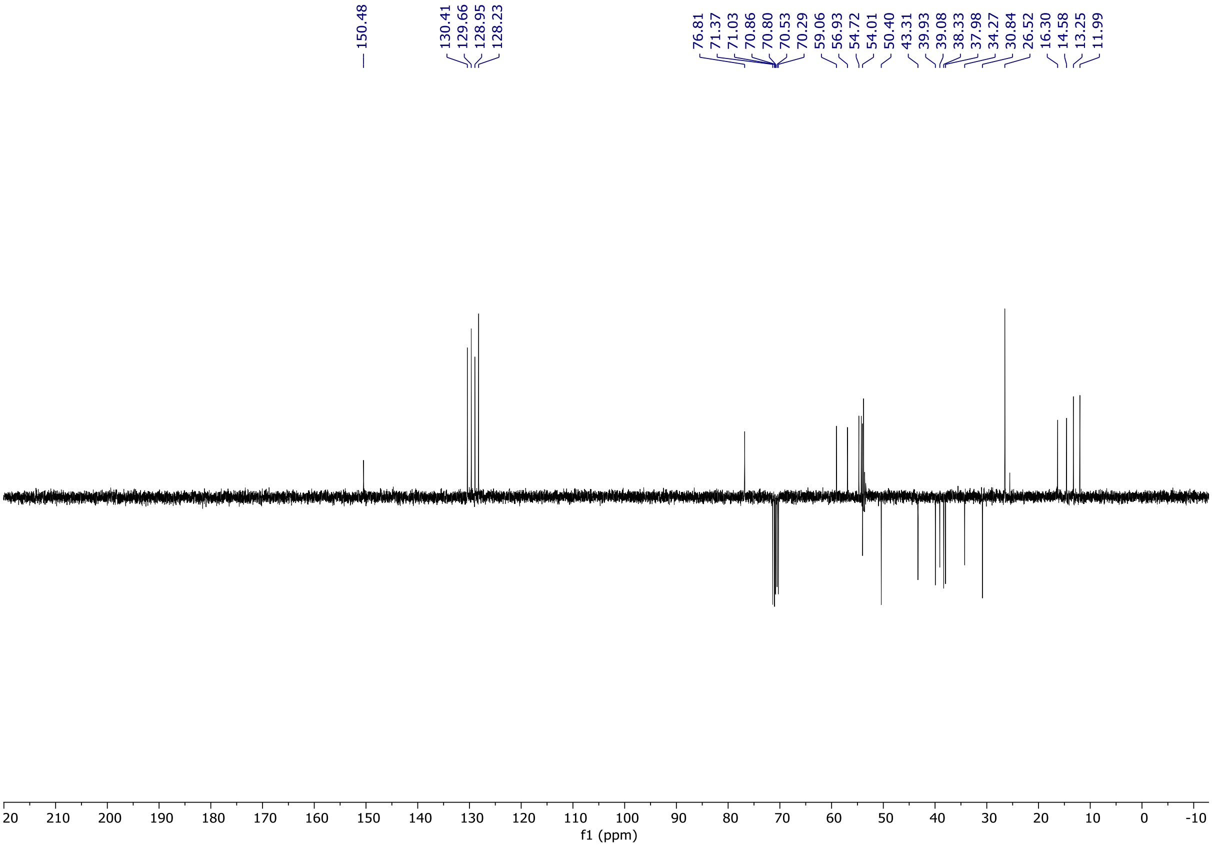
150.48
130.41
129.66
128.95
128.23
76.81
71.37
71.03
70.86
70.80
70.53
70.29
59.06
56.93
54.72
54.01
50.40
43.31
39.93
39.08
38.33
37.98
34.27
30.84
26.52
16.30
14.58
13.25
11.99
20 210 200 190 180 170 160 150 140 130 120 110 100 90 80 70 60 50 40 30 20 10 0 -10
f1 (ppm)

Compound ***S31***. *^{1}H NMR (500 MHz, Methylene Chloride-d₂) and ^{13}C NMR (126 MHz, Methylene Chloride-d₂)*

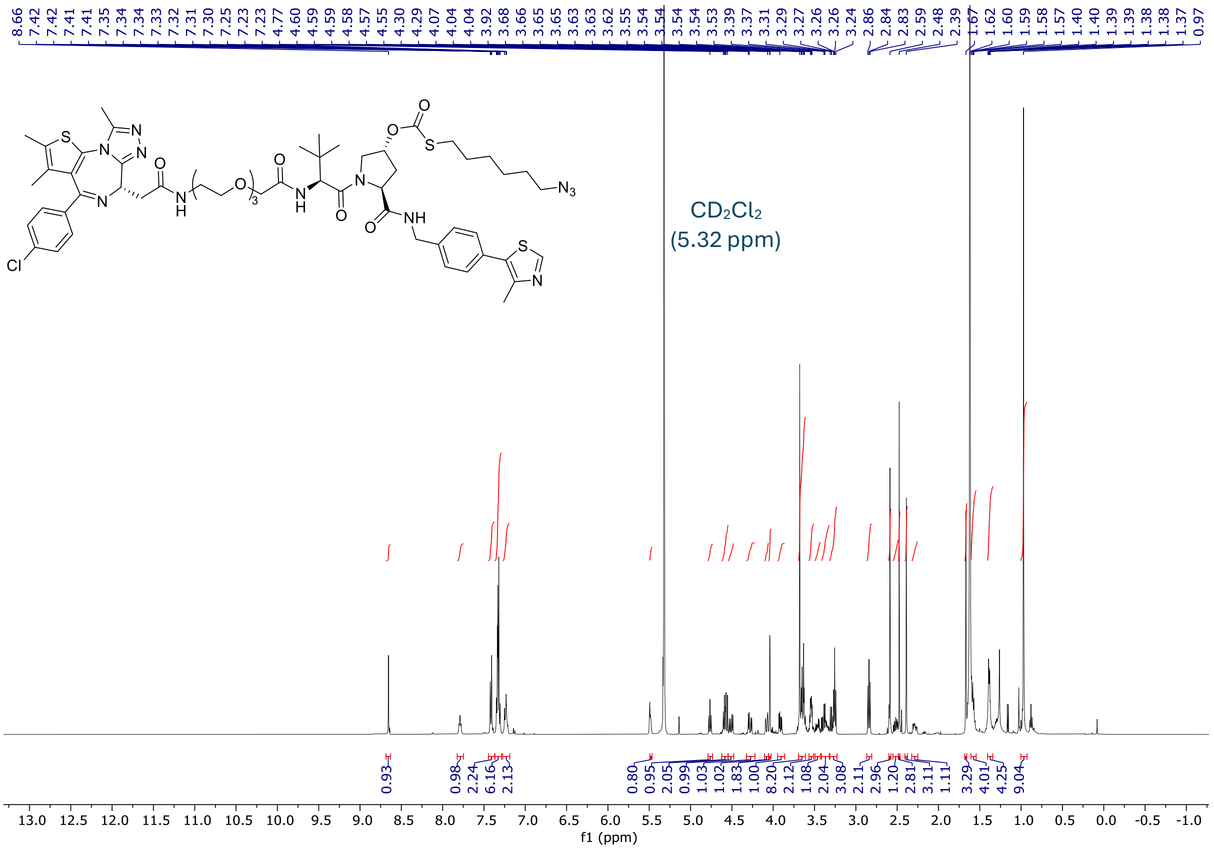

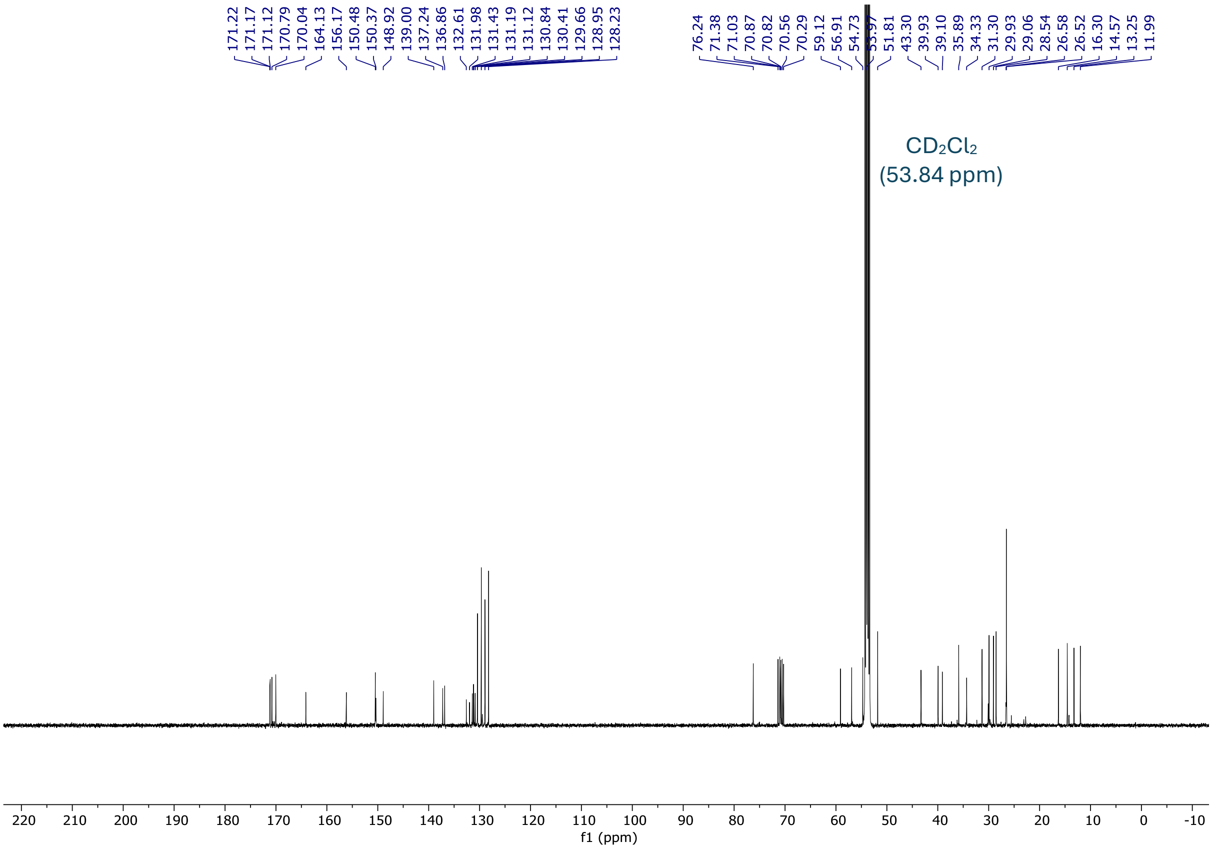

*Compound **1a**. ^{1}H NMR (500 MHz, DMSO-d_6) and ^{13}C NMR (126 MHz, DMSO-d_6)*

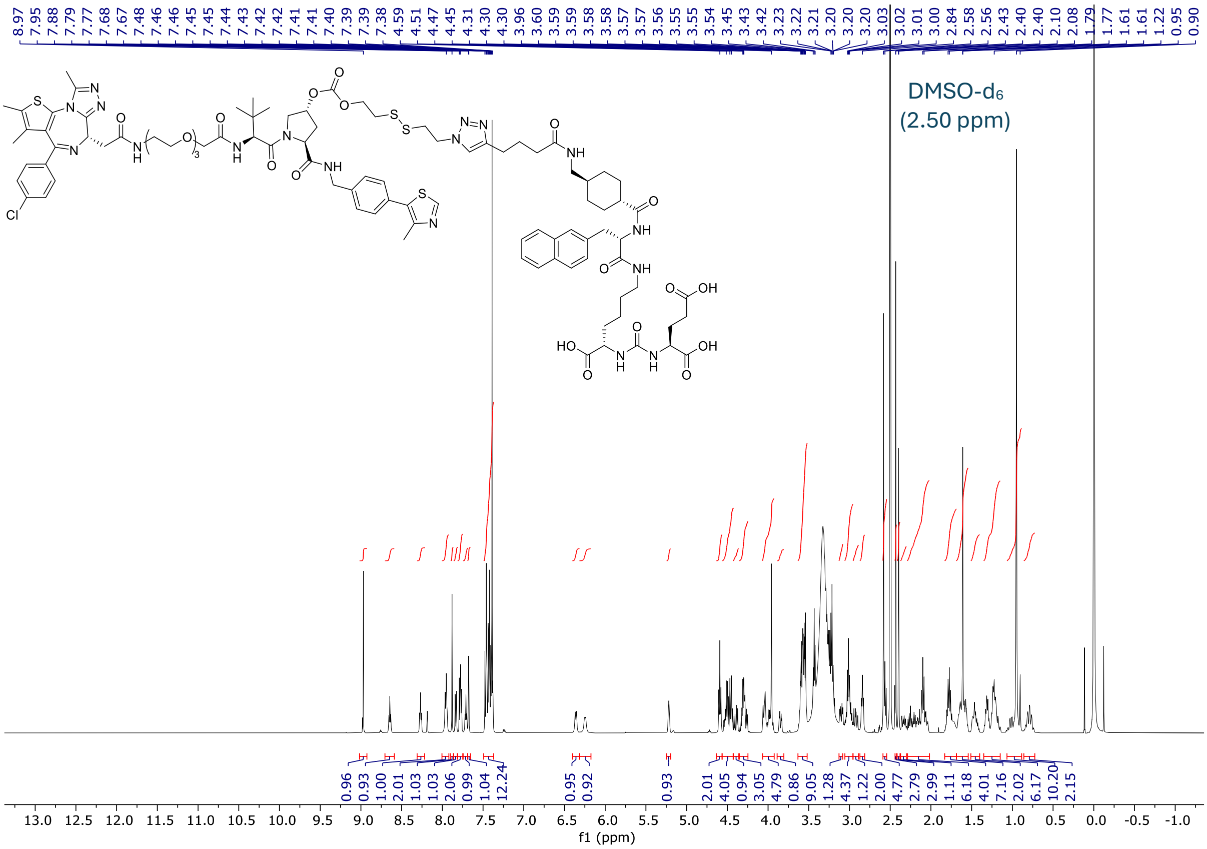

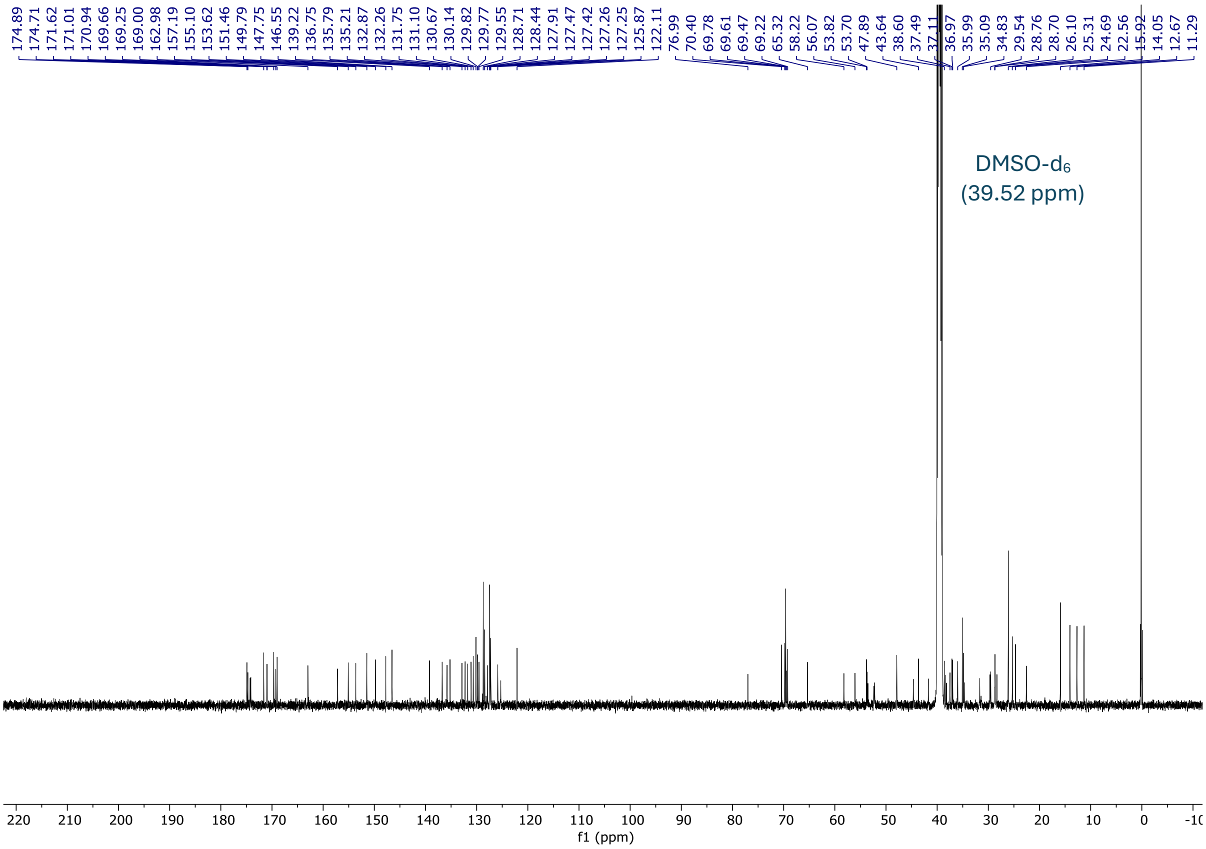

Compound ***1b****. ¹H NMR (500 MHz, DMSO-d₆) and ¹³C NMR (126 MHz, DMSO-d₆)*

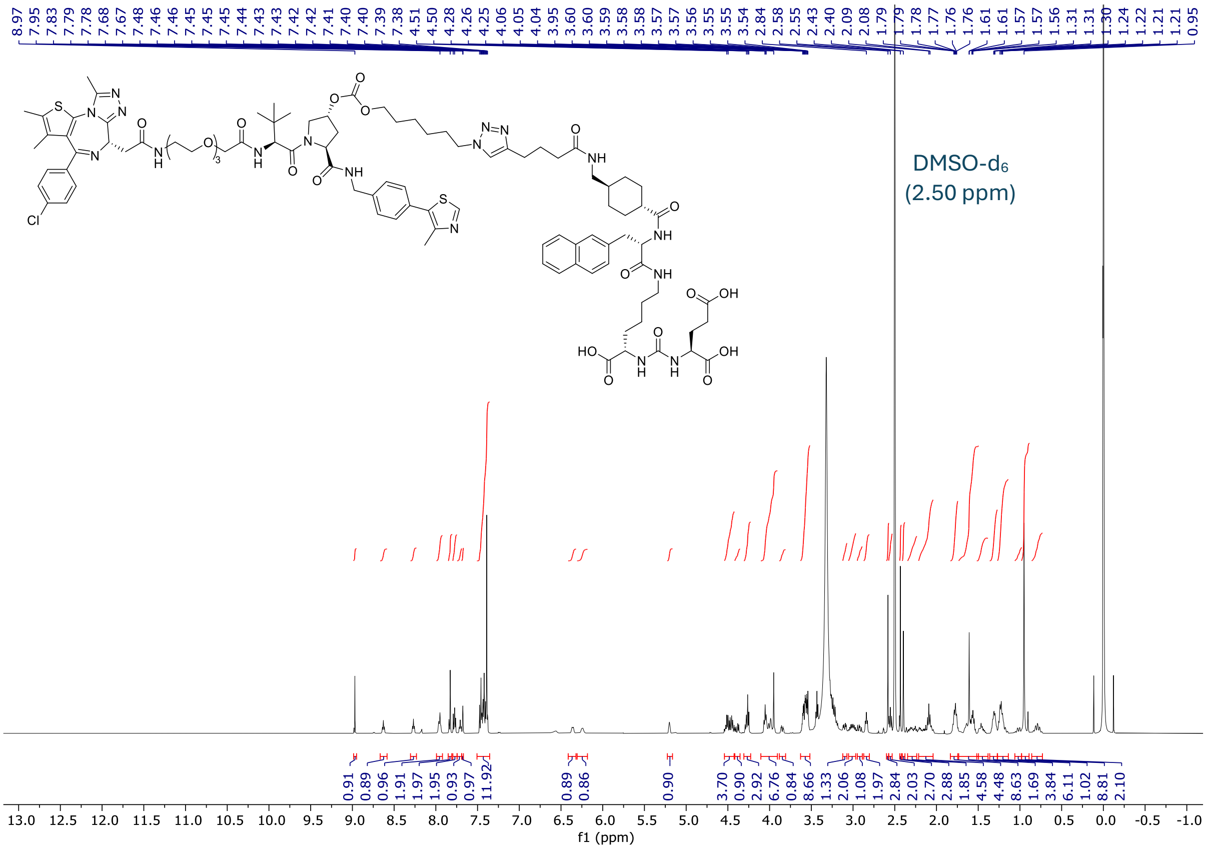

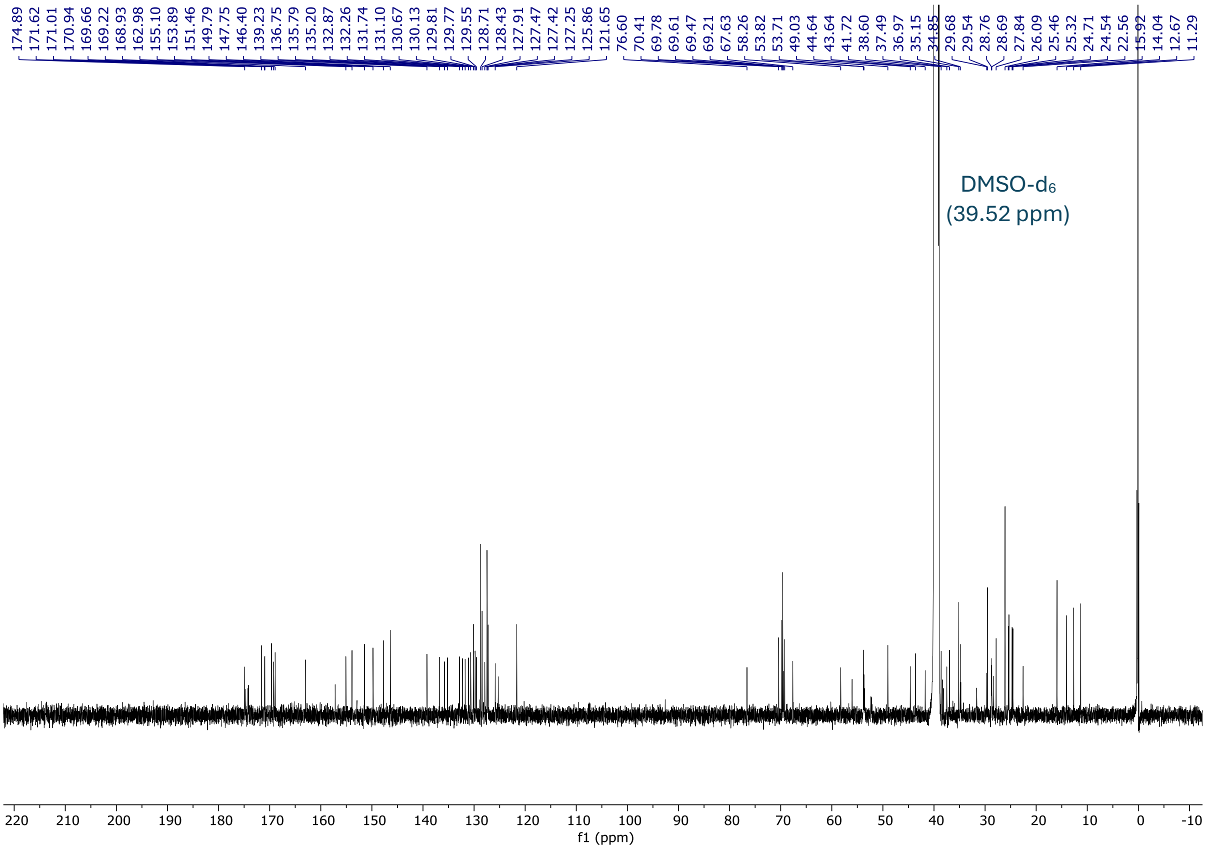

*Compound **2a**. ¹H NMR (500 MHz, DMSO-d₆) and ¹³C NMR (126 MHz, DMSO-d₆)*

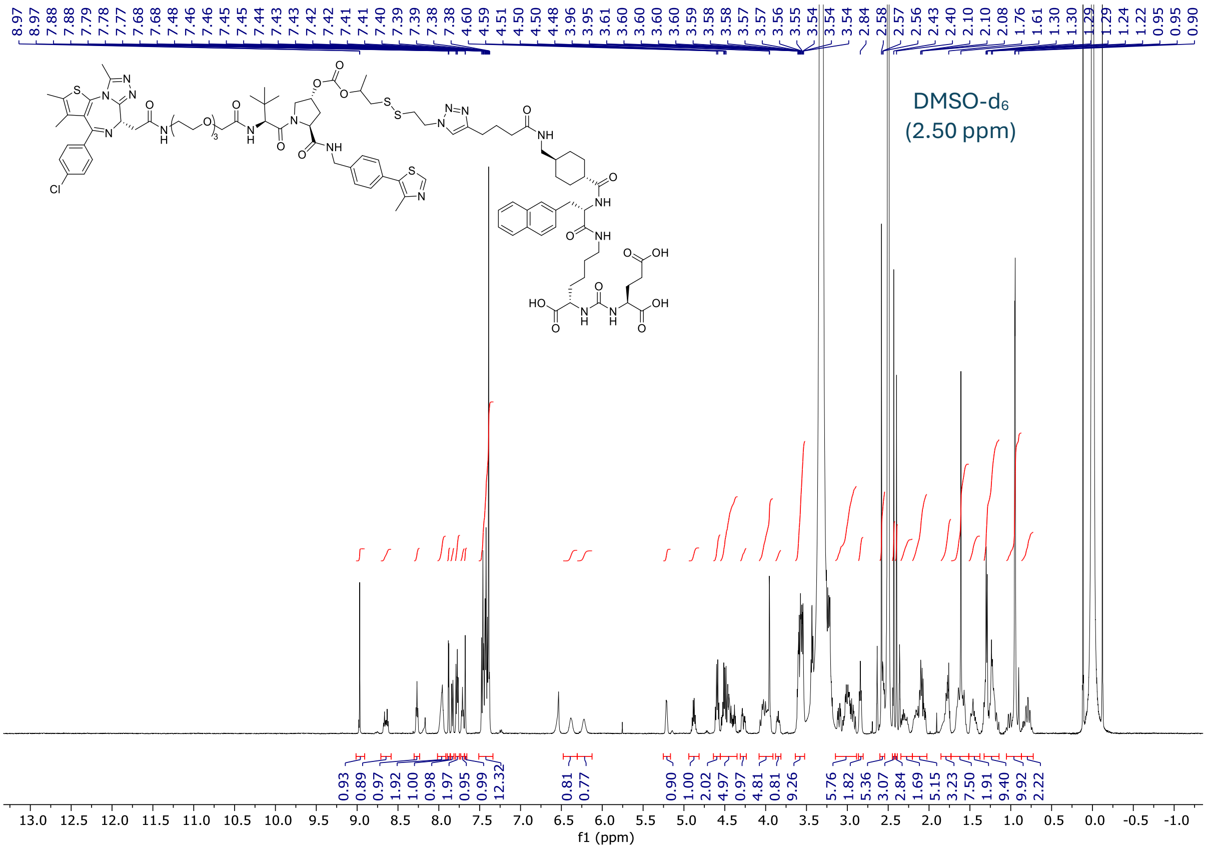

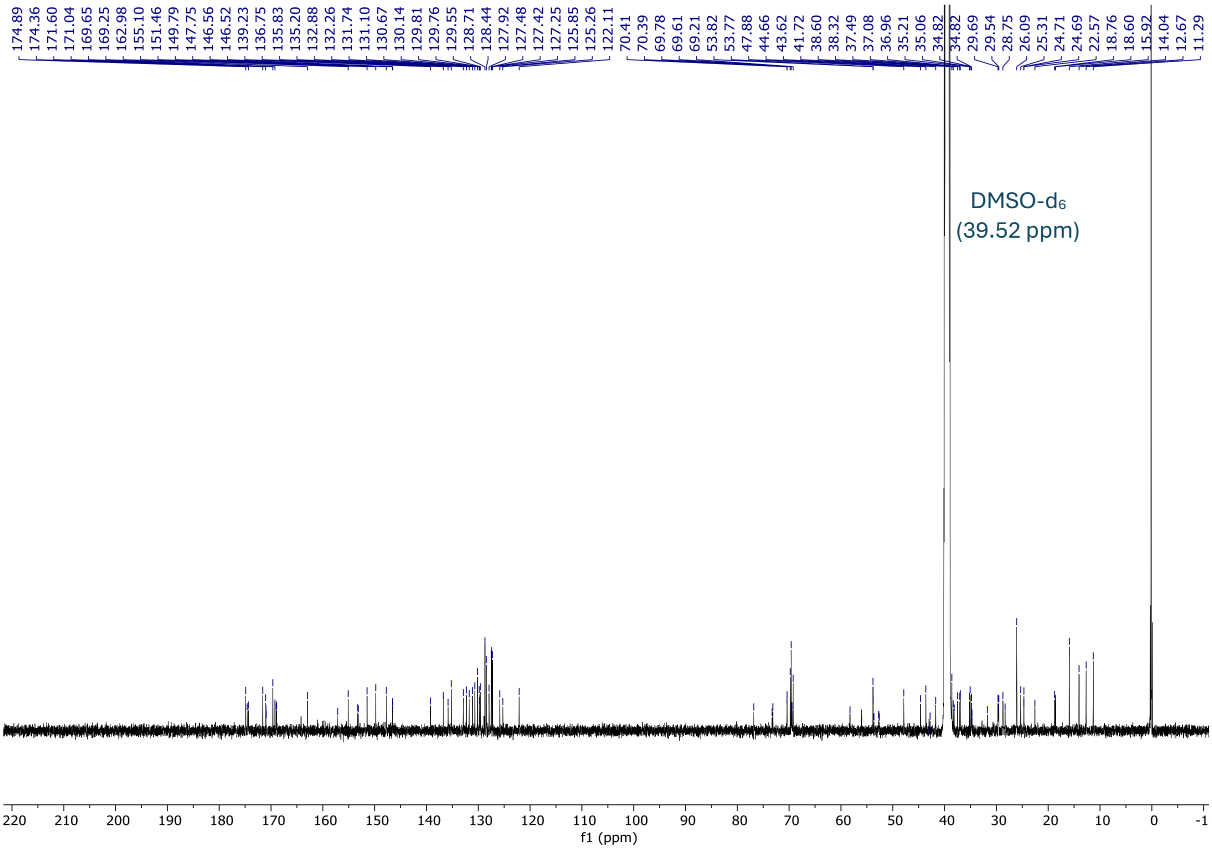

*Compound **2b**. ^{1}H NMR (500 MHz, DMSO-d_6) and ^{13}C NMR (126 MHz, DMSO-d_6)*

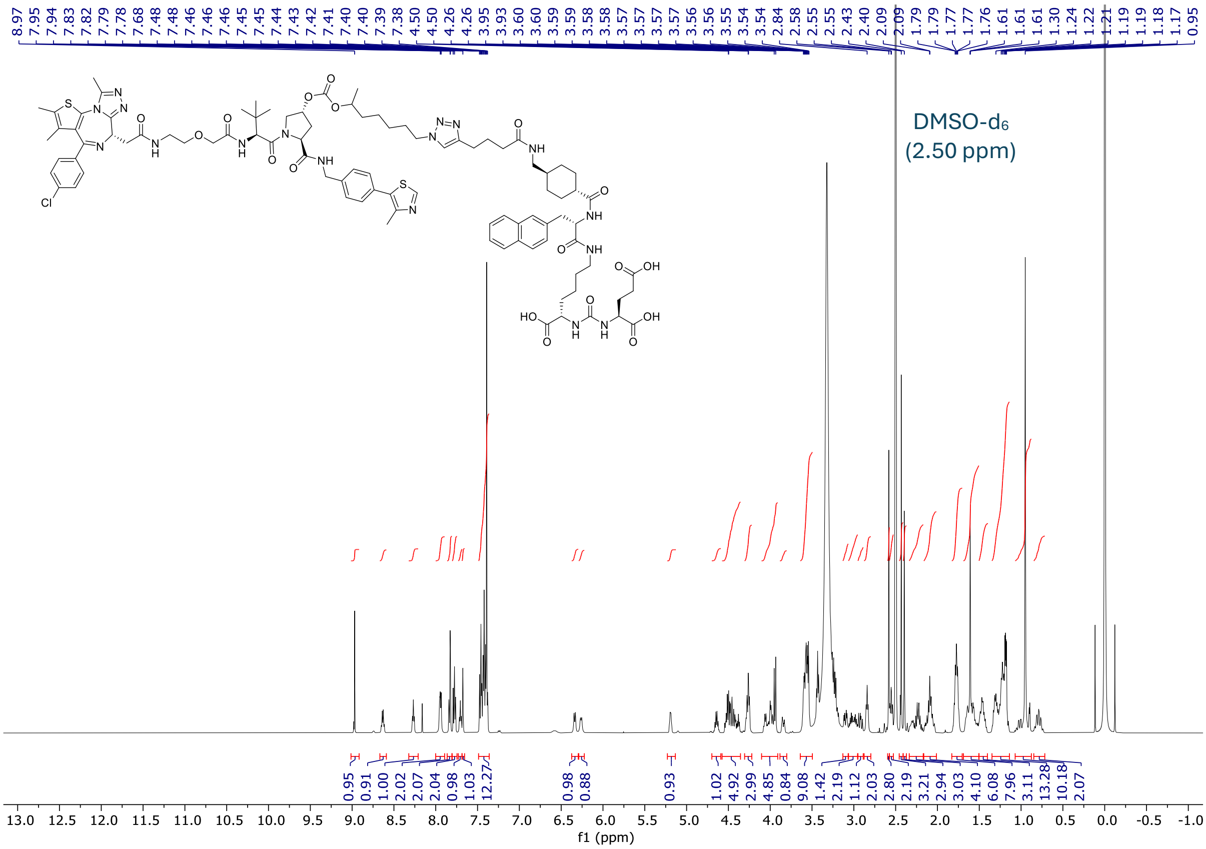

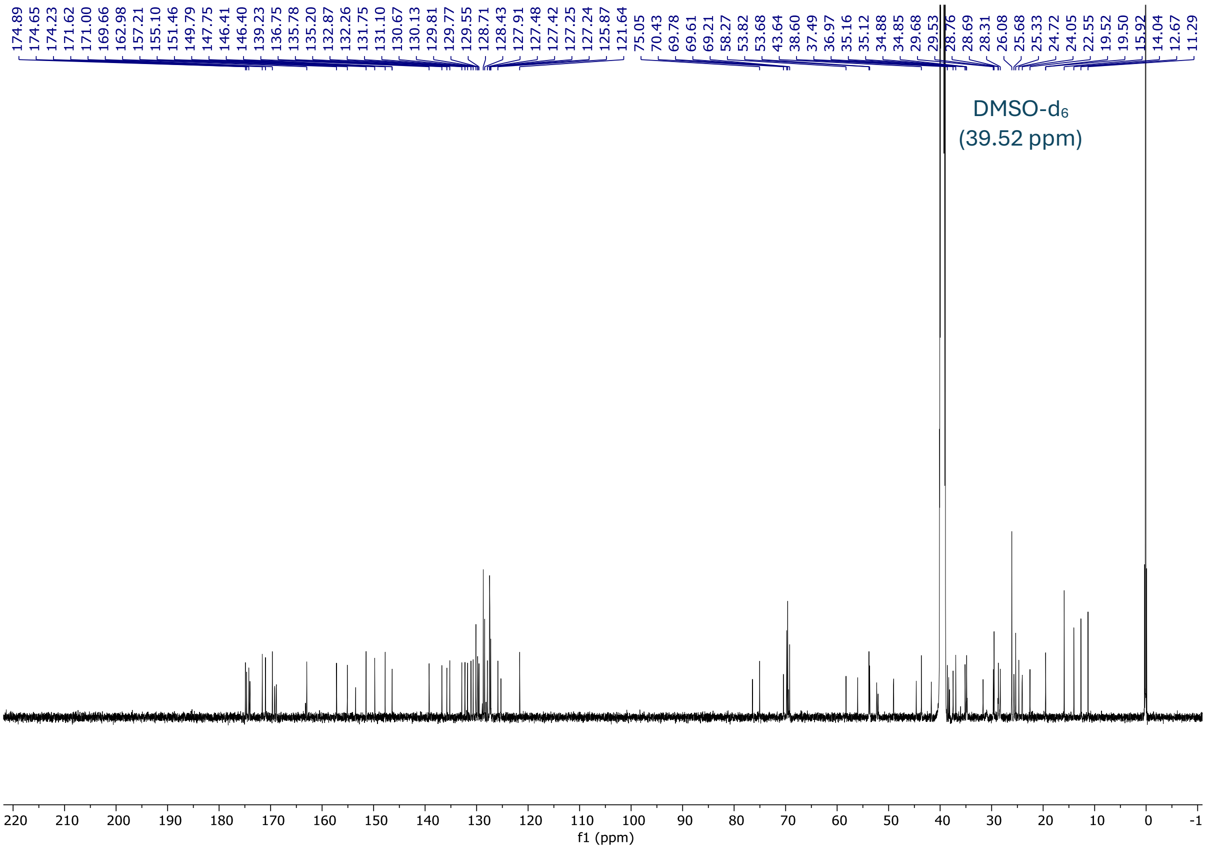

*Compound **3a**. ^{1}H NMR (500 MHz, DMSO-d_6) and ^{13}C NMR (126 MHz, DMSO-d_6)*

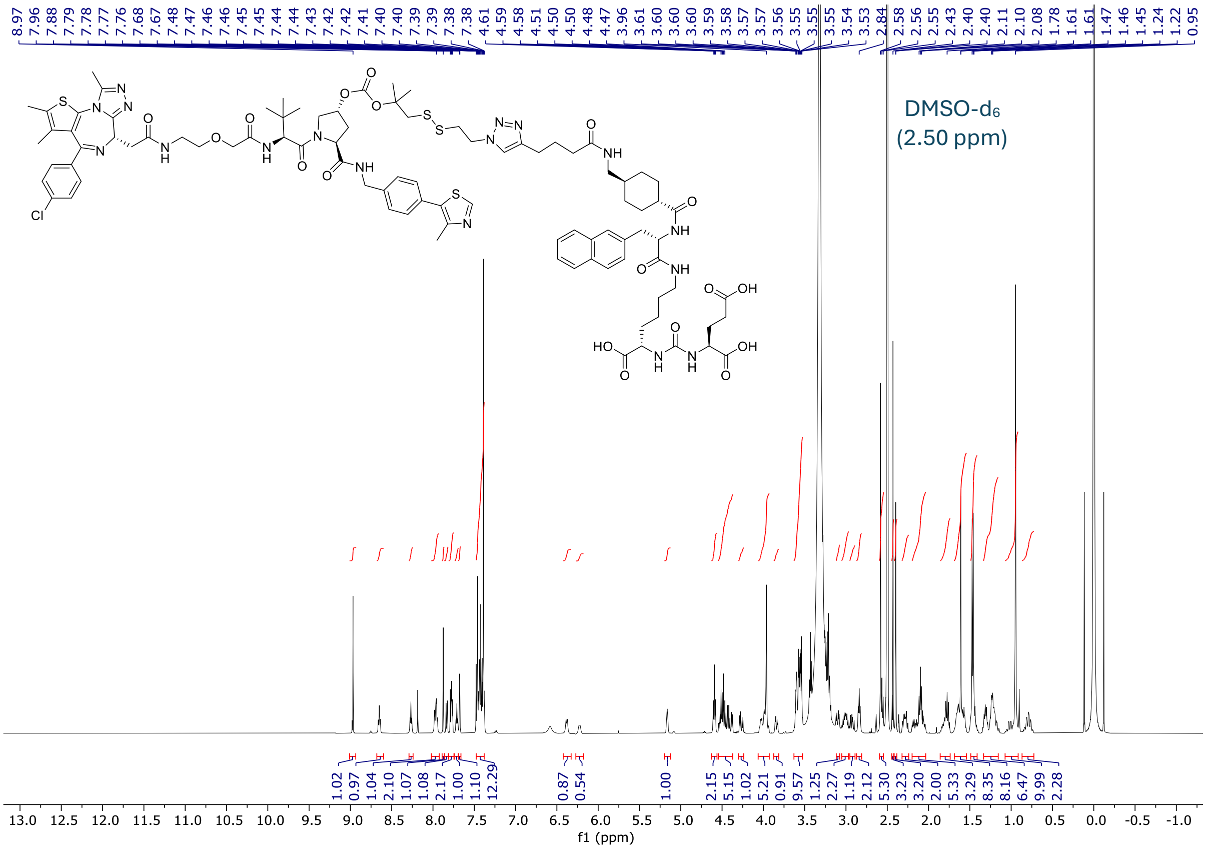

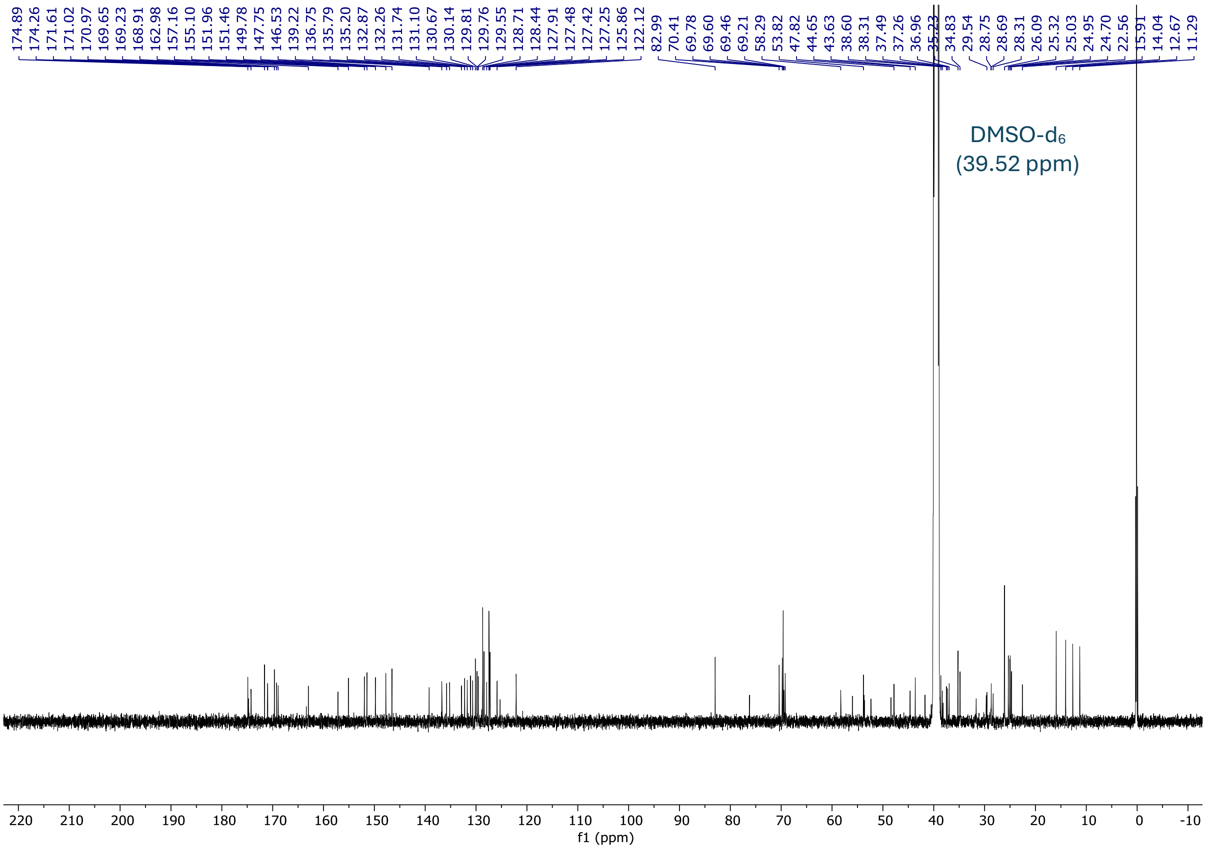

*Compound **3b**. ^{1}H NMR (500 MHz, DMSO-d_6) and ^{13}C NMR (126 MHz, DMSO-d_6)*

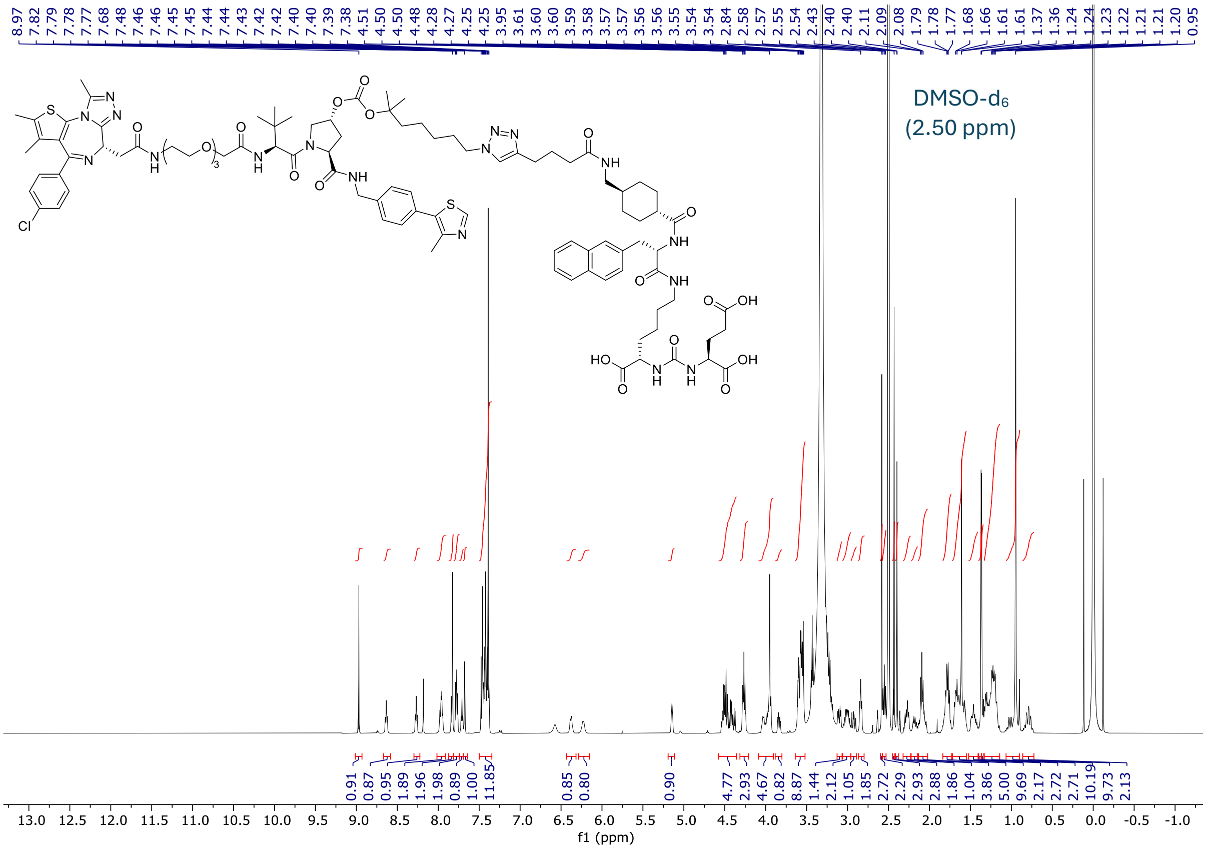

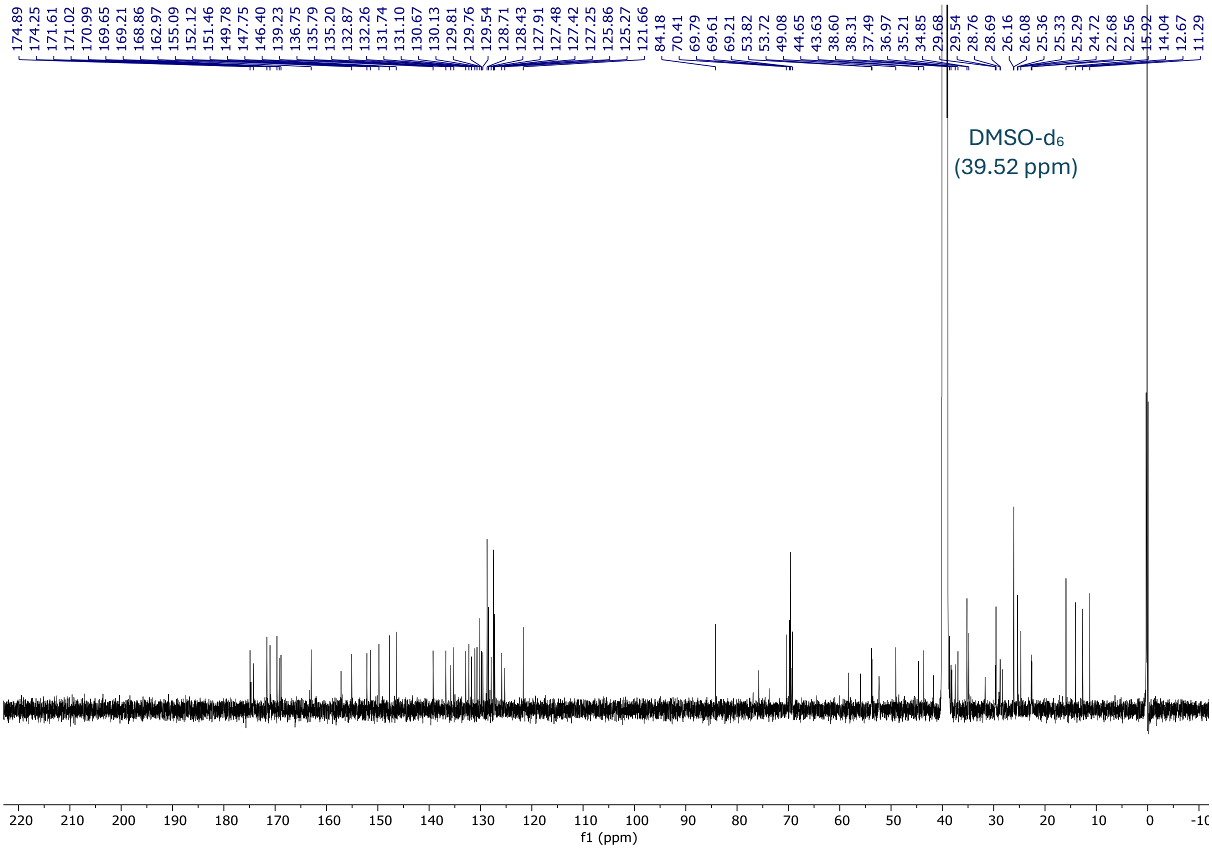

Compound **4a**. ^{1}H NMR (500 MHz, DMSO-d_6) and ^{13}C NMR (126 MHz, DMSO-d_6)

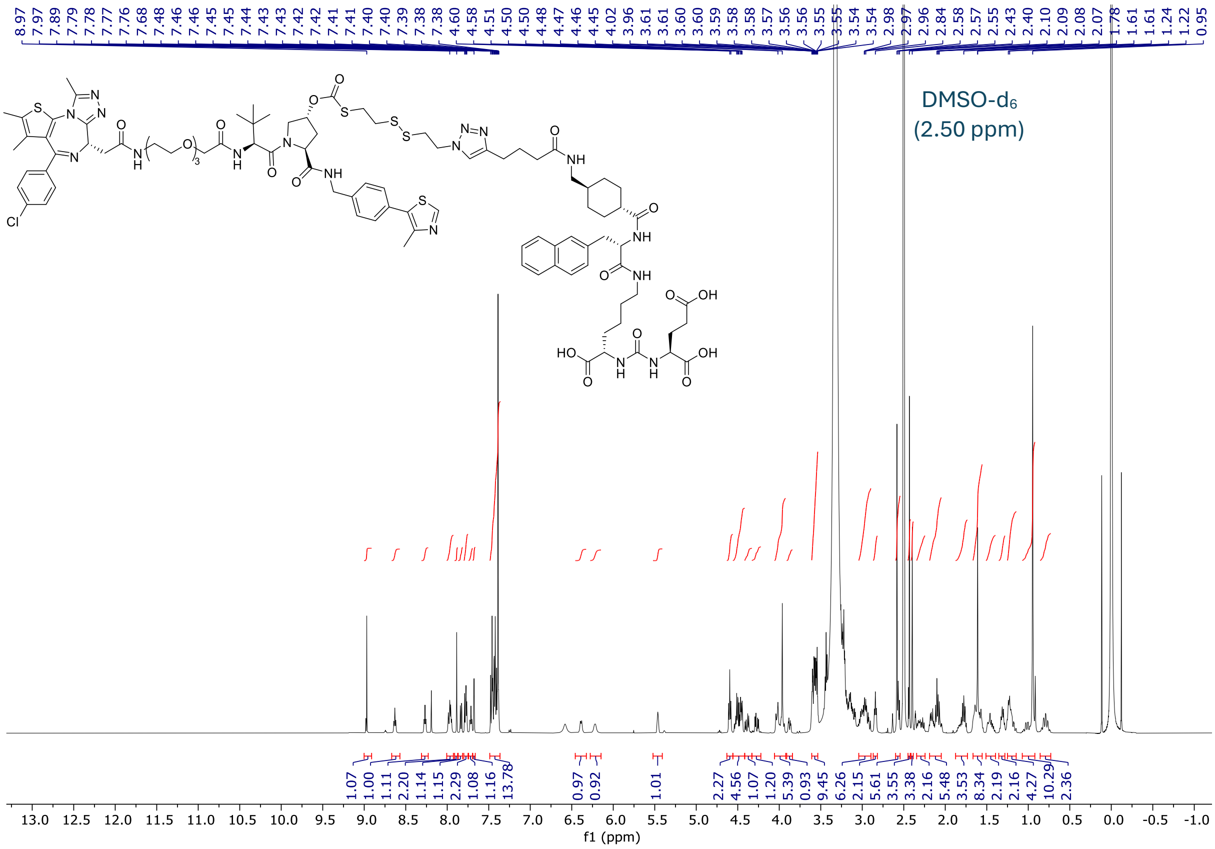

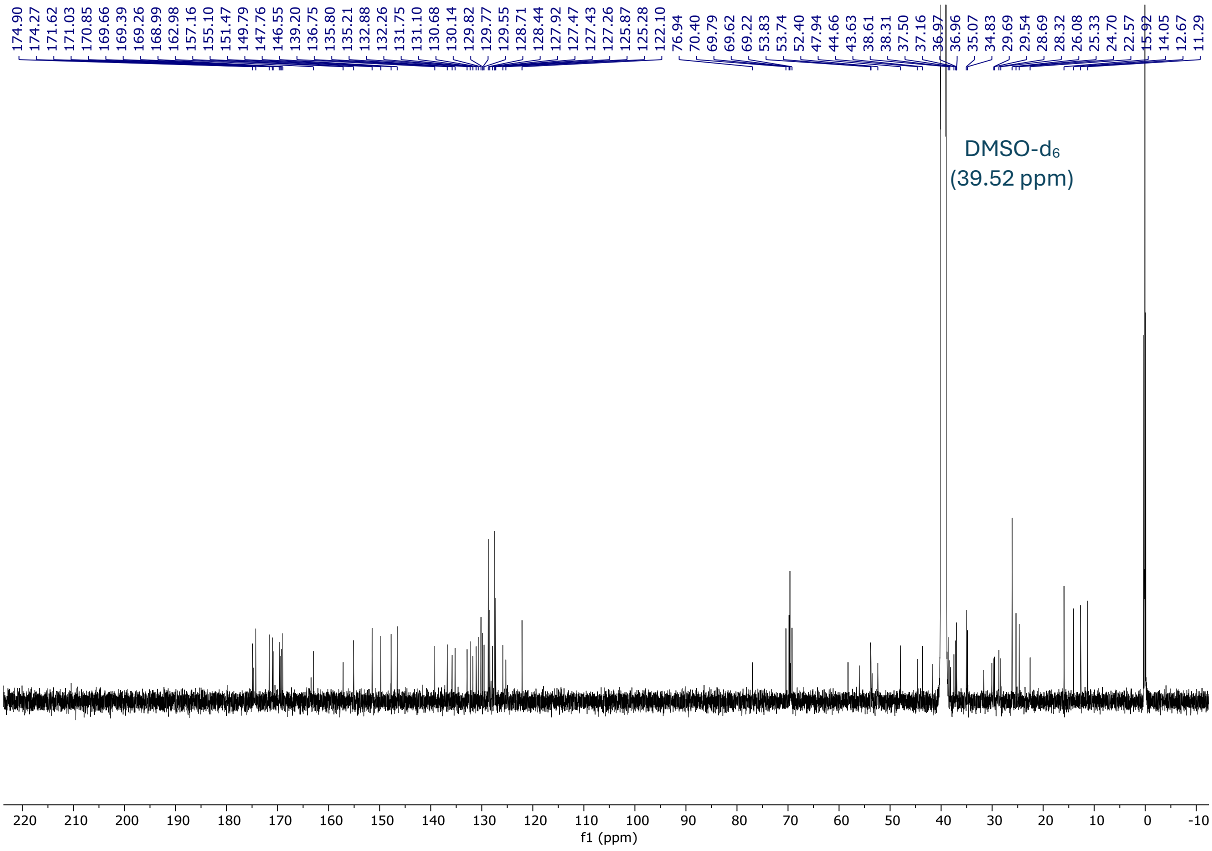

Compound ***4b****.* *^{1}H NMR (500 MHz, DMSO-d_6) and ^{13}C NMR (126 MHz, DMSO-d_6)*

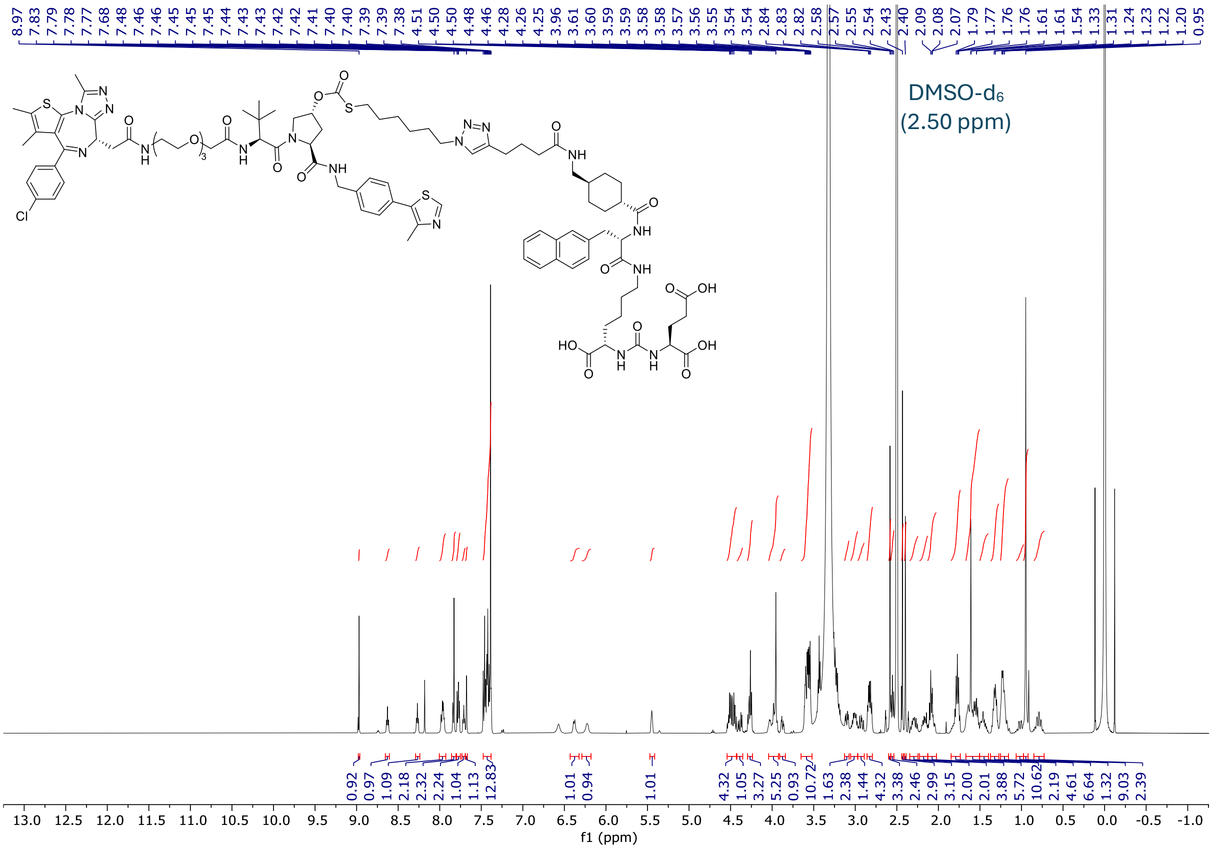

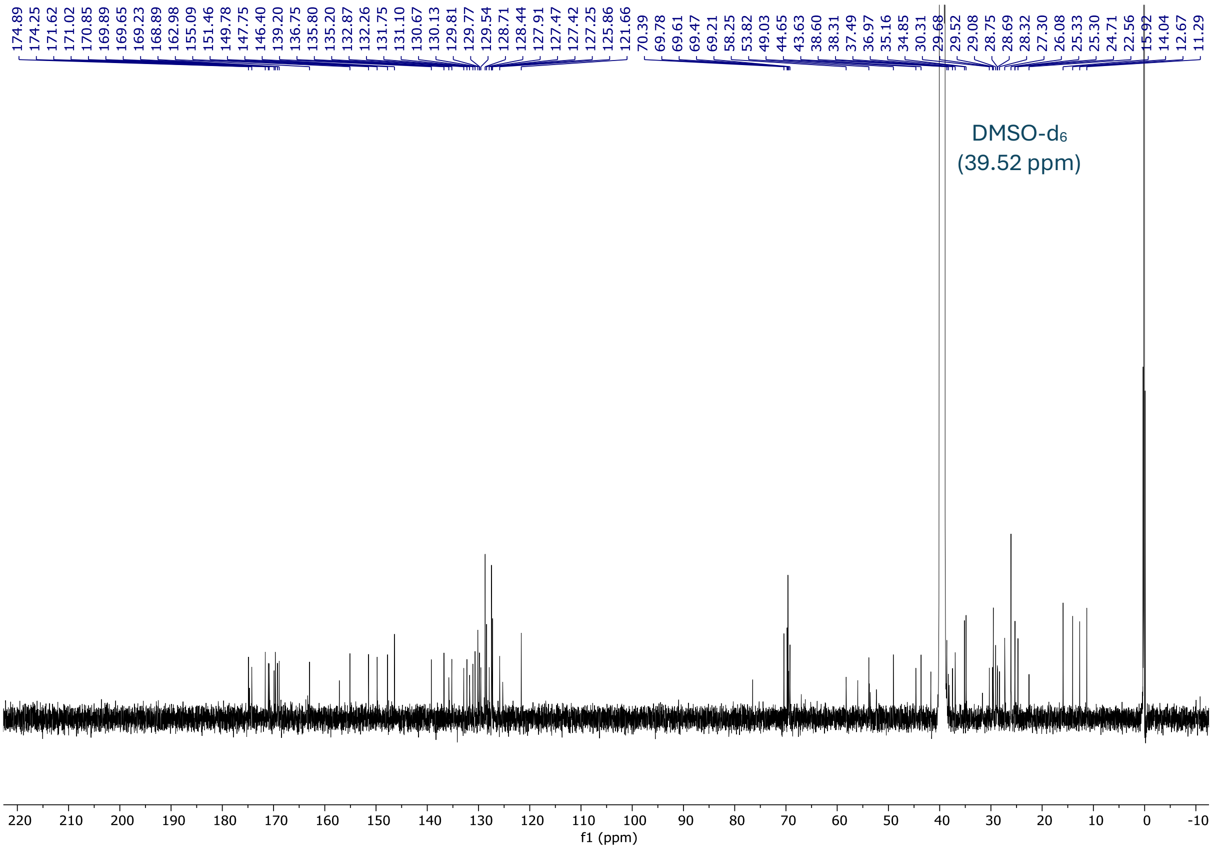

*Compound **S34**. ^{1}H NMR (400 MHz, Methanol-d_4) and ^{13}C NMR (126 MHz, Methanol-d_4)*

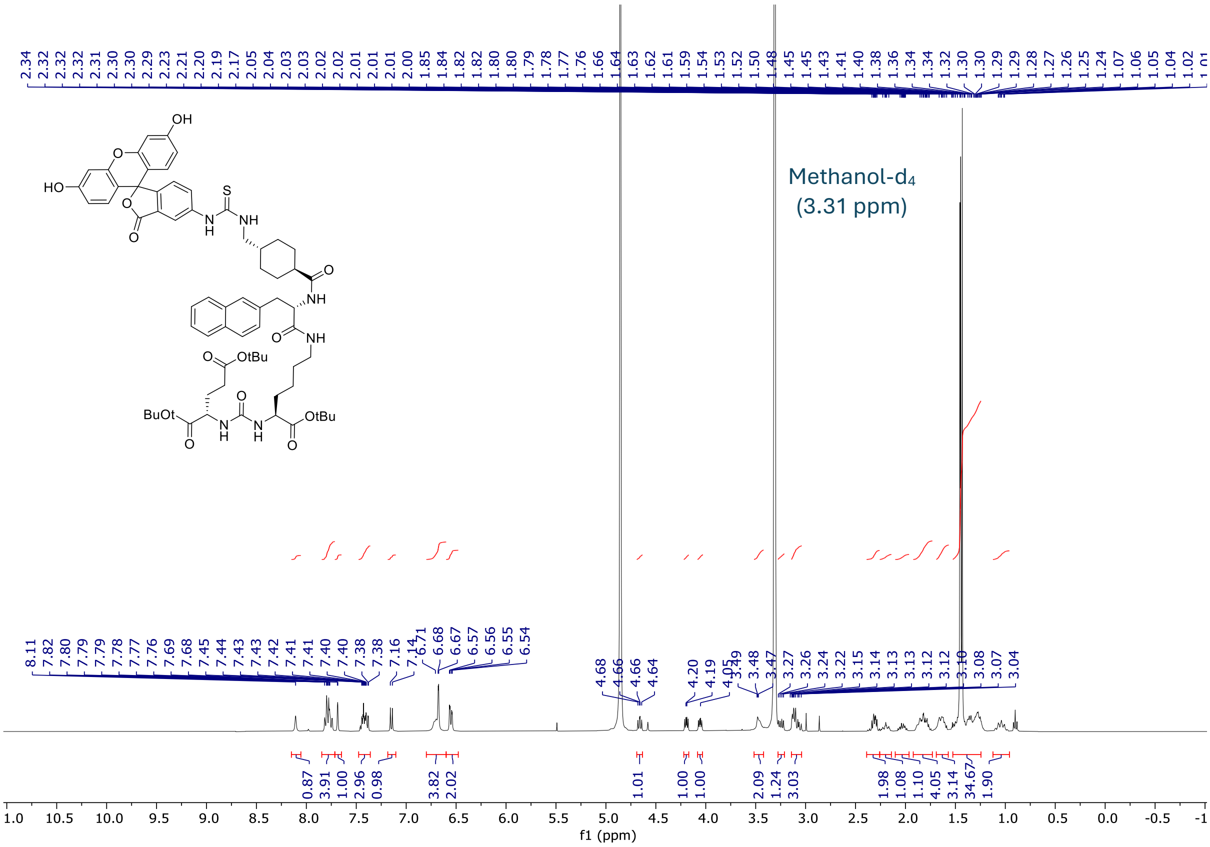

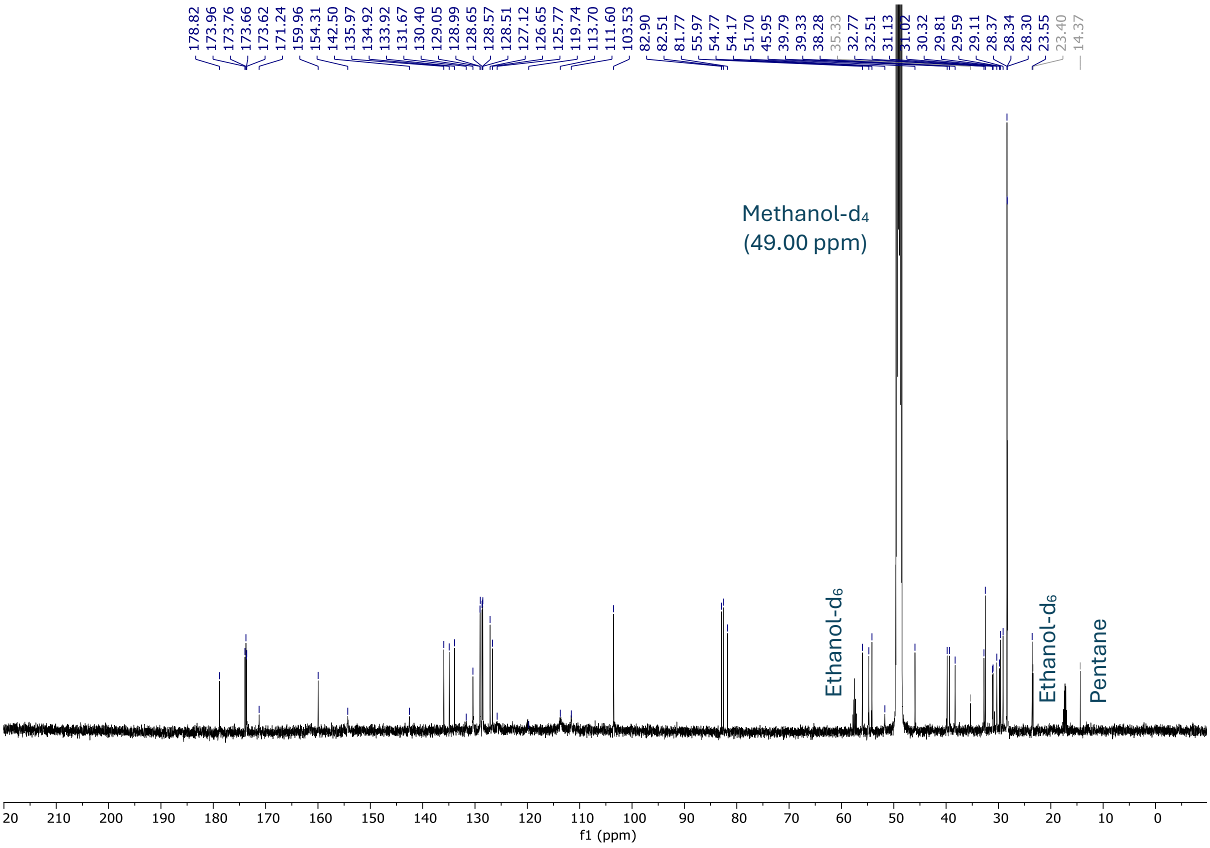

*Compound **PSMA-FITC**. ^{1}H NMR (500 MHz, DMSO-d_6) and ^{13}C NMR (126 MHz, DMSO-d_6)*

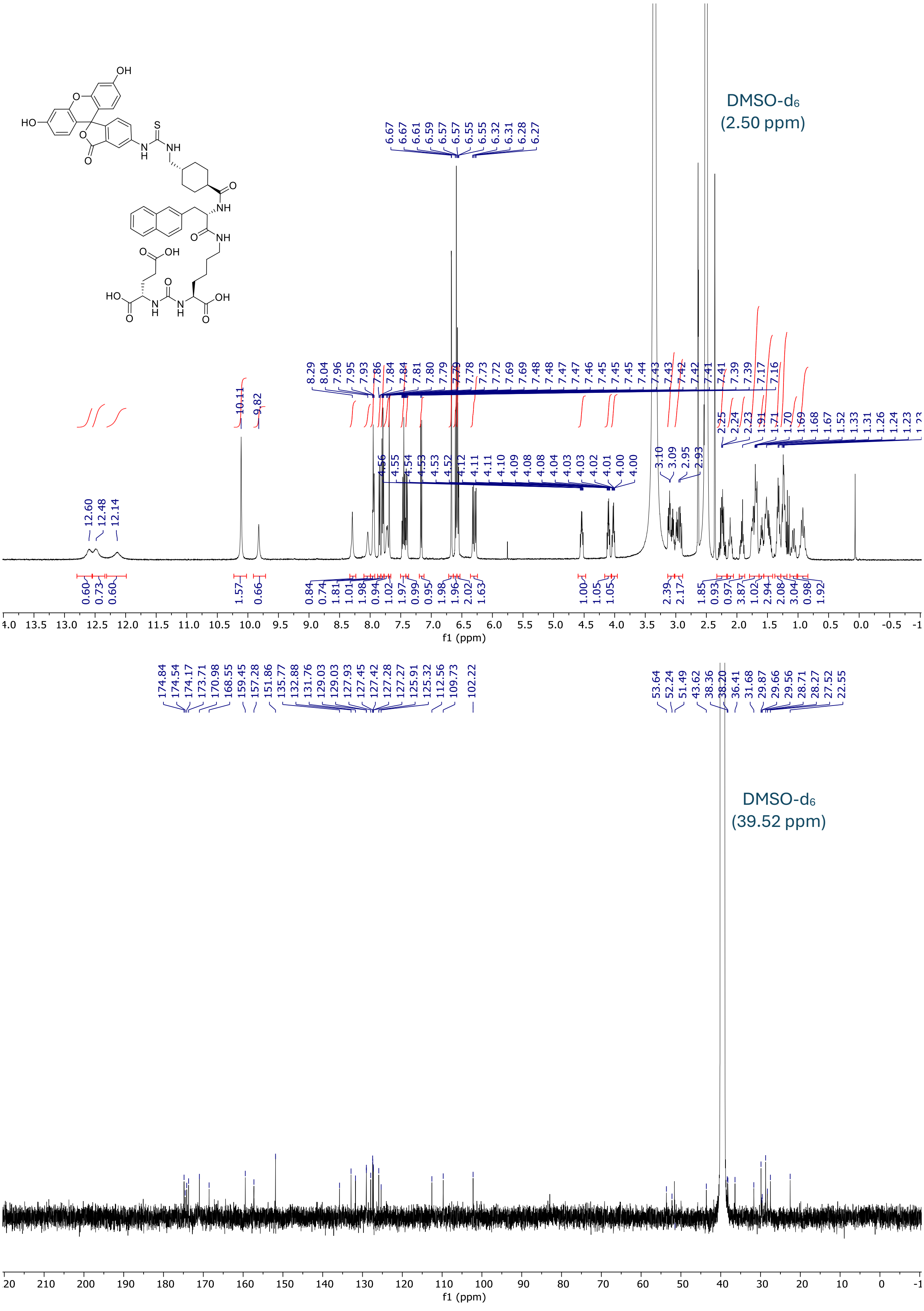

Compound **PSMA-Cbz**. ^{1}H NMR (500 MHz, DMSO-d_6) and ^{13}C NMR (126 MHz, DMSO-d_6)

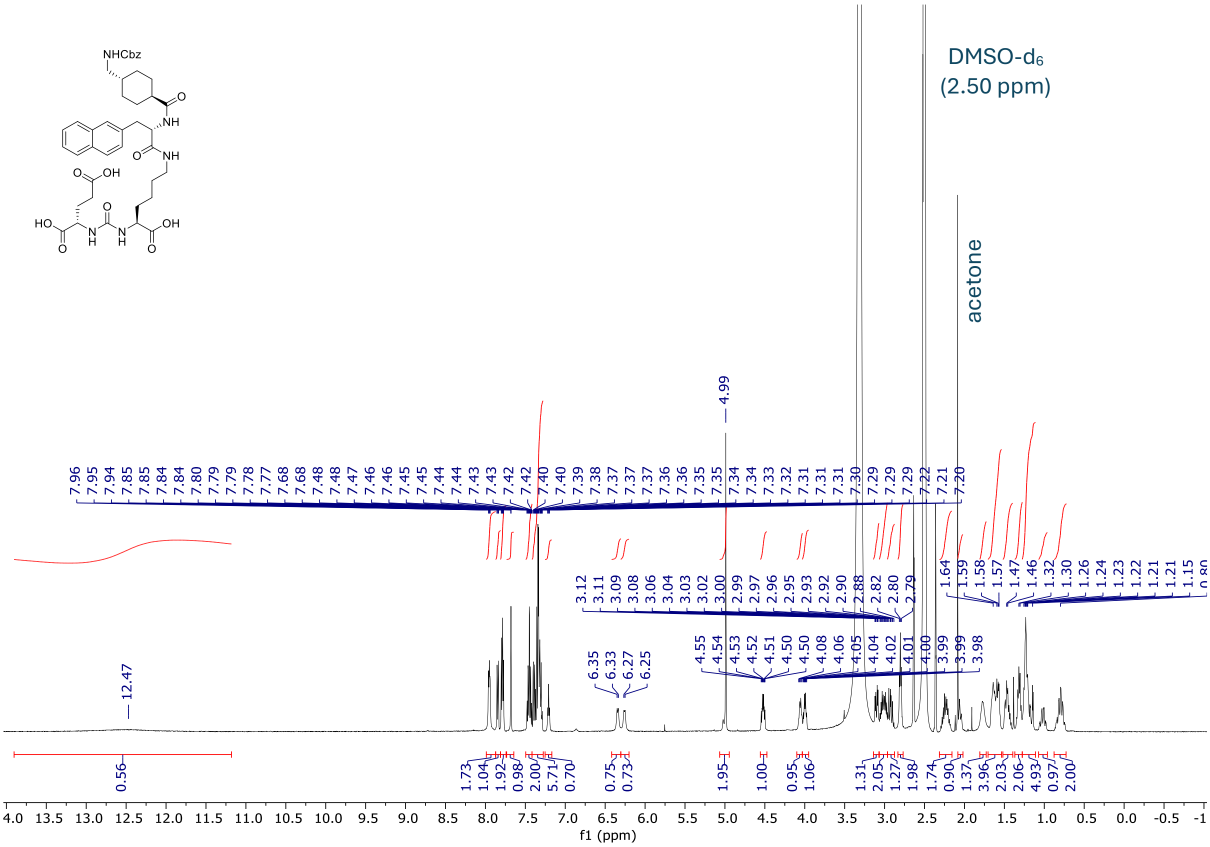

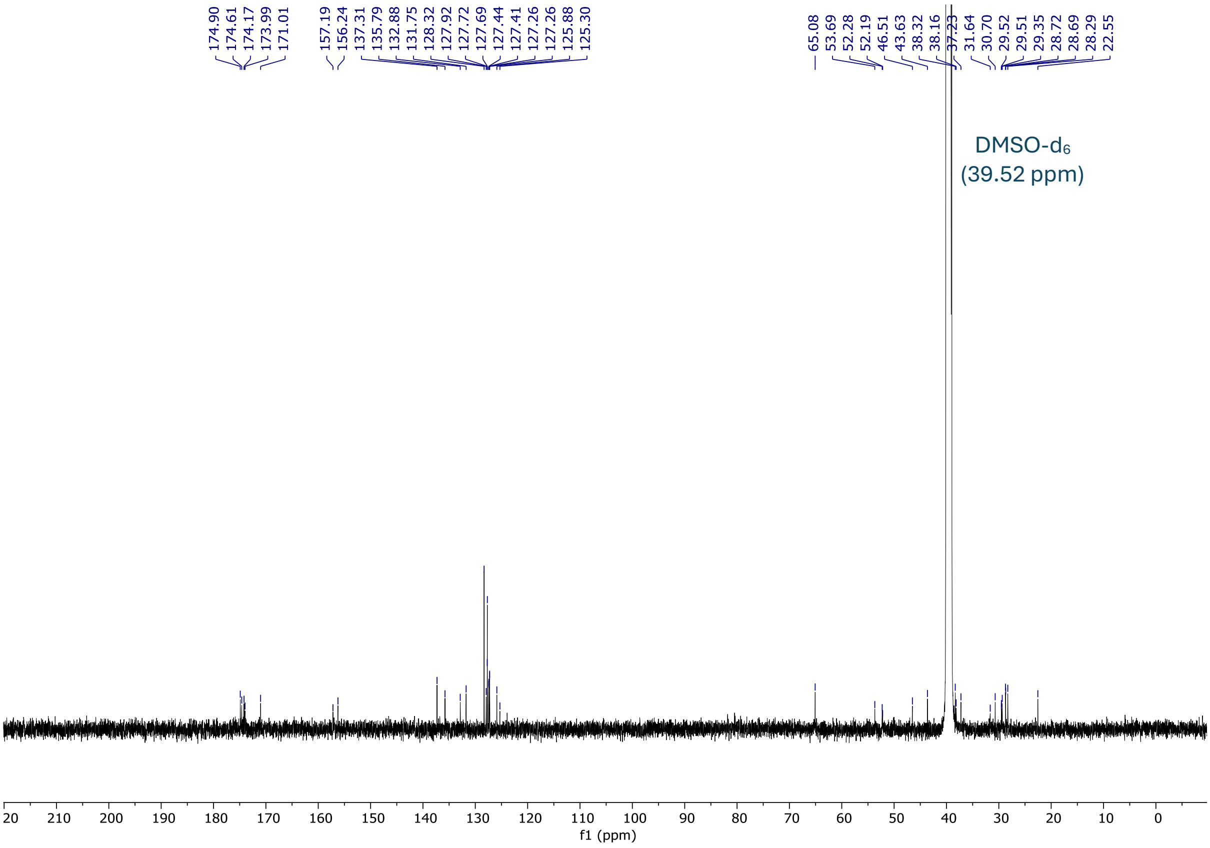

*Compound **S36**. ^1H NMR (500 MHz, DMSO-d_6) and ^{13}C NMR (126 MHz, DMSO-d_6)*

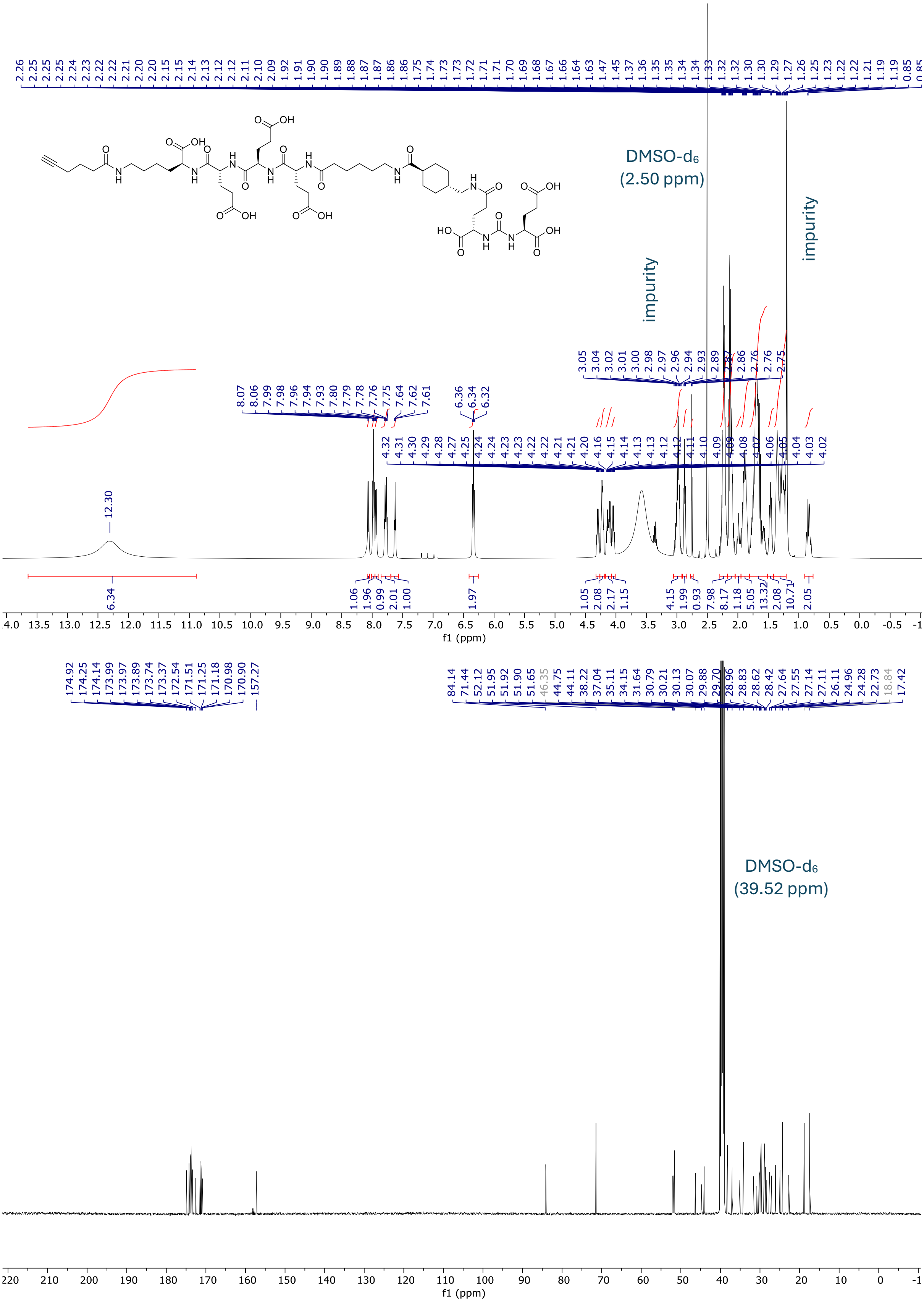

Compound ***5a***. *^{1}H NMR (500 MHz, DMSO-d6) and ^{13}C NMR (126 MHz, DMSO-d6)*

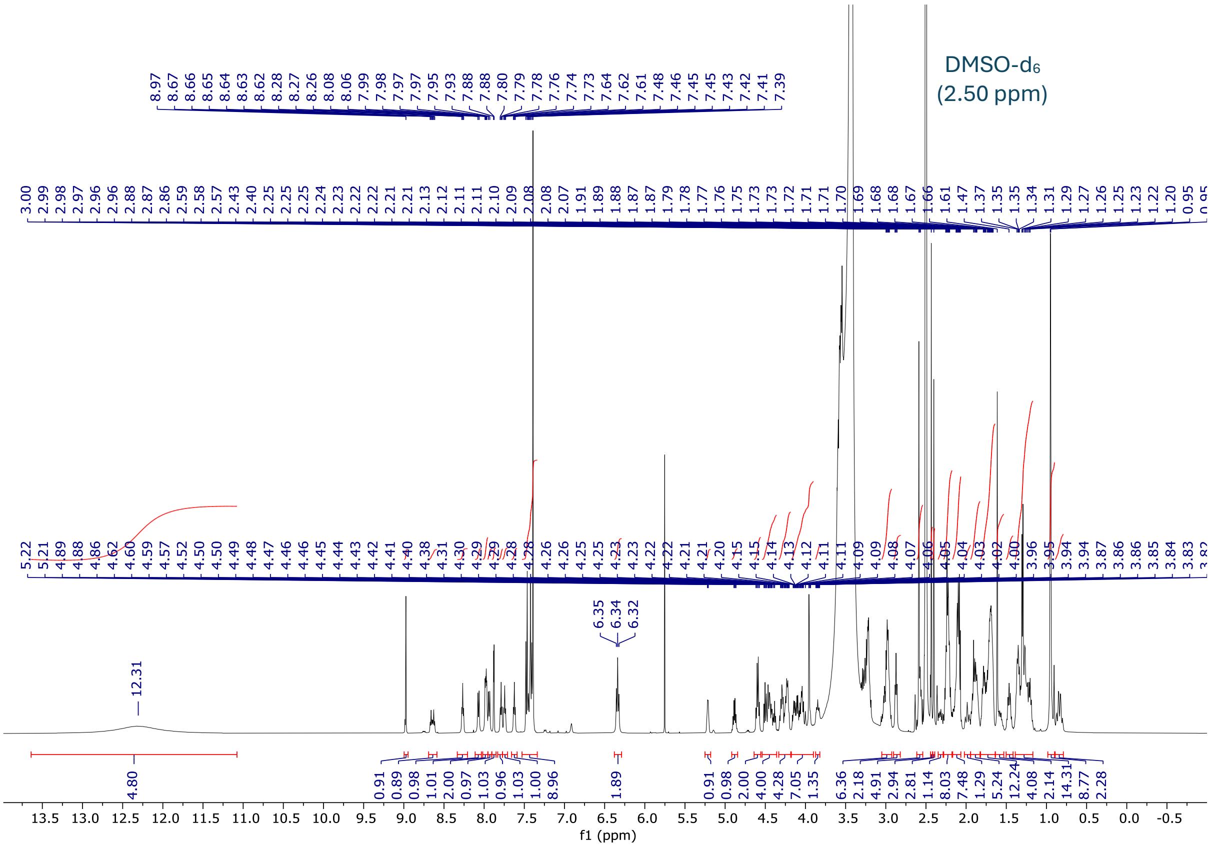

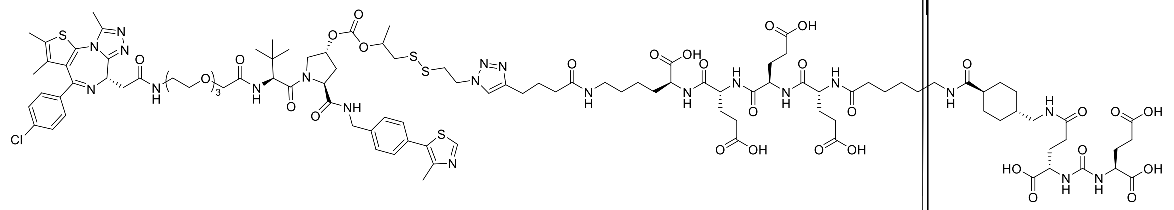

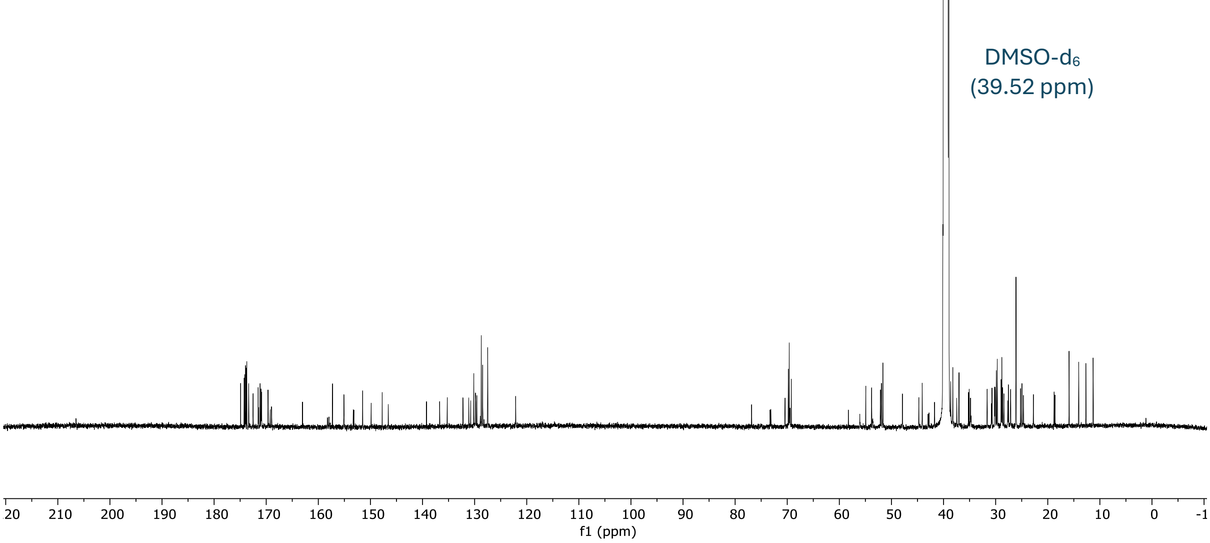

Compound **5b**. ^{1}H *NMR (500 MHz, DMSO-d$_6$) and* ^{13}C *NMR (126 MHz, DMSO-d$_6$)*

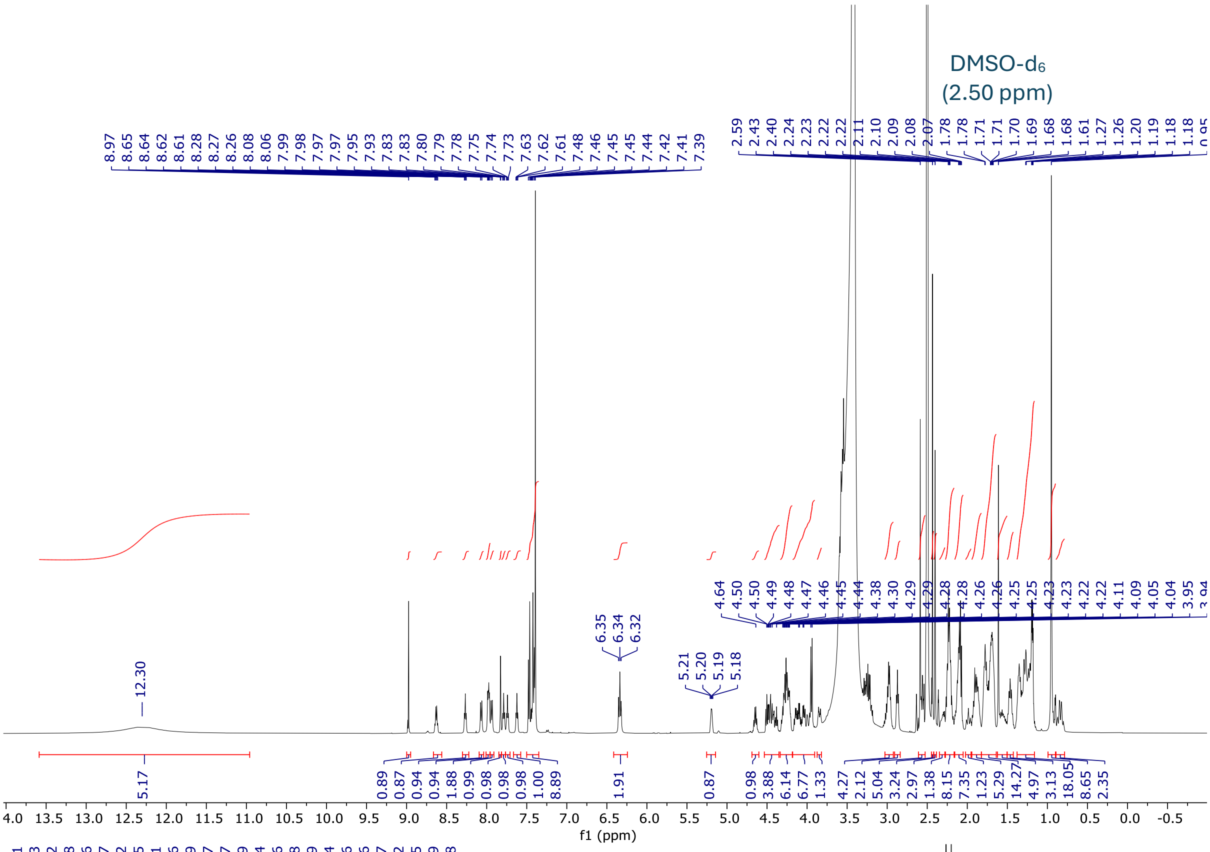

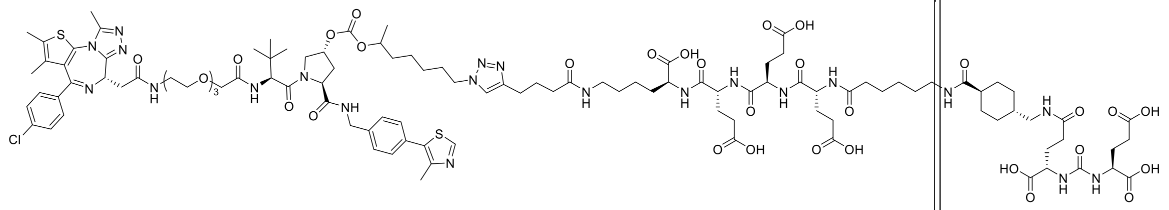

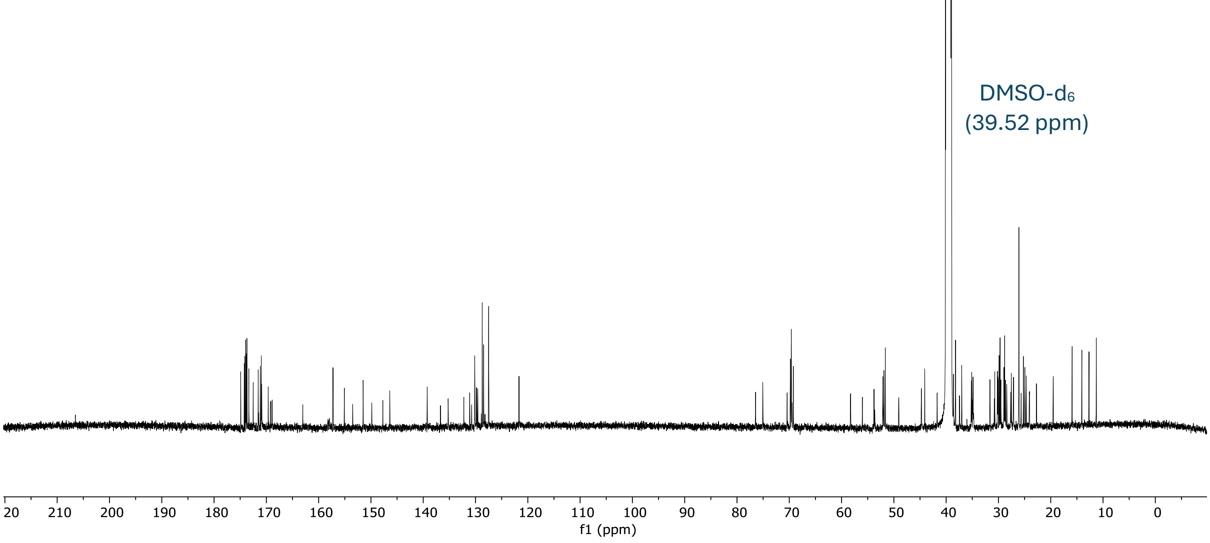

6. References

[1] Y.-J. Kang, L. Pan, Y. Liu, Z. Rong, J. Liu, F. Liu, “GEPIA3: Enhanced drug sensitivity and interaction network analysis for cancer research” *Nucleic Acids Res.* **2025**, *53*, W283–W290.

[2] M. Schäfer, U. Bauder-Wüst, M. Roscher, L. Motlová, Z. Kutilová, Y. Remde, K. D. Klika, J. Graf, C. Bařinka, M. Benešová-Schäfer, “Structure–Activity Relationships and Biological Insights into PSMA-617 and Its Derivatives with Modified Lipophilic Linker Regions” *ACS Omega* **2025**, *10*, 7077–7090.

[3] Z. Novakova, J. Cerny, C. J. Choy, J. R. Nedrow, J. K. Choi, J. Lubkowski, C. E. Berkman, C. Barinka, “Design of composite inhibitors targeting glutamate carboxypeptidase II: the importance of effector functionalities” *FEBS J.* **2016**, *283*, 130–143.

[4] K. Kopka, M. Benešová, C. Bařinka, U. Haberkorn, J. Babich, “Glu-Ureido–Based Inhibitors of Prostate-Specific Membrane Antigen: Lessons Learned During the Development of a Novel Class of Low-Molecular-Weight Theranostic Radiotracers” *Journal of Nuclear Medicine* **2017**, *58*, 17S-26S.

[5] N. M. O’Boyle, M. Banck, C. A. James, C. Morley, T. Vandermeersch, G. R. Hutchison, “Open Babel: An open chemical toolbox” *J. Cheminform.* **2011**, *3*, 33.

[6] K. Vanommeslaeghe, A. D. MacKerell, “Automation of the CHARMM General Force Field (CGenFF) I: Bond Perception and Atom Typing” *J. Chem. Inf. Model.* **2012**, *52*, 3144–3154.

[7] K. Vanommeslaeghe, E. Hatcher, C. Acharya, S. Kundu, S. Zhong, J. Shim, E. Darian, O. Guvench, P. Lopes, I. Vorobyov, “CHARMM general force field: A force field for drug-like molecules compatible with the CHARMM all-atom additive biological force fields” *J. Comput. Chem.* **2010**, *31*, 671–690.

[8] A. Kumar, O. Yoluk, A. D. MacKerell, “FFParam: Standalone package for CHARMM additive and Drude polarizable force field parametrization of small molecules” *J. Comput. Chem.* **2020**, *41*, 958–970.

[9] B. R. Brooks, C. L. Brooks, A. D. Mackerell, L. Nilsson, R. J. Petrella, B. Roux, Y. Won, G. Archontis, C. Bartels, S. Boresch, A. Caflisch, L. Caves, Q. Cui, A. R. Dinner, M. Feig, S. Fischer, J. Gao, M. Hodoscek, W. Im, K. Kuczera, T. Lazaridis, J. Ma, V. Ovchinnikov, E. Paci, R. W. Pastor, C. B. Post, J. Z. Pu, M. Schaefer, B. Tidor, R. M. Venable, H. L. Woodcock, X. Wu, W. Yang, D. M. York, M. Karplus, “CHARMM: The biomolecular simulation program” *J. Comput. Chem.* **2009**, *30*, 1545–1614.

[10] M. J. Frisch, G. W. Trucks, H. B. Schlegel, G. E. Scuseria, M. A. Robb, J. R. Cheeseman, G. Scalmani, V. Barone, G. A. Petersson, H. Nakatsuji, X. Li, M. Caricato, A. V. Marenich, J. Bloino, B. G. Janesko, R. Gomperts, B. Mennucci, H. P. Hratchian, J. V. Ortiz, A. F. Izmaylov, J. L. Sonnenberg, D. Williams-Young, F. Ding, F. Lipparini, F. Egidi, J. Goings, B. Peng, A. Petrone, T. Henderson, D. Ranasinghe, V. G. Zakrzewski, J. Gao, N. Rega, G. Zheng, W. Liang, M. Hada, M. Ehara, K. Toyota, R. Fukuda, J. Hasegawa, M. Ishida, T. Nakajima, Y. Honda, O. Kitao, H. Nakai, T. Vreven, K. Throssell, J. A. , Jr. Montgomery, J. E. Peralta, F. Ogliaro, M. J. Bearpark, J. J. Heyd, E. N. Brothers, K. N. Kudin, V. N. Staroverov, T. A. Keith, R. Kobayashi, J. Normand, K. Raghavachari, A. P. Rendell, J. C. Burant, S. S. Iyengar, J. Tomasi, M. Cossi, J. M. Millam, M. Klene, C. Adamo, R. Cammi, J. W. Ochterski, R. L. Martin, K. Morokuma, O. Farkas, J. B. Foresman, D. J. Fox, **2016**, Gaussian, Inc., Wallingford CT.

[11] E. P. Raman, C. Tetsassi, J. Lemkul, “cgenff_charmm2gmx_py3_nx2.py,” can be found under http://mackerell.umaryland.edu/charmm_ff.shtml#gromacs,” **2019** (accessed: 2025).

[12] R. B. Best, X. Zhu, J. Shim, P. E. M. Lopes, J. Mittal, M. Feig, A. D. MacKerell, "Optimization of the Additive CHARMM All-Atom Protein Force Field Targeting Improved Sampling of the Backbone ϕ, ψ and Side-Chain χ_1 and χ_2 Dihedral Angles" *J. Chem. Theory Comput.* **2012**, *8*, 3257–3273.

[13] M. J. Abraham, T. Murtola, R. Schulz, S. Páll, J. C. Smith, B. Hess, E. Lindahl, "GROMACS: High performance molecular simulations through multi-level parallelism from laptops to supercomputers" *SoftwareX* **2015**, *1–2*, 19–25.

[14] C. Caleman, P. J. van Maaren, M. Hong, J. S. Hub, L. T. Costa, D. van der Spoel, "Force Field Benchmark of Organic Liquids: Density, Enthalpy of Vaporization, Heat Capacities, Surface Tension, Isothermal Compressibility, Volumetric Expansion Coefficient, and Dielectric Constant" *J. Chem. Theory Comput.* **2012**, *8*, 61–74.

[15] B. Hess, H. Bekker, H. J. C. Berendsen, J. G. E. M. Fraaije, "LINCS: A linear constraint solver for molecular simulations" *J. Comput. Chem.* **1997**, *18*, 1463–1472.

[16] G. Bussi, D. Donadio, M. Parrinello, "Canonical sampling through velocity rescaling" *J. Chem. Phys.* **2007**, *126*, 014101.

[17] A. Caflisch, M. Karplus, "Acid and Thermal Denaturation of Barnase Investigated by Molecular Dynamics Simulations" *J. Mol. Biol.* **1995**, *252*, 672–708.

[18] M. N. Iannone, S. Stucchi, E. A. Turolla, C. Beretta, S. Ciceri, C. Chinello, L. Pagani, S. Todde, P. Ferraboschi, "Synthesis and automated fluorine-18 radiolabeling of new PSMA-617 derivatives with a CuAAC radiosynthetic approach" *J. Labelled Comp. Radiopharm.* **2022**, *65*, 48–62.

[19] P. Jangili, N. Kong, J. H. Kim, J. Zhou, H. Liu, X. Zhang, W. Tao, J. S. Kim, "DNA-Damage-Response-Targeting Mitochondria-Activated Multifunctional Prodrug Strategy for Self-Defensive Tumor Therapy" *Angewandte Chemie International Edition* **2022**, *61*, e202117075.

[20] A. Ciulli, M. Zengerle, K.-H. Chan, *Derivatives of 1-[(Cylopentyl or 2-Pyrrolidinyl)Carbonylaminemethyl]-4-(1,3-Thazol-5-Yl) Benzene Which Are Useful for the Treatment of Proliferative, Autoimmine or Inflammatory Diseases*, **2016**, WO2016146985A1.

[21] C. Yang, J. P. Flynn, J. Niu, "Facile Synthesis of Sequence-Regulated Synthetic Polymers Using Orthogonal SuFEx and CuAAC Click Reactions" *Angewandte Chemie International Edition* **2018**, *57*, 16194–16199.

[22] M. Abbasi, "One-Pot Tandem Synthesis of β-Trimethylsilyloxy Thioesters from Thioacids, Epoxides, and HMDS Catalyzed by Silica Gel Under Solvent-Free Conditions" *Synth. Commun.* **2013**, *43*, 1759–1765.

[23] J. R. L. Sousa, M. S. Franco, L. D. Mendes, L. A. Araújo, J. S. S. Neto, T. E. A. Frizon, V. B. dos Santos, E. Carasek, S. Saba, J. Rafique, A. L. Braga, "KIO_3-catalyzed selective oxidation of thiols to disulfides in water under ambient conditions" *Org. Biomol. Chem.* **2024**, *22*, 2175–2181.

[24] S. Erbas-Cakmak, F. P. Cakmak, S. D. Topel, T. B. Uyar, E. U. Akkaya, "Selective photosensitization through an AND logic response: optimization of the pH and glutathione response of activatable photosensitizers" *Chemical Communications* **2015**, *51*, 12258–12261.

[25] M. Ihara, T. Taniguchi, K. Makita, M. Takano, M. Ohnishi, N. Taniguchi, K. Fukumoto, C. Kabuto, "Synthesis of polycyclic cyclobutane derivatives by tandem intramolecular Michael-aldol reaction under two complementary conditions: TBDMSOTf-Et3N and TMSI-(TMS)2NH" *J. Am. Chem. Soc.* **1993**, *115*, 8107–8115.

[26] M. Nakagawa, Y. Matsuki, K. Nagao, H. Ohmiya, "A Triple Photoredox/Cobalt/Brønsted Acid Catalysis Enabling Markovnikov Hydroalkoxylation of Unactivated Alkenes" *J. Am. Chem. Soc.* **2022**, *144*, 7953–7959.

[27] Y. Nishimoto, A. Okita, M. Yasuda, A. Baba, "Synthesis of a Wide Range of Thioethers by Indium Triiodide Catalyzed Direct Coupling between Alkyl Acetates and Thiosilanes" *Org. Lett.* **2012**, *14*, 1846–1849.

[28] J. Roy, T. X. Nguyen, A. K. Kanduluru, C. Venkatesh, W. Lv, P. V. N. Reddy, P. S. Low, M. Cushman, "DUPA Conjugation of a Cytotoxic Indenoisoquinoline Topoisomerase I Inhibitor for Selective Prostate Cancer Cell Targeting" *J. Med. Chem.* **2015**, *58*, 3094–3103.
